# miRNA‐mediated ‘tug‐of‐war’ model reveals ceRNA propensity of genes in cancers

**DOI:** 10.1002/1878-0261.12198

**Published:** 2018-04-17

**Authors:** Arpit Chandan Swain, Bibekanand Mallick

**Affiliations:** ^1^ Department of Mathematics National Institute of Technology Rourkela Odisha India; ^2^ RNAi and Functional Genomics Laboratory Department of Life Science National Institute of Technology Rourkela Odisha India; ^3^ Present address: Department of Biology Utrecht University Utrecht The Netherlands

**Keywords:** ceRNA, genomics, microRNA, RNA‐Seq, SoCeR

## Abstract

Competing endogenous RNA (ceRNA) are transcripts that cross‐regulate each other at the post‐transcriptional level by competing for shared microRNA response elements (MREs). These have been implicated in various biological processes impacting cell‐fate decisions and diseases including cancer. There are several studies that predict possible ceRNA pairs by adopting various machine‐learning and mathematical approaches; however, there is no method that enables us to gauge as well as compare the propensity of the ceRNA of a gene and precisely envisages which among a pair exerts a stronger pull on the shared miRNA pool. In this study, we developed a method that uses the ‘tug of war of genes’ concept to predict and quantify ceRNA potential of a gene for the shared miRNA pool in cancers based on a score represented by SoCeR (score of competing endogenous RNA). The method was executed on the RNA‐Seq transcriptional profiles of genes and miRNA available at TCGA along with CLIP‐supported miRNA‐target sites to predict ceRNA in 32 cancer types which were validated with already reported cases. The proposed method can be used to determine the sequestering capability of the gene of interest as well as in ranking the probable ceRNA candidates of a gene. Finally, we developed standalone applications (SoCeR tool) to aid researchers in easier implementation of the method in analysing different data sets or diseases.

**Abbreviations**
CDH1: E‐cadherin
ceRNA: competing endogenous RNA
circRNA: circular RNA
CLIP: cross‐linking immuno‐precipitation
EPOR: erythropoietin receptor
ERBB2: erb‐b2 receptor tyrosine kinase 2
FOXO1: forkhead box protein
FPKM: fragments per kilobase million
GOI: gene of interest
HCC: hepatocellular carcinoma
HULC: highly upregulated in liver cancer
LAML: acute myeloid leukaemia
lncRNA: long‐noncoding RNA
miRNA: microRNA
MRE: microRNA response elements
mRNA: messenger RNA
NGS: next‐generation sequencing
PCC: probable ceRNA candidate
PTEN: phosphatase and tensin homolog
RPKM: reads per kilobase million
SoCeR: score of competing endogenous RNA
TBA: total binding affinity
TCGA: The Cancer Genome Atlas
TSG: tumour suppressor gene
VCAN: versican
ZEB1, ZEB2: zinc finger E‐box binding homeobox

## 1 Introduction

Recent studies have unmasked the ability of a transcript to influence the expression of similar or different transcript via sequestration of shared microRNA (miRNA). Such transcripts are named as competing endogenous RNA (ceRNA), which include a mixed pool or individual pools of messenger RNA (mRNA), long‐noncoding RNA (lncRNA), pseudogenes and circular RNA (circRNA) (Cesana *et al*., 2011; Hansen *et al*., 2013; Memczak *et al*., 2013). They regulate each other by competing for the common pool of miRNA(s) (Tay *et al*., 2011) and their shared miRNA response elements (MREs) act as natural molecular sponges for the miRNA (Cazalla *et al*., 2010; Jeyapalan *et al*., 2011; Kloc, 2008; Lee *et al*., 2009; Poliseno *et al*., 2010; Seitz, 2009). miRNA is a noncoding RNA of about 22 nucleotides modulating either degradation or silencing of the transcripts by binding to them (Bartel, 2009; Liu *et al*., 2013; Roy and Mallick, 2017; Samantarrai and Mallick, 2017) preferably on 3^/^UTR. The interactions among the pool of miRNA and their target MREs in a given biological system give rise to an unprecedented complex network of miRNA‐mediated regulations. The rationale for the existence of such a complex network is supported by the facts that (a) each transcript containing multiple MREs can be the target of multiple distinct miRNA (Rennie *et al*., 2016), and (b) each miRNA can have multiple target MREs belonging to the same or different transcripts (Liu *et al*., 2013). This redundancy in the synergy of the miRNA and the transcripts led to the discovery of ceRNA. Salmena and colleagues formally put forward the ceRNA hypothesis in August, 2011 (Salmena *et al*., 2011) that was later on endorsed by several studies demonstrating functions of the ceRNA in various biological processes such as viral infections (Cazalla *et al*., 2010), muscle development (Cesana *et al*., 2011), embryonic stem cell differentiation (Wang *et al*., 2013), angiogenesis (Gao *et al*., 2016a), tumorigenicity (Gao *et al*., 2016b; Jeyapalan *et al*., 2011; Li *et al*., 2014b) and metastases (Li *et al*., 2016; Zheng *et al*., 2015) in different cancers.

Although ceRNA research is in its infancy, emerging evidence suggests that ceRNA can regulate the function of miRNA by acting as oncogenes or tumour suppressors contributing to several tumorigenic processes. Hence, ceRNA are pivotal in understanding the additional dimension of post‐transcriptional gene regulation that will unearth the underlying unknown mechanism of cancers. Among the ceRNA, mRNA have recently emerged as notable ceRNA candidates in some of the cancers as well as in normal cells. The competition between forkhead box protein O1 (FOXO1) and E‐cadherin (CDH1) for miR‐9 was reported wherein FOXO1 acted as a ceRNA resulting in inhibition of metastasis of breast cancer cells by inducing CDH1 expression (Yang *et al*., 2014; Zhou *et al*., 2014). A study reported significant positive correlation between erythropoietin receptor (EPOR) and erb‐b2 receptor tyrosine kinase 2 (ERBB2) levels in breast cancer, both of which are targeted by miR‐125b and behave as ceRNA (Ferracin *et al*., 2013). The ceRNA activity of versican, a chondroitin sulphate proteoglycan present in the extracellular matrix, against fibronectin and CD34 via miR‐133a, miR‐199a, miR‐144 and miR‐431 resulted in the development of hepatocellular carcinoma (Fang *et al*., 2013). A group studying HCT116 colon cancer cells detected KRAS and ZEB2 to regulate and be regulated by PTEN through ceRNA mechanism (Gao *et al*., 2012). PTEN and ZEB2 are also found to modulate each other's expression in melanoma by competing for miR‐181, miR‐92, miR‐25 and miR‐200b (Karreth *et al*., 2011). In addition to these, several lncRNA and pseudogenes have been reported to be involved in different cancers by sponging miRNA. Linc‐RoR function as a ceRNA and impairs miR‐205‐dependent repression of zinc finger E‐box binding homeobox (ZEB1 and ZEB2), aiding in the progression of breast cancer by inducing epithelial mesenchymal transition (EMT) (Hou *et al*., 2014). An oncogenic lncRNA, highly upregulated in liver cancer (HULC) acts as a ceRNA of PRKACB through miR‐372 (Pilyugin and Irminger‐Finger, 2014). PTENP1 and KRAS1P affect the expression levels of their cognate genes, PTEN and KRAS, respectively, by sponging shared miRNA through ceRNA‐mediated mechanisms in carcinomas (Alimonti *et al*., 2010; Poliseno *et al*., 2010; Trotman *et al*., 2003).

Several parameters that determine ceRNA activity have been proposed to mimic the milieu within a cell (Ala *et al*., 2013), which include (a) the common number of miRNA, (b) the number of shared MREs, (c) the concentration levels of ceRNA and miRNA and (d) the binding affinity of miRNA to a transcript. These along with the availability of a plethora of data encouraged researchers to develop computational methods and databases for prediction of the ceRNA. A method named cefinder predicted ceRNA by ranking mRNA according to the number of MREs shared between two mRNA (Sarver and Subramanian, 2012). The predictions of this method are publicly available through a database, ceRDB. The method opened up a new arena of ideas for the scientific community but did not consider some essential variables such as the concentrations of the transcripts and miRNA in the system to predict ceRNA precisely. starBase v2.0 is another database which predicts ceRNA using hypergeometric test (Li *et al*., 2014a) based on the number of common miRNA shared between the transcripts. The Database of Human long‐noncoding RNA (lnCeDB) predicts lncRNA as candidate ceRNA by defining two scores (Das *et al*., 2014). However, this failed to infer whether highly ranked pairs with lower expression values or lowly ranked pairs with higher expression values should be preferred as ceRNA. A similar database, known as Pan‐ceRNADB, predicted only mRNA as ceRNA candidates across 20 cancer types (Xu *et al*., 2015) using hypergeometric test. Although it took into account the correlation between the expressions of the genes, it did not consider other features of ceRNA which makes it less sensitive in making reliable predictions. TraceRNA predicts ceRNA by considering most of the known attributes of the miRNA/mRNA interactions (Flores *et al*., 2014). However, this method did not include miRNA expression, an important parameter while making predictions, which was later considered optionally by its sequel, NetceRNA. Moreover, the utility of NetceRNA was illustrated and evaluated only in breast cancer. SpongeScan (Furio‐Tari *et al*., 2016) is another web interface to predict MREs of lncRNA that act as ceRNA. Apart from being concerned with only lncRNAs, the method talks about individual MREs in contrast to the entire transcript. The most recent addition to this effort is a machine‐learning approach, called CERNIA (ceRNA predIction Algorithm) that considers the density, distribution and energy of the MREs along with the number of common miRNA as well as correlation of the gene expressions (Sardina *et al*., 2017).

At present, there is a lack of methods that enable to predict which of the transcripts in a ceRNA pair will act as the natural miRNA sponge in a particular cancer type. The majority of the available resources simply predict pairs of transcripts along with their shared miRNA and users have to intuit which transcript in the pair sequesters more. To address this issue, we propose a mathematical model with the ability to predict potential ceRNA pairs as well as assign relative sequestering strength to each of the genes in the pair. The model computes SoCeR (score of competitive endogenous RNA) by considering several key target‐binding features of CLIP‐identified miRNA targets, expression profiles of individual miRNA and mRNA as well as correlation in expression between the transcripts obtained from RNA‐Seq data of TCGA. This score tells the user which of the transcripts sharing a miRNA pool is potentially the stronger attractive force and also indicates the strength of the competition between them in the specified cancer type.

## 2 Materials and methods

**Figure 1:**
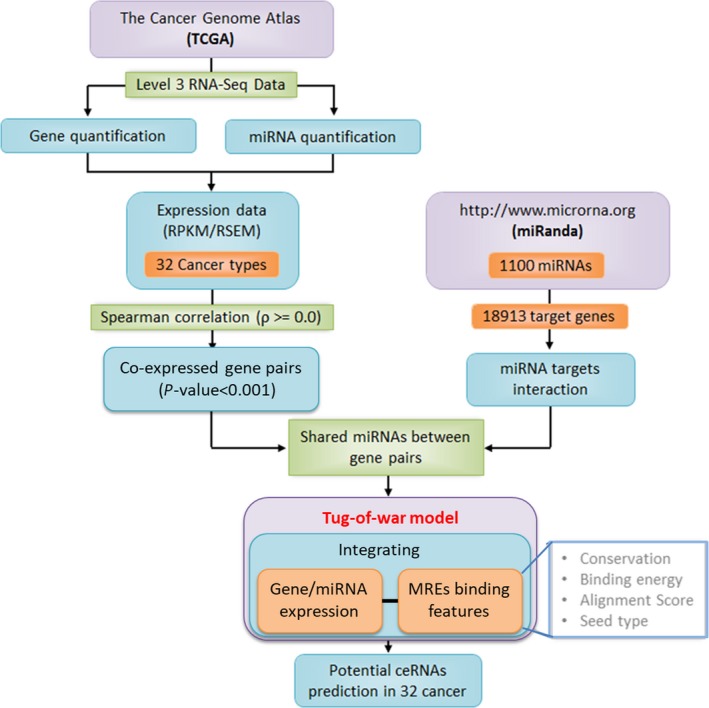
The prediction pipeline incorporating miRNA‐mediated ‘tug of war of genes’ model to predict ceRNA propensity of genes.

We adopted multiple steps and parameters (Fig. 1) to incorporate ceRNA concept into a novel mathematical model to predict ceRNA of a GOI (gene of interest) that share MREs from a set of miRNA expressed in 32 cancer types (Table S1).

### 2.1 Data collection and processing

The expression profile of genes and miRNA of different cancer types were obtained from RNA sequencing (RNA‐Seq) data available at The Cancer Genome Atlas (TCGA). We preferentially selected 32 cancer types for which transcriptional profiles of both genes and miRNA were available at TCGA. Importantly, we used level 3 RNA‐Seq (IlluminaGA and IlluminaHiSeq) data sets for genes as well as for miRNA that have normalized expression values in terms of RPKM/RSEM. These values indicate the abundances of an individual transcript in a sample from the transcriptome reads generated from the sequencing data. We parsed miRNA identifiers of TCGA using miRBase (Release 20) (Kozomara and Griffiths‐Jones, 2014) to get expression profiles of 1100 mature miRNA.

The targets of 1100 miRNA that comprised of 249 conserved and 851 nonconserved human miRNA were downloaded from miRSVR, which is a part of miRanda (http://www.microrna.org) (Betel *et al*., 2008). The target pool includes 2940741 MREs of 20 817 transcripts (18 913 genes), which contains information about its conservation, energy, alignment score and seed type residing on different transcripts. The miRSVR algorithm identifies a significant number of experimentally verified MREs based on a regression model trained on contextual features of CLIP‐Seq predicted target sites (Betel *et al*., 2010). The genes considered for the study included mostly 17 685 (93.51%) mRNA in addition to 218 (1.15%) lncRNA, 166 (0.88%) pseudogenes and 844 (4.46%) miscellaneous RNA (Fig. S1).

### 2.2 Computation of positively correlated genes

An elevated expression of ceRNA leads to increased expression of its paired gene by limiting the amount of shared miRNA bound to the latter. Consequently, genes of a ceRNA pair are positively co‐expressed in a cell (Chiu *et al*., 2015; Sumazin *et al*., 2011; Tay *et al*., 2011). Therefore, we computed the correlation between each pair of genes across all the available samples of cancer to identify preliminary sets of probable ceRNA pairs in them. We considered the transcripts with nonzero expression values (RPKM/RSEM) in a cancer type for correlation analyses. Spearman's correlation coefficient (ρ) was used over that of Pearson's as gene expression profiles of cancers are not normally distributed. Spearman correlation coefficient allows more robustness towards any extreme values in the expression profiles (Mukaka, 2012). We filtered only positively correlated genes with ρ > 0.0. The *P*‐values of all positively correlated pairs were sorted and then FDR corrected. The pairs with corrected *P*‐value ≤ 0.001 were considered in subsequent steps to find potential ceRNA pairs.

### 2.3 miRNA‐mediated ‘tug‐of‐war’ model for predicting ceRNA and their strength

We devised a model based on the ‘tug of war’ of genes for the shared pool of miRNA that can precisely predict the possible ceRNA gene candidates (PCCs) responsible for modulating the expression of co‐expressed genes indirectly through shared miRNA. Dynamic steady state of the cells precisely executes the processes of replication, transcription, and translation in a harmonic manner rejuvenating the cellular environment maintaining defined cellular fate. Here, the dynamic steady state is defined as the varying steady states of the tumour cells in the cancerous tissues. This is based on the observations that the primary tumour after attaining a steady state concentration in due time induces a secondary tumour which also attains a steady state concentration with time (Gatenby, 1995; Nishikawa *et al*., 1994).

**Figure 2:**
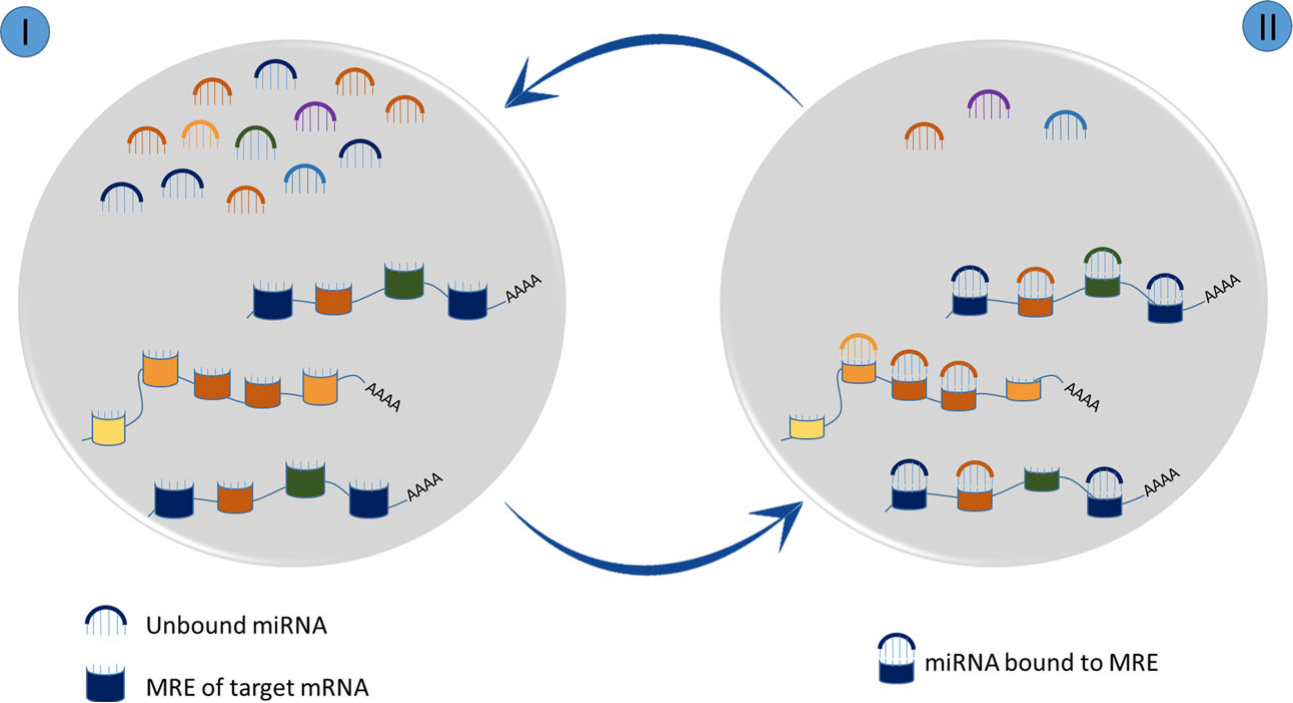
Two proposed steady states with respect to miRNA pool that our system can attain: (I) Shows the unbound state of miRNA and MREs. (II) Shows the bound states of the miRNA that have complementary MREs.

We assume such a steady state system in the model to mimic the ceRNA mechanism in a cell and propose a discrete time model that is characterized by two stages of action (Fig. 2). In the initial stage, there is no interaction between the miRNA and target genes, and hence, the complete pool of miRNA is freely available for binding to different genes in the system. In the next stage, the available miRNA bind to preferred MREs on individual genes according to their inclination and the concentration of individual genes. These stages are recursive because the corresponding cell system will be back to the first stage after completion of the second stage when it is replenished with a fresh pool of unbound miRNA and transcripts. The propensity of miRNA binding to targets can be influenced by (a) relative expression of miRNA and target genes, (b) seed types (8mer, 7mer‐m8, 7mer‐A1, 6mer) (Grimson *et al*., 2007; Liu *et al*., 2013), (c) binding energy of the miRNA‐target duplex, (d) alignment score of miRNA with target at the MRE and (e) conservation score of the MREs (Rennie *et al*., 2016). The seed types considerably influence the competition in binding to mRNA and the potency of repression (Agarwal *et al*., 2015; Chen *et al*., 2015; Denzler *et al*., 2016). Based on this, we define the weights of individual seed types to bring out their importance in calculating ceRNA propensity. We defined the binding affinity (*b*) of a miRNA towards a gene as the product of the features of miRNA‐target duplexes.

(1) b=c∗e∗a∗s

where *c, e, a* and *s* are conservation score, binding energy, alignment score and seed type, respectively. We used a multiplicative combination that would ensure homogeneous changes in the binding affinity with changes in the features, that is proportional changes in the features will result in proportional changes in the binding affinity. The rate of miRNA‐ceRNA target complex formation depends on the concentration and the relative binding affinity of the transcript. The relative binding affinity of a PCC with respect to its GOI ($b_{\mathrm{PCC}|\mathrm{GOI}}$) is defined as:

(2) $$b_{\mathrm{PCC}|\mathrm{GOI}}=\frac{b_{\mathrm{PCC}}*z}{b_{\mathrm{GOI}}+b_{\mathrm{PCC}}}$$

where *z* is the coefficient of relative binding affinity and is found to be 2 as *b*_g1|g1_ should be 1. An advantage of calculating the relative binding affinity is to avoid the bias introduced by the different scales and their extreme values.

In our miRNA‐sponging model, we have integrated the expression values of targets with above target‐binding features, which will be useful to break the tie in cases of similar binding affinities. The expression value, *E*_PCC_, used is the average of the expressions of the PCC taken from all the samples. This term is termed as the total binding affinity (TBA) of a gene (denoted as B) and is used to predict PCCs of a GOI.

(3) $$B_{\mathrm{PCC}|\mathrm{GOI}}=\sum_{\mathrm{PCC}\;\mathrm{MREs}}E_{\mathrm{PCC}}*\frac{b_{\mathrm{PCC}}*2}{b_{\mathrm{GOI}}+b_{\mathrm{PCC}}}$$

Here, PCC MREs are the MREs present on the PCC. The TBA of a PCC with respect to the GOI is the cumulative of the relative binding affinity of the MREs present on all the transcripts of the PCC. TBA is a measure of the rate of the complex formation expressed as the product of expression levels (*E*) and affinity of a gene towards a miRNA. Further, we have also included the expressions of miRNA in the model as it is a crucial factor in influencing the rate of degradation or inhibition of the genes. *E*_miRNA_ is defined as the average of all the miRNA samples.

As the system is in a steady state, the time taken for formation of the duplex by both the genes of a ceRNA pair is equal. Therefore,

(4) $$\frac{x}{B_{\mathrm{PCC}|\mathrm{GOI}}}=\frac{E_{\mathrm{miRNA}}-x}{B_{\mathrm{GOI}|\mathrm{PCC}}}$$

Here, *x* is the part of the miRNA pool that is sequestered due to the TBA of the PCC. The equation (4) is reminiscent of the ‘tug of war’ between GOI and PCC for their shared miRNA with respect to their TBAs. In equation (4), the denominators are the rates at which the miRNA form duplexes while the numerators are the expression levels of miRNA. Thus, values on left‐hand side and right‐hand side of the equation (4) symbolize the time taken for formation of complex with PCC and GOI, respectively. We defined our score, SoCeR, by solving equation (4) and calculating the differences between the amount of miRNA binding to PCCs and GOIs. Mathematically, we present SoCeR (non‐normalized) as:

(5) $$\sum_{\mathrm{All}\;\mathrm{miRNA}}E_{\mathrm{miRNA}}*\left[\frac{\sum_{\mathrm{PCC}\;\mathrm{MREs}}E_{\mathrm{PCC}}*b_{\mathrm{PCC}}-\sum_{\mathrm{GOI}\;\mathrm{MREs}}E_{\mathrm{GOI}}*b_{\mathrm{GOI}}}{\sum_{\mathrm{PCC}\;\mathrm{MREs}}E_{\mathrm{PCC}}*b_{\mathrm{PCC}}+\sum_{\mathrm{GOI}\;\mathrm{MREs}}E_{\mathrm{GOI}}*b_{\mathrm{GOI}}}\right]$$

Theoretically, equation (5) gives the excess (or deficit) of amount of miRNA binding to PCC over GOI of a ceRNA pair sharing MREs, which is mathematically represented by a positive (or negative) score. The scores lie between −1 and +1 by normalizing with the highest absolute score for a particular GOI. A negative score implies that the GOI sequesters a higher number of miRNA in the pair, and thus, the GOI has higher ceRNA propensity in that system with respect to the same pool of miRNA. Similarly, a positive score suggests that the predicted gene has higher ceRNA propensity in that particular system. The magnitude of the score indicates the imbalance in sequestering strength of the genes of a ceRNA pair. A magnitude close to unity suggests the case of ceRNA dominance, while a score closer to zero (or exactly zero) indicates a state of ceRNA dependency (or no ceRNA activity) of the genes in the cell. The terms ceRNA domination and dependency are defined with respect to the power of individual ceRNA to influence the system. A dominant ceRNA can strongly influence its partner gene and thus, the system, whereas a dependent ceRNA has a weak influence on its partner gene and the combined effect of the pair is needed to influence the system. These extreme cases show a clear rift in the strength of the genes symbolizing weak ceRNA regulation either due to the inability of a gene to compete or due to the similarity in strength of the two genes to sponge miRNA.

## 3 Results and Discussion

Here, we have proposed a novel miRNA‐mediated ‘tug‐of‐war’ sponging model and a well‐defined score (SoCeR), which can accurately interpret all possible conditions while predicting ceRNAs in a system. Standalone applications, SoCeR (for Windows as well as Linux), have been developed to assist the scientists in performing similar analysis on different data sets and systems and is available at http://vvekslab.in/tools.html. Hereon forth, we use the notation GOI–PCC for the ceRNA pairs. SoCeR not only gives a measure of the sequestering capability of the GOI but can also be used for ranking the PCCs of a GOI. For example, in breast cancer, PTEN‐PTENP1 is ranked 48th, and PTENP1‐PTEN is ranked 4th when their ceRNA are sorted in ascending and descending manner, respectively. To test the robustness of SoCeR, we swapped the GOI and PCC of a ceRNA pair. As expected, we obtained a non‐normalized score of the same magnitude but of opposite sign predicting the same conclusion as before. This ascertains the sturdiness of the score. Although the non‐normalized SoCeR for a pair can be obtained by multiplying negative unity to the same of the opposite pair, it is not true for SoCeR as the latter gives the relative strength of a pair with respect to all of the ceRNA pairs of its GOI. Refer Table 1 to compare SoCeR of PTEN‐PTENP1 and PTENP1‐PTEN predicted across all cancers. Our model is further validated by considering different cases and interpreting their scores. We tested cases with respect to three possible conditions in a cell: (a) expression of miRNA is zero; (b) either or both, expression and affinity of a gene is zero; and (c) total binding affinity of a gene is greater than its ceRNA counterpart. The case with no miRNA in the system gives an obvious score of zero because a common pool of miRNA is absent, and hence, there would be no influence on either the genes or the system. The second condition can be regarded as a case of complete ceRNA dominance due to the inability of binding or unavailability of the other genes. The last case, as expected, gives a positive score if the PCC has a greater total binding affinity (than the GOI) symbolizing that more miRNA would be sequestered by the PCC. The above evaluations reveal SoCeR as a dependable score. Refer to Table S2 that enlists all the experimentally validated ceRNA predicted by our model in different cancers.

**Table 1:** The ceRNA propensity of PTEN & PTENP1 predicted in 31 cancer types

Cancer type	Correlation coefficient	*P*‐Value	No. of Common miRNA	SoCeR (PTEN‐PTENP1)	SoCeR (PTENP1‐PTEN)
ACC	0.847	0	121	−0.67369	0.78630
BLCA	0.858	7.70E‐293	130	−0.50920	0.72302
BRCA	0.933	5.46E‐207	127	−0.67049	0.88367
CESC	0.872	0	130	−0.54521	0.83129
CHOL	0.862	0	117	−0.55465	0.73192
COAD	0.944	0	125	−0.40175	0.77568
DLBC	0.834	8.50E‐30	121	−0.50075	0.65581
ESCA	0.717	7.80E‐137	129	−0.45532	0.89090
HNSC	0.942	0	131	−0.47273	0.68261
KICH	0.8	0	117	−0.81225	0.81477
KIRC	0.96	0	122	−0.65480	0.89554
KIRP	0.81	0	125	−0.66183	0.90289
LGG	0.713	0	129	−0.28003	0.94196
LIHC	0.815	0	129	−0.46025	0.60944
LUAD	0.921	0	125	−0.54618	0.70292
LUSC	0.956	0	124	−0.52097	0.72832
MESO	0.877	0	123	−0.77569	0.74926
OV	0.841	0	128	−0.37445	0.95353
PAAD	0.85	0	123	−0.59451	0.68933
PCPG	0.781	0	125	−0.81538	0.90834
PRAD	0.807	0	128	−0.28020	0.82334
READ	0.943	0	118	−0.35608	0.73590
SARC	0.862	0	128	−0.52238	0.79559
SKCM	0.925	0	132	−0.63347	0.83782
STAD	0.83	0	127	−0.35283	0.88958
TGCT	0.722	5.33E‐128	130	−0.51230	0.76446
THCA	0.772	2.04E‐286	134	−0.53311	0.77455
THYM	0.932	0	128	−0.55396	0.91472
UCEC	0.963	0	128	−0.60520	0.78189
UCS	0.702	0	126	−0.72531	0.78462
UVM	0.941	0	122	−0.46564	0.64366

### 3.1 ceRNA profile across 32 cancers

**Figure 3:**
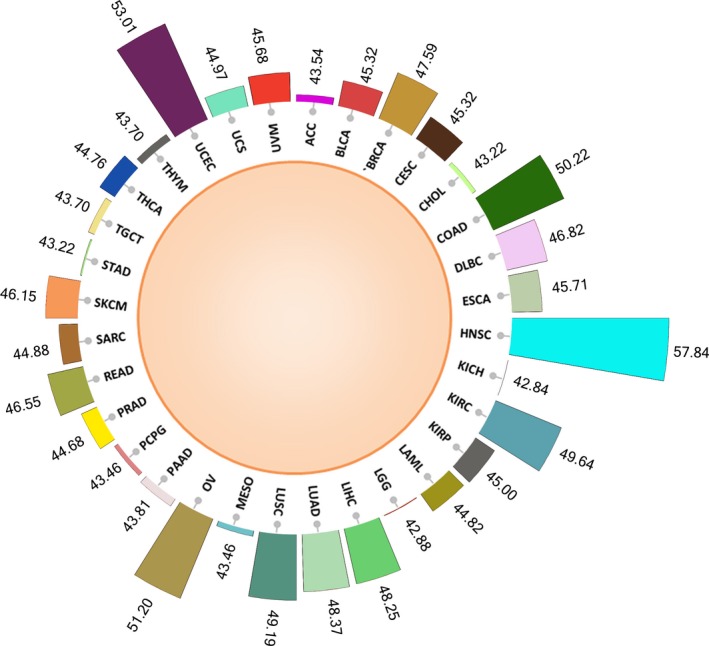
The percentage of total ceRNA pairs (out of all possible pairs) predicted by our method in individual cancer types.

The profiles of ceRNA across 32 cancers were predicted using our ‘tug‐of‐war’ model. We observed the highest percentage of ceRNA predicted in HNSC with 57.84% of all possible pairs followed by UCEC (53.01%) and OV (51.20%). Figure 3 gives the distribution of the percentage of ceRNA predicted in individual cancer types. From this, we can infer that mRNA‐related ceRNA activity is highest in HNSC among all cancers, whereas lowest ceRNA activity is in KICH (42.84%). Interestingly, we noticed that our method can predict ceRNA that are already reported and validated in cancers (Table S2), which indicate the reliability of our method for predicting ceRNA of any GOI in any other cancers. Table 2 lists some of these pairs that can be justified in their sequestration strength. We have discussed some of these cases below in detail.

**Table 2:** The list of already reported and validated ceRNA predicted by our method

Cancer	ceRNA pair	miRNA	Correlation	*P*‐Value	SoCeR	References
PRAD	PTEN‐PTENP1	hsa‐miR‐19b, hsa‐miR‐20a	0.807	0	−0.28020	(He *et al*., 2015)
COAD	PTEN‐CNOT6L	hsa‐miR‐17, hsa‐miR‐19a, hsa‐miR‐19b, hsa‐miR‐20a, hsa‐miR‐20b, hsa‐miR‐106b	0.702	0	−0.01492	(Qu *et al*., 2015)
COAD	PTEN‐VAPA	hsa‐miR‐20a, hsa‐miR‐26b	0.545	0	0.02606	(Qu *et al*., 2015)
PRAD	PTEN‐CNOT6L	hsa‐miR‐19a, hsa‐miR‐19b, hsa‐miR‐20a	0.412	0	−0.00507	(Tay *et al*., 2011)
LIHC	VCAN‐CD34	hsa‐miR‐431	0.416	0	0.05405	(Fang *et al*., 2013)
SKCM	PTEN‐ZEB2	hsa‐miR‐92a, hsa‐miR‐200b, hsa‐miR‐25	0.19	0	−0.09792	(Karreth *et al*., 2011)
ESCA	PTEN‐PTENP1	hsa‐miR‐130b	0.717	7.80E‐137	−0.45532	(Yu *et al*., 2015)
ESCA	PHLPP2‐IFT88	hsa‐miR‐224	0.538	0	−0.02266	(He *et al*., 2015)
ESCA	PHLPP2‐ZNF91	hsa‐miR‐224	0.526	0	0.01319	(He *et al*., 2015)

### 3.2 Case studies

#### 3.2.1 The ceRNA networks of PTEN

PTEN has been the subject of investigation by many researchers (Ioffe *et al*., 2012; Poliseno *et al*., 2010; Song *et al*., 2012; Sumazin *et al*., 2011; Yu *et al*., 2014) due to its tumour suppressing abilities across almost all known cancers that regulate the PI3K/AKT signalling pathway. The ceRNA network involving PTEN has been recorded in some cancers and speculated for others. The expression of PTEN is reported to be regulated by its pseudogene ceRNA, PTENP1 via their shared miRNA at the post‐transcriptional level. Influenced by these, we used our model to find possible cases of the ceRNA crosstalks between PTEN and PTENP1 across 32 cancers (Table 1). We noticed that SoCeR is not only dependent on the number of common miRNA but also on the binding site features of the shared miRNA which are evident in the cancer types such as CHOL and KICH. Both the cancers have the same number of common miRNA shared between PTEN and PTENP1, yet CHOL endorses PTENP1 as a stronger ceRNA candidate of PTEN than KICH. Another befitting comparison that supported the above argument is the case of CHOL and READ. We observed that the absolute score for PTEN and PTENP1 is lesser in READ as compared to CHOL although a higher number of miRNA are shared in the former. Further, PTEN‐PTENP1 pair was found to be strongly positively correlated in all cancers except acute myeloid leukaemia (LAML) where the correlation was found to be negative. Table S2 lists out the experimentally validated cases of PTEN interactions that have been predicted by our method.

PTEN and CNOT6L predicted as a ceRNA pair by our method gives a negative score. This can be explained by the observations of Tay *et al*. (2011) which stated that a change in CNOT6L changes the expression levels of PTEN significantly, while a change in the expression levels of PTEN brings about significantly lesser change in CNOT6L. Thus, it can be concluded that CNOT6L sequesters majority of the common pool of miRNA as indicated by our score. Further, our model has predicted several other mRNA that may function as PTEN ceRNA and regulate PTEN expression in some of the cancers, which have already been reported experimentally elsewhere (Karreth *et al*., 2011; Poliseno *et al*., 2010; Qu *et al*., 2015; Tay *et al*., 2011; Yu *et al*., 2015). One such case is repression of PTEN by the oncomir miR‐130b in oesophageal squamous cell carcinoma, which increases the proliferation and migration abilities of the tumour cells (Yu *et al*., 2015). SoCeR provides a negative score for the interaction of PTEN (GOI) and PTENP1 in this cancer mediated through miR‐130b.

In summary, the widespread occurrence of PTEN‐PTENP1 ceRNA network in almost all cancers (Table 1) predicted in our study indicated a common regulatory activity of the tumour suppression pathway. Moreover, this case study authenticated our tug‐of‐war model by predicting already validated cases of the PTEN‐centred ceRNA in cancers. Table S3 report the scores of all possible ceRNA of PTEN in breast cancer. Table S4 reports the shared list of miRNA along with their scores for the PTEN‐PTENP1 interaction in breast cancer.

#### 3.2.2 miRNA sponging by TSGs

While a cell in its normal condition is a quintessential blend of genes, the tumour‐afflicted cell is the result of dysregulation of tumour suppressors among other reasons. SoCeR gives a measure of the miRNA‐sponging ability (MSA) of a gene. A negative score would represent a higher MSA of the GOI or query gene. Similarly, a cumulative negative SoCeR would symbolize an overall downregulation of a gene in cancer on account of sequestering more miRNA than its counterparts. We selected three well‐known TSGs: PTEN, TP53 and RB1 to infer the fate of their tumour suppressing activities in all 32 cancers. We obtained a negative score for these TSGs, propelling us to hypothesize that miRNA sponging involving TSGs could be a possible event promoting the formation of cancer cells. It is evident from Table 3 that PTEN, TP53 and RB1 are important TSGs active across all cancers as speculated.

**Table 3:** The cumulative, mean and standard deviation (std) of the SoCeR of three well‐known TSGs: PTEN, TP53 and RB1 in 32 cancer types

Cancer	PTEN (mean) [std]	TP53 (mean) [std]	RB1 (mean) [std]
ACC	−838.77 (−0.091) [0.138]	−776.97 (−0.089) [0.151]	−872.57 (−0.093) [0.172]
BLCA	−1022.17 (−0.113) [0.150]	−883.14 (−0.101) [0.176]	−691.08 (−0.075) [0.172]
BRCA	−1045.34 (−0.106) [0.166]	−1557.58 (−0.132) [0.194]	−1963.48 (−0.193) [0.188]
CESC	−1186.79 (−0.118) [0.142]	−1113.33 (−0.115) [0.201]	−1278.69 (−0.132) [0.176]
CHOL	−975.56 (−0.092) [0.151]	−892.87 (−0.103) [0.205]	−919.29 (−0.091) [0.201]
COAD	−1134.33 (−0.105) [0.171]	−711.20 (−0.095) [0.156]	−1756.53 (−0.164) [0.169]
DLBC	−1312.99 (−0.140) [0.161]	−1113.58 (−0.133) [0.234]	−1370.16 (−0.137) [0.172]
ESCA	−862.23 (0.089) [0.135]	−1033.71 (−0.103) [0.183]	−976.30 (−0.112) [0.169]
HNSC	−955.08 (−0.095) [0.145]	−1757.28 (−0.125) [0.191]	−1793.16 (−0.193) [0.201]
KICH	−685.38 (−0.078) [0.165]	−864.40 (−0.089) [0.165]	−811.64 (−0.088) [0.201]
KIRC	−797.63 (−0.070) [0.150]	−1641.21 (−0.121) [0.183]	−2001.63 (−0.191) [0.213]
KIRP	−973.08 (−0.090) [0.200]	−788.89 (−0.086) [0.203]	−1284.95 (−0.129) [0.241]
LAML	−1338.54 (−0.166) [0.184]	−1207.01 (−0.117) [0.153]	−780.82 (−0.094) [0.164]
LGG	−1016.99 (−0.118) [0.234]	−994.71 (−0.114) [0.156]	−901.15 (−0.097) [0.181]
LIHC	−930.57 (−0.113) [0.153]	−1127.02 (−0.104) [0.163]	−899.09 (−0.080) [0.179]
LUAD	−906.70 (−0.083) [0.161]	−1173.81 (−0.120) [0.186]	−1362.52 (−0.158) [0.184]
LUSC	−905.92 (−0.098) [0.163]	−1413.73 (−0.132) [0.183]	−1885.41 (−0.179) [0.184]
MESO	−1108.92 (−0.127) [0.169]	−876.76 (−0.094) [0.185]	−1038.54 (−0.106) [0.178]
OV	−1035.39 (−0.106) [0.163]	−1520.83 (−0.137) [0.183]	−771.76 (−0.068) [0.195]
PAAD	−1358.64 (−0.126) [0.153]	−760.73 (−0.082) [0.167]	−1076.41 (−0.104) [0.178]
PCPG	−899.26 (−0.102) [0.158]	−880.78 (−0.091) [0.154]	−953.47 (−0.090) [0.194]
PRAD	−952.23 (−0.096) [0.166]	−783.22 (−0.111) [0.189]	−711.18 (−0.075) [0.211]
READ	−1096.93 (−0.094) [0.165]	−1156.39 (−0.125) [0.187]	−1631.73 (−0.145) [0.163]
SARC	−891.89 (−0.096) [0.149]	−1069.33 (−0.108) [0.167]	−518.40 (−0.061) [0.181]
SKCM	−1268.73 (−0.131) [0.162]	−1001.21 (−0.111) [0.159]	−1375.65 (−0.138) [0.176]
STAD	−1013.40 (−0.095) [0.150]	−828.68 (−0.099) [0.165]	−857.04 (−0.085) [0.169]
TGCT	−983.33 (−0.103) [0.140]	−1121.44 (−0.115) [0.170]	−405.37 (−0.039) [0.150]
THCA	−1100.37 (−0.119) [0.170]	−970.63 (−0.098) [0.151]	−893.63 (−0.103) [0.181]
THYM	−1116.61 (−0.133) [0.153]	−825.62 (−0.113) [0.151]	−1187.69 (−0.119) [0.187]
UCEC	−1025.59 (−0.083) [0.145]	−1976.21 (−0.163) [0.194]	−1981.18 (−0.165) [0.184]
UCS	−974.15 (−0.109) [0.163]	−946.54 (−0.115) [0.180]	−1032.58 (−0.092) [0.175]
UVM	−1757.38 (−0.161) [0.192]	−598.21 (−0.093) [0.139]	−1452.45 (−0.127) [0.226]

#### 3.2.3 Putative PTEN ceRNA regulate PTEN in human melanoma cells

Expression levels of PTEN can be modulated through miRNA by putative PTEN ceRNA (Karreth *et al*., 2011). The study by Karreth *et al*., 2011 selected nine well‐known PTEN ceRNA and silenced them in two different melanoma cell lines which resulted in attenuation of PTEN expression. We considered these nine candidates and pitted them individually against PTEN through our model. SoCeR of most of these pairs were found to be positive, revealing the ceRNA candidates have higher sequestering capability compared to PTEN (Table S2). This substantiates our model to be capable of predicting reported cases of ceRNA.

#### 3.2.4 VCAN‐CD34/FN1 ceRNA promoting oncogenesis

VCAN was investigated by Fang *et al*. (2013) to explore its role in HCC. It was found to promote the proliferation, survival, migration and invasion of the cells by modulating the activities of miR‐133a, miR‐199a*, miR‐144 and miR‐431 which were sequestered by mRNA such as CD34 and FN1. Our method predicted VCAN‐CD34 and VCAN‐FN1 to be co‐expressed and thus initially assumed as potential ceRNA pairs. Further, we noticed a positive SoCeR for both of these pairs with VCAN as the GOI, which indicated VCAN to be a stronger miRNA sponge compared to its partners. This observation supported the findings by Fang *et al*. about VCAN freeing CD34 and FN1 for protein translation through ceRNA mechanism by miR‐431 sponging and promoting HCC. The other reported miRNA were absent in the mediation of the interactions in question as our method is dependent on data generated by other predictive tools such as mirSVR. Table S5 and S6 contain the scores of the shared pool of miRNA between VCAN‐CD34 and VCAN‐FN1, respectively.

#### 3.2.5 EPOR and ERBB2 cooperatively regulate malignancy

Transfection of miR‐125b by Ferracin *et al*. (2013) confirmed EPOR and ERBB2 crosstalk with each other through ceRNA mechanism. miR‐125b is a well‐known tumour suppressor miRNA which has reduced expression in metastatic breast cancer. Ferracin *et al*. showed ERBB2 to act as a decoy of miR‐125b and induce the expression of EPOR by reducing the amount of free cellular miR‐125b. Our method also predicted ERBB2 to be the major miRNA sponge with a low positive score in breast cancer besides nine other cancers, for several miRNA but not miR‐125b. Ferracin *et al*. realized the inability of ERBB2 alone to explain the sustenance of the malignant behaviour of breast cancer cells which was solved by including EPOR into the pool as ceRNA crosstalks. Thus, to maintain the malignancy, both the transcripts are functioning cooperatively. This brings out the true essence of our score as the low magnitude of SoCeR suggests a dependent behaviour of the ceRNA in regulating the cell state.

### 3.3 Comparison of predictions and threshold of SoCeR

**Figure 4:**
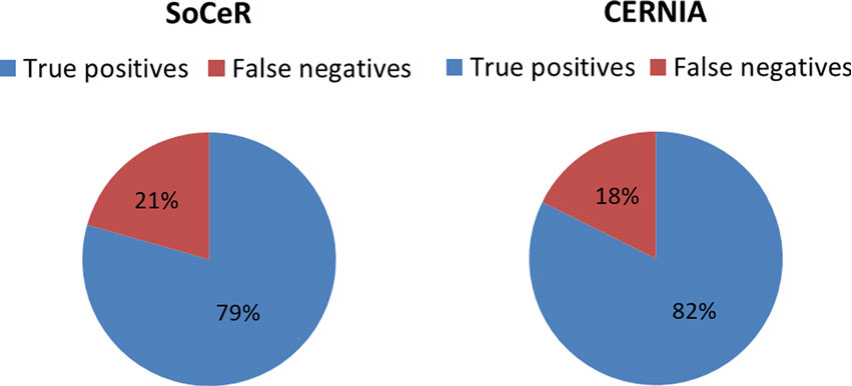
Pie charts showing the distribution of true positives and false negatives in the predictions of SoCeR and CERNIA.

The primary objective of our model is to predict the ceRNA propensity of the genes among the positively correlated pairs in a system. Moreover, it can also be used to predict ceRNA. Due to the unavailability of the negative class (putative non‐ceRNA), we could not perform the receiver operating characteristic analysis to calculate sensitivity and specificity of our method. Alternatively, we computed the number of true positives (TP) and false negatives (FN) of our predictions. The validated sets of 34 ceRNA reported in three cancers (breast, prostate and brain) reported in the literature (Poliseno *et al*., 2010; Sardina *et al*., 2017; Sumazin *et al*., 2011; Tay *et al*., 2011) were used for comparison by calculating TP and FN. Of these, our method correctly predicted 27 pairs, while CERNIA, the most recent method which has been reported to outperform previous methods predicted 28 pairs (Fig. 4). This indicates our method is equally competitive with other methods. However, availability of more number of validated ceRNA in different cancers in future will reinforce this comparison. We have shown the statistics on the classes of genes in the predicted ceRNA pairs in Fig. S2 for five cancers.

In order to define a range of SoCeR that could be used as a threshold to find significant ceRNA pairs, we considered the validated ceRNA predicted by our model as well as CERNIA and assessed their SoCeRs. We recommend the minimum and maximum of the absolute values of SoCeR of these validated ceRNAs as the threshold. Therefore, we recommend the pairs whose absolute SoCeRs lie in the range of [0.00403, 0.88367]. The number of predicted pairs are cut down by a third when these cut‐offs are enforced. Table S7 gives the percentages of the predicted pairs that fall in the suggested range.

## 4 Conclusions

In the current study, we devised a miRNA‐sponging model for predictions of ceRNA of a GOI in 32 cancer types to complement the resources available for ceRNA, which is still in its nascent stage of research. This model is motivated by the lack of algorithms or tools for predicting which of the transcripts with shared miRNA will be the most potential ceRNA by defining SoCeR scores that range between −1 and 1. To the best of our knowledge, our model is the only model that is specialized to envisage potential of each transcript sharing the same set of miRNA/MREs to behave as ceRNA based on sequence, thermodynamic and conservation features of miRNA‐target duplexes as well as cancer‐specific co‐expression patterns of genes and expression levels of shared miRNA. This method revealed the sponging ability of each of the transcripts expressed in a particular cancer type whose expression levels are obtained from RNA‐Seq data available at TCGA. Further, our method includes targets of both conserved and nonconserved human miRNA predicted from Ago interaction sites of CLIP‐Seq studies integrated into mirSVR of miRanda which enhances the reliability of our predictions by reducing the false miRNA targets in our miRNA‐target duplex data sets. The method can be used for effective retrieval of ceRNA of GOI in specific cancer as well as in multiple cancers which will enable the researchers to compare ceRNA candidates of GOI in selected cancers and validate whether similar or dissimilar ceRNA regulatory mechanisms exist in these cancers. This will help to decipher common oncogenic processes operated by the same set of ceRNA in multiple cancers and will thus offer new opportunities to manipulate these common ceRNA networks through miRNA competition for their therapy, perhaps by adopting one strategy.

Our method demonstrated some limitations because of which some of the ceRNA reported previously by other labs were not detected by our method. Some of these are due to the dependency of our method on data retrieved from other resources such as TCGA and mirSVR. For example, our method failed to predict FOXO1‐CDH1 as ceRNA reported earlier by Yang *et al*. (Yang *et al*., 2014). While investigating the reason for this miss, we surprisingly found their expressions to be negatively correlated. This is possibly due to anomalies in the expression data of these genes obtained from TCGA, which have prevented our method to predict these genes as a potential ceRNA pair. Moreover, we missed some miRNA in our prediction for few ceRNA pairs which are due to the absence of these miRNA in CLIP‐verified targets available at mirSVR or TCGA.

As discussed previously, the ceRNA, although in its infancy, has the potential to regulate several biological processes, both in normal and cancer cells, and hence provides a new perspective to understand oncogenic processes. Thus, understanding the regulatory mechanisms and functions exhibited by them is very essential. Our method will help the researchers to gain an in‐depth insight into the myriad roles of ceRNA language in diverse cancer types and devise strategies for miRNA loss‐of‐function studies as a therapeutic measure.

## Author contributions

ACS carried out the experiments, data analysis and drafted the manuscript. BM conceived the idea, designed the study, drafted the manuscript and supervised this study. All authors reviewed and approved the final manuscript.

## Supporting information


**Fig. S1.** The overview of different classes of genes considered in our study.
**Fig. S2.** The overview of different classes of ceRNA predicted by our model in five different cancers.
**Table S1**. The abbreviations of cancer types considered in our study.
**Table S2**. List of experimentally validated ceRNA predicted by our model in different cancers.
**Table S3**. The probable ceRNA of PTEN predicted by our model in BRCA.
**Table S4**. List of shared miRNA of PTEN‐PTENP1 pairs in BRCA along with their respective SoCeR.
**Table S5**. The shared miRNA of VCAN‐CD34 pairs in LIHC along with their corresponding scores.
**Table S6**. The shared miRNA of VCAN‐FN1 pair in LIHC along with their corresponding scores.
**Table S7**. The percentage of predicted pairs lying in the cut‐off range across cancers.
