# Supplementary material for: miRNA‐mediated ‘tug‐of‐war’ model reveals ceRNA propensity of genes in cancers
Source: Mol Oncol. 2018 Apr 17;12(6):855–68. doi: 10.1002/1878-0261.12198 (PMC5983123; doi:10.1002/1878-0261.12198)
Supplement: Supplementary file 1 — Fig. S1. The overview of different classes of genes considered in our study. Fig. S2. The overview of different classes of ceRNA predicted by our model in five different cancers. Table S1. The abbreviations of cancer types considered in our study. Table S2. List of experimentally validated ceRNA predicted by our model in different cancers. Table S3. The probable ceRNA of PTEN predicted by our model in BRCA. Table S4. List of shared miRNA of PTEN‐PTENP1 pairs in BRCA along with their respective SoCeR. Table S5. The shared miRNA of VCAN‐CD34 pairs in LIHC along with their corresponding scores. Table S6. The shared miRNA of VCAN‐FN1 pair in LIHC along with their corresponding scores. Table S7. The percentage of predicted pairs lying in the cut‐off range across cancers. [file MOL2-12-855-s001.pdf]

Supporting Information

miRNA-mediated 'tug-of-war' model reveals ceRNA propensity of genes in cancers

Arpit Chandan Swain[1,3] and Bibekanand Mallick[2,*]

[1]Department of Mathematics, National Institute of Technology, Rourkela, Odisha, India. [2]RNAi and Functional Genomics Laboratory, Department of Life Science, National Institute of Technology, Rourkela, Odisha, India. [3]Present address: Department of Biology, Utrecht University, Padualaan 8, 3584 CH Utrecht, The Netherlands.

[*]*Corresponding author*.

Dr. B. Mallick

E-mail: vivek.iitian@gmail.com, mallickb@nitrkl.ac.in

Fax: +91-661-2472926; Tel: +91-661-2462685

Running Title: ceRNA propensity of genes in cancers

Figure S1. The overview of different classes of genes considered in our study

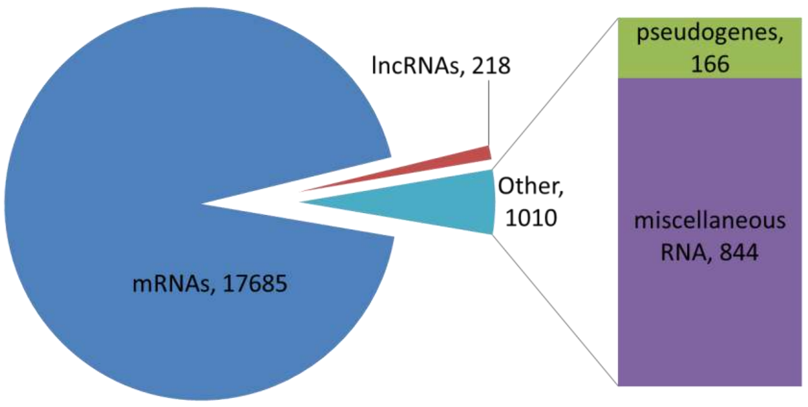

Figure S2. The overview of different classes of ceRNAs predicted by our model in five different cancers.

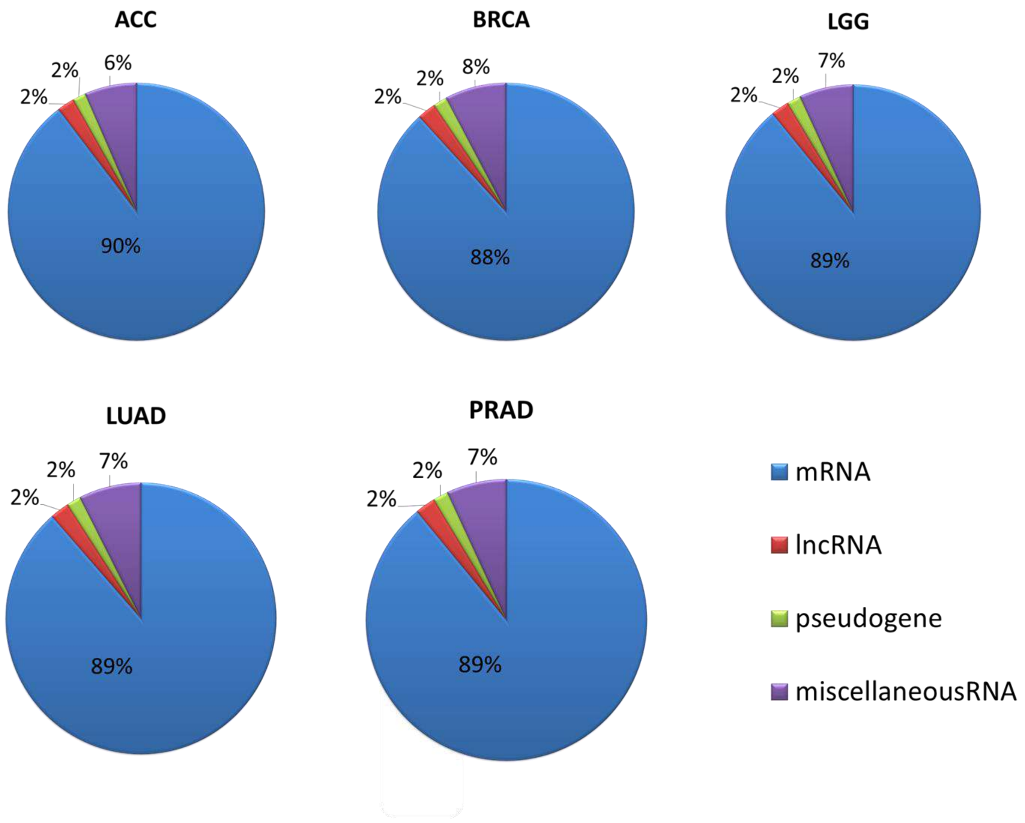

Table S1: The abbreviations of cancer types considered in our study.

Cancer Abbreviations	Expanded Form
ACC	Adrenocortical Carcinoma
BLCA	Bladder Urothelial Carcinoma
BRCA	Breast Invasive Carcinoma
CESC	Cervical Squamous Cell Carcinoma and Endocervical Adenocarcinoma
CHOL	Cholangiocarcinoma
COAD	Colon Adenocarcinoma
DLBC	Lymphoid Neoplasm Diffuse Large B-cell Lymphoma
ESCA	Esophageal Carcinoma
HNSC	Head and Neck Squamous Cell Carcinoma
KICH	Kidney Chromophobe
KIRC	Kidney Renal Clear Cell Carcinoma
KIRP	Kidney Renal Papillary Cell Carcinoma
LAML	Acute Myeloid Leukemia
LGG	Brain Lower Grade Glioma
LIHC	Liver Hepatocellular Carcinoma
LUAD	Lung Adenocarcinoma
LUSC	Lung Squamous Cell Carcinoma
MESO	Mesothelioma
OV	Ovarian Serous Cystadenocarcinoma
PAAD	Pancreatic Adenocarcinoma
PCPG	Pheochromocytoma and Paraganglioma
PRAD	Prostate Adenocarcinoma
READ	Rectum Adenocarcinoma
SARC	Sarcoma
SKCM	Skin Cutaneous Melanoma
STAD	Stomach Adenocarcinoma
TGCT	Testicular Germ Cell Tumors

THCA	Thyroid Carcinoma
THYM	Thymoma
UCEC	Uterine Corpus Endometrial Carcinoma
UCS	Uterine Carcinosarcoma
UVM	Uveal Melanoma

Table S2: List of experimentally validated ceRNAs predicted by our model.

Cancer	Gene1	Gene2	SoCeR	Reference
BRCA	CD44	CDC42	-0.06178	21149267
BRCA	CD44	COL1A1	-	22637644
BRCA	CD44	FN1	-	22637644
BRCA	VCAN	PTEN	0.12017	21049042
BRCA	VCAN	RB1	0.25194	21049042
BRCA	EPOR	ERBB2	0.00183	24165569
COAD	PTEN	CNOT6L	-0.01492	PMC4694204
COAD	PTEN	VAPA	0.02606	PMC4694204
ESCA	PHLPP2	IFT88	-0.02266	26245343
ESCA	PHLPP2	NEK3	0.00635	26245343
ESCA	PHLPP2	ZNF91	0.01319	26245343
ESCA	PTEN	PTENP1	-0.45532	25637514
KICH	VCAN	PTEN	0.39043	21049042
KICH	VCAN	RB1	0.17195	21049042
KIRC	VCAN	PTEN	0.05059	21049042
KIRC	VCAN	RB1	0.41294	21049042
KIRP	VCAN	PTEN	-0.30916	21049042
KIRP	VCAN	RB1	-0.27477	21049042
LIHC	HULC	PRKACB	-	20423907
LIHC	VCAN	CD34	0.05405	23180826
LIHC	VCAN	FN1	0.07237	23180826
LUAD	VCAN	PTEN	0.25957	21049042
LUAD	VCAN	RB1	0.32622	21049042
LUSC	VCAN	PTEN	0.29291	21049042
LUSC	VCAN	RB1	-	21049042
LGG	ABHD13	PTEN	0.59004	22000015
LGG	CCDC6	PTEN	-0.07527	22000015
LGG	CTBP2	PTEN	-0.05644	22000015
LGG	DCLK1	PTEN	-0.2436	22000015
LGG	DKK1	PTEN	0.65524	22000015
LGG	HIF1A	PTEN	-0.21296	22000015
LGG	KLF6	PTEN	0.57095	22000015
LGG	LRCH1	PTEN	0.16919	22000015
LGG	NRAS	PTEN	-	22000015
LGG	PTEN	NCOA7	-0.09297	22000013
LGG	PTEN	PDGFRA	0.47234	22000015
LGG	PTEN	RB1	-0.07384	22000015
LGG	PTEN	RUNX1	-	22000015
LGG	PTEN	SERINC1	-0.18699	22000013

LGG	PTEN	STAT3	-	22000015
LGG	PTEN	VAPA	0.46485	22000013
LGG	PTEN	VEGFA	-	22000015
LGG	PTEN	ZNF460	-0.0605	22000013
LGG	SRY	HIF1A	-	23446346
LGG	TAF5	PTEN	0.18533	22000015
LGG	TNKS2	PTEN	-0.20014	22000015
PRAD	CNOT6L	PTEN	0.0403	22000013
PRAD	PTEN	NCOA7	-0.09596	22000013
PRAD	PTEN	PTENP1	-0.2802	20577206
PRAD	PTEN	SERINC1	0.00403	22000013
PRAD	PTEN	ZNF460	-0.49162	22000013
PRAD	VAPA	PTEN	-0.21951	22000013
SKCM	PTEN	AFF1	0.08001	22000016
SKCM	PTEN	CNOT6L	0.00186	22000016
SKCM	PTEN	DCBLD2	0.06463	22000016
SKCM	PTEN	JARID2	-	22000016
SKCM	PTEN	MBNL1	0.24046	22000016
SKCM	PTEN	RBM9	0.05735	22000016
SKCM	PTEN	TNRC6a	-	22000016
SKCM	PTEN	ZEB2	-0.09792	22000016

Table S3: The probable ceRNAs of PTEN predicted by our model in BRCA.

Query gene	ceRNA	Correlation Co-efficient	p-Value	SoCeR
PTEN	FOXF1	2.89E-05	0	-0.213560413
PTEN	C15orf60	5.14E-05	0	-0.053030524
PTEN	SLC10A1	7.87E-05	0	-0.164177871
PTEN	NOD1	8.57E-05	0	-0.120728116
PTEN	TBC1D9B	0.000106	0	-0.098269414
PTEN	GPR110	0.000109	0	-0.160117966
PTEN	CCDC73	0.000113	0	-0.390979422
PTEN	ALOXE3	0.000136	0	-0.282310299
PTEN	RAMP2	0.000145	0	-0.00013257
PTEN	MGC23270	0.000195	0	-0.013440748
PTEN	C2orf84	0.000241	0	-0.116564534
PTEN	NEUROG3	0.000242	0	-0.000241
PTEN	ELOVL4	0.000261	0	-0.585786953
PTEN	DDX53	0.000295	0	-0.050119967
PTEN	C15orf43	0.000303	0	-0.200828803
PTEN	ZNF793	0.000317	0	-0.15852714
PTEN	RFXAP	0.000386	0	-0.162218764
PTEN	TRIM42	0.000411	0	-0.025163229
PTEN	CDK8	0.000411	0	-0.084035987
PTEN	BMP2	0.000416	0	-0.181426423
PTEN	TMED6	0.000414	0	-0.099530344
PTEN	CD300E	0.000427	0	-2.88E-08
PTEN	ZNF623	0.000447	0	-0.025083006
PTEN	HSFX1	0.000458	0	-0.00100439
PTEN	MESDC1	0.000462	0	-0.050506018
PTEN	WSB2	0.000466	0	-0.246318465
PTEN	TOLLIP	0.000509	0	-0.008185146
PTEN	C9orf144	0.00051	0	-0.183345184
PTEN	ZMYND17	0.000516	0	-0.215273282
PTEN	PTPRF	0.000523	0	-0.009147662
PTEN	POU3F4	0.000591	0	-0.034795552
PTEN	RNASE2	0.000611	0	-0.010418149
PTEN	COL18A1	0.000643	0	0.002535396
PTEN	PLIN5	0.000641	0	8.20E-05
PTEN	C2orf18	0.000677	0	-0.028495387
PTEN	PRPF18	0.000753	0	-0.028583319
PTEN	HEMK1	0.000763	0	-0.005933044
PTEN	LELP1	0.000777	0	-0.169114698

PTEN	CLCA4	0.000782	0	-0.361950131
PTEN	TMEM126B	0.000847	0	-0.034688861
PTEN	NFATC1	0.00085	0	-0.135802508
PTEN	LCE3C	0.000854	0	-7.21E-05
PTEN	ASB16	0.000916	0	-0.230222231
PTEN	CXCR4	0.00096	0	0.013567672
PTEN	AKAP1	0.000979	0	0.024519997
PTEN	HNRNPH3	0.000982	0	-0.10978702
PTEN	AMAC1	0.000994	0	-0.174180279
PTEN	TXLNB	0.00101	0	-0.550870636
PTEN	WNT4	0.00101	0	-0.451663864
PTEN	SLC2A12	0.00102	0	-0.480694098
PTEN	TGIF2LX	0.00102	0	-0.001306458
PTEN	IL5	0.00103	0	-0.038145194
PTEN	BRD4	0.0011	0	-0.173047711
PTEN	NOMO3	0.0012	0	0.001840041
PTEN	GP6	0.00124	0	-0.154954729
PTEN	TUBD1	0.00126	0	-0.208066203
PTEN	GABARAPL1	0.00127	0	-0.003557861
PTEN	THBS3	0.00132	0	-1.01E-06
PTEN	ASFMR1	0.00133	0	-0.711717739
PTEN	CNTN5	0.00134	0	-0.219909468
PTEN	MPP1	0.00135	0	-0.10647466
PTEN	KBTBD5	0.00139	0	-0.000485095
PTEN	CCR2	0.00139	0	-0.462597288
PTEN	RPL11	0.00142	0	1.18E-05
PTEN	KCNH7	0.00142	0	-0.190989839
PTEN	THRSP	0.00143	0	-0.157386962
PTEN	ZNF530	0.00147	0	-0.168376678
PTEN	ADARB1	0.00147	0	0.009211751
PTEN	JRKL	0.00157	0	-0.429407423
PTEN	NELL1	0.0016	0	-0.01938659
PTEN	SNORD116-12	0.00162	0	-0.004211348
PTEN	NOM1	0.00162	0	-0.047776597
PTEN	PARP15	0.00164	0	-0.13411192
PTEN	RAG1AP1	0.00167	0	0.015128361
PTEN	BAALC	0.00167	0	-0.243576217
PTEN	PRM1	0.00169	0	-0.00071629
PTEN	CSF3R	0.00173	0	-0.013137461
PTEN	ARHGEF33	0.00174	0	-0.331078712
PTEN	HLA-DQA2	0.00176	0	-0.061227571

PTEN	HEMGN	0.00177	0	-0.38060587
PTEN	ACBD4	0.00179	0	-0.102636521
PTEN	DQX1	0.00184	0	-0.009493677
PTEN	ZP1	0.00184	0	-2.47E-05
PTEN	RAB37	0.00186	0	-0.305178667
PTEN	HIATL2	0.00187	0	-0.019083881
PTEN	LASS3	0.00188	0	-0.181535548
PTEN	SEZ6L2	0.00188	0	-0.006385675
PTEN	SLC25A25	0.00192	0	0.018676888
PTEN	C1orf201	0.00195	0	-0.169915612
PTEN	NCRNA00119	0.00198	0	-0.593782316
PTEN	LHX4	0.00198	0	-0.010789304
PTEN	BOK	0.00199	0	-0.000304914
PTEN	IQCG	0.002	0	-0.001038237
PTEN	C21orf49	0.00201	0	-0.170322196
PTEN	RPL36AL	0.00203	0	9.57E-06
PTEN	CCDC116	0.00205	0	-0.003803313
PTEN	RBBP7	0.00206	0	0.175698886
PTEN	PHC1	0.00214	0	-0.019660962
PTEN	GNB2L1	0.00213	0	0.004255961
PTEN	MRPL10	0.00219	0	-0.050679677
PTEN	SLC17A8	0.00222	0	-0.237337677
PTEN	MTRF1	0.00223	0	-0.110287251
PTEN	LGI1	0.00224	0	0.063013663
PTEN	LOC286135	0.00229	0	-0.572323593
PTEN	APEH	0.00233	0	-0.001676874
PTEN	SNORD113-9	0.00233	0	-1.38E-05
PTEN	TDGF1	0.00234	0	-0.209876413
PTEN	FMNL2	0.00235	0	-0.229360953
PTEN	ABCA2	0.0024	0	-0.000688096
PTEN	WDR3	0.00243	0	0.027296018
PTEN	FAM106A	0.00249	0	-0.257810432
PTEN	PNRC1	0.00249	0	-0.160457982
PTEN	TSHR	0.00253	0	-0.613778054
PTEN	RBMXL3	0.00261	0	-0.009974139
PTEN	AMZ1	0.00263	0	-0.265197063
PTEN	ETS2	0.00265	0	-0.354046594
PTEN	KRTAP5-9	0.00268	0	-0.104633848
PTEN	NXF5	0.00272	0	-0.021376119
PTEN	LOC100272228	0.00275	0	-0.001257867
PTEN	TMOD1	0.00275	0	-0.672891677

PTEN	LOC100271832	0.00278	0	-0.190323697
PTEN	GABRB1	0.00278	0	-0.333313236
PTEN	SLC22A8	0.00283	0	-0.002054196
PTEN	WFDC13	0.00283	0	-0.043047586
PTEN	TMEM14E	0.00284	0	-0.472634509
PTEN	AIG1	0.0029	0	-0.150075522
PTEN	DNM1L	0.0029	0	-0.089127123
PTEN	CLC	0.00291	0	-8.30E-05
PTEN	RHOQ	0.00291	0	-0.063044243
PTEN	PPARA	0.00295	0	1.16E-06
PTEN	B9D2	0.00299	0	-0.002241825
PTEN	FABP2	0.003	0	-0.353526841
PTEN	FXYD3	0.00306	0	0.101526409
PTEN	UBA2	0.00308	0	-0.0160222
PTEN	GABRG2	0.00309	0	-0.513955755
PTEN	SPINT3	0.00309	0	-0.008963005
PTEN	SLC25A29	0.00316	0	-0.14512801
PTEN	AMHR2	0.00317	0	-0.001006923
PTEN	TUBAL3	0.00317	0	-0.03207783
PTEN	ZNF643	0.00318	0	-0.000148306
PTEN	TMEM8B	0.00318	0	-0.009499417
PTEN	KSR1	0.0032	0	-0.073782421
PTEN	FAM92A1	0.0032	0	-0.065130499
PTEN	RALYL	0.00325	0	-0.159660142
PTEN	CHST11	0.00326	0	-0.166425184
PTEN	DCTN2	0.00329	0	-0.002192129
PTEN	CD160	0.00337	0	-0.187026069
PTEN	FGFR1	0.00338	0	0.246768136
PTEN	C10orf54	0.0034	0	-0.01236296
PTEN	C1orf74	0.0034	0	-0.011424025
PTEN	STK10	0.00341	0	-0.424543328
PTEN	MPL	0.00345	0	-0.528023245
PTEN	FAM81A	0.00345	0	-0.15387965
PTEN	ZNRF4	0.00347	0	-0.039908624
PTEN	GALNTL4	0.00348	0	-0.166896416
PTEN	FAM123C	0.00348	0	-0.037380788
PTEN	CNR1	0.0035	0	-0.462522391
PTEN	FBXO33	0.00351	0	-0.557692089
PTEN	TMEM229A	0.00352	0	-0.401415455
PTEN	RTL1	0.00356	0	-2.81E-05
PTEN	ZNF3	0.00361	0	-0.06016976

PTEN	FAM83G	0.00363	0	0.001665483
PTEN	POPDC2	0.00368	0	-0.212199742
PTEN	DGKE	0.00369	0	-0.281773497
PTEN	MAGEH1	0.00373	0	-0.049015767
PTEN	PROZ	0.00377	0	-0.175625783
PTEN	SNORD115-8	0.00381	0	-0.000142747
PTEN	UBQLN3	0.00385	0	-0.01401427
PTEN	AKAP8	0.00391	0	-0.200430283
PTEN	DMTF1	0.00392	0	0.123896763
PTEN	NKD2	0.00394	0	5.25E-05
PTEN	SPINK8	0.00395	0	-0.001635654
PTEN	SCD	0.00399	0	0.333427537
PTEN	LOC100131691	0.00404	0	-0.007004657
PTEN	SCML4	0.00405	0	-0.284767336
PTEN	BEX1	0.00407	0	0.152542275
PTEN	ADARB2	0.00407	0	-0.170003065
PTEN	PGBD1	0.00419	0	-0.172273879
PTEN	EFHB	0.00421	0	-0.241045333
PTEN	HOXC10	0.00423	0	-0.042566516
PTEN	PLIN2	0.00428	0	-0.189633333
PTEN	LBX1	0.00429	0	-0.04011258
PTEN	HOXD11	0.00431	0	-0.001592644
PTEN	EDN2	0.00434	0	-0.149210665
PTEN	NCRNA00200	0.00434	0	-0.100574723
PTEN	EID2B	0.00439	0	-0.105167939
PTEN	LCE6A	0.00449	0	-0.000171223
PTEN	NFIX	0.00452	0	-0.005503091
PTEN	TSPY3	0.00454	0	-1.40E-08
PTEN	IL1RN	0.00455	0	-0.000639283
PTEN	KRT80	0.00455	0	-0.115715012
PTEN	PAR1	0.00456	0	-0.212914094
PTEN	PPP3R1	0.00457	0	-0.124089268
PTEN	ZNF761	0.0046	0	-0.104871016
PTEN	RYR3	0.00463	0	-0.364560222
PTEN	C17orf75	0.00466	0	-0.306560555
PTEN	CHRNA3	0.00467	0	-0.251491794
PTEN	GABRR1	0.00468	0	-0.189536839
PTEN	C4orf35	0.00475	0	-0.212654902
PTEN	SDHD	0.00476	0	-0.004462077
PTEN	GLS	0.00479	0	-0.063474886
PTEN	PLVAP	0.0048	0	-0.011842435

PTEN	DCLK3	0.00481	0	-0.629672709
PTEN	SPZ1	0.00482	0	-0.169209249
PTEN	LOC100132288	0.00483	0	-0.001902417
PTEN	SNORD113-4	0.00484	0	-0.000152065
PTEN	DUSP13	0.00485	0	-0.009733702
PTEN	FAM90A7	0.00486	0	-7.03E-05
PTEN	PFDN1	0.00487	0	0.000179133
PTEN	ACTG2	0.00498	0	-4.47E-06
PTEN	MTFMT	0.005	0	-0.149329293
PTEN	MAPK8IP2	0.00501	0	0.002167912
PTEN	TNFAIP8L1	0.00501	0	-0.147578527
PTEN	PSG4	0.00504	0	-0.012691974
PTEN	CAPZB	0.00505	0	-0.145572651
PTEN	IRS4	0.00508	0	-0.007328474
PTEN	ABCG5	0.00508	0	-0.235231819
PTEN	ARHGEF16	0.00509	0	-0.001022142
PTEN	SYCP3	0.0051	0	-0.133179037
PTEN	FLCN	0.00512	0	-0.120579433
PTEN	CSHL1	0.00513	0	-0.000803765
PTEN	EDN1	0.00516	0	-0.002972028
PTEN	RWDD1	0.00517	0	-0.057399053
PTEN	PRAMEF20	0.00518	0	-0.001884153
PTEN	EDDM3B	0.00521	0	-0.011548922
PTEN	CD3G	0.00521	0	-0.224125461
PTEN	GPR125	0.0054	0	0.031954953
PTEN	SLCO1A2	0.00546	0	-0.194257134
PTEN	C14orf70	0.00548	0	-0.284067337
PTEN	C11orf49	0.00553	0	-0.024157304
PTEN	NEIL2	0.00554	0	0.152642468
PTEN	CD207	0.00557	0	-0.108947511
PTEN	FAM57B	0.00572	0	-0.238831483
PTEN	PRAMEF16	0.0058	0	-0.000114589
PTEN	SNORD116-27	0.00587	0	-0.164090176
PTEN	RPS27A	0.00586	0	-3.65E-05
PTEN	ZNF525	0.00587	0	-0.027921302
PTEN	CIDEC	0.00589	0	-0.274567808
PTEN	WBP5	0.0059	0	0.032230499
PTEN	POLM	0.00589	0	-0.110591533
PTEN	UGP2	0.00591	0	0.099403405
PTEN	CAMK2G	0.00593	0	-0.169256825
PTEN	ENPP6	0.00598	0	-0.022877018

PTEN	AP1G2	0.00599	0	-0.040189109
PTEN	STATH	0.00599	0	-0.062468374
PTEN	AMZ2	0.00599	0	-0.00352112
PTEN	RUSC2	0.00601	0	-0.146638999
PTEN	NICN1	0.00602	0	-0.02249509
PTEN	C4orf22	0.00613	0	-0.003824656
PTEN	TRIM45	0.00613	0	-0.1467952
PTEN	PHKA2	0.00613	0	0.000194567
PTEN	RTKN2	0.00618	0	-0.05941183
PTEN	ZNF732	0.00618	0	-0.011596371
PTEN	IQCF3	0.00618	0	-0.000112969
PTEN	MLXIPL	0.00619	0	-0.144836339
PTEN	CCT2	0.00621	0	0.074438549
PTEN	HMX1	0.00621	0	-0.000119911
PTEN	MAGEA1	0.00624	0	-0.308659563
PTEN	HEXA	0.00626	0	0.125016182
PTEN	SLC25A31	0.00627	0	-0.395051762
PTEN	APCDD1L	0.00629	0	-0.135479536
PTEN	SCPEP1	0.0063	0	-0.012802639
PTEN	UGT1A10	0.0063	0	-0.138350037
PTEN	NFRKB	0.00633	0	-0.000801325
PTEN	RCSD1	0.00634	0	-0.06402565
PTEN	RAET1E	0.00637	0	-9.79E-08
PTEN	LHX6	0.0064	0	-0.269838284
PTEN	LRRC28	0.00641	0	-0.019292603
PTEN	CWC25	0.00641	0	-0.158425112
PTEN	GPR183	0.00641	0	-0.202454403
PTEN	WFDC1	0.00644	0	-0.000596486
PTEN	ELMOD3	0.00645	0	-0.06682582
PTEN	THNSL2	0.00646	0	-0.010386919
PTEN	IYD	0.00647	0	-0.040530049
PTEN	AP3M2	0.0065	0	-0.135570659
PTEN	RAB20	0.00651	0	-0.149456002
PTEN	ZNF622	0.00653	0	-3.26E-05
PTEN	PDCD1LG2	0.00656	0	-0.252104201
PTEN	EMILIN2	0.00659	0	-0.039813994
PTEN	KRTAP10-5	0.00661	0	-0.000147762
PTEN	ZNF256	0.00665	0	-5.81E-07
PTEN	SLC17A1	0.00666	0	-0.017559026
PTEN	CSTF3	0.00667	0	-0.095940632
PTEN	TRIM64	0.0067	0	-0.005792865

PTEN	FLJ37307	0.00674	0	-0.059510098
PTEN	RHOC	0.00674	0	0.01534557
PTEN	FLJ40292	0.00677	0	-0.132674733
PTEN	SPIN2A	0.00679	0	-0.188198599
PTEN	GPM6B	0.00679	0	-0.160725109
PTEN	TSSK3	0.00683	0	-0.000103075
PTEN	ADAM29	0.00689	0	-0.000563164
PTEN	NPM1	0.00693	0	0.26095827
PTEN	RAB11FIP3	0.00694	0	-0.015304279
PTEN	BLMH	0.00695	0	0.011450407
PTEN	APOA5	0.00706	0	-0.171831603
PTEN	SIAH1	0.00709	0	0.012669376
PTEN	NCRNA00052	0.0071	0	-0.669656439
PTEN	LGALS9	0.00713	0	-0.019612112
PTEN	MED20	0.00714	0	0.127809387
PTEN	SBDS	0.00714	0	0.112026263
PTEN	C1QTNF1	0.00716	0	-0.068973375
PTEN	KRTAP9-9	0.00718	0	-0.015146671
PTEN	ALDH7A1	0.00724	0	0.026915845
PTEN	C1orf210	0.00726	0	-0.048776709
PTEN	FAM134A	0.00727	0	-0.182669654
PTEN	PROP1	0.00727	0	-0.007644618
PTEN	MGST2	0.00728	0	-0.001072882
PTEN	SMYD1	0.00735	0	-0.183499342
PTEN	UBQLNL	0.00735	0	-0.179753281
PTEN	TMPRSS4	0.00737	0	-0.155545157
PTEN	ZPBP	0.00738	0	-0.041202402
PTEN	MYCL1	0.0074	0	-0.205036349
PTEN	SPATA19	0.0074	0	-0.000701891
PTEN	LOC145820	0.00743	0	-0.33524613
PTEN	TBC1D21	0.00742	0	-0.039899732
PTEN	LOC653786	0.00743	0	-0.000325871
PTEN	C8orf58	0.00748	0	-0.069359959
PTEN	ACOT9	0.0075	0	-0.001492741
PTEN	CCDC141	0.00754	0	-0.439411074
PTEN	GK3P	0.00759	0	-2.98E-05
PTEN	UIMC1	0.00758	0	-0.000546847
PTEN	TARS	0.00764	0	0.029516524
PTEN	KLRC3	0.00764	0	-0.002202185
PTEN	C1orf43	0.00768	0	0.065674794
PTEN	BCL9L	0.00773	0	-0.005526568

PTEN	ZDHHC4	0.00775	0	-0.000110332
PTEN	PRKACG	0.00777	0	-0.006324412
PTEN	HOXA11AS	0.00777	0	-0.000579399
PTEN	SNORD124	0.00781	0	-4.12E-05
PTEN	WDR81	0.00782	0	-0.044328575
PTEN	TDH	0.00787	0	-0.346216414
PTEN	C5orf56	0.00787	0	-0.213990787
PTEN	RDH5	0.00788	0	-0.060443711
PTEN	DMBX1	0.00789	0	-0.0227924
PTEN	ODF2	0.00789	0	-0.008947705
PTEN	TTC29	0.0079	0	-0.00507924
PTEN	GNL3L	0.00791	0	-0.00727302
PTEN	DTHD1	0.00793	0	-0.007181882
PTEN	C17orf72	0.00793	0	4.87E-05
PTEN	RNF128	0.00793	0	-0.342466306
PTEN	LOC55908	0.00797	0	-0.000154225
PTEN	TRMT61B	0.00798	0	-0.250487417
PTEN	RRN3P1	0.00798	0	-0.163742764
PTEN	KCNN2	0.00804	0	-0.488423441
PTEN	SNORD121B	0.00806	0	-0.001049577
PTEN	VASH2	0.00811	0	-0.780492621
PTEN	PTGES	0.00814	0	-0.007764929
PTEN	IDH3A	0.00814	0	0.006920097
PTEN	ASAP2	0.00818	0	-0.190889983
PTEN	C1orf93	0.00824	0	0.001678085
PTEN	EFCAB4A	0.00826	0	-0.075969104
PTEN	SSX3	0.00826	0	-0.259952591
PTEN	KDELR2	0.00829	0	0.002220106
PTEN	CD209	0.0083	0	-0.147049177
PTEN	CYB561D2	0.00834	0	-0.056372973
PTEN	DNAH1	0.00835	0	-0.000108713
PTEN	ZNF22	0.0084	0	-0.225088931
PTEN	IFNA2	0.00841	0	-0.03035486
PTEN	PNMAL2	0.00841	0	-0.009472742
PTEN	NFATC2IP	0.00853	0	-0.04022731
PTEN	SIK1	0.00856	0	-0.491900266
PTEN	NSUN7	0.0086	0	-0.103409675
PTEN	MTAP	0.00861	0	-0.160133687
PTEN	TTTY19	0.00865	0	-0.002821106
PTEN	CHMP4A	0.00873	0	0.019157223
PTEN	SNAR-A2	0.00875	0	-7.20E-07

PTEN	H2AFY2	0.00881	0	-0.029183605
PTEN	PRO1768	0.00881	0	-0.229238831
PTEN	GANAB	0.00885	0	0.003446097
PTEN	PYGM	0.00889	0	-0.094129683
PTEN	TNFRSF21	0.0089	0	-0.141554301
PTEN	GRM3	0.00891	0	-0.724588788
PTEN	CAND2	0.00891	0	-0.032996081
PTEN	SPACA3	0.00893	0	-0.000110394
PTEN	AGPAT9	0.00895	0	-0.357730727
PTEN	RAI1	0.00898	0	-0.245962483
PTEN	ALG8	0.009	0	-0.003573408
PTEN	LOC401127	0.00902	0	-0.14874851
PTEN	LOC654433	0.00902	0	-0.098732288
PTEN	L1TD1	0.00906	0	-0.002622239
PTEN	NPC1L1	0.00909	0	-0.000461411
PTEN	GPR146	0.0091	0	-0.034781787
PTEN	WT1	0.0091	0	-0.174596709
PTEN	LRRTM1	0.0091	0	-0.656651913
PTEN	ZNF711	0.00916	0	-0.061184166
PTEN	TMEM202	0.00919	0	-0.501769669
PTEN	RPL15	0.00919	0	0.155505238
PTEN	PCP2	0.00922	0	-0.003751202
PTEN	GCHFR	0.00923	0	-1.17E-06
PTEN	SRY	0.00922	0	-0.022251606
PTEN	LOC642852	0.00922	0	4.71E-05
PTEN	IL3RA	0.00923	0	-0.046885703
PTEN	APOL1	0.00924	0	0.003284769
PTEN	INSM1	0.00925	0	-0.227094831
PTEN	C4orf26	0.0093	0	-0.169740334
PTEN	TRIM21	0.00932	0	-0.002099417
PTEN	CXorf56	0.00941	0	-0.000120848
PTEN	SNRNP40	0.00941	0	-0.017914701
PTEN	SIRPA	0.00945	0	-0.051083415
PTEN	MSN	0.00945	0	0.155202564
PTEN	HSFYL1	0.00948	0	-0.577424223
PTEN	IGJ	0.0095	0	0.066984549
PTEN	CYP3A43	0.00951	0	-0.033018237
PTEN	KIAA1161	0.00953	0	-0.165473867
PTEN	F5	0.00953	0	-0.276774226
PTEN	SLC45A3	0.00955	0	-0.000476471
PTEN	PEG3AS	0.00955	0	-0.264063527

PTEN	KIF11	0.00956	0	0.023126763
PTEN	RAB42	0.00958	0	0.000377838
PTEN	FGF3	0.00964	0	-0.037166324
PTEN	LOC727924	0.00966	0	-0.166248884
PTEN	LAT2	0.00967	0	-0.005464649
PTEN	CCDC43	0.00967	0	0.181355408
PTEN	KDM4D	0.00974	0	-0.029347173
PTEN	ZNF69	0.00976	0	-0.241260193
PTEN	BTC	0.00981	0	-0.026938264
PTEN	CCDC7	0.00983	0	-0.006519099
PTEN	C2orf53	0.00985	0	-0.000277336
PTEN	GK	0.00986	0	-0.267504322
PTEN	NARS	0.00987	0	-0.008189587
PTEN	FOLH1B	0.00988	0	-0.014962925
PTEN	TSEN15	0.00988	0	0.03813542
PTEN	STT3A	0.0099	0	-0.004676593
PTEN	GPR62	0.00997	0	-0.440133971
PTEN	C9orf131	0.00998	0	-9.86E-05
PTEN	WFDC8	0.01	0	-0.207898059
PTEN	RPL3	0.0101	0	0.000262358
PTEN	GPR64	0.0101	0	-0.584090119
PTEN	SIL1	0.0102	0	0.000433497
PTEN	C2orf39	0.0102	0	-6.09E-05
PTEN	SUMO1P3	0.0102	0	-0.138114869
PTEN	SERPINB7	0.0103	0	-0.044653755
PTEN	TXNDC11	0.0103	0	-0.186684442
PTEN	RMND5B	0.0103	0	0.001587999
PTEN	BTBD16	0.0103	0	-0.000196755
PTEN	HCP5	0.0104	0	-0.055439199
PTEN	ARMCX6	0.0104	0	-0.122067903
PTEN	VPS52	0.0104	0	-0.001236025
PTEN	MYADM	0.0104	0	0.00743329
PTEN	PER1	0.0104	0	-0.048867155
PTEN	LOC643955	0.0104	0	-0.02046182
PTEN	XAF1	0.0104	0	-0.010121098
PTEN	EZR	0.0105	0	-0.061171736
PTEN	LRRC2	0.0105	0	-0.24119308
PTEN	ASB5	0.0106	0	-0.241259408
PTEN	FAM115C	0.0106	0	-0.006398105
PTEN	DEFA5	0.0106	0	-0.210061109
PTEN	OXT	0.0107	0	-3.86E-07

PTEN	FAM82A2	0.0107	0	-0.131939927
PTEN	ATP5A1	0.0107	0	-0.140516832
PTEN	LOC201651	0.0107	0	-0.170608185
PTEN	NBN	0.0108	0	-0.10343448
PTEN	SIGLEC1	0.0108	0	-0.167315179
PTEN	FOXE1	0.0108	0	-0.147480838
PTEN	C21orf57	0.0108	0	-0.020291261
PTEN	TEKT4	0.0108	0	-3.79E-06
PTEN	RPTN	0.0109	0	-0.349396287
PTEN	VANGL2	0.0109	0	-0.012034737
PTEN	LRRC8D	0.011	0	0.004476601
PTEN	ATP6V1G1	0.011	0	0.112890638
PTEN	LOC84740	0.0111	0	-0.151987434
PTEN	KRTAP3-2	0.0111	0	-0.156174255
PTEN	APP	0.0111	0	0.175606181
PTEN	POU1F1	0.0111	0	-0.041349706
PTEN	C21orf63	0.0111	0	-0.004656547
PTEN	C3orf32	0.0111	0	-0.010904872
PTEN	MFAP2	0.0112	0	0.003355767
PTEN	LGALS12	0.0112	0	-0.087985205
PTEN	DCBLD1	0.0112	0	9.90E-05
PTEN	LOC100129935	0.0113	0	-0.248367054
PTEN	STX6	0.0113	0	0.049657242
PTEN	LYSMD1	0.0113	0	-0.24386306
PTEN	INTS3	0.0113	0	-0.014670747
PTEN	CTAGE4	0.0114	0	-0.00385483
PTEN	ZNF556	0.0114	0	-3.26E-05
PTEN	EXT1	0.0114	0	0.149177422
PTEN	ELAC1	0.0115	0	-0.081242512
PTEN	ZNF137	0.0115	0	-0.180513248
PTEN	ING2	0.0115	0	-0.072572652
PTEN	ZFATAS	0.0115	0	-0.119892426
PTEN	ZNF485	0.0115	0	-0.077608901
PTEN	ACE	0.0115	0	-0.093090808
PTEN	CASP3	0.0116	0	0.017609668
PTEN	HTR1E	0.0116	0	-0.002671331
PTEN	ABHD10	0.0117	0	-0.041156622
PTEN	C18orf19	0.0117	0	-0.290302676
PTEN	AFARP1	0.0117	0	-0.095994579
PTEN	XGPY2	0.0117	0	-0.32688687
PTEN	LRRC41	0.0117	0	-0.025597618

PTEN	RAB26	0.0117	0	-0.116987876
PTEN	GCNT2	0.0118	0	-0.150927571
PTEN	SPO11	0.0118	0	-0.226505994
PTEN	ISX	0.0118	0	-0.086142719
PTEN	KIAA1539	0.0118	0	-0.021866993
PTEN	IPCEF1	0.0119	0	-0.184557416
PTEN	HPSE	0.0119	0	-0.000106571
PTEN	VTRNA1-1	0.0119	0	-2.04E-08
PTEN	EIF1AY	0.0119	0	-0.229617937
PTEN	CAPN3	0.0119	0	-0.004779877
PTEN	FAM133B	0.0119	0	-0.09570351
PTEN	GNG3	0.0119	0	-0.000291306
PTEN	C17orf42	0.0119	0	0.131680261
PTEN	USP35	0.012	0	-0.002195439
PTEN	SBDSP1	0.012	0	-0.051048786
PTEN	DHRS7C	0.012	0	-3.87E-07
PTEN	C6orf146	0.0121	0	-0.164955956
PTEN	MST4	0.0121	0	-0.386098726
PTEN	MEOX1	0.0121	0	-0.132560748
PTEN	C16orf3	0.0121	0	-0.000825044
PTEN	TRPC5	0.0122	0	-0.344527438
PTEN	MUM1	0.0122	0	-0.142799496
PTEN	NCRNA00176	0.0123	0	-0.174154433
PTEN	C12orf71	0.0123	0	-0.000485443
PTEN	FLJ37543	0.0123	0	-0.502770272
PTEN	SHC3	0.0123	0	-0.031570661
PTEN	CLDN2	0.0123	0	-0.196306072
PTEN	PDCD7	0.0124	0	-0.134607733
PTEN	LAPTM5	0.0124	0	0.111005148
PTEN	KIF24	0.0124	0	-0.028333942
PTEN	KLRG1	0.0124	0	-0.331108674
PTEN	MLANA	0.0124	0	-0.03733556
PTEN	MOBP	0.0125	0	-0.447874956
PTEN	PET112L	0.0126	0	-0.015520473
PTEN	RAD51L1	0.0126	0	-0.298446671
PTEN	ACTL7A	0.0126	0	-0.001057275
PTEN	AADAC	0.0127	0	-0.38539947
PTEN	MICB	0.0127	0	0.196182925
PTEN	PGM1	0.0127	0	0.159057285
PTEN	MCCD1	0.0127	0	-0.141827769
PTEN	IFNGR2	0.0128	0	0.018496209

PTEN	CDK19	0.0128	0	-0.187011887
PTEN	PSME1	0.0128	0	0.095828109
PTEN	UTP3	0.0128	0	-0.17933068
PTEN	KRTAP10-9	0.0128	0	-1.14E-05
PTEN	MTMR2	0.0129	0	-0.5171576
PTEN	NME6	0.0129	0	0.000295616
PTEN	MSH4	0.0129	0	-0.196835913
PTEN	MKI67IP	0.013	0	0.001317066
PTEN	SSX8	0.013	0	-0.010973609
PTEN	SERPINA7	0.013	0	-0.024999579
PTEN	EDN3	0.013	0	-0.270212643
PTEN	C22orf43	0.0131	0	-0.000124669
PTEN	IL9R	0.0131	0	-0.000371613
PTEN	SLC9A8	0.0131	0	0.160157539
PTEN	C2orf83	0.0132	0	-0.316771093
PTEN	CCNB3	0.0132	0	-0.226380089
PTEN	KIF26A	0.0132	0	-0.164102759
PTEN	RWDD2B	0.0132	0	-0.016133409
PTEN	NFE2	0.0132	0	-0.00125647
PTEN	ADAMTSL2	0.0132	0	-0.136346051
PTEN	C15orf2	0.0133	0	-0.265584744
PTEN	HINT1	0.0133	0	0.001742603
PTEN	FZD2	0.0133	0	-2.98E-06
PTEN	SCAND3	0.0133	0	-0.377256959
PTEN	FZD7	0.0133	0	-0.210944268
PTEN	FAM54B	0.0133	0	-0.041837396
PTEN	LOC728723	0.0134	0	-0.190241441
PTEN	R3HCC1	0.0134	0	-0.009961115
PTEN	SPACA4	0.0134	0	-0.020761923
PTEN	MED29	0.0134	0	0.025078849
PTEN	HSPA5	0.0134	0	0.398318643
PTEN	ARGLU1	0.0135	0	-0.192273917
PTEN	SNORD116-11	0.0135	0	-0.008102694
PTEN	SRGN	0.0135	0	-0.028942736
PTEN	PNN	0.0135	0	0.207511984
PTEN	DAPL1	0.0135	0	-0.015301516
PTEN	RAD52	0.0135	0	-0.02580879
PTEN	PINK1	0.0136	0	-0.019392336
PTEN	GRB10	0.0136	0	0.128863486
PTEN	SNRNP200	0.0136	0	0.00402599
PTEN	ACP2	0.0136	0	0.032241705

PTEN	NINJ1	0.0136	0	-0.14100739
PTEN	ANKRD34A	0.0136	0	-0.003300575
PTEN	SPN	0.0137	0	-0.005745565
PTEN	NECAP2	0.0137	0	0.017053525
PTEN	LOC284661	0.0138	0	-0.183774286
PTEN	CER1	0.0138	0	-0.174918069
PTEN	FGFR1OP	0.0138	0	-0.145813565
PTEN	ANKRD55	0.0138	0	-0.167281877
PTEN	GRHL1	0.0138	0	-0.096471698
PTEN	PADI4	0.0139	0	-0.137117929
PTEN	PAX7	0.0139	0	-0.005842138
PTEN	RFX6	0.0139	0	-0.019165198
PTEN	S100A13	0.0141	0	1.82E-06
PTEN	CD4	0.0141	0	-0.085495135
PTEN	KIAA1429	0.0141	0	-0.074045354
PTEN	SDS	0.0141	0	-0.048902321
PTEN	NRXN1	0.0141	0	-0.53803835
PTEN	BOC	0.0142	0	-0.160673509
PTEN	PTPRQ	0.0142	0	-0.539903391
PTEN	HERC5	0.0143	0	-0.000130517
PTEN	TES	0.0143	0	-0.055260028
PTEN	FAM91A1	0.0143	0	0.057140378
PTEN	CYP2R1	0.0143	0	-0.003650812
PTEN	LOC100132724	0.0143	0	-0.485097827
PTEN	MLLT4	0.0143	0	-0.279764251
PTEN	IVNS1ABP	0.0144	0	0.016796752
PTEN	SNAR-G1	0.0144	0	-0.001045098
PTEN	BET1	0.0144	0	-0.400833428
PTEN	CRTAM	0.0144	0	-0.448070542
PTEN	METTL2B	0.0145	0	-0.203699912
PTEN	ASMT	0.0145	0	-1.91E-05
PTEN	LZTS2	0.0145	0	-0.019753727
PTEN	SLC9A5	0.0145	0	-0.15075417
PTEN	C17orf28	0.0145	0	0.00073017
PTEN	SLC26A1	0.0145	0	-0.000220783
PTEN	TMEM53	0.0145	0	-0.394286247
PTEN	TP53I3	0.0145	0	-5.76E-05
PTEN	ADAMTS4	0.0145	0	-0.285567602
PTEN	LOC144571	0.0146	0	-0.184405469
PTEN	CDH19	0.0146	0	-0.126636687
PTEN	ZMYM3	0.0146	0	-0.138103576

PTEN	ALDH2	0.0146	0	0.150704152
PTEN	RNF186	0.0147	0	-0.309351044
PTEN	CUEDC2	0.0147	0	7.15E-05
PTEN	DPP10	0.0147	0	-0.433751687
PTEN	DNAJC8	0.0147	0	0.08043703
PTEN	RPH3AL	0.0147	0	-0.007500851
PTEN	SPCS1	0.0149	0	0.074392452
PTEN	TTC22	0.0149	0	-0.00315976
PTEN	RFX5	0.0149	0	-0.023315662
PTEN	IFFO1	0.0149	0	-0.35441082
PTEN	RPH3A	0.0149	0	-0.026702464
PTEN	RCAN1	0.015	0	-0.067079587
PTEN	RPS15A	0.015	0	0.012962612
PTEN	ST6GALNAC4	0.015	0	-4.29E-05
PTEN	KLRF1	0.015	0	-0.064070182
PTEN	ZNF497	0.015	0	-0.000372907
PTEN	RAB1A	0.0151	0	0.077747081
PTEN	SRP68	0.0151	0	0.000272178
PTEN	ABHD1	0.0151	0	-3.07E-06
PTEN	WNT11	0.0151	0	-0.185102257
PTEN	IL5RA	0.0151	0	-0.581150069
PTEN	HOXA1	0.0153	0	-0.326171207
PTEN	ARMS2	0.0153	0	-0.010960401
PTEN	IWS1	0.0153	0	-0.00789531
PTEN	CD163	0.0153	0	-0.016680571
PTEN	SRP14	0.0154	0	0.005815419
PTEN	ARHGEF35	0.0154	0	-0.000412335
PTEN	USHBP1	0.0154	0	0.000312871
PTEN	NDUFA10	0.0154	0	0.001399627
PTEN	UBTF	0.0154	0	-0.005134594
PTEN	C6orf130	0.0154	0	-0.001926611
PTEN	TMEM38B	0.0154	0	-0.384176693
PTEN	CNN2	0.0154	0	0.06532238
PTEN	F11R	0.0155	0	0.004864782
PTEN	RBM33	0.0155	0	-0.010274565
PTEN	LOC442308	0.0155	0	-0.176506272
PTEN	CARD11	0.0155	0	-0.005993867
PTEN	ALG9	0.0155	0	-0.123154866
PTEN	GMPPB	0.0155	0	-4.92E-05
PTEN	PWP1	0.0155	0	-0.062860538
PTEN	MMP23B	0.0156	0	-0.011358617

PTEN	SPANXN5	0.0156	0	-0.00808941
PTEN	NPY5R	0.0156	0	0.050037607
PTEN	CHD3	0.0157	0	0.012543113
PTEN	KIF6	0.0157	0	-0.020975455
PTEN	ZBTB2	0.0157	0	-0.104711654
PTEN	ACRBP	0.0157	0	-0.009344343
PTEN	TNP1	0.0157	0	-0.004992031
PTEN	GUCA1C	0.0158	0	-0.01799005
PTEN	MIA2	0.0158	0	-0.172760564
PTEN	MS4A10	0.0158	0	-0.37951228
PTEN	SPINK1	0.0159	0	-0.000122573
PTEN	FAM135A	0.0159	0	-0.179862567
PTEN	PHLDA1	0.0159	0	-0.113342326
PTEN	FAM99A	0.0159	0	-0.212129935
PTEN	TNFAIP3	0.0159	0	-0.240208337
PTEN	MAP1LC3B	0.0159	0	0.023293743
PTEN	TTTY22	0.016	0	-0.003786349
PTEN	RCN1	0.016	0	0.016989992
PTEN	FOXD2	0.016	0	-0.160448442
PTEN	WDR6	0.0161	0	-0.002192722
PTEN	TTC23	0.0161	0	-0.186899982
PTEN	GM2A	0.0161	0	0.288950634
PTEN	GALNT12	0.0161	0	-0.416916742
PTEN	VTCN1	0.0161	0	-0.011152441
PTEN	NPHS2	0.0162	0	-0.01257989
PTEN	SNORD113-5	0.0162	0	-1.94E-05
PTEN	PAOX	0.0162	0	-0.002290256
PTEN	MEST	0.0163	0	-0.304540767
PTEN	LOC221710	0.0163	0	-0.375733753
PTEN	SLC9A2	0.0163	0	-0.24555356
PTEN	KCNC1	0.0163	0	-0.307494017
PTEN	SAAL1	0.0163	0	-0.006463128
PTEN	WDR1	0.0163	0	0.134384245
PTEN	PRR23B	0.0164	0	-0.006856658
PTEN	BIRC3	0.0164	0	-0.006919768
PTEN	MDP1	0.0164	0	-0.312262141
PTEN	NUP107	0.0164	0	-0.000335002
PTEN	MS4A12	0.0164	0	-0.197985124
PTEN	TMEM44	0.0164	0	-0.238897878
PTEN	DUSP28	0.0166	0	-0.166032539
PTEN	CDR2	0.0166	0	-0.128357256

PTEN	ZNF705D	0.0166	0	-0.567989137
PTEN	RPL4	0.0166	0	1.84E-07
PTEN	BPY2	0.0167	0	-0.797055504
PTEN	KPNA7	0.0167	0	-0.048466575
PTEN	XKR4	0.0167	0	-0.076899535
PTEN	KLK12	0.0167	0	-0.028865822
PTEN	C9	0.0168	0	-0.321044874
PTEN	SERPINE3	0.0168	0	-0.014522733
PTEN	GPC1	0.0168	0	-1.64E-06
PTEN	OXCT2	0.0168	0	-0.01018331
PTEN	FLJ44082	0.0169	0	-0.242941718
PTEN	HNRNPA3	0.017	0	0.319485528
PTEN	FAM65A	0.017	0	-0.162721741
PTEN	ITGB3BP	0.017	0	-0.092271351
PTEN	EEF1B2	0.017	0	0.000160617
PTEN	DRP2	0.017	0	-0.17167209
PTEN	C1orf144	0.0171	0	-0.099300391
PTEN	ZFY	0.0171	0	-0.247064948
PTEN	LPPR2	0.0172	0	9.33E-05
PTEN	CNDP2	0.0172	0	0.011303612
PTEN	CAPN6	0.0172	0	-0.1407729
PTEN	SLC17A2	0.0172	0	-0.144877544
PTEN	ZCCHC11	0.0173	0	-0.146599947
PTEN	HARBI1	0.0173	0	-0.283752852
PTEN	SLA	0.0173	0	-0.147897209
PTEN	PGK2	0.0173	0	-0.001591718
PTEN	CCDC19	0.0173	0	-0.003196603
PTEN	MPDU1	0.0174	0	0.000884686
PTEN	SNORD115-13	0.0174	0	-1.20E-07
PTEN	KRCC1	0.0174	0	-0.121822026
PTEN	ZWILCH	0.0174	0	-0.077023139
PTEN	DERA	0.0175	0	0.09639336
PTEN	ADPGK	0.0175	0	-0.008558708
PTEN	MBD1	0.0175	0	-0.166623566
PTEN	RFX1	0.0175	0	-0.044680248
PTEN	C14orf166B	0.0175	0	-0.007119325
PTEN	BSPRY	0.0175	0	-0.04275735
PTEN	C3orf74	0.0176	0	-0.184106538
PTEN	PLXND1	0.0176	0	-0.011547512
PTEN	ATP2C2	0.0176	0	-0.007030842
PTEN	CMKLR1	0.0176	0	-0.14724011

PTEN	PYGO2	0.0177	0	-0.038729813
PTEN	POM121C	0.0177	0	0.163120956
PTEN	HGSNAT	0.0177	0	-0.010985805
PTEN	SLC22A13	0.0177	0	-0.019514111
PTEN	PVALB	0.0178	0	7.94E-05
PTEN	LOC285194	0.0178	0	-0.747436251
PTEN	SLC9A3R1	0.0179	0	0.019814819
PTEN	GTF2E2	0.0179	0	0.136317067
PTEN	PRKACA	0.0179	0	0.122246137
PTEN	C1orf213	0.0179	0	-0.534591054
PTEN	LRRTM3	0.0179	0	-0.634104016
PTEN	ARHGAP15	0.0179	0	-0.139753317
PTEN	JAKMIP2	0.018	0	-0.137860777
PTEN	NIPAL3	0.018	0	-0.160351253
PTEN	SCUBE3	0.018	0	-0.046782808
PTEN	FAM164C	0.0181	0	-0.000128322
PTEN	KLHL6	0.0181	0	-0.153520718
PTEN	POLR3C	0.0181	0	2.04E-05
PTEN	CSF2RA	0.0181	0	-0.005650708
PTEN	SERTAD3	0.0181	0	-0.001275097
PTEN	ASPDH	0.0182	0	-2.31E-08
PTEN	GPR6	0.0182	0	-0.291180836
PTEN	ADH7	0.0182	0	-0.218610963
PTEN	PLEKHA6	0.0182	0	0.142774553
PTEN	SLC7A7	0.0182	0	-0.095779299
PTEN	NUDT18	0.0182	0	-0.00011986
PTEN	NCRNA00120	0.0182	0	-0.126127405
PTEN	PLD2	0.0182	0	-0.000181498
PTEN	OGFOD1	0.0183	0	-0.023218783
PTEN	NPPA	0.0183	0	-0.18603938
PTEN	ASB13	0.0184	0	-0.0079697
PTEN	RGS11	0.0184	0	0.000472562
PTEN	SLC22A18	0.0184	0	-1.22E-07
PTEN	EPHA7	0.0185	0	-0.459685098
PTEN	ZNF498	0.0186	0	-0.050070459
PTEN	KCNK16	0.0186	0	-0.166896389
PTEN	ZP2	0.0186	0	-1.53E-06
PTEN	POM121L12	0.0186	0	-0.020035733
PTEN	RNPEP	0.0186	0	0.000120622
PTEN	CDK2	0.0186	0	-0.020882131
PTEN	C18orf26	0.0186	0	-0.344387849

PTEN	SNORD1C	0.0186	0	-0.004730245
PTEN	MUC21	0.0187	0	-0.155174364
PTEN	POLB	0.0187	0	-0.02729661
PTEN	HES3	0.0187	0	-2.09E-07
PTEN	VWC2	0.0188	0	-0.148700244
PTEN	HTR4	0.0188	0	-0.902501355
PTEN	EIF4E1B	0.0188	0	-0.001961934
PTEN	PPP1R3D	0.0188	0	-0.552844202
PTEN	FAM122B	0.0188	0	-0.142997719
PTEN	AGPAT3	0.0188	0	-0.100236586
PTEN	AKR1C1	0.019	0	-0.000242456
PTEN	PTPN22	0.019	0	-0.443792092
PTEN	UFC1	0.019	0	0.0092979
PTEN	EMR2	0.0191	0	-0.396171289
PTEN	GABRR2	0.0191	0	-0.001581507
PTEN	NUDT6	0.0191	0	-0.027585115
PTEN	OXCT1	0.0191	0	-0.039990099
PTEN	KRT25	0.0191	0	-0.021772018
PTEN	LRP12	0.0191	0	-0.511449128
PTEN	TAS2R7	0.0192	0	-0.001089449
PTEN	HAS3	0.0192	0	-0.46718367
PTEN	CIR1	0.0192	0	-0.122850462
PTEN	NIT1	0.0193	0	0.010344905
PTEN	HLA-DMB	0.0193	0	0.001072876
PTEN	HAMP	0.0193	0	-6.86E-05
PTEN	MMP23A	0.0194	0	-0.011653889
PTEN	ARL4D	0.0194	0	0.008076458
PTEN	DIO3	0.0194	0	-0.001100402
PTEN	RBP4	0.0194	0	-0.16045419
PTEN	C20orf108	0.0194	0	0.193480482
PTEN	TMSL3	0.0194	0	0.246388976
PTEN	CYGB	0.0194	0	5.45E-05
PTEN	SMC4	0.0194	0	0.009894549
PTEN	CLEC4A	0.0194	0	-0.000934649
PTEN	KRTAP5-7	0.0194	0	-0.010102318
PTEN	MCM3AP	0.0195	0	-0.035774145
PTEN	CRTAC1	0.0195	0	-0.076643003
PTEN	LPA	0.0195	0	-0.229258238
PTEN	POLR1D	0.0195	0	0.128216889
PTEN	C16orf89	0.0196	0	-0.138318999
PTEN	FLJ43859	0.0196	0	-0.368784228

PTEN	LOC100130331	0.0196	0	-0.209243053
PTEN	GLB1	0.0196	0	-0.014761478
PTEN	MYO15A	0.0197	0	-0.180090195
PTEN	GPN2	0.0198	0	-0.003617223
PTEN	POU6F2	0.0199	0	-0.003909992
PTEN	RPL12	0.0199	0	0.000445935
PTEN	ENAM	0.0199	0	-0.44894265
PTEN	MIF4GD	0.02	0	-0.075026914
PTEN	NEIL1	0.02	0	-0.010817817
PTEN	PNMA5	0.02	0	-0.167147411
PTEN	SNAP47	0.02	0	-0.009915372
PTEN	SLC16A1	0.0201	0	-0.382507015
PTEN	TSPAN16	0.0201	0	-3.04E-05
PTEN	CBFB	0.0202	0	0.016575771
PTEN	AGBL3	0.0202	0	-0.174072773
PTEN	TPSD1	0.0202	0	1.30E-05
PTEN	SSX5	0.0203	0	-0.001185089
PTEN	RPGRIP1	0.0203	0	-0.019393372
PTEN	C6orf103	0.0203	0	-0.000894752
PTEN	AKAP3	0.0203	0	-0.000304098
PTEN	GRB2	0.0203	0	0.220222338
PTEN	OR2L2	0.0203	0	-0.000169201
PTEN	SYT2	0.0204	0	-0.144855614
PTEN	DOLPP1	0.0204	0	0.14321166
PTEN	C17orf102	0.0204	0	-0.019124204
PTEN	LDHA	0.0204	0	0.187884814
PTEN	C2orf78	0.0204	0	-7.74E-05
PTEN	FPR3	0.0204	0	-0.08371097
PTEN	SFT2D3	0.0204	0	-0.289337227
PTEN	FCGR2B	0.0204	0	0.110568288
PTEN	CABYR	0.0205	0	-0.164093777
PTEN	VSX1	0.0205	0	-0.24863859
PTEN	PTPRZ1	0.0205	0	-0.387628443
PTEN	C6orf114	0.0205	0	-0.217457646
PTEN	ZC3H12C	0.0205	0	-0.620702042
PTEN	CDAN1	0.0206	0	-0.149717605
PTEN	MSX1	0.0206	0	-0.046694481
PTEN	C9orf72	0.0206	0	-0.048180434
PTEN	C22orf29	0.0206	0	-0.008673244
PTEN	DFNB59	0.0206	0	-0.000296339
PTEN	TMEM182	0.0206	0	-0.285901597

PTEN	TRDMT1	0.0207	0	-0.285831209
PTEN	CHDH	0.0207	0	-0.015529931
PTEN	SEMG2	0.0207	0	-0.153819022
PTEN	PPP1R1A	0.0207	0	-0.050723106
PTEN	LOC649330	0.0208	0	-3.75E-07
PTEN	HPS1	0.0208	0	0.00011806
PTEN	DAOA	0.0208	0	-0.000155924
PTEN	PRMT8	0.0208	0	-0.183554648
PTEN	HNRNPH1	0.0208	0	0.028569508
PTEN	ABHD3	0.0209	0	-0.470924163
PTEN	FLJ35776	0.0209	0	-0.234229189
PTEN	SNORD115-5	0.0209	0	-1.20E-07
PTEN	LRRC37A4	0.0209	0	-0.195863628
PTEN	AZGP1	0.0209	0	9.31E-05
PTEN	VENTXP7	0.0209	0	-0.166775412
PTEN	DMRTA1	0.0209	0	-0.459120359
PTEN	SELPLG	0.0209	0	-0.109999366
PTEN	CLSTN3	0.021	0	-0.052756949
PTEN	ZNF467	0.021	0	0.000668351
PTEN	FNBP1	0.021	0	-0.000413586
PTEN	KNCN	0.021	0	-0.003430649
PTEN	ZFP28	0.021	0	-0.053763031
PTEN	BTBD3	0.021	0	-0.182783241
PTEN	KIF12	0.021	0	-0.000815387
PTEN	SNORD116-8	0.0211	0	-5.35E-05
PTEN	CD68	0.0211	0	0.007096699
PTEN	NR4A1	0.0211	0	-0.026714794
PTEN	CD80	0.0211	0	-0.027364635
PTEN	FGF12	0.0212	0	-0.177176713
PTEN	RNF19A	0.0212	0	-0.071849635
PTEN	MAPK8IP1	0.0212	0	-0.019240902
PTEN	SDC3	0.0212	0	-0.005452042
PTEN	GPRIN2	0.0212	0	0.000444551
PTEN	PLAC1	0.0213	0	-0.010941469
PTEN	NLRC4	0.0213	0	-0.001403544
PTEN	EPSTI1	0.0213	0	-0.007190847
PTEN	ST8SIA5	0.0213	0	-0.017734452
PTEN	MBOAT4	0.0213	0	-0.133552877
PTEN	CFTR	0.0213	0	-0.614653854
PTEN	POLD4	0.0214	0	0.087958225
PTEN	KRTAP10-2	0.0214	0	-1.14E-05

PTEN	FHOD1	0.0214	0	-0.014672289
PTEN	ARAP1	0.0214	0	0.013439527
PTEN	UBA7	0.0214	0	-0.000433811
PTEN	STX8	0.0214	0	3.08E-06
PTEN	SUSD2	0.0215	0	-0.001173514
PTEN	RPL17	0.0215	0	0.001160509
PTEN	C7orf54	0.0215	0	-0.338283576
PTEN	C6orf62	0.0215	0	0.297530667
PTEN	ATG10	0.0216	0	-0.062747105
PTEN	KLK13	0.0216	0	-0.009167361
PTEN	WBSCR17	0.0216	0	-0.148996149
PTEN	LOC93432	0.0216	0	-3.64E-06
PTEN	SNORD114-9	0.0216	0	-9.63E-05
PTEN	RAP1GAP	0.0216	0	0.002539619
PTEN	SCGB1D4	0.0216	0	-0.004827173
PTEN	HOOK2	0.0217	0	-0.115066698
PTEN	CPOX	0.0217	0	0.116018311
PTEN	P4HA2	0.0217	0	0.162313946
PTEN	MAEL	0.0217	0	-0.026318173
PTEN	TNFSF12	0.0218	0	-0.029824491
PTEN	WFIKKN2	0.0218	0	-0.292893417
PTEN	PSME3	0.0218	0	0.178516106
PTEN	ZNF518B	0.0218	0	-0.273750712
PTEN	UTY	0.0218	0	-0.424417149
PTEN	GTF2I	0.0218	0	0.001737876
PTEN	MCART1	0.0219	0	-4.10E-05
PTEN	IL17D	0.0219	0	-0.00545752
PTEN	SFTPA1	0.0219	0	-0.164458025
PTEN	LDHAL6B	0.0219	0	-0.003826926
PTEN	INSL6	0.022	0	-0.015848397
PTEN	CST9L	0.022	0	-0.163610985
PTEN	HMGN2	0.022	0	0.160324177
PTEN	ST3GAL6	0.022	0	-0.288564937
PTEN	SNORA27	0.022	0	-0.014145051
PTEN	ARHGAP10	0.0221	0	-0.007211863
PTEN	GCC1	0.0221	0	-0.142403479
PTEN	SMARCC1	0.0221	0	0.119596743
PTEN	IKZF1	0.0221	0	-0.247512057
PTEN	REG3G	0.0221	0	-0.013004671
PTEN	GRSF1	0.0221	0	-0.187518525
PTEN	FOXN2	0.0222	0	-0.327215468

PTEN	STAU2	0.0222	0	-0.075164039
PTEN	ANKRD49	0.0222	0	-0.027665453
PTEN	SLC27A3	0.0223	0	-0.000264419
PTEN	NUDCD3	0.0223	0	-0.001053709
PTEN	GABRQ	0.0223	0	-1.53E-05
PTEN	SLC22A9	0.0223	0	-0.00999647
PTEN	SLC5A1	0.0224	0	-0.189381036
PTEN	NACAP1	0.0224	0	-0.013541063
PTEN	CYP2E1	0.0225	0	-0.03022076
PTEN	PATL1	0.0226	0	0.252012338
PTEN	UBE2DNL	0.0226	0	-1.14E-05
PTEN	IRF2BP2	0.0226	0	0.243734575
PTEN	UGT3A2	0.0227	0	-0.00995054
PTEN	RSPH9	0.0227	0	-0.007055494
PTEN	BTF3L1	0.0227	0	-0.305671314
PTEN	SNORD114-2	0.0227	0	-0.001883928
PTEN	TMEM117	0.0227	0	-0.166043986
PTEN	LOC440563	0.0227	0	-3.75E-07
PTEN	MYO3B	0.0227	0	-0.172516544
PTEN	PIK3R5	0.0227	0	-0.169113652
PTEN	UBAP2L	0.0228	0	0.007131639
PTEN	CACNG2	0.0228	0	-0.017314356
PTEN	GDF9	0.0228	0	-0.120456543
PTEN	ANO10	0.0228	0	-0.000537624
PTEN	NLRP10	0.0229	0	-2.35E-07
PTEN	HOXA2	0.0229	0	-0.038811589
PTEN	URB2	0.0229	0	-0.298142271
PTEN	PLEKHA8	0.0229	0	-0.004065489
PTEN	PLEKHG7	0.0229	0	-0.06959375
PTEN	CTSF	0.0229	0	-0.026046934
PTEN	RPL5	0.023	0	9.97E-05
PTEN	DENND1A	0.023	0	-0.012312454
PTEN	CLDND1	0.023	0	-0.07601532
PTEN	CT47A6	0.023	0	-0.015991177
PTEN	CYP2C9	0.023	0	-0.105490363
PTEN	GPR180	0.023	0	-0.211346905
PTEN	LOC150527	0.0231	0	-0.243231515
PTEN	AKTIP	0.0231	0	-0.163127647
PTEN	PKDREJ	0.0232	0	-0.040667079
PTEN	TRAF3IP2	0.0232	0	0.153844147
PTEN	ECHS1	0.0233	0	-0.000256389

PTEN	FAM160B2	0.0233	0	-0.000732364
PTEN	CLCNKB	0.0233	0	-0.108119244
PTEN	ESCO2	0.0233	0	-0.11551087
PTEN	IFI44	0.0233	0	0.017782606
PTEN	REG4	0.0233	0	-0.028150131
PTEN	FAM55D	0.0233	0	-0.010964855
PTEN	CLEC17A	0.0233	0	-0.243759125
PTEN	TMEM56	0.0233	0	-0.100445376
PTEN	ATP6V0A1	0.0234	0	-0.129470798
PTEN	SLC24A5	0.0234	0	-9.70E-05
PTEN	CPNE4	0.0234	0	-0.396027075
PTEN	MT1DP	0.0234	0	-0.00091288
PTEN	SPRYD5	0.0234	0	-0.007893873
PTEN	ZNF470	0.0234	0	-0.611108444
PTEN	MYL2	0.0234	0	-4.48E-05
PTEN	NEUROD6	0.0235	0	-0.576280708
PTEN	IGFL1	0.0235	0	-0.008935476
PTEN	UCN2	0.0235	0	-0.096468036
PTEN	LOC158572	0.0235	0	-0.723967196
PTEN	HIF1A	0.0235	0	-0.014535248
PTEN	SHISA3	0.0235	0	-0.077238072
PTEN	LOC285401	0.0235	0	-0.162281355
PTEN	PLXNB2	0.0236	0	-0.000487928
PTEN	C5orf48	0.0236	0	-3.97E-06
PTEN	ALDH1L1	0.0236	0	-0.000150197
PTEN	STK24	0.0237	0	-0.09839635
PTEN	CD300A	0.0237	0	-0.007388347
PTEN	PLEKHG2	0.0237	0	-0.073135365
PTEN	FAM19A5	0.0237	0	-0.052453727
PTEN	PADI6	0.0237	0	-0.097013419
PTEN	ID3	0.0237	0	-0.017930043
PTEN	CUX2	0.0237	0	-0.305251585
PTEN	NRXN2	0.0238	0	-0.000977541
PTEN	NMT1	0.0239	0	-0.00363584
PTEN	VSIG8	0.0239	0	-0.008148559
PTEN	DAO	0.0239	0	-0.176317114
PTEN	SNORA15	0.0239	0	-0.001833089
PTEN	FOXJ1	0.0239	0	-0.030163615
PTEN	BANK1	0.0239	0	-0.391358311
PTEN	ICA1L	0.0239	0	-0.42770954
PTEN	N6AMT2	0.0239	0	-0.000346732

PTEN	UGT3A1	0.0239	0	-0.012855627
PTEN	CTAGE6	0.024	0	-0.021062172
PTEN	CLIP4	0.024	0	-0.044626853
PTEN	TIFA	0.0241	0	0.065075506
PTEN	FAM40A	0.0241	0	0.020736026
PTEN	HSPA8	0.0241	0	0.031015971
PTEN	GSTO1	0.0241	0	0.000110455
PTEN	CKAP5	0.0241	0	-0.005060337
PTEN	UTP18	0.0241	0	-0.125492211
PTEN	C17orf108	0.0241	0	-0.021573383
PTEN	HIST1H2AJ	0.0242	0	0.000215703
PTEN	C1orf92	0.0242	0	-0.01933131
PTEN	WDHD1	0.0243	0	-0.011312188
PTEN	FASTKD1	0.0243	0	-0.003762387
PTEN	LOC29034	0.0243	0	-0.348228344
PTEN	DCC	0.0243	0	-0.082925395
PTEN	MT1M	0.0243	0	-0.239604605
PTEN	OGFRL1	0.0243	0	-0.022401542
PTEN	KRT8	0.0243	0	7.13E-06
PTEN	LOC285205	0.0243	0	-0.003754463
PTEN	CCDC53	0.0243	0	-0.002414784
PTEN	FAM9C	0.0243	0	-0.156372312
PTEN	GOLGA3	0.0245	0	0.004915459
PTEN	CTXN3	0.0245	0	-0.254268621
PTEN	C6orf168	0.0246	0	-0.143347039
PTEN	C9orf109	0.0246	0	-0.008235367
PTEN	FGF4	0.0246	0	-0.043397033
PTEN	TM2D3	0.0247	0	-0.144388092
PTEN	CNIH4	0.0247	0	0.033552502
PTEN	TTC27	0.0247	0	-0.018390634
PTEN	DLEU1	0.0247	0	-0.02988804
PTEN	MPP2	0.0247	0	-0.17559987
PTEN	LGMN	0.0247	0	-0.001293535
PTEN	RIMBP3	0.0247	0	-0.011846293
PTEN	HCG9	0.0248	0	-0.000519037
PTEN	PCSK9	0.0248	0	-0.005807805
PTEN	NANOS1	0.0248	0	-0.209392735
PTEN	ADH4	0.0248	0	-0.158348523
PTEN	IL15	0.0248	0	-0.20882318
PTEN	MAGEB16	0.0248	0	-0.138426691
PTEN	C1orf125	0.0248	0	-0.041244181

PTEN	TTLL7	0.0249	0	-0.263302398
PTEN	ZBTB3	0.0249	0	-0.32727288
PTEN	KRTAP4-9	0.0249	0	-0.012398356
PTEN	AFM	0.0249	0	-0.180176547
PTEN	UBE2D4	0.0249	0	-0.135916833
PTEN	NOL11	0.0249	0	-0.000959385
PTEN	ID4	0.0249	0	-0.113042755
PTEN	P2RY2	0.025	0	-0.005445957
PTEN	PEG3	0.025	0	-0.024118809
PTEN	FAM71F1	0.025	0	-0.029921856
PTEN	VGLL2	0.025	0	-0.332930658
PTEN	PLUNC	0.025	0	-0.000803092
PTEN	ELANE	0.025	0	-5.40E-05
PTEN	SELV	0.0251	0	-0.025459611
PTEN	FUNDC1	0.0251	0	-0.161112825
PTEN	GPR109A	0.0251	0	-0.018591826
PTEN	SIGLEC9	0.0251	0	-0.002688724
PTEN	DKFZp434L192	0.0252	0	-0.024811329
PTEN	UBE2A	0.0252	0	-0.254715907
PTEN	FCGR3B	0.0252	0	-0.029650656
PTEN	RNF214	0.0252	0	-0.049651939
PTEN	HES1	0.0252	0	-0.119945662
PTEN	BMP3	0.0252	0	-0.481436027
PTEN	MOBKL2A	0.0252	0	-0.094944145
PTEN	CHST14	0.0253	0	0.002146152
PTEN	GPR17	0.0253	0	-0.007135125
PTEN	HRASLS2	0.0253	0	0.080152043
PTEN	PSG1	0.0253	0	-0.3198277
PTEN	GNMT	0.0253	0	1.18E-09
PTEN	ACSL1	0.0254	0	-0.098335848
PTEN	KIF9	0.0254	0	-0.091230436
PTEN	KLHL31	0.0254	0	-0.291725924
PTEN	IQCC	0.0255	0	-0.005303434
PTEN	PEG10	0.0255	0	0.146510582
PTEN	KCTD8	0.0255	0	-0.26179754
PTEN	TBC1D4	0.0255	0	-0.328671446
PTEN	ADRB3	0.0256	0	-0.003918362
PTEN	LRRC52	0.0256	0	-0.000353842
PTEN	WNT5B	0.0256	0	-0.050229451
PTEN	FBXL16	0.0256	0	-0.162573229
PTEN	BRI3BP	0.0257	0	-0.157001521

PTEN	C3orf36	0.0257	0	-0.000352517
PTEN	CAMK1D	0.0257	0	-0.142333619
PTEN	MKS1	0.0257	0	-0.000364467
PTEN	MLLT6	0.0257	0	0.079715221
PTEN	DKFZP686I15217	0.0257	0	0.023483127
PTEN	ANGPTL3	0.0258	0	-0.523773924
PTEN	CCNI2	0.0258	0	-0.189394279
PTEN	DUSP11	0.0258	0	-0.130021463
PTEN	METTL6	0.0258	0	-0.103298251
PTEN	DHFR	0.0259	0	-0.070492306
PTEN	LMOD3	0.0259	0	-0.063330491
PTEN	TMTC4	0.0259	0	-0.032781512
PTEN	SNAP91	0.026	0	-0.621497872
PTEN	FAM5C	0.026	0	-0.118417933
PTEN	COMMD6	0.0261	0	0.015679054
PTEN	CCNA1	0.0261	0	-0.112544998
PTEN	CD86	0.0261	0	-0.009462105
PTEN	C20orf12	0.0261	0	-0.211935897
PTEN	ZNF677	0.0262	0	-0.351187536
PTEN	PPM1K	0.0262	0	-0.132983369
PTEN	KIAA1731	0.0262	0	6.90E-05
PTEN	WFDC2	0.0262	0	-0.115464427
PTEN	CYLD	0.0263	0	0.149882533
PTEN	SNORD116-29	0.0263	0	-0.128937994
PTEN	ST18	0.0263	0	-0.517269351
PTEN	PPP1R16B	0.0263	0	-0.234243962
PTEN	FIP1L1	0.0265	0	0.060814825
PTEN	NDST4	0.0265	0	-4.97E-07
PTEN	FAM161A	0.0265	0	-0.188024859
PTEN	DEFB119	0.0265	0	-0.000760437
PTEN	DKFZp434J0226	0.0265	0	-0.162523955
PTEN	PAAF1	0.0265	0	0.162548482
PTEN	TMEM99	0.0265	0	0.032507055
PTEN	PYROXD2	0.0265	0	-0.00054651
PTEN	ANAPC10	0.0266	0	0.029829044
PTEN	MOCS2	0.0266	0	0.143340511
PTEN	ZNF193	0.0266	0	-0.110067075
PTEN	C14orf176	0.0266	0	-0.014114452
PTEN	DHDPSL	0.0267	0	-0.018405929
PTEN	LOC550112	0.0267	0	-0.02642032
PTEN	SNORD115-48	0.0267	0	-6.48E-05

PTEN	FSHR	0.0267	0	-0.081793733
PTEN	C12orf69	0.0267	0	-0.474243297
PTEN	NBPF16	0.0267	0	-0.097069676
PTEN	PPIL6	0.0268	0	-0.284040647
PTEN	UBE2W	0.0268	0	-0.224846348
PTEN	EFHD1	0.0268	0	0.090219847
PTEN	PSG8	0.0269	0	-0.00044891
PTEN	EARS2	0.0269	0	-0.033718627
PTEN	IL11RA	0.0269	0	-0.146019613
PTEN	PSD4	0.0269	0	-0.058813561
PTEN	GRAMD2	0.0269	0	-0.026756507
PTEN	CNNM3	0.027	0	-0.035473547
PTEN	NXNL1	0.027	0	-4.05E-05
PTEN	WDR87	0.0271	0	-4.51E-07
PTEN	HPN	0.0271	0	-0.000300387
PTEN	ANKRD36	0.0271	0	0.000698461
PTEN	CDKN1C	0.0271	0	-0.309826528
PTEN	TSPO2	0.0271	0	-5.28E-05
PTEN	ZC3H4	0.0271	0	-0.166120252
PTEN	TNKS1BP1	0.0271	0	0.005470833
PTEN	IMPAD1	0.0272	0	0.002350222
PTEN	ETV6	0.0272	0	0.001970063
PTEN	FAM176A	0.0272	0	-0.184368131
PTEN	TDRD10	0.0272	0	-0.184644458
PTEN	RNF121	0.0272	0	-0.002200116
PTEN	MYBPC1	0.0272	0	-0.005889337
PTEN	LOC728989	0.0272	0	-0.000364842
PTEN	ZNF703	0.0272	0	-0.000129063
PTEN	NSMCE1	0.0273	0	0.000132759
PTEN	C10orf131	0.0273	0	-0.176955251
PTEN	C11orf88	0.0274	0	-0.003299653
PTEN	RPL23A	0.0274	0	0.124282855
PTEN	CELA2A	0.0275	0	-0.00041797
PTEN	LOC643923	0.0275	0	-0.363562822
PTEN	KLHL26	0.0275	0	-0.008865014
PTEN	EIF1	0.0275	0	0.334111683
PTEN	FAM46D	0.0275	0	-0.299878346
PTEN	SLC9A4	0.0275	0	-0.222445089
PTEN	BTK	0.0275	0	-0.009675683
PTEN	AQP4	0.0276	0	-0.411883171
PTEN	SNORA63	0.0276	0	-0.000179338

PTEN	HP	0.0277	0	-0.003489362
PTEN	AGBL1	0.0277	0	-0.029613667
PTEN	LOC255167	0.0277	0	-0.015896904
PTEN	SYNCRIP	0.0278	0	0.278572552
PTEN	FOXC2	0.0279	0	6.43E-10
PTEN	GBA3	0.0279	0	-0.302582975
PTEN	C1GALT1C1	0.0279	0	-0.011867403
PTEN	HIST1H2AC	0.0279	0	0.000332208
PTEN	SPAG8	0.0279	0	-0.006276563
PTEN	TESK2	0.0279	0	-0.149629293
PTEN	ITGA7	0.0279	0	0.003596172
PTEN	INTS4L1	0.028	0	-0.390676839
PTEN	TOPBP1	0.028	0	0.00622265
PTEN	KISS1R	0.028	0	-0.011299393
PTEN	NMNAT3	0.028	0	-0.004146924
PTEN	SIPA1L3	0.028	0	-0.012176548
PTEN	ZNF280C	0.028	0	-0.359865392
PTEN	C17orf77	0.028	0	-0.009073118
PTEN	SLC22A11	0.028	0	-0.152575188
PTEN	SORBS3	0.0281	0	-0.000498803
PTEN	NCRNA00230B	0.0281	0	-0.000533665
PTEN	MFSD9	0.0281	0	-0.352322509
PTEN	CD1D	0.0281	0	-0.202396618
PTEN	MUC20	0.0281	0	-0.26953665
PTEN	SULT1A2	0.0281	0	-1.13E-05
PTEN	CLECL1	0.0281	0	-0.00560498
PTEN	LCP2	0.0282	0	-0.196230552
PTEN	RNF10	0.0282	0	-0.000572549
PTEN	ZNF833	0.0282	0	-0.270702302
PTEN	CLTCL1	0.0282	0	-0.002899002
PTEN	C19orf30	0.0282	0	-0.007290814
PTEN	LHFPL4	0.0282	0	-0.235255679
PTEN	GHDC	0.0282	0	-0.073199726
PTEN	GPR174	0.0282	0	-0.145298179
PTEN	HOXA3	0.0282	0	-0.692369306
PTEN	TMEM125	0.0283	0	-0.000959445
PTEN	CHRNA2	0.0282	0	-0.153473198
PTEN	CHST10	0.0283	0	-0.00948657
PTEN	SLC25A26	0.0283	0	-0.025497092
PTEN	HIST4H4	0.0283	0	-6.16E-08
PTEN	NLRP2	0.0283	0	-0.06357725

PTEN	ASXL1	0.0283	0	-0.00735075
PTEN	NTNG2	0.0283	0	-0.000751608
PTEN	LRRC8E	0.0283	0	-0.094784228
PTEN	PROSC	0.0284	0	-0.089928994
PTEN	PKHD1L1	0.0284	0	-0.531808361
PTEN	C15orf40	0.0284	0	-0.384073489
PTEN	HSPA1L	0.0285	0	-0.002587177
PTEN	ITGB8	0.0285	0	-0.378893804
PTEN	LASS4	0.0285	0	4.96E-05
PTEN	CACNA1H	0.0285	0	-0.000213822
PTEN	GFAP	0.0285	0	-0.03084726
PTEN	CHGA	0.0285	0	-0.149744101
PTEN	SYN2	0.0285	0	-0.555867664
PTEN	FTH1	0.0286	0	0.028456597
PTEN	UNC45B	0.0286	0	-0.587578806
PTEN	LYPLA1	0.0286	0	-0.069122095
PTEN	DYDC1	0.0287	0	-2.42E-05
PTEN	GLOD5	0.0287	0	-6.61E-05
PTEN	SDAD1	0.0287	0	0.034544756
PTEN	NPTX1	0.0287	0	-0.45280429
PTEN	SNORA13	0.0288	0	-0.007538698
PTEN	FAM134C	0.0288	0	-0.003778223
PTEN	RRAD	0.0288	0	0.104830739
PTEN	PTGFR	0.0288	0	-0.327020849
PTEN	CDC42EP2	0.0288	0	-0.003166981
PTEN	PTPRH	0.0289	0	-0.004638733
PTEN	LOC441869	0.0289	0	-0.002021232
PTEN	FSHB	0.0289	0	-0.25206161
PTEN	TTTY7	0.0289	0	-0.204532908
PTEN	C3orf62	0.029	0	-0.127747358
PTEN	NBR2	0.029	0	-0.032912644
PTEN	CWH43	0.029	0	-0.008789313
PTEN	MSX2P1	0.029	0	-0.023462596
PTEN	DARS2	0.029	0	-0.119592017
PTEN	FLJ42393	0.029	0	-0.324298043
PTEN	KRTAP24-1	0.0291	0	-0.175409846
PTEN	TACR2	0.0291	0	-0.040082461
PTEN	OR2A9P	0.0292	0	-0.000426691
PTEN	C21orf29	0.0292	0	-0.020089672
PTEN	ZNF514	0.0292	0	-0.050937504
PTEN	CAMK2B	0.0292	0	-0.210471205

PTEN	C20orf3	0.0292	0	-0.122568009
PTEN	PPL	0.0292	0	-0.025197767
PTEN	MTSS1	0.0292	0	-0.133578173
PTEN	LOC285593	0.0292	0	-0.245522558
PTEN	NRD1	0.0293	0	-0.006921806
PTEN	psiTPTE22	0.0293	0	-0.285186897
PTEN	ZNF257	0.0293	0	-0.349941065
PTEN	C12orf57	0.0293	0	1.17E-09
PTEN	HIST3H2BB	0.0293	0	8.43E-05
PTEN	FBXO32	0.0294	0	-0.042146989
PTEN	EPB41L4B	0.0294	0	-0.526066011
PTEN	C6orf182	0.0294	0	-0.056308161
PTEN	CBWD3	0.0294	0	0.105040949
PTEN	MET	0.0295	0	-0.478174987
PTEN	C5AR1	0.0295	0	-0.007742371
PTEN	SEC22A	0.0295	0	-0.271365801
PTEN	GRIN2B	0.0295	0	-0.255006319
PTEN	DOLK	0.0296	0	-0.005847397
PTEN	CXorf30	0.0296	0	-0.15978091
PTEN	C5orf32	0.0296	0	-0.002775772
PTEN	LHFPL3	0.0296	0	-0.403965809
PTEN	SMAD6	0.0297	0	-0.265877165
PTEN	C12orf75	0.0297	0	-0.202486914
PTEN	C8B	0.0297	0	-0.002188996
PTEN	KCTD1	0.0297	0	-0.183985866
PTEN	C12orf12	0.0298	0	-0.247454851
PTEN	NDNL2	0.0298	0	-0.128321595
PTEN	C16orf73	0.0298	0	0.040674623
PTEN	ITPRIPL1	0.0298	0	-2.04E-05
PTEN	PDLIM4	0.0299	0	-0.134450631
PTEN	SNORD93	0.0299	0	-0.000577866
PTEN	TBX10	0.0299	0	-0.001461326
PTEN	LRIT3	0.0299	0	-0.241602774
PTEN	ANKRD22	0.0299	0	-0.197396764
PTEN	SP6	0.03	0	-0.054836057
PTEN	ACTR6	0.03	0	-0.027901034
PTEN	RPE65	0.03	0	-0.236751242
PTEN	VCPIP1	0.03	0	-0.401585692
PTEN	BMS1	0.03	0	-0.082857261
PTEN	SERBP1	0.03	0	0.062943856
PTEN	ZDHHC7	0.03	0	-0.489034152

PTEN	IL17REL	0.03	0	-0.000463363
PTEN	RICH2	0.0301	0	-0.291809562
PTEN	LMOD2	0.0302	0	-0.183082861
PTEN	ST8SIA3	0.0302	0	-3.57E-06
PTEN	GRASP	0.0302	0	-0.023540344
PTEN	FBXO45	0.0302	0	-3.31E-05
PTEN	ABCA5	0.0303	0	-0.188618136
PTEN	PARK2	0.0304	0	-0.102771101
PTEN	HUS1	0.0304	0	-0.282017902
PTEN	FAM69C	0.0304	0	-0.01557345
PTEN	HHIPL1	0.0304	0	0.106664255
PTEN	ZNF2	0.0306	0	-0.081249081
PTEN	IQSEC2	0.0306	0	-0.002600787
PTEN	PDE8A	0.0306	0	-0.32989757
PTEN	LOC285419	0.0306	0	0.032810288
PTEN	HIST2H4A	0.0307	0	0.000776431
PTEN	GPR173	0.0308	0	-0.001571875
PTEN	HSCB	0.0309	0	0.003489673
PTEN	SNORD114-6	0.0309	0	-2.51E-06
PTEN	PDK2	0.0309	0	-0.000317986
PTEN	NFYA	0.031	0	-0.166857131
PTEN	HOXD3	0.031	0	-0.029569
PTEN	OS9	0.0311	0	9.71E-05
PTEN	PI15	0.0311	0	-0.128445923
PTEN	SPA17	0.0311	0	-0.116392063
PTEN	TRIB2	0.0312	0	-0.239952608
PTEN	HNRNPA3P1	0.0312	0	0.037755192
PTEN	NNAT	0.0313	0	-0.012937971
PTEN	VNN2	0.0313	0	-0.041818672
PTEN	CCNJ	0.0313	0	-0.332897234
PTEN	SNORA38B	0.0313	0	-0.001470302
PTEN	HIST1H2BD	0.0314	0	-0.134085849
PTEN	FAM71F2	0.0315	0	-0.000386624
PTEN	LOC100169752	0.0315	0	-0.011321358
PTEN	OPN4	0.0315	0	-0.254088149
PTEN	TMEM200B	0.0316	0	-0.011584021
PTEN	ZDHHC11	0.0316	0	-0.023674199
PTEN	TERF2	0.0316	0	-0.14067202
PTEN	LRRFIP2	0.0316	0	-0.018894639
PTEN	AOAH	0.0316	0	-0.043687917
PTEN	CCDC67	0.0316	0	-0.573918214

PTEN	TDP1	0.0317	0	-0.12256831
PTEN	CCDC140	0.0317	0	-0.314574051
PTEN	PACSIN2	0.0317	0	-0.12282719
PTEN	BMF	0.0317	0	-0.114079761
PTEN	PTCHD2	0.0318	0	-0.225348005
PTEN	TFIP11	0.0318	0	-0.017986839
PTEN	SIRPB2	0.0318	0	-0.003878984
PTEN	C10orf55	0.0319	0	-0.18234415
PTEN	PLG	0.0319	0	-0.000852231
PTEN	ELOVL3	0.032	0	-0.009760288
PTEN	C20orf70	0.032	0	-0.001615891
PTEN	APEX1	0.032	0	-0.145849582
PTEN	TRIM25	0.032	0	-0.025134988
PTEN	ILDR1	0.0321	0	-0.119698464
PTEN	RASD1	0.0321	0	0.141967033
PTEN	JARID2	0.0321	0	-0.274204759
PTEN	ABCC6	0.0322	0	-0.22570982
PTEN	IFT140	0.0322	0	-0.128389747
PTEN	GCM1	0.0322	0	-0.285017882
PTEN	CPVL	0.0322	0	-0.141467123
PTEN	GEFT	0.0322	0	-0.010727279
PTEN	GIP	0.0322	0	-0.004221976
PTEN	ZNF248	0.0323	0	-0.287573756
PTEN	PLK3	0.0323	0	-0.009324251
PTEN	SNORD114-10	0.0323	0	-9.74E-05
PTEN	IL1A	0.0323	0	-0.091158356
PTEN	SYN3	0.0324	0	-0.066515404
PTEN	IRF8	0.0324	0	-0.082350388
PTEN	RGS3	0.0324	0	0.006244968
PTEN	DHRS2	0.0324	0	0.009461919
PTEN	RAB3C	0.0324	0	-0.017979848
PTEN	ELMO2	0.0325	0	-0.046292173
PTEN	CBLN3	0.0325	0	-0.02060955
PTEN	USP13	0.0325	0	-0.118213973
PTEN	STK31	0.0325	0	-0.000898012
PTEN	TTL	0.0326	0	-0.096769735
PTEN	PANK1	0.0326	0	-0.379927441
PTEN	CALHM1	0.0327	0	-0.039151161
PTEN	HSFY2	0.0327	0	-0.40387929
PTEN	DEFB109P1B	0.0327	0	-0.001582014
PTEN	SCFD2	0.0327	0	-0.01421708

PTEN	TNPO3	0.0328	0	-0.264964841
PTEN	KCTD19	0.0328	0	-2.98E-07
PTEN	PRRG1	0.0328	0	-0.116692133
PTEN	SLC24A6	0.0328	0	-0.000521967
PTEN	HLCS	0.0329	0	-0.109113637
PTEN	FAM40B	0.0329	0	-0.415059894
PTEN	RAD51AP2	0.0329	0	-0.007215398
PTEN	HS3ST5	0.0329	0	-0.273737702
PTEN	CDKAL1	0.033	0	0.108986369
PTEN	SETDB1	0.033	0	-0.022067613
PTEN	BRIP1	0.033	0	-0.282609338
PTEN	SOX30	0.033	0	-0.559311306
PTEN	TTPAL	0.0331	0	-0.150846719
PTEN	TXK	0.0331	0	-0.052546125
PTEN	RPS14	0.0331	0	1.98E-05
PTEN	LUC7L3	0.0332	0	0.123738172
PTEN	MAGED1	0.0332	0	0.095044547
PTEN	PLEK2	0.0332	0	-0.162870397
PTEN	SNORD73A	0.0332	0	-0.001691121
PTEN	JUN	0.0332	0	0.151182527
PTEN	CACNA1A	0.0332	0	-0.138563247
PTEN	SPIN4	0.0333	0	-0.085574038
PTEN	IL18R1	0.0333	0	-0.20650413
PTEN	ATP8A1	0.0333	0	-0.542921035
PTEN	NPY2R	0.0333	0	-0.614997284
PTEN	CYorf15A	0.0333	0	-0.129360825
PTEN	SLITRK5	0.0334	0	-0.346801377
PTEN	WDR76	0.0334	0	-0.341217306
PTEN	CCT6B	0.0334	0	-0.027086159
PTEN	SNTB1	0.0335	0	-0.179324933
PTEN	GYPA	0.0335	0	-0.350106206
PTEN	DCTD	0.0335	0	0.031107825
PTEN	FDFT1	0.0335	0	-0.145127628
PTEN	ROS1	0.0336	0	-0.173772946
PTEN	FAM26D	0.0336	0	-0.31462387
PTEN	ABCD2	0.0336	0	-0.36602519
PTEN	MGC2752	0.0336	0	-0.066938545
PTEN	GPA33	0.0336	0	-0.003568366
PTEN	MST1R	0.0337	0	-0.000130468
PTEN	ALDH1A3	0.0337	0	-0.259143721
PTEN	PGA5	0.0337	0	-0.003799579

PTEN	LOC645332	0.0337	0	-0.177828412
PTEN	DYNLL2	0.0337	0	0.051279362
PTEN	GTPBP8	0.0338	0	-0.149238577
PTEN	CCDC90B	0.0338	0	-0.155674418
PTEN	CD200R1L	0.0338	0	-0.001499471
PTEN	ZNF710	0.0339	0	-0.000842647
PTEN	STAT1	0.0339	0	0.200988875
PTEN	GOSR2	0.0339	0	0.027042018
PTEN	HS6ST2	0.0339	0	-0.33945493
PTEN	VENTX	0.034	0	-0.089397147
PTEN	C12orf77	0.0341	0	-0.014102249
PTEN	VSTM2A	0.0341	0	-0.055154064
PTEN	SNORD24	0.0341	0	-0.000742234
PTEN	LIX1	0.0341	0	-0.900834546
PTEN	ZNF124	0.0342	0	-0.196799244
PTEN	C1orf146	0.0342	0	-0.187152032
PTEN	PLAGL2	0.0342	0	0.028371458
PTEN	KIAA0922	0.0342	0	-0.013229444
PTEN	C10orf67	0.0342	0	-0.351256252
PTEN	RPL7A	0.0342	0	0.005618579
PTEN	SAG	0.0342	0	-0.000271362
PTEN	SNORA4	0.0343	0	-3.44E-06
PTEN	LGALS14	0.0344	0	-0.000309283
PTEN	ATPBD4	0.0345	0	-0.700665704
PTEN	HIST1H3E	0.0345	0	-6.71E-06
PTEN	TAAR5	0.0345	0	-8.96E-07
PTEN	HOXB7	0.0345	0	-0.147510481
PTEN	SLC24A4	0.0345	0	-0.367144284
PTEN	PRG1	0.0346	0	-0.025230045
PTEN	LILRA4	0.0346	0	-0.01560944
PTEN	IFIT1B	0.0346	0	-0.491857075
PTEN	GFRA2	0.0346	0	-0.030229482
PTEN	GPR112	0.0347	0	-0.028809147
PTEN	DOK5	0.0347	0	-0.274704997
PTEN	ARAP3	0.0348	0	-0.381365085
PTEN	LOC162632	0.0348	0	-0.342780867
PTEN	MDH1B	0.0349	0	-0.189362683
PTEN	HKR1	0.0349	0	-0.00502815
PTEN	OLFM4	0.0349	0	-0.002045847
PTEN	FLII	0.035	0	-0.126937253
PTEN	RPS6	0.035	0	8.99E-06

PTEN	SRRM3	0.035	0	-0.153560382
PTEN	DNAH10	0.0351	0	-0.027882088
PTEN	TAF5	0.0351	0	-0.024582703
PTEN	ARHGEF37	0.0351	0	-0.037182913
PTEN	CNTNAP5	0.0351	0	-0.159631833
PTEN	MAGEE1	0.0352	0	-0.111956806
PTEN	MRPL42	0.0352	0	-0.040876371
PTEN	AJAP1	0.0353	0	-0.356753395
PTEN	YIPF7	0.0353	0	-9.34E-05
PTEN	COQ10A	0.0353	0	-0.142145526
PTEN	KCNK10	0.0353	0	-0.407171905
PTEN	TCEB3	0.0353	0	-0.130094179
PTEN	SLC35A4	0.0353	0	-0.028493919
PTEN	HOXD13	0.0353	0	-0.349385372
PTEN	HINT3	0.0354	0	-0.014771832
PTEN	KRT27	0.0354	0	-0.003943229
PTEN	HPCAL4	0.0354	0	-0.300241473
PTEN	ITPKB	0.0354	0	-0.133878158
PTEN	MGC34034	0.0355	0	-0.235481254
PTEN	CHMP5	0.0355	0	-0.01673313
PTEN	DNAJC19	0.0355	0	0.03967683
PTEN	SERTAD4	0.0356	0	-0.22109274
PTEN	NAGA	0.0356	0	-0.046005489
PTEN	RASGRP3	0.0356	0	-0.088957636
PTEN	HNF4A	0.0357	0	-0.020450665
PTEN	CDNF	0.0357	0	0.006827452
PTEN	MUC6	0.0357	0	-9.44E-06
PTEN	C12orf36	0.0357	0	-0.168920929
PTEN	C3orf70	0.0357	0	-0.676805241
PTEN	GTF2B	0.0357	0	0.012212462
PTEN	C2orf50	0.0358	0	-1.32E-05
PTEN	C13orf38	0.0358	0	-0.160998991
PTEN	TMC4	0.0358	0	6.57E-07
PTEN	ARV1	0.0358	0	0.020447171
PTEN	DPPA5	0.0358	0	-0.006781535
PTEN	SERPIND1	0.0358	0	-0.001818143
PTEN	KIAA0174	0.0358	0	-0.052794865
PTEN	LRAT	0.0358	0	-0.247442201
PTEN	URB1	0.0359	0	-0.119113458
PTEN	LOC286367	0.0359	0	-0.120876604
PTEN	LIN9	0.036	0	-0.380879904

PTEN	ASB9	0.036	0	-0.022269235
PTEN	ACSBG2	0.036	0	-0.002988047
PTEN	ODF4	0.036	0	-0.000686293
PTEN	ZFAND3	0.0361	0	-0.180032449
PTEN	SLC4A10	0.0361	0	-0.597492808
PTEN	TMEM123	0.0361	0	0.265266045
PTEN	HES2	0.0361	0	-0.039961575
PTEN	QTRTD1	0.0362	0	-0.158228555
PTEN	LIPC	0.0362	0	-0.173717783
PTEN	NUP35	0.0362	0	-0.146819166
PTEN	RAB22A	0.0362	0	0.015949724
PTEN	KCNJ16	0.0363	0	-0.205237986
PTEN	SLC7A11	0.0363	0	-0.209162975
PTEN	HAP1	0.0363	0	-0.011809811
PTEN	BIVM	0.0363	0	-0.26146393
PTEN	SPON2	0.0363	0	-0.07690103
PTEN	C11orf57	0.0363	0	-0.459099715
PTEN	PLGLA	0.0364	0	-0.116327993
PTEN	PRRT3	0.0365	0	-0.135057065
PTEN	B3GALNT2	0.0365	0	0.13266499
PTEN	THUMPD3	0.0365	0	-0.246390935
PTEN	SUB1	0.0365	0	0.313119855
PTEN	TFPI2	0.0365	0	-0.115716202
PTEN	SCN5A	0.0366	0	-0.373098129
PTEN	STRADA	0.0366	0	-0.000282571
PTEN	NOS2	0.0366	0	-0.135567108
PTEN	PRAMEF8	0.0366	0	-0.170169885
PTEN	SNORD48	0.0366	0	-1.14E-05
PTEN	LGSN	0.0367	0	-0.2792526
PTEN	SIP1	0.0367	0	-0.322519753
PTEN	C7orf64	0.0367	0	-0.524170419
PTEN	CLCC1	0.0367	0	0.227191744
PTEN	MYL9	0.0367	0	-0.000102579
PTEN	DAZ3	0.0368	0	-0.555583704
PTEN	ZNF764	0.0368	0	-0.036529757
PTEN	B3GNT1	0.0368	0	-0.183261826
PTEN	SNHG8	0.0368	0	0.000310287
PTEN	ZNF10	0.0369	0	-0.336992854
PTEN	KLRC4	0.0369	0	-0.191104838
PTEN	CCDC104	0.0369	0	-2.55E-06
PTEN	ZNF589	0.0369	0	-0.01742376

PTEN	PRPF38B	0.0369	0	-0.244383278
PTEN	CTNNA2	0.0369	0	-0.25511831
PTEN	DNAJA4	0.0369	0	0.054016253
PTEN	HMGN5	0.0369	0	-0.337283981
PTEN	RAD51L3	0.037	0	-0.033649915
PTEN	PTCHD3	0.037	0	-0.056125101
PTEN	KRT24	0.037	0	-0.007393206
PTEN	NACA2	0.037	0	-0.001998196
PTEN	PTH	0.037	0	-0.517567832
PTEN	SDHC	0.037	0	-0.017085587
PTEN	KRTAP12-2	0.037	0	-0.011482587
PTEN	SPPL3	0.0371	0	-0.038555987
PTEN	SPP2	0.0371	0	-0.173658789
PTEN	PPP1CB	0.0371	0	0.031144747
PTEN	ADAMTS17	0.0371	0	-0.169122798
PTEN	MED4	0.0371	0	-0.172786382
PTEN	SLC38A7	0.0371	0	0.157236832
PTEN	HOXC4	0.0372	0	-0.173222889
PTEN	ACADL	0.0372	0	-0.289387911
PTEN	SFRS14	0.0372	0	0.01015754
PTEN	RABEPK	0.0372	0	-0.003048575
PTEN	CASC5	0.0373	0	-0.030946249
PTEN	FAM47A	0.0373	0	-0.171096075
PTEN	IGFN1	0.0373	0	-0.001009245
PTEN	DDX47	0.0373	0	-0.046646265
PTEN	STARD3NL	0.0374	0	-0.185288525
PTEN	NEK3	0.0374	0	-0.018822766
PTEN	SNORD1A	0.0374	0	-1.78E-07
PTEN	RAB34	0.0375	0	0.038389263
PTEN	ACOX3	0.0375	0	-0.014296946
PTEN	DDX59	0.0375	0	-0.005177165
PTEN	DYDC2	0.0375	0	-0.201163683
PTEN	NPC1	0.0375	0	-0.368569857
PTEN	DNASE1L3	0.0375	0	-5.04E-05
PTEN	TACR1	0.0376	0	0.035649611
PTEN	OCRL	0.0376	0	-0.033385506
PTEN	ABI1	0.0376	0	-0.204300838
PTEN	CCDC88C	0.0376	0	-0.000939941
PTEN	DEFB106A	0.0376	0	-0.014670956
PTEN	C20orf118	0.0377	0	-0.408717428
PTEN	LOC646851	0.0377	0	-0.00660771

PTEN	C15orf24	0.0377	0	-0.14823253
PTEN	ARSE	0.0377	0	-0.011170603
PTEN	LOC91948	0.0377	0	-0.000896076
PTEN	COX7C	0.0378	0	1.77E-05
PTEN	CDH22	0.0378	0	-0.009158923
PTEN	IRAK2	0.0378	0	-0.408037028
PTEN	OSBPL5	0.0379	0	-0.036191615
PTEN	NLRP4	0.0379	0	-0.273546538
PTEN	NDEL1	0.0379	0	0.179885205
PTEN	EFCAB5	0.0379	0	-0.237129698
PTEN	RAB41	0.0379	0	-0.069840973
PTEN	BTF3L4	0.0379	0	0.048317778
PTEN	LTBP4	0.0379	0	0.000353439
PTEN	SNORD114-15	0.038	0	-1.09E-09
PTEN	LRSAM1	0.038	0	-0.02066295
PTEN	PSG11	0.038	0	-0.318218611
PTEN	HOXC9	0.038	0	-0.018587541
PTEN	CCRL2	0.038	0	-0.18967778
PTEN	MPPED2	0.038	0	-0.4938561
PTEN	WNT7B	0.038	0	-0.000170142
PTEN	LOC100128239	0.038	0	-0.018092039
PTEN	MAP6	0.0381	0	-0.107898597
PTEN	DYRK1B	0.0381	0	-0.241268227
PTEN	ATP2A3	0.0381	0	0.001634323
PTEN	CKAP4	0.0382	0	-0.05115934
PTEN	VPS11	0.0382	0	0.000861447
PTEN	TMEM90A	0.0382	0	-0.014374838
PTEN	C19orf42	0.0382	0	-0.091059749
PTEN	CYP4F3	0.0382	0	-0.164780874
PTEN	FOXD4L5	0.0383	0	-0.015781547
PTEN	MUC7	0.0383	0	-0.218126466
PTEN	SNORD56B	0.0384	0	-0.168002486
PTEN	P2RX6	0.0384	0	-0.009096808
PTEN	PECR	0.0384	0	-0.301256221
PTEN	SNORD115-41	0.0384	0	-1.20E-07
PTEN	SIGLEC14	0.0384	0	-0.004664121
PTEN	VCAM1	0.0384	0	0.13806265
PTEN	ACOT6	0.0384	0	-0.00013217
PTEN	AKR1C2	0.0384	0	-0.131665711
PTEN	TPRG1L	0.0384	0	0.174159907
PTEN	ZBBX	0.0385	0	-0.245057263

PTEN	BCL2L1	0.0385	0	0.167790939
PTEN	ISCA2	0.0385	0	-0.020473092
PTEN	SPDYE2	0.0385	0	-0.039078471
PTEN	TDGF3	0.0386	0	-0.218908323
PTEN	TMEM184B	0.0386	0	-0.021774948
PTEN	DSCR8	0.0386	0	-0.014832383
PTEN	RPLP0	0.0386	0	0.000462794
PTEN	TFAP2D	0.0386	0	-0.00193688
PTEN	FGL1	0.0386	0	-0.031107449
PTEN	RBMXL2	0.0386	0	-0.794820196
PTEN	GCM2	0.0387	0	-0.370594965
PTEN	TTC16	0.0387	0	-0.005652996
PTEN	RXRA	0.0387	0	0.016704339
PTEN	EIF4ENIF1	0.0387	0	-0.014381164
PTEN	HSP90AA1	0.0387	0	0.105523659
PTEN	SPSB4	0.0388	0	-0.040324259
PTEN	INHBC	0.0388	0	-0.003750129
PTEN	ZFP82	0.0388	0	-0.026686148
PTEN	ANKRD2	0.0388	0	-6.10E-09
PTEN	ADAMTS7	0.0388	0	-0.015711494
PTEN	VIP	0.0388	0	-0.442406116
PTEN	CD1E	0.0388	0	-0.166005639
PTEN	MUC2	0.0389	0	-0.010399053
PTEN	BST2	0.0389	0	2.20E-07
PTEN	PITPNC1	0.0389	0	-0.133006933
PTEN	APOL5	0.0389	0	-0.166709764
PTEN	AFMID	0.039	0	-0.137284679
PTEN	AMPD3	0.039	0	-0.406570549
PTEN	AGA	0.0391	0	-0.04045065
PTEN	IRX5	0.0391	0	-0.05877996
PTEN	HIPK4	0.0392	0	-0.000896994
PTEN	BTG4	0.0392	0	-0.420477674
PTEN	CMPK2	0.0393	0	-0.372140486
PTEN	TRA2B	0.0393	0	0.010019995
PTEN	CDC42SE1	0.0393	0	0.050659225
PTEN	ASPHD2	0.0393	0	-0.156042282
PTEN	NDUFA5	0.0393	0	0.006295946
PTEN	KRTAP4-1	0.0393	0	-0.018882657
PTEN	DCAF11	0.0393	0	-0.120887636
PTEN	ARL6IP6	0.0394	0	-0.033274703
PTEN	C6orf176	0.0394	0	-0.253870737

PTEN	FAM95B1	0.0394	0	-0.01337252
PTEN	B2M	0.0395	0	0.027806836
PTEN	MORN5	0.0395	0	-0.000295283
PTEN	GIMAP4	0.0395	0	-0.379061412
PTEN	GUCY2D	0.0396	0	-0.001465516
PTEN	TULP3	0.0396	0	-0.069890231
PTEN	THEMIS	0.0396	0	-0.60122073
PTEN	IL4	0.0396	0	-0.226136634
PTEN	NCRNA00183	0.0396	0	-0.109361254
PTEN	WDR37	0.0396	0	-0.292733059
PTEN	NUP43	0.0396	0	-0.156005333
PTEN	THUMPD2	0.0396	0	-0.190335419
PTEN	FAM66D	0.0396	0	-0.165961113
PTEN	VSNL1	0.0396	0	-0.095497382
PTEN	CCDC60	0.0397	0	-0.176674685
PTEN	PFKM	0.0397	0	-0.135116484
PTEN	SLC19A3	0.0397	0	-0.034599722
PTEN	IFT172	0.0397	0	1.87E-05
PTEN	AGPAT4	0.0398	0	-0.055214283
PTEN	TAS2R5	0.0398	0	-0.000263196
PTEN	KRTAP9-2	0.0398	0	-0.001525998
PTEN	IFITM1	0.0399	0	0.000795987
PTEN	C4orf14	0.0399	0	-0.02725544
PTEN	RFWD2	0.04	0	-0.112004655
PTEN	DEFB110	0.04	0	-0.137476891
PTEN	PPP1R3G	0.04	0	-0.000974539
PTEN	LRRC20	0.0401	0	-0.00442037
PTEN	MAP7D3	0.0401	0	0.013783167
PTEN	KIAA0226	0.0401	0	-0.107005274
PTEN	KLHDC9	0.0401	0	-0.16340204
PTEN	ITPK1	0.0401	0	0.014696235
PTEN	AMELX	0.0401	0	-0.191333162
PTEN	LOC113230	0.0402	0	0.000229782
PTEN	PITX3	0.0402	0	-0.007138707
PTEN	CDH7	0.0402	0	-0.000124216
PTEN	ADAMTS10	0.0402	0	-0.013662105
PTEN	RPS24	0.0403	0	-0.110278796
PTEN	E2F6	0.0403	0	-0.044431798
PTEN	FAM160A2	0.0403	0	-0.090026826
PTEN	TGDS	0.0403	0	0.001092004
PTEN	AK3L1	0.0403	0	-0.26432662

PTEN	OTUD7A	0.0404	0	-4.87E-07
PTEN	RPRD1B	0.0404	0	-0.231521988
PTEN	ABCC6P1	0.0404	0	-0.713989254
PTEN	FAM125B	0.0404	0	-0.170900266
PTEN	MASP2	0.0406	0	-0.130963947
PTEN	TMC7	0.0406	0	-0.337939781
PTEN	ITPRIP	0.0406	0	-0.14473944
PTEN	LGR4	0.0406	0	-0.369101097
PTEN	LOC285954	0.0406	0	-0.482773148
PTEN	MTA3	0.0407	0	-0.045043895
PTEN	KRTAP9-8	0.0407	0	-0.001527521
PTEN	DYNC1I1	0.0407	0	-0.196033832
PTEN	HNRNPC	0.0407	0	0.025135942
PTEN	PLA2G4C	0.0407	0	-0.131909514
PTEN	SSX2	0.0408	0	-0.001804638
PTEN	DOCK6	0.0408	0	-0.080267053
PTEN	GOSR1	0.0409	0	-0.171126466
PTEN	LOC653545	0.0408	0	-3.51E-05
PTEN	THEM4	0.0409	0	-0.120461509
PTEN	CCDC127	0.0409	0	-0.224651496
PTEN	PAK1	0.0409	0	-0.001555978
PTEN	PCSK4	0.0409	0	-4.42E-06
PTEN	C1orf185	0.0409	0	-0.223555649
PTEN	NCRNA00173	0.0409	0	-0.155663368
PTEN	C7orf10	0.041	0	-0.004850939
PTEN	MEGF8	0.041	0	-0.114635583
PTEN	ELFN1	0.041	0	-0.203911713
PTEN	NCRNA00161	0.0411	0	-0.027023755
PTEN	SH3GL2	0.0411	0	-0.198486812
PTEN	ZC3H15	0.0411	0	0.033584984
PTEN	SIAH3	0.0411	0	-0.13611588
PTEN	KLHDC8A	0.0413	0	-0.249899887
PTEN	UCP2	0.0413	0	0.000143444
PTEN	ZW10	0.0413	0	-0.001419654
PTEN	MS4A6E	0.0413	0	-0.005659036
PTEN	PCDHA9	0.0413	0	-0.725280731
PTEN	TDRD12	0.0414	0	-3.37E-08
PTEN	DCD	0.0415	0	0.103177635
PTEN	MTMR4	0.0415	0	-0.057625776
PTEN	KRT20	0.0415	0	-0.249707274
PTEN	TMEM114	0.0415	0	-2.25E-06

PTEN	IL2	0.0415	0	-0.189205275
PTEN	CIB1	0.0415	0	6.66E-06
PTEN	FAT2	0.0415	0	-0.174227267
PTEN	TLR8	0.0416	0	-0.428696002
PTEN	IGSF6	0.0416	0	-0.099533273
PTEN	SOX2	0.0416	0	-0.199901202
PTEN	SULT2A1	0.0416	0	-0.200950662
PTEN	FBXO48	0.0416	0	-0.36378677
PTEN	CPXCR1	0.0416	0	-0.066486822
PTEN	CMIP	0.0416	0	-0.019798914
PTEN	KREMEN1	0.0416	0	-0.015097578
PTEN	TMX4	0.0417	0	-0.105419525
PTEN	HS3ST4	0.0417	0	-0.327244421
PTEN	LRRC42	0.0417	0	0.029052505
PTEN	SLC22A2	0.0418	0	-0.180126311
PTEN	LOC100133920	0.0418	0	-0.297059116
PTEN	TMEM110	0.0418	0	-0.00201538
PTEN	DAPP1	0.0419	0	0.001809441
PTEN	GORASP1	0.0419	0	-0.020960012
PTEN	ASAP3	0.0419	0	-0.018826965
PTEN	MRPS16	0.0419	0	0.001061953
PTEN	ZIM3	0.042	0	-0.053583667
PTEN	SETD5	0.0421	0	0.168561485
PTEN	C9orf117	0.0421	0	-0.012162241
PTEN	FLJ42627	0.0421	0	-1.33E-06
PTEN	CNGB3	0.0421	0	-0.456799093
PTEN	RNF139	0.0421	0	0.125212141
PTEN	GATA1	0.0421	0	-0.168724689
PTEN	ALAS2	0.0421	0	-0.000244535
PTEN	REN	0.0421	0	-0.000232593
PTEN	PKD1L2	0.0422	0	-0.122764524
PTEN	CEACAM3	0.0422	0	-0.000471965
PTEN	MFSD11	0.0422	0	-4.22E-05
PTEN	ZNF565	0.0422	0	-0.010719892
PTEN	STMN4	0.0424	0	-0.128147301
PTEN	DYNC1LI1	0.0424	0	-0.162943206
PTEN	ODF3L1	0.0424	0	-0.000109371
PTEN	TMEM92	0.0424	0	-0.019489296
PTEN	ZSCAN22	0.0425	0	-0.069205495
PTEN	BRCA2	0.0425	0	-0.16861326
PTEN	CHST3	0.0425	0	-0.263655335

PTEN	ANXA3	0.0425	0	-0.28781888
PTEN	ESF1	0.0425	0	0.13808532
PTEN	NR1D1	0.0426	0	-0.156993344
PTEN	CCDC28A	0.0426	0	-0.153007792
PTEN	PCYOX1L	0.0427	0	-0.014359824
PTEN	NT5C2	0.0428	0	-0.027412947
PTEN	KRTAP10-7	0.0428	0	-0.097098729
PTEN	HELLS	0.0428	0	0.088177217
PTEN	OR10W1	0.0428	0	-0.027594278
PTEN	TAF12	0.0429	0	-0.154200758
PTEN	FAM185A	0.0429	0	-0.167061409
PTEN	PSD2	0.0429	0	-0.197873329
PTEN	GORASP2	0.0429	0	-0.13976155
PTEN	CREB3	0.043	0	-0.005854794
PTEN	GLT25D2	0.0431	0	-0.171390199
PTEN	AADACL2	0.0431	0	-0.002026063
PTEN	TTC1	0.0431	0	-0.13156759
PTEN	RP1-177G6.2	0.0431	0	-0.006426786
PTEN	CRTC1	0.0432	0	-0.002318044
PTEN	PYY	0.0432	0	-6.32E-05
PTEN	KCNA3	0.0432	0	-0.461710058
PTEN	NAGS	0.0432	0	-5.19E-05
PTEN	KIAA0182	0.0432	0	-0.005603499
PTEN	CDH1	0.0432	0	0.131611874
PTEN	RNF138	0.0432	0	0.006390751
PTEN	MSRA	0.0433	0	-0.019728422
PTEN	ADAM3A	0.0433	0	-0.031449928
PTEN	KCNB1	0.0434	0	-0.181687411
PTEN	LOC338651	0.0434	0	-0.005909223
PTEN	RBM46	0.0435	0	-0.14754325
PTEN	KIAA1191	0.0435	0	0.044284667
PTEN	SEMA3F	0.0435	0	-0.010142929
PTEN	TAC4	0.0436	0	-0.230063665
PTEN	SLC5A8	0.0436	0	-0.221153565
PTEN	TSPAN12	0.0436	0	-0.290194721
PTEN	ART1	0.0436	0	-0.010035959
PTEN	ACTR3	0.0437	0	0.172752226
PTEN	C14orf50	0.0437	0	-4.16E-05
PTEN	CCDC54	0.0437	0	-0.000914164
PTEN	GGT5	0.0437	0	-1.49E-06
PTEN	FAM149A	0.0437	0	-0.14900757

PTEN	CBFA2T2	0.0438	0	-0.215018408
PTEN	12-Sep	0.0439	0	-0.011150294
PTEN	HIF3A	0.0439	0	-0.135131997
PTEN	CAMK1	0.0439	0	0.001745411
PTEN	LOC285375	0.044	0	-0.004251356
PTEN	C2orf58	0.044	0	-0.295850964
PTEN	TIAM2	0.044	0	-0.352540211
PTEN	GBP2	0.044	0	-0.11855654
PTEN	OAS1	0.0441	0	-0.086067944
PTEN	CXorf57	0.0441	0	-0.288587619
PTEN	SLAMF9	0.0441	0	-0.007681085
PTEN	PLSCR1	0.0441	0	-0.319874
PTEN	LPCAT3	0.0442	0	-0.00233872
PTEN	PIWIL1	0.0442	0	-0.040007048
PTEN	MYD88	0.0442	0	0.14710991
PTEN	SLC25A18	0.0442	0	-0.007499864
PTEN	TIPRL	0.0443	0	-0.298074383
PTEN	TUB	0.0443	0	-0.070685787
PTEN	C12orf41	0.0444	0	-0.184331661
PTEN	GPR52	0.0444	0	-0.26579758
PTEN	RHBDL1	0.0445	0	-0.019719323
PTEN	MTL5	0.0445	0	-0.157289734
PTEN	FASN	0.0445	0	0.000127975
PTEN	TPD52L3	0.0445	0	-0.263489888
PTEN	ASB15	0.0446	0	-0.320274686
PTEN	JMJD5	0.0446	0	-0.0105419
PTEN	KLHL38	0.0446	0	-3.63E-07
PTEN	GAS2L3	0.0446	0	-4.12E-06
PTEN	TM7SF2	0.0446	0	0.000219208
PTEN	AQP7P3	0.0446	0	-0.174040542
PTEN	HMGA2	0.0446	0	-0.399037294
PTEN	ARL4C	0.0447	0	0.099926267
PTEN	ELMO1	0.0448	0	-0.094438078
PTEN	CLDN10	0.0448	0	-0.117752039
PTEN	EPB41L3	0.0448	0	-0.078723111
PTEN	HDAC9	0.0448	0	0.047280968
PTEN	LRRN2	0.0449	0	0.000390414
PTEN	SRP19	0.0449	0	-0.311016295
PTEN	PPP4R4	0.0449	0	-0.134569268
PTEN	PMS2L2	0.0449	0	-0.095500389
PTEN	SAMSN1	0.0449	0	-0.306552271

PTEN	BRCC3	0.045	0	-0.082000908
PTEN	PDHB	0.045	0	-0.070280511
PTEN	OSTBETA	0.045	0	-5.82E-05
PTEN	RNF39	0.045	0	-0.263424023
PTEN	SNORD98	0.045	0	-0.175850502
PTEN	DPH3	0.045	0	-0.184307365
PTEN	OR51B2	0.045	0	-0.000197709
PTEN	SNORD11	0.0451	0	-0.010524259
PTEN	TMEM220	0.0451	0	-0.379584128
PTEN	AKAP14	0.0451	0	-0.004798786
PTEN	C4BPA	0.0451	0	-0.007270879
PTEN	APCDD1	0.0451	0	-0.02722501
PTEN	ISG20L2	0.0452	0	0.001810297
PTEN	EID3	0.0452	0	-0.170048155
PTEN	CTSD	0.0452	0	0.001437965
PTEN	NRAP	0.0452	0	-0.000135607
PTEN	ABCA3	0.0452	0	-0.017096854
PTEN	PGAP3	0.0452	0	-0.054402612
PTEN	IFIH1	0.0452	0	-0.097531771
PTEN	GLA	0.0453	0	-2.72E-06
PTEN	SHC2	0.0453	0	-0.001000805
PTEN	HPR	0.0453	0	-0.002679912
PTEN	ZC3H8	0.0453	0	-0.036321619
PTEN	APOL3	0.0454	0	-0.129598338
PTEN	UGT1A6	0.0454	0	-0.133467841
PTEN	AIMP1	0.0454	0	-0.230024591
PTEN	RPL34	0.0454	0	-0.004868754
PTEN	MGC57346	0.0454	0	-0.233705051
PTEN	CLDN5	0.0455	0	-0.146133229
PTEN	ZNF286A	0.0455	0	0.086683276
PTEN	CCRN4L	0.0455	0	-0.212683302
PTEN	SORD	0.0455	0	0.09534879
PTEN	DEPDC5	0.0456	0	-0.125626737
PTEN	ZNF646	0.0456	0	-0.101936338
PTEN	HBG1	0.0456	0	-0.007428346
PTEN	JPH3	0.0456	0	-0.243145059
PTEN	LMO2	0.0456	0	-0.106152758
PTEN	BAG2	0.0457	0	-0.046739439
PTEN	S100G	0.0457	0	-0.040745825
PTEN	TMPRSS2	0.0458	0	-0.192395551
PTEN	METTL13	0.0458	0	-0.142400274

PTEN	DSC1	0.0458	0	-0.309381319
PTEN	C17orf105	0.0458	0	-0.003645457
PTEN	LRRC34	0.0458	0	-0.033128588
PTEN	SNORD114-27	0.0458	0	-0.00162415
PTEN	PIGR	0.0459	0	-0.00685358
PTEN	GADL1	0.0459	0	-0.614388371
PTEN	SREBF1	0.0459	0	-0.108553793
PTEN	ADIPOR1	0.0459	0	0.004859023
PTEN	HSD17B8	0.0459	0	6.48E-05
PTEN	CNKSR2	0.0459	0	-0.017701422
PTEN	GNE	0.0459	0	-0.232215797
PTEN	ARL17A	0.046	0	0.074328926
PTEN	PTF1A	0.046	0	-0.44084854
PTEN	C16orf63	0.046	0	-0.193168189
PTEN	USP6	0.046	0	-0.343972077
PTEN	PIK3C2G	0.0461	0	-0.258813225
PTEN	ZNF174	0.0462	0	-0.189318205
PTEN	C7orf23	0.0462	0	-0.167472158
PTEN	SNORD113-1	0.0462	0	-4.21E-05
PTEN	ZAN	0.0462	0	-0.000386102
PTEN	PHACTR1	0.0463	0	-0.00194256
PTEN	SOCS2	0.0463	0	-0.132997121
PTEN	LYPD1	0.0463	0	-0.142283796
PTEN	HBII-52-45	0.0463	0	-0.001314118
PTEN	CST8	0.0463	0	-0.00794877
PTEN	ANKRD53	0.0464	0	-0.000237544
PTEN	RANBP10	0.0464	0	0.136096928
PTEN	RAPGEF1	0.0464	0	0.010129295
PTEN	C13orf28	0.0464	0	-1.14E-05
PTEN	HDHD1A	0.0464	0	-0.008423036
PTEN	R3HDML	0.0464	0	-0.028021653
PTEN	TPPP3	0.0465	0	-2.29E-05
PTEN	ZNF204P	0.0465	0	-0.13632302
PTEN	KIAA1614	0.0465	0	0.001058668
PTEN	MON1B	0.0465	0	-0.034736864
PTEN	CHPT1	0.0465	0	0.126517509
PTEN	AMN1	0.0466	0	-0.366679462
PTEN	ARHGEF6	0.0466	0	0.120737887
PTEN	SKA2	0.0466	0	0.04566272
PTEN	WDR86	0.0466	0	-0.174119408
PTEN	GPHN	0.0467	0	-0.322014986

PTEN	ADAMTS8	0.0467	0	-0.102564844
PTEN	IQCD	0.0467	0	-0.02592941
PTEN	NXPH2	0.0467	0	-0.224922636
PTEN	TBL1X	0.0467	0	0.13908993
PTEN	FAM43A	0.0468	0	-0.034372642
PTEN	CDC14C	0.0469	0	-0.567165814
PTEN	TBRG1	0.0468	0	-0.02366349
PTEN	CLIC2	0.047	0	-0.19479315
PTEN	RNF135	0.047	0	0.149307848
PTEN	PI16	0.047	0	-0.004248296
PTEN	PPP1R8	0.047	0	-0.02018898
PTEN	KCTD4	0.0471	0	-0.51482057
PTEN	TPPP	0.0471	0	-0.121166801
PTEN	AMBP	0.0471	0	-3.26E-06
PTEN	PAQR8	0.0471	0	-0.389869397
PTEN	DSP	0.0471	0	0.190303171
PTEN	FAM118B	0.0472	0	-0.022776641
PTEN	HSD3B2	0.0472	0	-0.114368753
PTEN	C12orf54	0.0472	0	-0.017408451
PTEN	ZNHIT6	0.0472	0	-0.248192488
PTEN	PIGA	0.0472	0	-0.135231208
PTEN	CUX1	0.0472	0	-0.158780102
PTEN	FAM109B	0.0473	0	0.139144449
PTEN	DBC1	0.0473	0	-0.081427573
PTEN	KIAA1383	0.0473	0	-0.271203221
PTEN	HN1L	0.0473	0	0.118924557
PTEN	WRB	0.0474	0	-0.017474362
PTEN	TAOK2	0.0474	0	-0.02194084
PTEN	ATP5SL	0.0474	0	-0.156202117
PTEN	ZC4H2	0.0474	0	-0.21922781
PTEN	SALL3	0.0474	0	-0.105999066
PTEN	NCRNA00207	0.0474	0	-0.000473111
PTEN	FAM3C	0.0475	0	0.138634888
PTEN	SIX1	0.0475	0	0.024017229
PTEN	ATP9A	0.0475	0	0.030186319
PTEN	GPR115	0.0475	0	-0.1032482
PTEN	KRTAP2-1	0.0475	0	-0.000410138
PTEN	IGDCC3	0.0476	0	-0.012000158
PTEN	GSN	0.0476	0	-0.014408637
PTEN	MEPE	0.0476	0	-0.038452727
PTEN	HDAC3	0.0476	0	0.001148525

PTEN	MATN2	0.0477	0	-0.148456359
PTEN	EMILIN1	0.0476	0	6.38E-08
PTEN	EIF3F	0.0477	0	0.003871964
PTEN	CHRNA7	0.0477	0	-0.234102878
PTEN	GPR148	0.0477	0	-0.160902289
PTEN	IL17RD	0.0478	0	-0.004916362
PTEN	ETFA	0.0478	0	0.020300096
PTEN	TEX11	0.0478	0	-0.008299654
PTEN	RAB39	0.0478	0	-0.369109681
PTEN	C12orf76	0.0478	0	-0.36763881
PTEN	METTL12	0.0479	0	-0.023215563
PTEN	TBXAS1	0.0479	0	-0.0001611
PTEN	UNC5CL	0.0479	0	-0.019176606
PTEN	ABHD6	0.0479	0	0.110085394
PTEN	SOAT2	0.0479	0	-0.169945725
PTEN	RPL13AP6	0.0479	0	-4.19E-08
PTEN	GSTT2	0.0479	0	-0.041606673
PTEN	BCL2L13	0.0479	0	-0.1692894
PTEN	SRR	0.0479	0	-0.010959725
PTEN	RNF24	0.048	0	-0.268127743
PTEN	COL4A1	0.0481	0	0.225667026
PTEN	VAT1L	0.0481	0	-0.22998316
PTEN	MAN2A2	0.0481	0	-0.029392534
PTEN	TMEM43	0.0481	0	-0.250586442
PTEN	NPTX2	0.0482	0	-0.290638367
PTEN	TLE6	0.0482	0	-0.00120075
PTEN	ZNF501	0.0482	0	-0.267964791
PTEN	PLIN3	0.0482	0	0.034265209
PTEN	HFM1	0.0482	0	-0.354322017
PTEN	NR6A1	0.0483	0	-0.007066035
PTEN	LOC121952	0.0483	0	-0.495614216
PTEN	HNRNPA0	0.0483	0	0.146421541
PTEN	ZNF157	0.0484	0	-0.029087982
PTEN	PADI3	0.0484	0	-0.153841673
PTEN	ST3GAL2	0.0484	0	-0.031472116
PTEN	LYPD4	0.0485	0	-4.22E-06
PTEN	CMTM8	0.0485	0	0.030478975
PTEN	TEC	0.0485	0	0.01398899
PTEN	NCOA6	0.0485	0	-0.341526775
PTEN	IGFBP3	0.0485	0	-0.012506816
PTEN	ACOT12	0.0485	0	-0.192261023

PTEN	OR52N4	0.0485	0	-5.30E-07
PTEN	FAM66C	0.0486	0	-0.348020028
PTEN	PPCS	0.0486	0	0.088623223
PTEN	RFFL	0.0486	0	0.141313505
PTEN	PCSK2	0.0488	0	-0.106054405
PTEN	ORC5L	0.0488	0	-0.234693687
PTEN	ACLY	0.0489	0	-0.026313555
PTEN	CEP78	0.0489	0	-0.139973934
PTEN	TNP2	0.0489	0	-0.008957766
PTEN	C12orf53	0.0489	0	-0.021658012
PTEN	IQCF6	0.049	0	-0.000365471
PTEN	EXOC2	0.049	0	-0.056085445
PTEN	RPL10	0.0491	0	0.025989107
PTEN	ATP5G2	0.0492	0	0.002673239
PTEN	NGLY1	0.0492	0	-0.043370558
PTEN	LOC286238	0.0492	0	-0.260623027
PTEN	PSD	0.0492	0	-0.222194388
PTEN	PTGIS	0.0493	0	0.088778331
PTEN	MARCKS	0.0493	0	0.185517646
PTEN	YES1	0.0494	0	0.089774734
PTEN	BTG1	0.0494	0	0.118509406
PTEN	SSBP3	0.0494	0	-0.027479694
PTEN	APOL4	0.0495	0	-0.190550469
PTEN	IPO9	0.0495	0	2.55E-05
PTEN	PRKD3	0.0495	0	-0.122494603
PTEN	DLX1	0.0495	0	-0.210874527
PTEN	GLRX	0.0495	0	-0.006169415
PTEN	MAGEB18	0.0495	0	-0.41245817
PTEN	S100A16	0.0496	0	0.028249848
PTEN	C10orf95	0.0496	0	-0.001552749
PTEN	CD63	0.0497	0	0.000338291
PTEN	NFXL1	0.0497	0	-0.198896616
PTEN	DNAJB8	0.0497	0	-1.01E-06
PTEN	LOC647288	0.0497	0	-0.498288553
PTEN	TSPYL6	0.0497	0	-0.188194765
PTEN	SARM1	0.0497	0	-0.004501371
PTEN	C15orf26	0.0498	0	-0.022642805
PTEN	XYLT2	0.0498	0	-0.018925731
PTEN	PLEKHH1	0.0498	0	-0.295913314
PTEN	HMGCLL1	0.0498	0	-0.385347784
PTEN	SPATA5L1	0.0498	0	-0.267748892

PTEN	GNAI2	0.0499	0	0.130868412
PTEN	FOXI1	0.0499	0	-0.010007418
PTEN	GATA2	0.0499	0	-0.066678341
PTEN	NR4A3	0.0499	0	-0.517115094
PTEN	MFSD6L	0.05	0	-0.001077382
PTEN	GAB2	0.05	0	-0.006944079
PTEN	NCRNA00219	0.05	0	0.152893025
PTEN	SCGB3A1	0.0501	0	0.002327348
PTEN	C9orf128	0.0501	0	-0.329657896
PTEN	MS4A4A	0.0501	0	-0.141039597
PTEN	KCNA5	0.0501	0	-0.081904534
PTEN	PON1	0.0501	0	-0.009533424
PTEN	KLF17	0.0501	0	-0.369109484
PTEN	CALM2	0.0502	0	0.373294711
PTEN	MLLT10	0.0502	0	-0.004105668
PTEN	NXT2	0.0502	0	-0.20861805
PTEN	SSR1	0.0502	0	2.44E-07
PTEN	TMED2	0.0502	0	0.203079538
PTEN	OSBPL7	0.0503	0	-0.150882962
PTEN	CLDN8	0.0503	0	-0.479109934
PTEN	SLC2A14	0.0503	0	-0.012614053
PTEN	PLEKHO2	0.0503	0	0.049506897
PTEN	MAP2	0.0503	0	-0.286464877
PTEN	SGK3	0.0503	0	-0.098149852
PTEN	CELF6	0.0503	0	-0.25451989
PTEN	PSG3	0.0504	0	-0.42681129
PTEN	STAB1	0.0504	0	-0.001054919
PTEN	ATP2C1	0.0504	0	-0.142136168
PTEN	CPM	0.0504	0	-0.079134867
PTEN	TMOD2	0.0504	0	-0.005311819
PTEN	PAGE4	0.0505	0	-0.184775666
PTEN	PCDHB3	0.0505	0	-0.036510859
PTEN	PTK2B	0.0505	0	-0.011728847
PTEN	ANXA2P3	0.0505	0	-0.003351993
PTEN	SLC22A12	0.0506	0	-0.102983943
PTEN	DENND2C	0.0507	0	-0.134642262
PTEN	RNF146	0.0507	0	0.057507091
PTEN	MFSD2A	0.0508	0	-0.127132465
PTEN	ACTN1	0.0508	0	0.156154861
PTEN	KLF11	0.0509	0	-0.138515014
PTEN	TMEM69	0.0509	0	0.142103116

PTEN	ENSA	0.0509	0	0.117601255
PTEN	PPP2R4	0.0509	0	-0.126767482
PTEN	SAP130	0.051	0	-0.075973982
PTEN	MRPL44	0.0512	0	-0.237839121
PTEN	LRRIQ3	0.0512	0	-0.047976489
PTEN	STX3	0.0512	0	-0.316340376
PTEN	ZNF334	0.0512	0	-0.121331457
PTEN	LY96	0.0512	0	-0.020246119
PTEN	CA8	0.0512	0	-0.13443866
PTEN	GCNT1	0.0513	0	-0.271269218
PTEN	NGFR	0.0514	0	-0.004995557
PTEN	CPNE9	0.0514	0	-0.000513261
PTEN	PNPLA7	0.0514	0	-0.00030105
PTEN	SERHL	0.0514	0	-0.004145086
PTEN	AKIRIN1	0.0515	0	0.034097353
PTEN	PRRT2	0.0515	0	0.142872492
PTEN	POU6F1	0.0516	0	-0.14931458
PTEN	SH3YL1	0.0516	0	0.15218457
PTEN	SCGB1C1	0.0516	0	-4.35E-07
PTEN	IL1B	0.0516	0	-0.147850621
PTEN	CEACAM8	0.0517	0	-0.203837003
PTEN	DCHS2	0.0517	0	-0.289980012
PTEN	OTOA	0.0517	0	-0.000325771
PTEN	ZNRF3	0.0517	0	-0.029642174
PTEN	PCK1	0.0518	0	-0.189494917
PTEN	PLOD2	0.0519	0	-0.185777362
PTEN	GAMT	0.0519	0	-0.135414012
PTEN	NPAS2	0.0519	0	-0.060753053
PTEN	BTBD11	0.052	0	-0.354333334
PTEN	CCDC109B	0.052	0	-0.001040558
PTEN	PIGF	0.052	0	-0.041880793
PTEN	PAX3	0.052	0	-0.396395424
PTEN	OPA3	0.0521	0	-0.024208402
PTEN	FIGN	0.0521	0	-0.464453819
PTEN	TDRD5	0.0521	0	-0.054233306
PTEN	RNF19B	0.0521	0	-0.260242633
PTEN	PRSSL1	0.0522	0	-0.000247906
PTEN	PSMB11	0.0522	0	-0.014193884
PTEN	TGIF1	0.0522	0	-0.195682029
PTEN	RAB32	0.0522	0	0.16446778
PTEN	RRH	0.0522	0	-0.211979831

PTEN	SNAPC1	0.0523	0	-0.176838313
PTEN	C2orf65	0.0523	0	0.133650243
PTEN	CLVS1	0.0523	0	-0.472056351
PTEN	PLA2G4F	0.0523	0	-0.12388365
PTEN	RPL10L	0.0524	0	-0.159243289
PTEN	LRRK1	0.0524	0	-0.236535203
PTEN	CORO1C	0.0524	0	0.034713583
PTEN	RASL12	0.0525	0	0.101591861
PTEN	CDRT4	0.0525	0	-0.020055115
PTEN	PHF7	0.0525	0	-0.000884746
PTEN	ZNF83	0.0526	0	-0.082262461
PTEN	TSPAN3	0.0526	0	-0.075136873
PTEN	C5orf58	0.0526	0	-0.020515941
PTEN	POLE3	0.0526	0	0.001138825
PTEN	C2orf3	0.0526	0	-0.281480269
PTEN	ATP6V0A2	0.0527	0	0.126019363
PTEN	C7orf57	0.0528	0	-0.257257804
PTEN	CELA2B	0.0528	0	-0.002598927
PTEN	MXI1	0.0528	0	0.005963553
PTEN	IFNK	0.0528	0	-0.016421301
PTEN	IFI44L	0.0528	0	-0.016541147
PTEN	SLC6A5	0.0528	0	-0.000264365
PTEN	C14orf93	0.0528	0	-3.76E-05
PTEN	MGC27382	0.0528	0	-0.000704074
PTEN	ACOT1	0.0528	0	-0.001130227
PTEN	VPS18	0.0529	0	-0.101405406
PTEN	CA7	0.0529	0	-0.155468342
PTEN	ZMYND12	0.0529	0	-0.008562871
PTEN	RNF113B	0.053	0	-0.007218986
PTEN	LCORL	0.053	0	-0.43300957
PTEN	NACA	0.053	0	-0.014044588
PTEN	COQ5	0.053	0	-0.005665679
PTEN	GRHL3	0.053	0	-0.184201746
PTEN	VSTM1	0.0531	0	-0.001846121
PTEN	ITIH2	0.0531	0	-0.236099255
PTEN	DDX1	0.0531	0	-0.12623994
PTEN	SUMF1	0.0531	0	0.064007705
PTEN	TARDBP	0.0532	0	-0.008288546
PTEN	MED17	0.0533	0	-0.343672994
PTEN	MTO1	0.0534	0	-0.034717222
PTEN	AVPR2	0.0534	0	-0.002247852

PTEN	NEURL1B	0.0534	0	0.148805737
PTEN	TCERG1L	0.0534	0	-0.030255044
PTEN	C14orf179	0.0534	0	-0.036179968
PTEN	LRRC37B	0.0534	0	-0.008811936
PTEN	EGFR	0.0534	0	0.013810636
PTEN	ZNF345	0.0534	0	-0.01870571
PTEN	KCNJ6	0.0535	0	-0.576794782
PTEN	GCNT3	0.0535	0	-0.126252435
PTEN	AKR7A2	0.0535	0	-0.000247603
PTEN	YPEL4	0.0535	0	-0.035425651
PTEN	P4HTM	0.0535	0	-0.000277155
PTEN	C6orf25	0.0536	0	-0.000398346
PTEN	NKX3-2	0.0537	0	-0.119439272
PTEN	LY6H	0.0537	0	-0.149152309
PTEN	CD69	0.0537	0	-0.474097423
PTEN	FAM49A	0.0537	0	-0.02874197
PTEN	EFNB3	0.0537	0	-0.160874007
PTEN	KIF17	0.0539	0	-0.006732431
PTEN	C19orf75	0.0539	0	-0.037352797
PTEN	LOC283314	0.0539	0	-0.000182357
PTEN	MAS1	0.0539	0	-0.011217212
PTEN	PLEKHG1	0.0539	0	-0.251899109
PTEN	PRKRIR	0.054	0	0.109631886
PTEN	SERPINB9	0.054	0	-0.024411889
PTEN	SR140	0.054	0	-0.020277963
PTEN	CCDC13	0.054	0	-0.000524701
PTEN	FAAH	0.054	0	-9.95E-06
PTEN	VN1R1	0.0541	0	-0.002330026
PTEN	DEK	0.0541	0	-0.143018272
PTEN	C1GALT1	0.0541	0	-0.219608923
PTEN	ZNF554	0.0541	0	-0.089389389
PTEN	MFRP	0.0542	0	-0.150877538
PTEN	SCARNA1	0.0542	0	-0.041298716
PTEN	TAS2R1	0.0542	0	-0.000105079
PTEN	TRNT1	0.0543	0	-0.252153062
PTEN	RND3	0.0543	0	0.164267806
PTEN	BMP5	0.0543	0	-0.002523948
PTEN	DEFA6	0.0545	0	-0.007752124
PTEN	VIM	0.0545	0	0.173087504
PTEN	VRK3	0.0545	0	-0.022893898
PTEN	MXD4	0.0545	0	-0.000868537

PTEN	LOC643837	0.0545	0	0.012053133
PTEN	PDLIM3	0.0545	0	-0.021328551
PTEN	4-Mar	0.0545	0	-0.215114526
PTEN	PARP1	0.0546	0	-0.096283805
PTEN	CSNK2A1	0.0546	0	-0.029245742
PTEN	FBXO9	0.0547	0	-0.076105495
PTEN	IRX4	0.0547	0	-0.222571943
PTEN	C11orf61	0.0547	0	-0.2532126
PTEN	CDC16	0.0547	0	4.72E-05
PTEN	RRP8	0.0548	0	-0.000336039
PTEN	ZNF529	0.0548	0	0.088274811
PTEN	PTGS2	0.0548	0	-0.198519419
PTEN	RNF157	0.0549	0	-0.184772727
PTEN	ZNF215	0.0549	0	-0.421420869
PTEN	LRRC55	0.055	0	-0.176298624
PTEN	SOX13	0.055	0	0.159989248
PTEN	NUP50	0.055	0	-0.048606208
PTEN	FAM46B	0.0551	0	-0.130110208
PTEN	TNMD	0.0551	0	-0.000425977
PTEN	DCK	0.0552	0	-0.112868536
PTEN	NXF3	0.0552	0	-0.011793977
PTEN	CIC	0.0552	0	-0.006413689
PTEN	CRAMP1L	0.0552	0	-0.013112965
PTEN	MED31	0.0552	0	0.003733985
PTEN	COMMD2	0.0553	0	-0.036399736
PTEN	ASPRV1	0.0553	0	-0.001545161
PTEN	DLG3	0.0553	0	-0.138665047
PTEN	TAS2R13	0.0553	0	-0.007725573
PTEN	SLC35A1	0.0553	0	-0.203953754
PTEN	ZBTB5	0.0554	0	-0.24888099
PTEN	SDK1	0.0554	0	-0.194389473
PTEN	IL23R	0.0554	0	-0.31425671
PTEN	PPP1R13B	0.0554	0	-0.03027188
PTEN	OR2C3	0.0555	0	-0.137782412
PTEN	DSCR3	0.0555	0	-0.04721019
PTEN	TTTY3B	0.0555	0	-0.024568935
PTEN	CEACAM1	0.0555	0	0.150023018
PTEN	SLC31A1	0.0555	0	-0.038963875
PTEN	KRT28	0.0556	0	-0.04296396
PTEN	CD99	0.0556	0	0.170551559
PTEN	KIAA0892	0.0556	0	-0.004728438

PTEN	LMAN2L	0.0557	0	0.000555652
PTEN	LOC254559	0.0557	0	-0.009975725
PTEN	ST6GALNAC6	0.0557	0	-0.002700535
PTEN	TIGD7	0.0557	0	-0.01249011
PTEN	FLJ22536	0.0558	0	-0.071936046
PTEN	ISOC1	0.0558	0	-0.400047928
PTEN	HAPLN4	0.0558	0	-0.176733195
PTEN	GCLM	0.0558	0	-0.364060741
PTEN	BDNFOS	0.0558	0	-0.425414436
PTEN	UCA1	0.0559	0	-0.107538058
PTEN	ELAVL2	0.056	0	-0.453011719
PTEN	RGS8	0.056	0	-0.009572421
PTEN	LOC645166	0.056	0	-0.231605929
PTEN	EBF4	0.056	0	-0.128849286
PTEN	SCARA5	0.056	0	-0.056873995
PTEN	RDH11	0.056	0	0.000530984
PTEN	KLB	0.056	0	-0.209697353
PTEN	GAS2	0.056	0	-0.238289431
PTEN	LEFTY2	0.0561	0	-0.14316714
PTEN	SMARCA1	0.0561	0	-0.536167882
PTEN	14-Sep	0.0561	0	-0.221907161
PTEN	TMPRSS11D	0.0561	0	-0.244766575
PTEN	CARD10	0.0561	0	-0.001503099
PTEN	B3GALNT1	0.0561	0	-0.262512937
PTEN	CSN1S1	0.0561	0	0.159578762
PTEN	TMEM63A	0.0561	0	-4.33E-06
PTEN	C17orf39	0.0561	0	-0.372297817
PTEN	KY	0.0561	0	-0.018144525
PTEN	TFAP2A	0.0562	0	0.277999797
PTEN	TPTE2P1	0.0562	0	-0.198937573
PTEN	APOBEC3F	0.0562	0	-0.013061626
PTEN	SNORD114-31	0.0563	0	-0.000452248
PTEN	WFDC11	0.0563	0	-0.010983209
PTEN	FAM71C	0.0563	0	-0.144583721
PTEN	CREB3L2	0.0563	0	-0.062935041
PTEN	WNT8B	0.0564	0	-0.001576649
PTEN	SMYD4	0.0564	0	-0.053718887
PTEN	DIO1	0.0564	0	-0.00570098
PTEN	MARK1	0.0564	0	-0.216964527
PTEN	ELSPBP1	0.0564	0	-0.007567123
PTEN	GDF3	0.0565	0	-0.006310967

PTEN	NIPSNAP3B	0.0565	0	-0.226711281
PTEN	LOC644538	0.0565	0	-0.437981997
PTEN	SART3	0.0566	0	0.014526235
PTEN	ANXA1	0.0566	0	0.064241863
PTEN	GLIPR1L1	0.0566	0	-0.209929895
PTEN	ADAMTSL4	0.0566	0	-0.142776122
PTEN	GDF11	0.0567	0	-0.473520046
PTEN	ZNF266	0.0568	0	-0.111950556
PTEN	LEAP2	0.0569	0	-0.051996098
PTEN	PRIMA1	0.0569	0	-0.136766112
PTEN	SNORD1B	0.0569	0	-0.169300308
PTEN	PRSS12	0.0569	0	-0.009341353
PTEN	TP53AIP1	0.057	0	-0.251011036
PTEN	FAM105B	0.057	0	-0.038311538
PTEN	SPDYE6	0.057	0	-0.041200333
PTEN	BRDT	0.057	0	-0.215330693
PTEN	CD180	0.057	0	-0.001646513
PTEN	ARMC4	0.057	0	-0.009860657
PTEN	ADCY8	0.057	0	-0.020783686
PTEN	SASS6	0.0571	0	-0.294249531
PTEN	HSPB2	0.0572	0	-9.71E-06
PTEN	NLK	0.0572	0	-0.225493639
PTEN	FOLR2	0.0572	0	-0.000398565
PTEN	KAZ	0.0572	0	-0.200466385
PTEN	ARHGDIB	0.0573	0	-0.002013888
PTEN	PLIN4	0.0573	0	-2.87E-05
PTEN	KRTAP12-1	0.0573	0	-0.002005237
PTEN	DMD	0.0573	0	-0.142906377
PTEN	RPL9	0.0573	0	0.030273658
PTEN	EPS8L3	0.0573	0	-0.17059652
PTEN	LOC100130932	0.0574	0	-0.128681117
PTEN	MED12	0.0574	0	-0.043065354
PTEN	DNAJC28	0.0574	0	-0.185847535
PTEN	C9orf122	0.0575	0	-0.203340349
PTEN	C20orf196	0.0575	0	-0.00018451
PTEN	MRPL30	0.0575	0	-0.053987504
PTEN	SATB1	0.0575	0	-0.141341956
PTEN	NUDT13	0.0575	0	0.067733473
PTEN	GAL3ST3	0.0575	0	-0.176682528
PTEN	YOD1	0.0576	0	-0.429967026
PTEN	NRIP2	0.0576	0	-0.001496553

PTEN	PLA2G2A	0.0577	0	-0.000144009
PTEN	KIAA0196	0.0577	0	0.01924751
PTEN	CCDC136	0.0577	0	-0.008829569
PTEN	ZNF805	0.0577	0	-0.004442816
PTEN	MAGEA8	0.0577	0	-0.140454018
PTEN	SCN11A	0.0579	0	-0.025509364
PTEN	MYOZ2	0.0579	0	-0.352364696
PTEN	C3orf15	0.0579	0	-0.250823012
PTEN	NPY6R	0.0579	0	-0.056097307
PTEN	METTL7A	0.058	0	-0.009166421
PTEN	RHOU	0.058	0	-0.253062771
PTEN	SLC26A4	0.058	0	-0.34193843
PTEN	DDI1	0.058	0	-0.202109945
PTEN	CSRP1	0.058	0	-0.093583306
PTEN	LEP	0.058	0	0.086496051
PTEN	ABR	0.0581	0	-0.139258195
PTEN	CD1C	0.0581	0	-0.034502957
PTEN	COLQ	0.0581	0	-0.020562495
PTEN	KRTAP3-1	0.0581	0	-0.008412892
PTEN	LOC440040	0.0582	0	-0.037809415
PTEN	MALAT1	0.0582	0	3.55E-06
PTEN	TPSG1	0.0582	0	-3.47E-05
PTEN	FGB	0.0582	0	-0.045857127
PTEN	DAD1	0.0583	0	0.016826125
PTEN	C20orf30	0.0583	0	0.003314434
PTEN	IGSF1	0.0583	0	-0.338589102
PTEN	PROC	0.0583	0	-0.004185744
PTEN	EIF3M	0.0583	0	0.000381565
PTEN	SMPD3	0.0583	0	-0.12185835
PTEN	TMEM100	0.0584	0	-0.398739993
PTEN	PON3	0.0584	0	-0.295025039
PTEN	LARS2	0.0584	0	-0.039626369
PTEN	RIMKLB	0.0584	0	-0.307575726
PTEN	EPHA6	0.0584	0	-0.159309344
PTEN	SNORD113-2	0.0584	0	-0.00043867
PTEN	RABL2B	0.0585	0	-0.000858325
PTEN	LY6G6C	0.0585	0	-0.172873299
PTEN	GPR137C	0.0586	0	-0.367055614
PTEN	CCDC151	0.0586	0	-0.001113923
PTEN	MYO16	0.0586	0	-0.332214516
PTEN	PODXL	0.0587	0	-0.121526039

PTEN	OBFC2A	0.0587	0	-0.334212209
PTEN	PAG1	0.0587	0	-0.173234253
PTEN	GCN1L1	0.0587	0	-0.109539852
PTEN	EMP2	0.0588	0	0.198669776
PTEN	CASP1	0.0588	0	-0.150210802
PTEN	SPATA20	0.0588	0	0.000243391
PTEN	GJA4	0.0589	0	-0.00832665
PTEN	EMX1	0.0589	0	-0.001682588
PTEN	CHRNB2	0.0589	0	-0.021315111
PTEN	SEC63	0.0589	0	-0.024966273
PTEN	TMEM132E	0.059	0	-0.151974469
PTEN	MAPKAP1	0.059	0	0.107675132
PTEN	FAM86B2	0.0591	0	-0.179620751
PTEN	SP2	0.0591	0	0.007204268
PTEN	GVIN1	0.0591	0	-0.57665554
PTEN	PRMT3	0.0591	0	-0.109354031
PTEN	LAMC3	0.0591	0	-0.094293621
PTEN	THAP9	0.0592	0	-0.069636824
PTEN	ZBTB49	0.0592	0	-0.067822787
PTEN	KCNIP1	0.0592	0	-0.308409689
PTEN	SERPINB11	0.0592	0	-0.013625973
PTEN	RMND1	0.0593	0	0.013162292
PTEN	SETD1B	0.0593	0	0.12527097
PTEN	ZNF404	0.0593	0	-0.014922633
PTEN	STRBP	0.0593	0	0.005874323
PTEN	RPLP0P2	0.0593	0	-0.01585759
PTEN	HERC6	0.0593	0	-0.023067043
PTEN	ZNF607	0.0593	0	-0.293699515
PTEN	HCG11	0.0593	0	-0.019751978
PTEN	STAT5B	0.0594	0	0.008740735
PTEN	CLVS2	0.0594	0	-0.001953297
PTEN	PRAMEF2	0.0595	0	-0.041622652
PTEN	C4orf37	0.0595	0	-0.002032053
PTEN	SNPH	0.0595	0	-0.160761666
PTEN	TMEFF2	0.0596	0	-0.186242363
PTEN	B4GALNT3	0.0596	0	0.00025241
PTEN	ABCE1	0.0596	0	-0.032916427
PTEN	FCGR3A	0.0596	0	0.005965564
PTEN	ADAMTS3	0.0597	0	-0.434215309
PTEN	TRIB1	0.0597	0	0.08428217
PTEN	BGN	0.0597	0	0.004697417

PTEN	SLC48A1	0.0598	0	-0.160550116
PTEN	FLNA	0.0598	0	0.001503246
PTEN	MXD1	0.0598	0	-0.493635639
PTEN	CHST1	0.0599	0	0.004229341
PTEN	PRAMEF13	0.0599	0	-0.382311201
PTEN	EPS15L1	0.0599	0	-0.00109484
PTEN	HADH	0.0599	0	-0.022797722
PTEN	ACIN1	0.0599	0	-0.063179857
PTEN	ROBO4	0.06	0	-0.001947878
PTEN	KIAA1274	0.06	0	-0.247752653
PTEN	CD274	0.0601	0	-0.337606414
PTEN	BEAN	0.0601	0	0.070325348
PTEN	ZNF747	0.0601	0	-0.119121186
PTEN	ZNF665	0.0601	0	-0.261091824
PTEN	SNTG1	0.0602	0	-0.003647178
PTEN	MYT1	0.0602	0	-0.334984581
PTEN	CYYR1	0.0603	0	-0.15197536
PTEN	SIRT3	0.0603	0	-0.211153531
PTEN	PLGLB2	0.0603	0	-0.45932305
PTEN	RGPD1	0.0603	0	-0.245584226
PTEN	OXA1L	0.0603	0	0.00033406
PTEN	PRSS38	0.0604	0	-0.000568643
PTEN	RTN2	0.0605	0	-0.00188701
PTEN	PNLIPRP3	0.0605	0	-0.371374235
PTEN	CEP76	0.0605	0	-0.053646882
PTEN	MGC42105	0.0605	0	-0.001177902
PTEN	IGFBPL1	0.0607	0	-0.008103023
PTEN	CDH12	0.0607	0	-0.017500762
PTEN	MAP1LC3C	0.0608	0	-0.229276832
PTEN	C20orf85	0.0608	0	-0.004826902
PTEN	POLR1A	0.0608	0	-0.004583453
PTEN	PEX11B	0.0608	0	-0.000982708
PTEN	SESN2	0.0608	0	-0.227981444
PTEN	TNFSF18	0.0609	0	-0.001241387
PTEN	CCDC97	0.0609	0	0.171030982
PTEN	MLXIP	0.061	0	0.150785376
PTEN	SCAMP2	0.0611	0	0.011032626
PTEN	SERPINB2	0.0611	0	-0.01143881
PTEN	FMR1	0.0611	0	-0.111623041
PTEN	MAK16	0.0611	0	-0.218533027
PTEN	LLPH	0.0611	0	-0.095038466

PTEN	NIPAL2	0.0611	0	-0.287476797
PTEN	C1orf227	0.0611	0	-0.009260912
PTEN	CMTM4	0.0611	0	-0.044027849
PTEN	SCGB1A1	0.0611	0	-0.001218664
PTEN	DAB1	0.0612	0	-0.010829991
PTEN	XIRP2	0.0613	0	-0.357170163
PTEN	OTC	0.0614	0	-0.32102424
PTEN	PCDHA1	0.0614	0	-0.386735402
PTEN	PCGF3	0.0614	0	0.040849834
PTEN	LOC647309	0.0614	0	-0.245758104
PTEN	SERPINA10	0.0614	0	-0.320793199
PTEN	CAPN5	0.0615	0	0.127799068
PTEN	HLA-DOA	0.0615	0	0.014152568
PTEN	CPPED1	0.0616	0	-0.215209969
PTEN	RUSC1	0.0615	0	0.000201102
PTEN	IGFBP6	0.0616	0	-7.20E-05
PTEN	POGK	0.0616	0	0.030229456
PTEN	CREBZF	0.0616	0	-0.048804041
PTEN	GRIA2	0.0616	0	0.126863368
PTEN	SCRN1	0.0616	0	-0.118171092
PTEN	DZIP1	0.0616	0	-0.226483789
PTEN	HIST2H2BF	0.0617	0	0.14712585
PTEN	C10orf11	0.0617	0	-0.018577162
PTEN	BRCA1	0.0617	0	-0.03648899
PTEN	CD28	0.0618	0	-0.04951055
PTEN	SLC46A2	0.0618	0	-0.28587779
PTEN	C14orf4	0.0618	0	-0.370066934
PTEN	C13orf39	0.0618	0	-0.121072783
PTEN	FIBCD1	0.0618	0	-0.146340243
PTEN	DARC	0.0619	0	0.002015544
PTEN	POLD3	0.0619	0	0.005257727
PTEN	C9orf3	0.0619	0	-0.175855548
PTEN	CCNJL	0.062	0	-0.185385972
PTEN	ACAD11	0.062	0	-0.280811079
PTEN	PABPC1L2B	0.0621	0	-0.594362518
PTEN	CDC20B	0.0621	0	-0.012603401
PTEN	C21orf94	0.0621	0	-0.233876464
PTEN	LEPRE1	0.0622	0	-0.00177593
PTEN	GPR68	0.0622	0	-0.068897293
PTEN	ZNF233	0.0622	0	0.083032715
PTEN	MKNK1	0.0622	0	-0.079167076

PTEN	NLRP1	0.0622	0	-0.201876658
PTEN	INHBB	0.0623	0	-0.309266102
PTEN	GPR22	0.0623	0	-0.259634714
PTEN	STK16	0.0623	0	-0.045050934
PTEN	LECT2	0.0624	0	-0.306194797
PTEN	ZNF629	0.0624	0	-0.089185355
PTEN	TCP10	0.0625	0	-0.000113414
PTEN	LYPLAL1	0.0625	0	0.023179003
PTEN	TEKT1	0.0625	0	-0.000593721
PTEN	SNORD115-14	0.0625	0	-1.20E-07
PTEN	C21orf7	0.0626	0	-0.19325689
PTEN	HOXB9	0.0626	0	-0.02538054
PTEN	OMA1	0.0626	0	-0.228825623
PTEN	FAM198A	0.0626	0	-0.25351021
PTEN	TEKT2	0.0627	0	-0.011027676
PTEN	SIKE1	0.0627	0	-0.325719241
PTEN	CD22	0.0627	0	-0.00120684
PTEN	INPP1	0.0627	0	-0.012123682
PTEN	DHH	0.0627	0	-0.001022201
PTEN	PEX13	0.0628	0	0.215889564
PTEN	FAM13AOS	0.0629	0	-0.243117196
PTEN	DES	0.0629	0	-0.000753304
PTEN	FAM168B	0.0629	0	-0.009548871
PTEN	SIGLEC5	0.0629	0	-0.00546906
PTEN	TRA2A	0.0629	0	-0.002364853
PTEN	BCAM	0.0629	0	0.007156422
PTEN	CBFA2T3	0.063	0	-0.174528165
PTEN	GSG1L	0.063	0	-0.003148279
PTEN	GOLT1B	0.0631	0	-0.152670166
PTEN	PPA1	0.0631	0	-0.090784479
PTEN	VEPH1	0.0631	0	-0.007078513
PTEN	DIAPH2	0.0631	0	-0.208218763
PTEN	KDM2A	0.0631	0	0.149247383
PTEN	SNORD116-23	0.0632	0	-8.98E-08
PTEN	ZNF425	0.0632	0	-0.197537283
PTEN	ODZ1	0.0632	0	-0.494626762
PTEN	ZNF443	0.0633	0	-0.14340443
PTEN	RPL23	0.0633	0	0.184460967
PTEN	SCGN	0.0633	0	-0.177138566
PTEN	PCGF2	0.0634	0	-0.004706796
PTEN	TRIM52	0.0634	0	-0.19817554

PTEN	ATOH8	0.0634	0	-0.006579777
PTEN	EPB42	0.0634	0	-3.57E-05
PTEN	TMEM218	0.0635	0	-0.00856825
PTEN	ARG1	0.0635	0	-0.03720519
PTEN	VTI1B	0.0635	0	0.013037874
PTEN	HDAC7	0.0635	0	-0.046894886
PTEN	10-Mar	0.0636	0	-0.171530644
PTEN	MBL2	0.0636	0	-0.46560195
PTEN	C17orf80	0.0637	0	-0.423016881
PTEN	ALDH3B2	0.0637	0	0.069156868
PTEN	CERK	0.0637	0	-0.240636217
PTEN	DEPDC7	0.0637	0	-0.023491629
PTEN	GPD1	0.0637	0	-0.231618567
PTEN	HIC1	0.0638	0	-0.027583354
PTEN	PLA2G4A	0.0638	0	-0.015279546
PTEN	RXFP1	0.0638	0	-9.57E-05
PTEN	TGFB1	0.0638	0	-0.000589451
PTEN	CFLAR	0.0639	0	0.00665328
PTEN	MPPED1	0.0639	0	-0.077563025
PTEN	CEP57	0.064	0	-0.003569017
PTEN	NR2C1	0.0641	0	-0.109548957
PTEN	ZCCHC12	0.064	0	-0.016966629
PTEN	C14orf142	0.0641	0	-0.164830465
PTEN	C12orf70	0.0641	0	-0.00085558
PTEN	INPP5D	0.0641	0	-0.173575739
PTEN	C1orf114	0.0642	0	-0.151158704
PTEN	KLHL10	0.0642	0	-0.005030345
PTEN	NR2E3	0.0642	0	-0.607036573
PTEN	C6orf154	0.0642	0	-0.136848014
PTEN	UBXN2A	0.0642	0	-0.263526641
PTEN	RABGEF1	0.0643	0	-0.069475535
PTEN	CST1	0.0643	0	1.00E-07
PTEN	HS6ST3	0.0643	0	-0.181791743
PTEN	TUBGCP3	0.0643	0	-0.091368832
PTEN	DDX42	0.0644	0	-0.132640854
PTEN	TMEM156	0.0644	0	-0.011363868
PTEN	CHRM5	0.0644	0	-0.4147685
PTEN	NSUN6	0.0644	0	-0.173229672
PTEN	C12orf49	0.0644	0	-0.262269177
PTEN	CCDC33	0.0644	0	-0.004900166
PTEN	PEX7	0.0644	0	-0.012464553

PTEN	SV2A	0.0644	0	-0.326029791
PTEN	ZNF853	0.0645	0	-0.206958927
PTEN	ZDHHC5	0.0645	0	0.098772248
PTEN	TSPAN8	0.0646	0	0.109955837
PTEN	ZMAT2	0.0646	0	0.049502283
PTEN	TAX1BP1	0.0647	0	0.093309373
PTEN	R3HDM1	0.0647	0	0.002809775
PTEN	CCDC108	0.0647	0	-0.062010344
PTEN	CCDC11	0.0647	0	-0.000186031
PTEN	ACSM2A	0.0648	0	-0.035031446
PTEN	DPY19L4	0.0647	0	0.093745965
PTEN	FLJ36000	0.0648	0	-0.128029534
PTEN	C11orf1	0.0648	0	-0.00032072
PTEN	SLC35F1	0.0648	0	-0.236807102
PTEN	ADRB1	0.0649	0	-0.238643746
PTEN	INTS4	0.0649	0	0.005590598
PTEN	ST6GALNAC1	0.0649	0	-0.003409456
PTEN	USP49	0.065	0	-0.065752269
PTEN	RFTN1	0.0651	0	-0.275704375
PTEN	CINP	0.0651	0	-0.056102118
PTEN	ADAM19	0.0651	0	0.135494493
PTEN	CUBN	0.0651	0	-0.214965372
PTEN	S100A14	0.0651	0	0.00759684
PTEN	C14orf39	0.0651	0	-0.441915861
PTEN	HMP19	0.0652	0	-0.012510479
PTEN	PHF21B	0.0652	0	-0.33306936
PTEN	TXNDC3	0.0652	0	-0.004186212
PTEN	FAM176B	0.0652	0	7.17E-07
PTEN	RAB17	0.0652	0	-0.001056118
PTEN	RELL1	0.0653	0	-0.15315628
PTEN	CLEC4GP1	0.0654	0	0.000237006
PTEN	VPS33A	0.0654	0	-0.039720445
PTEN	ZNF331	0.0654	0	-0.11499932
PTEN	IFNA21	0.0654	0	-0.179720988
PTEN	CXXC5	0.0654	0	-0.002511201
PTEN	PNPLA2	0.0655	0	0.04363756
PTEN	ARAF	0.0655	0	-0.152059237
PTEN	LCN6	0.0655	0	-0.001465651
PTEN	CYP3A7	0.0656	0	-0.022393413
PTEN	HAUS4	0.0656	0	0.00176483
PTEN	C6orf10	0.0656	0	-0.010425344

PTEN	NDST3	0.0656	0	-0.585069278
PTEN	SPINK13	0.0656	0	-0.043031734
PTEN	CACNB2	0.0656	0	-0.332732128
PTEN	ZNF512	0.0657	0	-0.103928196
PTEN	GPR153	0.0657	0	-0.05840324
PTEN	C18orf45	0.0657	0	-0.157607178
PTEN	TTC21A	0.0657	0	-0.000111232
PTEN	TMEM159	0.0659	0	-0.018124877
PTEN	ZNF496	0.0659	0	-6.60E-06
PTEN	HMGCL	0.0659	0	-0.003344892
PTEN	SNORD115-6	0.0659	0	-0.000873579
PTEN	CRADD	0.0659	0	-0.212759116
PTEN	CCR6	0.0659	0	-0.021496953
PTEN	ESM1	0.066	0	-0.178354444
PTEN	OSTC	0.0661	0	-0.002224627
PTEN	OXR1	0.0661	0	0.040102404
PTEN	GJB1	0.0661	0	-0.007920356
PTEN	TMEFF1	0.0661	0	0.117299442
PTEN	CLDN19	0.0662	0	-0.060037418
PTEN	CLEC1B	0.0662	0	-0.003854636
PTEN	ISL1	0.0662	0	-0.454981965
PTEN	PTPN12	0.0663	0	-0.214357752
PTEN	THADA	0.0664	0	-4.96E-05
PTEN	PYCARD	0.0664	0	0.035678109
PTEN	BAG1	0.0664	0	0.150742567
PTEN	TRAPPC10	0.0664	0	-0.345542488
PTEN	KBTBD6	0.0664	0	0.050203818
PTEN	FSCB	0.0665	0	-0.172115996
PTEN	FLNB	0.0665	0	0.011316121
PTEN	BICD2	0.0666	0	-0.261162276
PTEN	APOD	0.0666	0	0.00010287
PTEN	ITGB4	0.0666	0	-0.010070405
PTEN	CA14	0.0666	0	-0.015813735
PTEN	SMARCAL1	0.0666	0	-0.020206347
PTEN	BNC1	0.0666	0	-0.164096269
PTEN	MRI1	0.0666	0	0.086489457
PTEN	DPCR1	0.0667	0	-0.165969969
PTEN	HBEGF	0.0667	0	-0.191810969
PTEN	ACSM2B	0.0668	0	-0.128736007
PTEN	IQSEC1	0.0669	0	0.02619657
PTEN	MAP4K4	0.067	0	-0.049299806

PTEN	ZNF880	0.0671	0	0.000814992
PTEN	ANKRD24	0.0671	0	-0.002031066
PTEN	YSK4	0.0671	0	-0.181647527
PTEN	RAB40A	0.0672	0	-0.000149802
PTEN	TRPM4	0.0672	0	-0.000610361
PTEN	C4orf42	0.0672	0	-0.017052584
PTEN	C1orf70	0.0672	0	-0.001408622
PTEN	STARD5	0.0673	0	-0.157286317
PTEN	HMGXB3	0.0673	0	-0.008711392
PTEN	NKAIN1	0.0673	0	0.14907371
PTEN	KCNE3	0.0673	0	-0.494369123
PTEN	NCKAP1L	0.0673	0	-0.00134708
PTEN	LOC100130987	0.0673	0	-0.000764729
PTEN	IFT122	0.0674	0	-1.01E-06
PTEN	DLK1	0.0674	0	-0.003837723
PTEN	VLDLR	0.0675	0	-0.181351588
PTEN	DKFZP586I1420	0.0675	0	-0.298572118
PTEN	CHRNA1	0.0675	0	-0.025416359
PTEN	C2orf54	0.0676	0	-0.012585811
PTEN	SKI	0.0676	0	-0.179133465
PTEN	MTUS2	0.0676	0	-0.11652704
PTEN	RNF4	0.0676	0	0.025078709
PTEN	ZNF142	0.0677	0	-0.288384452
PTEN	TFDP2	0.0677	0	-0.00033049
PTEN	KDELR3	0.0678	0	0.007291227
PTEN	C17orf107	0.0678	0	-0.011531725
PTEN	MAOB	0.0678	0	0.000474756
PTEN	EVI2B	0.0678	0	-0.14229145
PTEN	KLHL14	0.0678	0	-0.398604245
PTEN	CALM1	0.0679	0	0.167250651
PTEN	LRRC37A2	0.0679	0	-0.000112029
PTEN	TLK2	0.0679	0	-0.135535008
PTEN	ARL8B	0.0679	0	0.33316408
PTEN	CRYGC	0.068	0	-0.000858417
PTEN	C9orf110	0.068	0	-0.008521523
PTEN	GPX2	0.0681	0	-0.000120304
PTEN	C18orf32	0.0681	0	-0.053954066
PTEN	C13orf1	0.0683	0	-0.402767584
PTEN	FBXL13	0.0684	0	-0.200385273
PTEN	KRT71	0.0684	0	-0.050899878
PTEN	XKR5	0.0684	0	-0.169543952

PTEN	RBP5	0.0684	0	-0.009844381
PTEN	RASSF5	0.0684	0	-0.167834254
PTEN	BSN	0.0685	0	-0.459729471
PTEN	DHDDS	0.0685	0	-0.136497278
PTEN	RRP15	0.0685	0	-0.189459181
PTEN	NTS	0.0685	0	-0.487453427
PTEN	TSLP	0.0685	0	-0.497324795
PTEN	MAP1D	0.0685	0	-0.116485943
PTEN	TTC39B	0.0685	0	-0.154927808
PTEN	ADHFE1	0.0685	0	-0.184689663
PTEN	DEFB123	0.0687	0	-0.000442521
PTEN	MOBKL2B	0.0687	0	-0.010945734
PTEN	WFDC6	0.0688	0	-0.006973149
PTEN	SPTA1	0.0688	0	-0.382485225
PTEN	C17orf58	0.0688	0	-0.089880447
PTEN	ADAMTS9	0.0688	0	-0.102142724
PTEN	FBN2	0.0688	0	-0.281539851
PTEN	SMARCE1	0.0688	0	0.049475168
PTEN	HEPN1	0.0688	0	-0.129295535
PTEN	PRR13	0.0688	0	0.008778932
PTEN	HSDL1	0.0689	0	-0.108255957
PTEN	ZNF70	0.0689	0	-0.01201564
PTEN	SLC5A9	0.0689	0	-0.033704965
PTEN	ZNF438	0.0689	0	-0.027100952
PTEN	HIATL1	0.0689	0	-0.638569997
PTEN	MTF2	0.0689	0	-0.426358066
PTEN	UPF2	0.069	0	0.089936983
PTEN	GDAP1	0.069	0	-0.147893726
PTEN	SRRD	0.0691	0	-0.004550928
PTEN	TMEM194A	0.0691	0	-0.218926768
PTEN	RDX	0.0691	0	-0.017445768
PTEN	CDH20	0.0691	0	-0.17112292
PTEN	RLN2	0.0691	0	-0.092664391
PTEN	TCP10L	0.0691	0	-0.00194819
PTEN	ZNF687	0.0692	0	-0.001660533
PTEN	C5orf23	0.0692	0	-0.385592274
PTEN	GIF	0.0692	0	-0.004450745
PTEN	A2BP1	0.0692	0	-0.607509792
PTEN	ASCL4	0.0692	0	-0.300450968
PTEN	ADPRHL1	0.0692	0	0.121448269
PTEN	RMND5A	0.0693	0	0.183088898

PTEN	TPM3	0.0693	0	0.351076868
PTEN	GNAT2	0.0693	0	-0.164494697
PTEN	ZNF599	0.0694	0	-0.157980201
PTEN	CDKL2	0.0694	0	-0.357982324
PTEN	PHF10	0.0694	0	-0.012741942
PTEN	ENTPD8	0.0694	0	1.38E-05
PTEN	DHX57	0.0694	0	-0.147592578
PTEN	CCL22	0.0695	0	-0.163424074
PTEN	ANO2	0.0696	0	-0.259343706
PTEN	CRYBB1	0.0695	0	-6.16E-05
PTEN	CAMKK2	0.0696	0	0.16288927
PTEN	PLB1	0.0696	0	-0.015506076
PTEN	SPIRE1	0.0696	0	-0.084124827
PTEN	LASS5	0.0697	0	-0.013682083
PTEN	C1orf216	0.0697	0	-0.134250474
PTEN	C12orf43	0.0697	0	-0.012247136
PTEN	PVRL2	0.0697	0	-0.14918015
PTEN	SGCE	0.0697	0	0.000364415
PTEN	NTRK3	0.0697	0	-0.13406632
PTEN	ENO2	0.0697	0	-0.013861613
PTEN	CCDC117	0.0697	0	0.020499513
PTEN	TFRC	0.0698	0	0.145232659
PTEN	RHAG	0.0697	0	-0.200626223
PTEN	SMC6	0.0698	0	-0.102186921
PTEN	PIK3AP1	0.0699	0	-0.282197812
PTEN	TMEM173	0.0699	0	-0.000231772
PTEN	PAQR3	0.0699	0	-0.098615792
PTEN	LMO3	0.0699	0	-0.372225142
PTEN	PHF16	0.0699	0	-0.01362517
PTEN	ZNF500	0.0699	0	-0.256855575
PTEN	EXOSC1	0.0699	0	0.013908388
PTEN	TRAFD1	0.0699	0	-0.119411271
PTEN	CADM3	0.07	0	-0.017628703
PTEN	ARPC2	0.07	0	0.076284662
PTEN	C1QTNF2	0.07	0	-0.288698633
PTEN	GALK2	0.07	0	-0.173535915
PTEN	C5orf49	0.07	0	-0.151490499
PTEN	RBM20	0.07	0	-0.058870089
PTEN	ARHGEF11	0.0701	0	-0.009158675
PTEN	DEFB122	0.0701	0	-0.024104422
PTEN	AIM1	0.0702	0	-0.015808907

PTEN	MOV10L1	0.0702	0	-0.006896
PTEN	RPL26	0.0702	0	0.007020609
PTEN	VSIG4	0.0702	0	-0.011269473
PTEN	GTDC1	0.0702	0	-0.264347855
PTEN	KBTBD8	0.0703	0	-0.575174142
PTEN	CCDC144NL	0.0703	0	-0.563041247
PTEN	PXMP4	0.0703	0	-0.030558408
PTEN	ACOT2	0.0703	0	0.010058911
PTEN	LOC284551	0.0703	0	-0.387571884
PTEN	CLEC11A	0.0703	0	-0.117575872
PTEN	PRSS21	0.0703	0	3.33E-07
PTEN	CHRFAM7A	0.0703	0	-0.363975398
PTEN	GLI1	0.0703	0	-0.130369767
PTEN	GPR82	0.0703	0	-0.019857134
PTEN	NCRNA00181	0.0704	0	-0.191636364
PTEN	SLC27A1	0.0704	0	-0.017981933
PTEN	ZCRB1	0.0704	0	0.15788951
PTEN	ASCL3	0.0704	0	-0.000214023
PTEN	KIAA0649	0.0705	0	-0.047195307
PTEN	SHISA4	0.0706	0	-7.11E-05
PTEN	RLN1	0.0706	0	-0.227951615
PTEN	H2AFV	0.0706	0	0.220766454
PTEN	MPPE1	0.0707	0	-0.214751963
PTEN	C6orf35	0.0707	0	-0.233819972
PTEN	TREH	0.0708	0	-0.009792962
PTEN	CPLX1	0.0708	0	-0.009122208
PTEN	ZNF642	0.0708	0	-0.031161034
PTEN	RPL22	0.0708	0	0.021828544
PTEN	IFNW1	0.0709	0	-0.042404704
PTEN	SLC38A5	0.0709	0	-0.004482089
PTEN	SUDS3	0.0709	0	0.144148062
PTEN	KLHL23	0.0709	0	-0.137936059
PTEN	MTMR1	0.071	0	0.073501313
PTEN	PDCD10	0.071	0	-0.024787451
PTEN	DSCAM	0.071	0	-0.423503496
PTEN	FSD2	0.071	0	-1.13E-07
PTEN	FAM84B	0.071	0	0.152443087
PTEN	ACVRL1	0.0711	0	-0.198721829
PTEN	SUPT3H	0.0711	0	0.119720013
PTEN	PGA3	0.0711	0	-0.168557466
PTEN	HEPACAM	0.0711	0	-0.015811242

PTEN	FLJ10038	0.0712	0	-0.227285104
PTEN	CAV3	0.0712	0	-0.153972352
PTEN	GDEP	0.0713	0	-0.309975157
PTEN	FAM83E	0.0712	0	-0.000173974
PTEN	C4orf10	0.0713	0	-0.127474398
PTEN	KANK1	0.0713	0	-0.103498547
PTEN	BMP8B	0.0713	0	-0.186674147
PTEN	NOS1	0.0713	0	-0.041850761
PTEN	CDKN1A	0.0713	0	0.049210736
PTEN	KIAA0495	0.0714	0	-0.033371189
PTEN	ATP5B	0.0714	0	0.020241636
PTEN	BMP8A	0.0714	0	-0.281088551
PTEN	CUL9	0.0715	0	-1.12E-06
PTEN	ZNF35	0.0715	0	-0.333487106
PTEN	IL19	0.0715	0	-0.00213919
PTEN	CLUAP1	0.0715	0	0.157175247
PTEN	C7orf46	0.0715	0	-0.304755066
PTEN	FAM138E	0.0715	0	-0.120080408
PTEN	FRMD1	0.0715	0	-0.002326059
PTEN	RIT2	0.0715	0	-0.162707481
PTEN	RALA	0.0716	0	0.051885656
PTEN	XPNPEP2	0.0717	0	-0.136617861
PTEN	SPATA22	0.0717	0	-0.029471828
PTEN	ATP11B	0.0718	0	0.326263274
PTEN	ANKRD10	0.0718	0	-0.10768149
PTEN	SYNGAP1	0.0718	0	-0.034677425
PTEN	FGF13	0.0718	0	-0.354451937
PTEN	KRT37	0.0719	0	-0.13531892
PTEN	TCEAL6	0.0719	0	-0.196915654
PTEN	DLGAP2	0.0719	0	-0.245683705
PTEN	TPSB2	0.0719	0	-0.001481927
PTEN	CUTC	0.0719	0	-0.157049342
PTEN	NKIRAS1	0.072	0	-0.161649522
PTEN	KCNA4	0.072	0	-0.341922203
PTEN	FABP4	0.072	0	-0.148329061
PTEN	AKAP7	0.0721	0	-0.488471547
PTEN	PABPC1L2A	0.0721	0	-0.245048501
PTEN	PPARG	0.0721	0	-0.136190699
PTEN	ZNF469	0.0721	0	-0.069758812
PTEN	TRIM72	0.0722	0	-0.167932969
PTEN	PLCZ1	0.0723	0	-0.000319687

PTEN	RWDD4A	0.0723	0	0.148197472
PTEN	CPA1	0.0723	0	-0.000138192
PTEN	FLJ33360	0.0723	0	-0.258139853
PTEN	GPX3	0.0724	0	0.002435856
PTEN	DNAJC15	0.0724	0	-0.17523992
PTEN	C10orf75	0.0724	0	-0.012612006
PTEN	FBXW7	0.0724	0	-0.318237301
PTEN	CSTT	0.0725	0	-0.024278842
PTEN	C17orf51	0.0725	0	-0.005722444
PTEN	LRRC36	0.0725	0	-0.000327885
PTEN	AMDHD1	0.0726	0	-0.011905199
PTEN	NBL1	0.0726	0	-0.077746874
PTEN	RAD1	0.0727	0	-0.020465997
PTEN	WDR75	0.0727	0	-0.000458084
PTEN	PCMTD2	0.0727	0	0.147268714
PTEN	IDH1	0.0727	0	0.191592381
PTEN	EPM2A	0.0728	0	-0.516121209
PTEN	NPAS4	0.0728	0	-0.174259051
PTEN	GTF3C1	0.0728	0	-0.097513669
PTEN	ALMS1P	0.0729	0	-0.030395943
PTEN	PRTFDC1	0.0729	0	-0.157986719
PTEN	OR6A2	0.073	0	-0.172728672
PTEN	KIF21A	0.073	0	-0.340942661
PTEN	CHN1	0.073	0	-0.092706373
PTEN	GRIN1	0.073	0	-8.52E-05
PTEN	NRN1	0.073	0	-0.030804972
PTEN	HEY2	0.0731	0	-0.6199063
PTEN	LOC642597	0.0731	0	-0.235392948
PTEN	LOC126536	0.0731	0	-0.326714033
PTEN	FAM177A1	0.0731	0	-0.112356483
PTEN	SNTN	0.0732	0	-0.605902577
PTEN	CAPN11	0.0732	0	-0.001040619
PTEN	LRRC37A	0.0732	0	-0.000112561
PTEN	SLC1A3	0.0732	0	-0.290847389
PTEN	FAIM3	0.0733	0	-0.12582427
PTEN	ZNF326	0.0733	0	-0.156844151
PTEN	PRSS16	0.0733	0	-0.129774083
PTEN	PARN	0.0734	0	-0.019622194
PTEN	FGF19	0.0734	0	-0.178133697
PTEN	VAMP2	0.0734	0	-0.004003169
PTEN	TPMT	0.0734	0	-0.067753853

PTEN	FBXO22	0.0734	0	-0.367918012
PTEN	CATSPERG	0.0734	0	0.055570448
PTEN	B3GALT5	0.0734	0	-0.314872182
PTEN	LYPD3	0.0735	0	0.003206411
PTEN	EXOC3	0.0735	0	0.000104716
PTEN	SCGB1D1	0.0736	0	-0.000310042
PTEN	C9orf106	0.0736	0	-0.009808283
PTEN	JPH4	0.0736	0	-0.008887033
PTEN	CYP4F12	0.0736	0	-0.000639368
PTEN	HSN2	0.0736	0	-0.133182057
PTEN	SNORD114-24	0.0736	0	-4.83E-07
PTEN	ZNF382	0.0737	0	-0.079053355
PTEN	CA4	0.0737	0	-1.13E-05
PTEN	STK4	0.0737	0	-0.065680472
PTEN	A4GNT	0.0739	0	-0.285528012
PTEN	GYG1	0.0739	0	-0.084987778
PTEN	CRB1	0.0739	0	-0.322170935
PTEN	ADAM9	0.0739	0	0.009628455
PTEN	EXPH5	0.0739	0	-0.060532373
PTEN	IFT52	0.074	0	0.015126685
PTEN	C10orf96	0.074	0	-0.170704081
PTEN	KCNIP2	0.074	0	-0.170602785
PTEN	MS4A8B	0.0741	0	-0.002035551
PTEN	TRIM59	0.0741	0	-0.40076364
PTEN	HPS4	0.0741	0	-0.045277568
PTEN	RWDD2A	0.0742	0	-0.080584192
PTEN	FOXQ1	0.0742	0	-0.040951101
PTEN	ANP32A	0.0742	0	0.095909791
PTEN	MPI	0.0742	0	0.126982305
PTEN	TBC1D13	0.0742	0	-0.008348192
PTEN	LOC153328	0.0743	0	-0.023308911
PTEN	LRRC3	0.0743	0	0.029811675
PTEN	GRM4	0.0743	0	-0.00045938
PTEN	APLNR	0.0743	0	-0.192534378
PTEN	C9orf169	0.0744	0	-9.63E-09
PTEN	DEFB118	0.0744	0	-0.325655225
PTEN	PRPF8	0.0744	0	0.005723133
PTEN	GFI1B	0.0744	0	-0.027552874
PTEN	GABRB3	0.0744	0	-0.221556615
PTEN	LGI2	0.0745	0	-0.403429219
PTEN	TTC36	0.0745	0	-4.05E-07

PTEN	MRFAP1L1	0.0745	0	-0.021407528
PTEN	TIAM1	0.0745	0	-0.447620527
PTEN	CYP17A1	0.0745	0	-5.14E-05
PTEN	PXN	0.0745	0	-0.003421515
PTEN	VGLL4	0.0746	0	-0.006279128
PTEN	ARHGAP23	0.0746	0	-0.016329251
PTEN	PZP	0.0746	0	-0.00211912
PTEN	EIF3IP1	0.0746	0	-0.028247856
PTEN	RXRG	0.0746	0	-0.023704584
PTEN	ZNF705A	0.0746	0	-0.48112417
PTEN	HOXD4	0.0746	0	-0.003611612
PTEN	C6orf126	0.0746	0	-0.00875152
PTEN	GALNT13	0.0747	0	-0.242755025
PTEN	PLAGL1	0.0747	0	-0.252130466
PTEN	SLC14A2	0.0747	0	-0.125690079
PTEN	DALRD3	0.0747	0	-0.002343187
PTEN	PTPRC	0.0747	0	0.191761123
PTEN	LDLR	0.0748	0	-0.063782874
PTEN	P2RY14	0.0748	0	-0.129098248
PTEN	TCEAL3	0.0748	0	-0.020899349
PTEN	RAVER2	0.0749	0	-0.18526576
PTEN	HSD3B1	0.0749	0	-0.030760635
PTEN	HTR3B	0.0749	0	-0.000611732
PTEN	C14orf159	0.075	0	-0.010770169
PTEN	PGBD2	0.0751	0	-0.029861347
PTEN	PI4K2B	0.0751	0	0.014583042
PTEN	DDX3Y	0.0752	0	-0.327822003
PTEN	TSNAXIP1	0.0752	0	-2.26E-06
PTEN	USP28	0.0752	0	-0.260589071
PTEN	CXorf27	0.0753	0	-0.000135433
PTEN	SNORD113-7	0.0753	0	-0.000319197
PTEN	SERPINI2	0.0753	0	-0.166048246
PTEN	GGTA1	0.0753	0	0.06342192
PTEN	MFHAS1	0.0753	0	-0.125843072
PTEN	DPYSL2	0.0753	0	0.029229453
PTEN	LUC7L2	0.0755	0	-0.026292587
PTEN	LOC116437	0.0755	0	-0.098146161
PTEN	C14orf79	0.0755	0	-0.007519806
PTEN	WDFY4	0.0756	0	-0.254942605
PTEN	NUP188	0.0756	0	0.009083386
PTEN	GPR182	0.0756	0	-0.018728767

PTEN	TTC9	0.0756	0	0.013805794
PTEN	FLT4	0.0756	0	-0.37440991
PTEN	LOC340017	0.0757	0	-0.133837142
PTEN	MUC4	0.0757	0	-0.167884203
PTEN	BDNF	0.0757	0	-0.725962998
PTEN	GPR161	0.0757	0	-0.17304525
PTEN	PDP1	0.0758	0	-0.069556123
PTEN	FMO2	0.0758	0	-0.167363477
PTEN	PROL1	0.0758	0	-0.151734141
PTEN	PMP2	0.0758	0	-0.067018694
PTEN	RPL23P8	0.0758	0	-0.015870603
PTEN	TPRN	0.0758	0	0.00161068
PTEN	SPDYE1	0.0758	0	-0.028721018
PTEN	C22orf26	0.0758	0	-0.301549016
PTEN	SMG7	0.0758	0	-0.201228251
PTEN	DMP1	0.0758	0	-0.099168267
PTEN	CCDC15	0.0759	0	-0.332571729
PTEN	SLC22A17	0.0759	0	-8.91E-05
PTEN	KCNB2	0.0759	0	-0.338310041
PTEN	B3GALT2	0.0759	0	-0.24953284
PTEN	KIAA0652	0.076	0	-0.0231291
PTEN	MAP2K6	0.076	0	-0.06747094
PTEN	TMEM145	0.0761	0	-0.001489288
PTEN	NAT8	0.0761	0	-0.012031718
PTEN	NCRNA00094	0.0761	0	-0.000960845
PTEN	NCRNA00188	0.0761	0	0.194915851
PTEN	EGLN1	0.0761	0	-0.182851558
PTEN	RND1	0.0761	0	-0.134099495
PTEN	LOC723972	0.0762	0	-0.464020895
PTEN	ATP11C	0.0762	0	-0.193950163
PTEN	TMEM154	0.0762	0	-0.231592146
PTEN	RAB3D	0.0762	0	-0.155310481
PTEN	GRM7	0.0762	0	-0.524109519
PTEN	PLCD4	0.0763	0	-0.016485975
PTEN	ATRIP	0.0763	0	-3.08E-07
PTEN	PCDH9	0.0764	0	-0.50817958
PTEN	YWHAB	0.0764	0	0.180677389
PTEN	PYDC1	0.0764	0	0.001430635
PTEN	RERGL	0.0764	0	-0.068352718
PTEN	FGF6	0.0764	0	-0.000353521
PTEN	GAD2	0.0764	0	-0.029374349

PTEN	YTHDF2	0.0765	0	-0.245866222
PTEN	STX2	0.0765	0	-0.139172012
PTEN	MC2R	0.0766	0	-0.276129008
PTEN	POLR1B	0.0766	0	-0.169495691
PTEN	PCMTD1	0.0767	0	0.16486206
PTEN	SNORD70	0.0767	0	-1.03E-06
PTEN	FBP1	0.0767	0	0.000790251
PTEN	HRC	0.0767	0	-3.04E-05
PTEN	TP73	0.0768	0	-0.013959116
PTEN	TADA1	0.0768	0	-0.126453333
PTEN	IRX2	0.0768	0	-0.19896414
PTEN	SERAC1	0.0768	0	-0.159654776
PTEN	GNAI1	0.077	0	-0.483424345
PTEN	CCDC122	0.077	0	-0.164671182
PTEN	NCBP1	0.077	0	-0.157073316
PTEN	GOLGA2	0.077	0	0.070825631
PTEN	SGCA	0.077	0	-0.000760818
PTEN	ARF3	0.077	0	0.115890829
PTEN	GPRC5C	0.0771	0	-0.012517131
PTEN	LOC149134	0.0771	0	-0.011903474
PTEN	C12orf68	0.0771	0	-0.493127956
PTEN	TMEM204	0.0771	0	-0.007834744
PTEN	IFNA8	0.0772	0	-0.190546752
PTEN	ZSCAN12P1	0.0772	0	-0.125808467
PTEN	FBLN5	0.0772	0	-0.00921914
PTEN	AMFR	0.0773	0	0.062631263
PTEN	MTERF	0.0773	0	-0.251136079
PTEN	NLRP14	0.0773	0	-0.154300336
PTEN	RAGE	0.0774	0	-0.009129043
PTEN	BET1L	0.0774	0	0.000671779
PTEN	GNPDA1	0.0774	0	0.139180327
PTEN	MCTP2	0.0775	0	-0.086836293
PTEN	MKNK2	0.0775	0	0.071083457
PTEN	PATE2	0.0776	0	-0.228098447
PTEN	C1orf129	0.0777	0	-0.392580473
PTEN	TEF	0.0778	0	-0.031657603
PTEN	ZHX2	0.0778	0	-0.006147476
PTEN	BCL2L2	0.0779	0	-0.254324992
PTEN	KDM5D	0.0779	0	-0.027784226
PTEN	ANPEP	0.0779	0	0.007178925
PTEN	TCEAL4	0.0779	0	-0.026668859

PTEN	CCDC3	0.078	0	0.000158432
PTEN	GIMAP2	0.078	0	-0.240040695
PTEN	BTN3A1	0.078	0	0.029630816
PTEN	C9orf171	0.0781	0	-0.034178432
PTEN	RSPO3	0.0781	0	-0.048420588
PTEN	RNASE6	0.0781	0	-0.005128078
PTEN	ZNF670	0.0781	0	-0.017335581
PTEN	C22orf31	0.0782	0	-0.176244113
PTEN	C15orf54	0.0782	0	-0.341310825
PTEN	APOBEC4	0.0782	0	-0.368883122
PTEN	DENND2D	0.0782	0	-0.128560865
PTEN	KCNK13	0.0782	0	-0.017278806
PTEN	GAL3ST4	0.0782	0	-0.009739665
PTEN	ZBTB7A	0.0783	0	0.055075709
PTEN	TMEM136	0.0783	0	-0.000476447
PTEN	RNF13	0.0783	0	-0.207296809
PTEN	N4BP2L1	0.0784	0	-0.247092987
PTEN	KIAA0513	0.0784	0	-0.237793568
PTEN	LOC728819	0.0785	0	-0.046438499
PTEN	PNLDC1	0.0785	0	-0.000554886
PTEN	FMO3	0.0785	0	-0.190829435
PTEN	OTOR	0.0786	0	-0.01564272
PTEN	TLR5	0.0786	0	-0.002576045
PTEN	MIS12	0.0786	0	-0.009976592
PTEN	LOC100124692	0.0786	0	-0.182125089
PTEN	ZXDC	0.0786	0	-0.052697873
PTEN	C1orf107	0.0786	0	-0.013982463
PTEN	CCDC88A	0.0786	0	-0.274992085
PTEN	EEPD1	0.0787	0	0.08787944
PTEN	UBN1	0.0787	0	0.160759095
PTEN	C1orf127	0.0787	0	-0.006363194
PTEN	HAVCR2	0.0787	0	-0.260978062
PTEN	TMPRSS11F	0.0788	0	-0.328831228
PTEN	POLH	0.0788	0	-0.064744437
PTEN	SLC7A3	0.0788	0	-0.110045615
PTEN	PEX26	0.0788	0	-0.132315831
PTEN	NACC2	0.0789	0	-0.163029039
PTEN	RAB3B	0.0789	0	-0.170847855
PTEN	LTBP3	0.0789	0	1.13E-06
PTEN	CSAD	0.079	0	0.110433287
PTEN	CARD6	0.079	0	-0.127081002

PTEN	C11orf65	0.079	0	-0.007708924
PTEN	CRYBA1	0.0791	0	-0.018055874
PTEN	SLC23A2	0.0792	0	-0.3610404
PTEN	C4orf43	0.0792	0	-0.19408109
PTEN	CA10	0.0792	0	-0.173770959
PTEN	SGK2	0.0792	0	-0.003045295
PTEN	ASB14	0.0792	0	-0.346767398
PTEN	CLIP3	0.0793	0	-0.04752582
PTEN	IGFL2	0.0793	0	-0.001181844
PTEN	TMCO1	0.0793	0	0.121758641
PTEN	KIAA0802	0.0793	0	-0.165111056
PTEN	CTF1	0.0793	0	-0.216240684
PTEN	MORF4L2	0.0794	0	0.028212194
PTEN	EFCAB1	0.0794	0	-0.394685162
PTEN	RCN2	0.0794	0	0.001269474
PTEN	KIT	0.0794	0	-0.257348086
PTEN	POLR3GL	0.0794	0	-0.000934781
PTEN	CASS4	0.0794	0	-0.024830194
PTEN	ZNF131	0.0795	0	-0.014061831
PTEN	B4GALT6	0.0795	0	-0.275907645
PTEN	TMEM132D	0.0795	0	-0.030093274
PTEN	COX16	0.0795	0	-0.242424128
PTEN	KRT12	0.0796	0	-0.086471929
PTEN	GPR75	0.0796	0	-0.181845703
PTEN	PSMD1	0.0796	0	0.091472403
PTEN	GPR87	0.0797	0	-2.20E-05
PTEN	HNRNPA1L2	0.0798	0	0.008219581
PTEN	C14orf53	0.0798	0	-0.130360222
PTEN	FZD5	0.0799	0	-0.75361288
PTEN	HAO2	0.0799	0	-0.004188958
PTEN	SLC6A4	0.0799	0	-0.43536314
PTEN	USP42	0.0799	0	-0.534092564
PTEN	TJP2	0.0799	0	-0.064834215
PTEN	KCNF1	0.0799	0	-0.000359912
PTEN	PRX	0.0799	0	-0.000130846
PTEN	GTF2A1L	0.0799	0	-0.139155505
PTEN	PGA4	0.08	0	-0.168649805
PTEN	ZNF101	0.08	0	-0.001256859
PTEN	FXC1	0.08	0	-0.059120814
PTEN	PLAU	0.08	0	-0.17180439
PTEN	SPINK9	0.0801	0	-0.001038488

PTEN	CCDC89	0.0801	0	-0.242384651
PTEN	TM6SF2	0.0801	0	-0.130901513
PTEN	ZNF550	0.0802	0	-0.018819381
PTEN	SMAD7	0.0802	0	-0.166387565
PTEN	C8orf37	0.0802	0	-0.199857989
PTEN	ZNF765	0.0803	0	-0.144955672
PTEN	OAS3	0.0803	0	0.041279872
PTEN	TOMM70A	0.0803	0	-0.032695179
PTEN	NDP	0.0803	0	-0.110801068
PTEN	SOX21	0.0803	0	-0.428075493
PTEN	PRPF4	0.0803	0	0.002278761
PTEN	SCARA3	0.0803	0	0.104798763
PTEN	LOC90246	0.0803	0	-0.179411365
PTEN	PHF11	0.0804	0	-0.00131158
PTEN	TP53BP2	0.0804	0	0.045931575
PTEN	GRK4	0.0804	0	-0.000219097
PTEN	DPH5	0.0805	0	-0.078811862
PTEN	ZBTB42	0.0805	0	-0.000723013
PTEN	CD9	0.0806	0	0.04088357
PTEN	DHX33	0.0806	0	-0.025167672
PTEN	SLC47A1	0.0806	0	-0.358102337
PTEN	P4HA1	0.0806	0	0.166679075
PTEN	KRT18	0.0806	0	0.000335254
PTEN	BTN3A3	0.0806	0	-0.150152394
PTEN	CDC5L	0.0807	0	0.007323729
PTEN	PRKRA	0.0807	0	-0.052147969
PTEN	LETM2	0.0807	0	-0.015679201
PTEN	CLDN1	0.0807	0	-0.107088804
PTEN	TTC25	0.0807	0	8.71E-05
PTEN	ABCB5	0.0808	0	-0.320742282
PTEN	DNAH2	0.0808	0	-0.001392902
PTEN	SGSM1	0.0809	0	-0.104897755
PTEN	IARS	0.0809	0	-0.031795317
PTEN	EPT1	0.0809	0	-0.004974875
PTEN	UAP1	0.0809	0	0.096523071
PTEN	HTR1D	0.0809	0	-0.028690106
PTEN	ALDH9A1	0.0809	0	0.094946188
PTEN	PAPD5	0.081	0	-0.298772466
PTEN	TMPRSS7	0.081	0	-0.02765376
PTEN	C14orf126	0.081	0	-0.040877793
PTEN	SPRY2	0.0811	0	-0.277626686

PTEN	C2CD4B	0.0811	0	-0.003705497
PTEN	C3	0.0811	0	1.74E-05
PTEN	GKAP1	0.0812	0	-0.049646339
PTEN	GSTZ1	0.0812	0	-0.004499001
PTEN	EDA	0.0812	0	-0.41930531
PTEN	TBL1Y	0.0812	0	-0.009862496
PTEN	ECT2L	0.0813	0	-0.223851515
PTEN	RAB5A	0.0813	0	-0.041750285
PTEN	NTNG1	0.0813	0	-0.220340553
PTEN	SARDH	0.0813	0	-0.007788189
PTEN	KLHL12	0.0813	0	0.011734325
PTEN	ARFGEF1	0.0814	0	0.078414122
PTEN	FRAT1	0.0814	0	-0.184793383
PTEN	DEPDC4	0.0814	0	-0.008744326
PTEN	MED6	0.0814	0	0.129178583
PTEN	SATL1	0.0815	0	-0.009877299
PTEN	USP45	0.0815	0	0.095033398
PTEN	C15orf48	0.0815	0	-0.096894559
PTEN	SMURF1	0.0815	0	-0.130304726
PTEN	TUSC5	0.0815	0	-0.003480599
PTEN	BEX5	0.0815	0	-0.051640237
PTEN	ZNF416	0.0815	0	-0.014823462
PTEN	GAS6	0.0816	0	-0.01574413
PTEN	C14orf180	0.0816	0	-5.12E-06
PTEN	ZFYVE27	0.0817	0	-0.121651753
PTEN	ATXN8OS	0.0817	0	-0.192450934
PTEN	ARSI	0.0817	0	-0.195445222
PTEN	ADCYAP1	0.0817	0	-0.152607084
PTEN	CDHR3	0.0817	0	-0.085630245
PTEN	SLC35D2	0.0817	0	-1.10E-05
PTEN	PRRG4	0.0818	0	0.000332922
PTEN	PRR15L	0.0819	0	0.041119082
PTEN	SNX12	0.0819	0	-0.15136605
PTEN	CYP19A1	0.0819	0	-0.118504671
PTEN	CASP9	0.0819	0	-0.009055305
PTEN	HIST2H2BE	0.082	0	0.000476781
PTEN	RSAD2	0.082	0	-0.156620422
PTEN	WBP4	0.082	0	-0.153500339
PTEN	COQ10B	0.082	0	-0.057160139
PTEN	IL4R	0.082	0	-0.160847229
PTEN	BEND6	0.0821	0	-0.414678856

PTEN	PRELP	0.0821	0	-0.001097072
PTEN	INTS10	0.0822	0	-0.104159627
PTEN	CCL28	0.0822	0	-0.009110212
PTEN	TBC1D14	0.0822	0	-0.013730439
PTEN	ZNF471	0.0823	0	-0.136291519
PTEN	MAG	0.0823	0	-0.160942751
PTEN	TRIM62	0.0823	0	-0.170406384
PTEN	CD33	0.0823	0	-0.010604383
PTEN	GLDN	0.0824	0	-0.204872461
PTEN	KLRAQ1	0.0824	0	-0.176458723
PTEN	LOC728276	0.0824	0	-0.000274109
PTEN	RILPL2	0.0824	0	-0.162953454
PTEN	CTNNA3	0.0825	0	-0.019452685
PTEN	DIAPH1	0.0825	0	0.001637626
PTEN	SKAP1	0.0825	0	-0.002349579
PTEN	ACSS3	0.0825	0	-0.263289749
PTEN	NECAP1	0.0826	0	-0.005581756
PTEN	CLEC3A	0.0826	0	0.186558052
PTEN	NIPA2	0.0826	0	-0.234375682
PTEN	CCDC62	0.0826	0	-0.757885148
PTEN	CYB5D2	0.0826	0	-0.00469962
PTEN	SLC37A2	0.0827	0	-0.081851536
PTEN	USP1	0.0827	0	-0.183317479
PTEN	TRRAP	0.0827	0	-0.403535855
PTEN	EXOG	0.0828	0	-0.41052571
PTEN	BCKDHB	0.0828	0	-0.243189294
PTEN	OSBPL6	0.0828	0	-0.194204409
PTEN	ABCG1	0.0828	0	-0.039469561
PTEN	LRTOMT	0.0828	0	-0.556840359
PTEN	ZNF177	0.0828	0	-0.000506162
PTEN	C15orf21	0.0828	0	-0.008031646
PTEN	KLF8	0.0828	0	-0.041341006
PTEN	COL7A1	0.0828	0	-0.142065423
PTEN	NPAS3	0.0828	0	-0.868588361
PTEN	TMEM209	0.0828	0	0.048464005
PTEN	MYCBPAP	0.0829	0	-2.46E-06
PTEN	RARRES3	0.0829	0	2.78E-05
PTEN	LRRIQ1	0.0829	0	0.011861882
PTEN	MAP3K7	0.083	0	0.032773139
PTEN	TPH2	0.083	0	-0.308873417
PTEN	TMEM169	0.083	0	-0.178410685

PTEN	ANKMY1	0.0831	0	-0.001291183
PTEN	C14orf149	0.0831	0	-0.013658975
PTEN	FAM167A	0.0831	0	-0.118392162
PTEN	CAPZA1	0.0831	0	0.165679907
PTEN	FCGR2C	0.0831	0	-0.004495695
PTEN	CANT1	0.0831	0	0.005180497
PTEN	CTNND2	0.0831	0	-0.085121547
PTEN	EHBP1	0.0831	0	-0.331457018
PTEN	RARA	0.0831	0	-0.155055316
PTEN	ZNF75A	0.0831	0	-0.282834
PTEN	HOXA11	0.0832	0	0.004847528
PTEN	GALNT3	0.0832	0	0.088911923
PTEN	PCNXL2	0.0832	0	-0.00029205
PTEN	GCET2	0.0832	0	0.107112026
PTEN	DNTTIP2	0.0833	0	-0.061697908
PTEN	TRIM9	0.0833	0	-0.229015056
PTEN	TMPRSS3	0.0834	0	-0.101515174
PTEN	AGPHD1	0.0834	0	-0.000338294
PTEN	RGN	0.0834	0	-0.027104929
PTEN	LHFPL5	0.0835	0	-0.272725183
PTEN	RAB5C	0.0835	0	0.030544137
PTEN	SCD5	0.0835	0	-0.356480511
PTEN	C1orf97	0.0836	0	-0.002714204
PTEN	LOC652276	0.0836	0	-0.140564314
PTEN	CLRN1OS	0.0836	0	-0.368587214
PTEN	UBE2J1	0.0837	0	0.058630697
PTEN	ABO	0.0837	0	-0.019701268
PTEN	WEE2	0.0837	0	-0.051680136
PTEN	DCT	0.0838	0	-0.007653581
PTEN	OXTR	0.0838	0	-0.289341365
PTEN	ST7L	0.0838	0	-0.384476011
PTEN	KIAA1530	0.0838	0	-0.018271278
PTEN	SDCCAG8	0.0839	0	-0.071849206
PTEN	CABLES1	0.084	0	-0.239827157
PTEN	KCNJ5	0.084	0	-0.159792801
PTEN	RGS12	0.084	0	0.002386238
PTEN	CPNE3	0.084	0	0.006791311
PTEN	SNORD115-26	0.0841	0	-1.20E-07
PTEN	GEMIN8	0.0841	0	-0.195404912
PTEN	DIRC2	0.0841	0	-0.409421289
PTEN	SULT1B1	0.0841	0	-0.001231951

PTEN	GRPEL2	0.0841	0	-0.052236143
PTEN	ETS1	0.0841	0	-0.083806052
PTEN	C10orf27	0.0841	0	-1.99E-07
PTEN	NXPH3	0.0842	0	-7.45E-05
PTEN	NHLH2	0.0842	0	-1
PTEN	APBB1IP	0.0843	0	-0.015763603
PTEN	C6orf192	0.0843	0	-0.347885969
PTEN	MS4A6A	0.0843	0	-0.007689352
PTEN	ECT2	0.0844	0	-0.157096284
PTEN	AQP3	0.0844	0	-0.008476171
PTEN	PSG9	0.0844	0	-0.264911378
PTEN	PCDHA2	0.0845	0	-0.399500102
PTEN	C7orf42	0.0845	0	-0.068728189
PTEN	ZSCAN2	0.0845	0	-0.409050662
PTEN	GAS2L2	0.0846	0	-0.000788592
PTEN	SLC35E3	0.0846	0	-0.011228757
PTEN	GIMAP8	0.0846	0	-0.168368794
PTEN	RABGGTB	0.0846	0	0.001690803
PTEN	CXCR2	0.0846	0	-0.419148709
PTEN	LOC387646	0.0847	0	-0.372992183
PTEN	ALDOB	0.0848	0	-0.033973799
PTEN	LOC100129716	0.0848	0	-0.327428433
PTEN	SNX31	0.0848	0	-0.498641803
PTEN	SLC15A4	0.0848	0	-0.216793817
PTEN	CEBPZ	0.0849	0	-0.01902701
PTEN	LOC441204	0.0849	0	-0.178233452
PTEN	NFE2L1	0.0849	0	0.035711751
PTEN	LOC400940	0.0849	0	-0.044279075
PTEN	FER1L5	0.0849	0	-0.173407105
PTEN	FASTKD5	0.085	0	-0.105293083
PTEN	TCEAL2	0.085	0	-0.159463605
PTEN	RGL3	0.085	0	-0.001976103
PTEN	XCR1	0.085	0	-0.001930502
PTEN	GARNL3	0.085	0	-0.01436175
PTEN	GRIN3A	0.0851	0	-0.409634735
PTEN	C1R	0.0851	0	0.021657634
PTEN	ZSCAN4	0.0851	0	-0.00070668
PTEN	RAB4A	0.0851	0	0.23344656
PTEN	SH3RF3	0.0851	0	-0.03001884
PTEN	CRYL1	0.0852	0	-0.002750983
PTEN	ZNF814	0.0852	0	-0.041949201

PTEN	SNORA81	0.0852	0	-0.065430363
PTEN	POF1B	0.0852	0	-0.144029071
PTEN	PAIP2B	0.0853	0	-0.317912584
PTEN	TREM2	0.0853	0	-0.001437286
PTEN	CRYGN	0.0853	0	-0.094477385
PTEN	MNDA	0.0854	0	-0.177775793
PTEN	DENND2A	0.0854	0	-6.86E-05
PTEN	ADC	0.0854	0	-0.005654569
PTEN	ZNF584	0.0855	0	-0.000298467
PTEN	SLC28A3	0.0855	0	-0.163585882
PTEN	KIAA1549	0.0855	0	0.144386664
PTEN	CBLN1	0.0855	0	-0.36333778
PTEN	C13orf15	0.0855	0	0.083827246
PTEN	GDF15	0.0855	0	-0.001743501
PTEN	RG9MTD3	0.0855	0	-0.137129631
PTEN	OCIAD1	0.0856	0	0.050348382
PTEN	TNFRSF10D	0.0857	0	-0.399970803
PTEN	CD226	0.0857	0	-0.338467086
PTEN	TNFSF11	0.0857	0	-0.850000739
PTEN	ZBTB7B	0.0858	0	-0.000629937
PTEN	CKMT2	0.0858	0	-0.000242588
PTEN	CD200R1	0.0858	0	-0.220923384
PTEN	KNDC1	0.0858	0	-0.008409222
PTEN	HSPA12B	0.0858	0	-0.009936922
PTEN	GAB3	0.086	0	-0.27295437
PTEN	SOX4	0.086	0	-0.215111835
PTEN	WDR66	0.0861	0	-0.005888213
PTEN	MRPS35	0.0861	0	-0.107635186
PTEN	PLEKHA2	0.0861	0	-0.019477167
PTEN	FABP3	0.0861	0	-0.216729163
PTEN	NQO1	0.0861	0	-0.343534523
PTEN	MED18	0.0862	0	-0.104358031
PTEN	TMEM37	0.0862	0	-0.013802876
PTEN	NCK1	0.0863	0	-0.310738759
PTEN	OR51E1	0.0863	0	-0.313254857
PTEN	KRTAP10-11	0.0863	0	-0.009704653
PTEN	IRAK1BP1	0.0864	0	-0.105909224
PTEN	PPP2R1B	0.0864	0	-0.16256542
PTEN	PTBP2	0.0864	0	-0.089140261
PTEN	CCDC70	0.0864	0	-0.001560576
PTEN	MYH9	0.0865	0	0.237933746

PTEN	ABCA4	0.0865	0	8.51E-05
PTEN	FAM65C	0.0866	0	-0.308571229
PTEN	ACP6	0.0866	0	-0.000340778
PTEN	UGT2A3	0.0866	0	0.064874185
PTEN	CYP51A1	0.0867	0	-0.010840535
PTEN	FAM184B	0.0867	0	-0.002463594
PTEN	FLJ44635	0.0867	0	0.121583981
PTEN	CCDC55	0.0868	0	-0.169500759
PTEN	CRIPAK	0.0868	0	-0.013251013
PTEN	EXOSC10	0.0868	0	-0.006149417
PTEN	C1orf189	0.0869	0	2.20E-07
PTEN	KRTAP3-3	0.0869	0	-0.177815706
PTEN	TSPAN14	0.0869	0	-0.12879111
PTEN	C15orf17	0.0869	0	0.00848604
PTEN	SFRS2B	0.087	0	0.008934055
PTEN	UBE2G1	0.0871	0	-0.029072471
PTEN	DCP1B	0.0871	0	-0.006663025
PTEN	LY75	0.0871	0	-0.26285922
PTEN	LOC550643	0.0872	0	0.023054153
PTEN	STARD10	0.0872	0	0.00996085
PTEN	PALM	0.0872	0	-0.009282339
PTEN	C11orf42	0.0872	0	-3.75E-06
PTEN	VAMP1	0.0873	0	-0.060487355
PTEN	CXorf22	0.0873	0	-0.016488897
PTEN	SNRNP48	0.0873	0	-0.038407408
PTEN	HNF4G	0.0873	0	-0.846145217
PTEN	C1orf103	0.0874	0	-0.113704751
PTEN	ANO5	0.0874	0	-0.557412718
PTEN	LY86	0.0874	0	-0.001822298
PTEN	SLC11A2	0.0874	0	-0.185680061
PTEN	MXRA7	0.0875	0	-0.306910545
PTEN	POFUT1	0.0875	0	0.004165861
PTEN	MRFAP1	0.0875	0	0.131359106
PTEN	BCO2	0.0875	0	-0.30702001
PTEN	C15orf44	0.0876	0	-0.062919993
PTEN	DNAH14	0.0876	0	-0.22115543
PTEN	TLR2	0.0876	0	-0.263046274
PTEN	PHF14	0.0876	0	-0.136589361
PTEN	RSRC1	0.0876	0	-0.028680788
PTEN	PLCB1	0.0876	0	-0.25149389
PTEN	APOM	0.0876	0	-1.70E-05

PTEN	RNGTT	0.0876	0	-0.277167216
PTEN	INPP4A	0.0876	0	-0.119544522
PTEN	SYAP1	0.0877	0	0.033624775
PTEN	HOPX	0.0877	0	-0.051757094
PTEN	SLC46A1	0.0878	0	-0.02234316
PTEN	SUSD5	0.0878	0	-0.359394294
PTEN	SNORD12	0.0878	0	-0.001518235
PTEN	MPEG1	0.0878	0	-0.152567722
PTEN	MCF2L	0.0878	0	0.043523085
PTEN	FCGBP	0.0879	0	-0.008994173
PTEN	SLFN13	0.088	0	-0.289999652
PTEN	IL24	0.088	0	-0.333794361
PTEN	LIMS2	0.088	0	-0.003845723
PTEN	DOCK2	0.0881	0	-0.052048185
PTEN	AP2A2	0.0881	0	-0.002059816
PTEN	NRCAM	0.0881	0	-0.298839823
PTEN	SHPK	0.0881	0	-0.074428332
PTEN	UPRT	0.0881	0	0.125386165
PTEN	C1orf96	0.0881	0	-0.166792686
PTEN	NBPF15	0.0882	0	-0.211477536
PTEN	FAM173B	0.0882	0	-0.164271804
PTEN	APOBEC3C	0.0883	0	-0.000245217
PTEN	C9orf85	0.0883	0	-0.012943041
PTEN	EMR3	0.0883	0	-0.003376842
PTEN	IFNGR1	0.0883	0	-0.005209199
PTEN	HIVEP2	0.0884	0	-0.586967508
PTEN	PLA2G16	0.0884	0	-0.00280013
PTEN	KIAA0913	0.0884	0	-0.000193443
PTEN	ANKH	0.0885	0	-0.362374991
PTEN	HOXC5	0.0886	0	-0.190095601
PTEN	GOLGA6B	0.0886	0	-0.000697607
PTEN	PBXIP1	0.0886	0	-0.012590021
PTEN	HNRNPF	0.0886	0	0.063473294
PTEN	TANK	0.0887	0	-0.144370129
PTEN	CPT2	0.0887	0	-0.161757359
PTEN	SPDEF	0.0887	0	-0.075335098
PTEN	RABL2A	0.0888	0	-0.001312681
PTEN	PTGER2	0.0888	0	-0.38823765
PTEN	ADSSL1	0.0889	0	-0.001370078
PTEN	KIAA0895	0.0889	0	-0.219741275
PTEN	ZNF785	0.0889	0	-0.29951982

PTEN	RBM12B	0.089	0	-0.386640462
PTEN	DTX1	0.089	0	-0.000787563
PTEN	ZNF503	0.0891	0	-0.085223824
PTEN	EIF3J	0.0891	0	0.019624582
PTEN	BCORL1	0.0891	0	-0.052491447
PTEN	WNK4	0.0892	0	-0.07795633
PTEN	FAM46A	0.0892	0	0.065042589
PTEN	KCNQ1	0.0892	0	-0.051229749
PTEN	SERINC2	0.0892	0	4.29E-06
PTEN	PJA1	0.0893	0	-0.150095997
PTEN	COPA	0.0893	0	0.005952984
PTEN	TWIST1	0.0894	0	-0.175128608
PTEN	L2HGDH	0.0894	0	-0.208349128
PTEN	RNF170	0.0894	0	-0.18838666
PTEN	WNT2B	0.0894	0	-0.172424739
PTEN	AGT	0.0894	0	-0.005046795
PTEN	CETN3	0.0895	0	-0.153586749
PTEN	SALL4	0.0895	0	-0.240051726
PTEN	ATP1B2	0.0895	0	-0.014635656
PTEN	MAML2	0.0896	0	-0.297368912
PTEN	LOC90110	0.0896	0	-0.166277745
PTEN	HPS6	0.0896	0	-0.13049164
PTEN	FRMD3	0.0896	0	-0.259194422
PTEN	LOC221122	0.0897	0	-0.30222717
PTEN	ZNF618	0.0897	0	0.01039789
PTEN	CCDC48	0.0897	0	-0.12047078
PTEN	CD300LB	0.0897	0	-0.001268393
PTEN	ASB4	0.0897	0	-0.032809354
PTEN	FCGR2A	0.0897	0	-0.04668309
PTEN	CPEB1	0.0898	0	-0.692571607
PTEN	FKSG83	0.0899	0	-0.018944082
PTEN	PARP16	0.0899	0	0.013717257
PTEN	PLAA	0.0899	0	-0.138844799
PTEN	VPS54	0.0899	0	-0.158366165
PTEN	SSH3	0.09	0	0.002191091
PTEN	C19orf36	0.09	0	-8.59E-06
PTEN	ZBTB47	0.0901	0	-0.240777586
PTEN	PM20D1	0.0901	0	-0.294284057
PTEN	GRAPL	0.0901	0	-0.000124529
PTEN	RIBC1	0.0902	0	-0.243412711
PTEN	MOXD1	0.0902	0	-0.021497162

PTEN	SNORD116-2	0.0902	0	-5.39E-05
PTEN	LPPR1	0.0903	0	-0.342265617
PTEN	KLHL18	0.0903	0	-0.053223234
PTEN	FYB	0.0903	0	-0.304314542
PTEN	GDAP2	0.0903	0	-0.202912479
PTEN	ARF4	0.0903	0	0.262094194
PTEN	GPR4	0.0903	0	-0.425890732
PTEN	TMEM194B	0.0904	0	-0.024797619
PTEN	MRPL19	0.0904	0	-0.002280495
PTEN	CYB5R1	0.0904	0	0.020073502
PTEN	SPTBN4	0.0904	0	-0.000984896
PTEN	SLC2A3	0.0905	0	-0.191974846
PTEN	CACNG4	0.0905	0	-0.01328547
PTEN	SORCS3	0.0905	0	-0.225992995
PTEN	LMO7	0.0906	0	-0.150134226
PTEN	SLC35B3	0.0906	0	-0.18007042
PTEN	COMP	0.0906	0	8.66E-08
PTEN	SGK1	0.0906	0	-0.155039761
PTEN	SELP	0.0907	0	-0.012527505
PTEN	SPHAR	0.0907	0	-0.001120441
PTEN	DBR1	0.0907	0	-0.171949241
PTEN	ABCB11	0.0907	0	-0.18305777
PTEN	SRL	0.0908	0	-0.015931295
PTEN	NDE1	0.0908	0	-0.112986703
PTEN	FNDC5	0.0908	0	-0.201511483
PTEN	ZMAT1	0.0909	0	-0.084192189
PTEN	MGC4473	0.0909	0	-0.012452888
PTEN	HSD3B7	0.0909	0	0.072010846
PTEN	CACNA2D3	0.091	0	-0.243735431
PTEN	PFKFB1	0.091	0	-3.64E-06
PTEN	EPYC	0.0911	0	-0.02383257
PTEN	ZNF185	0.0911	0	-0.094388258
PTEN	NSMCE4A	0.0911	0	-0.173486227
PTEN	C14orf139	0.0912	0	-0.07119544
PTEN	CCDC144C	0.0912	0	-0.505757568
PTEN	ME3	0.0912	0	-0.000918929
PTEN	GSC	0.0912	0	-0.010110144
PTEN	SNX5	0.0912	0	-0.06471701
PTEN	B3GNT8	0.0912	0	-4.52E-05
PTEN	DHRS9	0.0912	0	-0.010817424
PTEN	RBMY1B	0.0913	0	-0.452374505

PTEN	CNTD1	0.0913	0	-0.211359487
PTEN	ZNF140	0.0913	0	-0.064464315
PTEN	IRS2	0.0913	0	0.037934545
PTEN	DSPP	0.0913	0	-0.1793482
PTEN	CYP11A1	0.0913	0	-4.03E-07
PTEN	NOLC1	0.0913	0	-0.125457635
PTEN	ZFP36	0.0915	0	-0.084496533
PTEN	ANO4	0.0915	0	-0.525962194
PTEN	DNAJB5	0.0915	0	-0.148683538
PTEN	GDAP1L1	0.0916	0	-0.134553111
PTEN	PP14571	0.0916	0	-0.191027632
PTEN	DARS	0.0917	0	-0.175824408
PTEN	KIAA1239	0.0917	0	-0.256232616
PTEN	BMP1	0.0918	0	-0.047573522
PTEN	MRC1	0.0918	0	-0.359811316
PTEN	SLC20A2	0.0919	0	-0.163343695
PTEN	NCRNA00095	0.0919	0	-0.01451257
PTEN	PIK3C2B	0.0919	0	0.132254564
PTEN	FGG	0.0919	0	-0.116560866
PTEN	HLF	0.0919	0	-0.061185172
PTEN	BRMS1L	0.092	0	-0.339461616
PTEN	DCAF12	0.092	0	-0.367727135
PTEN	C21orf82	0.0921	0	-0.305370583
PTEN	TAGLN	0.0921	0	0.215641813
PTEN	VHL	0.0922	0	-0.002210582
PTEN	ATXN10	0.0922	0	-0.014067697
PTEN	IFIT3	0.0922	0	-0.030572864
PTEN	TTC19	0.0922	0	-0.011994754
PTEN	DSCAML1	0.0923	0	-0.170681173
PTEN	MTERFD3	0.0923	0	-0.006886806
PTEN	HNRPDL	0.0923	0	-0.166636212
PTEN	ASRGL1	0.0923	0	-0.019093549
PTEN	NMNAT2	0.0924	0	-0.467151139
PTEN	PIK3IP1	0.0924	0	-0.000236411
PTEN	CIDEA	0.0924	0	-0.000153614
PTEN	C14orf104	0.0925	0	-0.041096025
PTEN	IFNA14	0.0925	0	-0.000121848
PTEN	WFS1	0.0925	0	-0.071805169
PTEN	SUPT6H	0.0926	0	-0.000954034
PTEN	GPN3	0.0926	0	-0.038573745
PTEN	C6orf222	0.0927	0	-0.017375167

PTEN	TBC1D16	0.0927	0	-0.001099853
PTEN	AGPAT5	0.0927	0	-0.200231974
PTEN	RSPH4A	0.0927	0	-0.387777214
PTEN	RFESD	0.0928	0	-0.015290958
PTEN	KIAA0467	0.0928	0	-0.031568142
PTEN	MRO	0.0928	0	-0.283530287
PTEN	C1orf230	0.0928	0	-0.011984298
PTEN	TNFSF15	0.0929	0	-0.004399194
PTEN	ARFGAP3	0.0929	0	-0.188423372
PTEN	MFSD8	0.093	0	-0.148883226
PTEN	TSGA10	0.093	0	-0.619565548
PTEN	SFXN1	0.093	0	-0.157162545
PTEN	UNC50	0.093	0	-0.009626155
PTEN	ISLR2	0.093	0	-0.021643777
PTEN	CRHR2	0.0931	0	-3.76E-06
PTEN	GDPD4	0.0931	0	-0.003215392
PTEN	XPO1	0.0931	0	0.15246574
PTEN	DDIT4L	0.0931	0	-0.363357062
PTEN	C19orf55	0.0932	0	-0.005951987
PTEN	RPL10A	0.0932	0	0.001141028
PTEN	ETNK2	0.0932	0	0.16065105
PTEN	RARB	0.0932	0	-0.173860189
PTEN	C9orf103	0.0933	0	-0.01059043
PTEN	RBMS1	0.0934	0	-0.224691173
PTEN	ALKBH3	0.0934	0	-0.141761358
PTEN	CAMSAP1	0.0935	0	-0.448098307
PTEN	CDC42EP3	0.0935	0	-0.213688082
PTEN	LOC339290	0.0935	0	-0.009002511
PTEN	LTBP1	0.0935	0	-0.229253524
PTEN	TMEM50A	0.0936	0	0.009034388
PTEN	NAMPT	0.0936	0	0.049559485
PTEN	LOC100129055	0.0936	0	-0.547317227
PTEN	LGR5	0.0937	0	-0.009079434
PTEN	CLEC1A	0.0937	0	-0.167246859
PTEN	NUP62CL	0.0937	0	-0.240275845
PTEN	GLYR1	0.0937	0	0.101438281
PTEN	GOLGA6A	0.0938	0	-0.000697679
PTEN	TSPAN9	0.0938	0	-0.001188333
PTEN	ART5	0.0939	0	-0.157278209
PTEN	NPFFR2	0.0939	0	-0.128139166
PTEN	ARHGEF5	0.094	0	-0.086798465

PTEN	SPAG17	0.094	0	-0.327675742
PTEN	SRCIN1	0.0941	0	-0.015558264
PTEN	FXYD1	0.0941	0	-1.28E-06
PTEN	SNAPIN	0.0941	0	0.13185505
PTEN	PCDHA7	0.0941	0	-0.403568438
PTEN	LIAS	0.0941	0	-0.00301635
PTEN	SLC5A12	0.0941	0	-0.381580218
PTEN	TIE1	0.0942	0	-0.005758913
PTEN	THAP10	0.0942	0	-0.125715719
PTEN	KIAA1586	0.0942	0	-0.028010073
PTEN	ZNF684	0.0943	0	-0.328791044
PTEN	C14orf147	0.0943	0	-0.170276135
PTEN	MDGA1	0.0944	0	-8.79E-06
PTEN	SPDYA	0.0944	0	-0.089184953
PTEN	FSTL4	0.0944	0	0.034616267
PTEN	FANCF	0.0944	0	-0.008617894
PTEN	C1orf110	0.0944	0	-0.36078087
PTEN	CD276	0.0944	0	-0.001596718
PTEN	ATP8B3	0.0945	0	-0.005734751
PTEN	GTF2E1	0.0945	0	0.149037885
PTEN	ABCC2	0.0946	0	-0.025583532
PTEN	BTNL9	0.0946	0	-0.040538821
PTEN	APOF	0.0946	0	-0.396783075
PTEN	NETO1	0.0947	0	-0.262318851
PTEN	MRPL50	0.0947	0	-0.163053726
PTEN	RETSAT	0.0947	0	-0.160185361
PTEN	ODF2L	0.0948	0	-1.36E-06
PTEN	TYRP1	0.0948	0	-0.091071078
PTEN	SLC25A44	0.0948	0	-0.315523872
PTEN	CXorf1	0.0949	0	-0.207868732
PTEN	CAMK2A	0.0949	0	-0.177261922
PTEN	CHML	0.0949	0	-0.128329137
PTEN	TMEM130	0.0949	0	-0.10586324
PTEN	SLC37A3	0.095	0	-0.000800843
PTEN	CA3	0.095	0	-0.209655169
PTEN	RIMS4	0.0951	0	-0.08353048
PTEN	CDCP1	0.0951	0	-0.135939671
PTEN	CERCAM	0.0952	0	-0.001592327
PTEN	KIAA1967	0.0952	0	-5.50E-05
PTEN	AKAP4	0.0952	0	-0.112210989
PTEN	WTAP	0.0952	0	-0.160978815

PTEN	PTPLAD1	0.0953	0	0.092535968
PTEN	AQP7	0.0953	0	-0.007052238
PTEN	AGTR2	0.0954	0	-0.275892423
PTEN	USP50	0.0954	0	-0.154657099
PTEN	LRMP	0.0955	0	-0.009113439
PTEN	MMD	0.0955	0	-0.203755817
PTEN	NNMT	0.0956	0	-3.31E-07
PTEN	KCNE1	0.0956	0	-0.314752832
PTEN	RABL5	0.0956	0	-0.140121819
PTEN	PLCXD2	0.0956	0	-0.358190366
PTEN	ENPP3	0.0957	0	-0.192529551
PTEN	ZNF169	0.0957	0	-0.14558069
PTEN	ACPP	0.0957	0	-0.404716619
PTEN	KLHL13	0.0957	0	-0.144491626
PTEN	BHLHE22	0.0957	0	-0.271541333
PTEN	SFRS1	0.0958	0	0.098086647
PTEN	PKIA	0.0958	0	-0.284877684
PTEN	C10orf18	0.0958	0	0.135278494
PTEN	FARP1	0.0959	0	-0.07506735
PTEN	ALB	0.0959	0	-0.320509126
PTEN	ARHGEF7	0.096	0	-0.130930702
PTEN	DLEU7	0.096	0	-0.232429825
PTEN	PROM2	0.096	0	0.025297832
PTEN	PELO	0.0961	0	0.090069955
PTEN	PAPLN	0.0961	0	-0.13402952
PTEN	PDK3	0.0961	0	-0.021076569
PTEN	NFATC3	0.0961	0	-0.102702026
PTEN	EPRS	0.0961	0	-0.000116983
PTEN	PLRG1	0.0962	0	-0.00136866
PTEN	ADH5	0.0963	0	0.009870011
PTEN	ZDHHC22	0.0963	0	-0.515134506
PTEN	C3orf20	0.0964	0	-0.00770501
PTEN	FIGF	0.0964	0	-0.010365998
PTEN	NEURL	0.0965	0	-0.000265572
PTEN	CASQ1	0.0965	0	-0.049860453
PTEN	ATP6V1G3	0.0965	0	-0.286482775
PTEN	OSBPL9	0.0966	0	-0.084519187
PTEN	ZNF558	0.0966	0	-0.102218244
PTEN	FRG1	0.0967	0	0.000327382
PTEN	CD59	0.0967	0	-0.008948316
PTEN	TPSAB1	0.0967	0	-0.001508243

PTEN	TRPC4	0.0967	0	-0.179678021
PTEN	FIGNL2	0.0968	0	-0.032032278
PTEN	SLC30A8	0.0968	0	0.008681182
PTEN	RAP1A	0.0968	0	-0.005267765
PTEN	CCBP2	0.0968	0	-0.153203456
PTEN	GRIA4	0.0969	0	-0.291669803
PTEN	C15orf32	0.0969	0	-0.039495829
PTEN	DLL4	0.0969	0	-0.10991707
PTEN	ZNF323	0.0969	0	-0.269873514
PTEN	PGAP1	0.097	0	-0.01425432
PTEN	OR51E2	0.097	0	-0.260386122
PTEN	ZNF354B	0.097	0	-0.011363774
PTEN	DIS3L2	0.097	0	-0.143458023
PTEN	MORN1	0.097	0	-0.007079393
PTEN	HECA	0.0971	0	-0.193089364
PTEN	TRIM35	0.0971	0	0.063629378
PTEN	C13orf18	0.0972	0	-0.100807631
PTEN	ANO3	0.0972	0	-0.252587042
PTEN	COL6A2	0.0972	0	0.009292423
PTEN	STX18	0.0973	0	-0.0054362
PTEN	CRKL	0.0973	0	-0.011168867
PTEN	ANXA2	0.0973	0	0.030506899
PTEN	ISCU	0.0973	0	-0.069849633
PTEN	TSHB	0.0973	0	-0.177991264
PTEN	ZER1	0.0974	0	-0.003904199
PTEN	OGT	0.0974	0	-0.152875288
PTEN	FBXO39	0.0974	0	-0.337059742
PTEN	INMT	0.0974	0	-0.005034485
PTEN	DDHD1	0.0975	0	-0.009634029
PTEN	TMEM25	0.0976	0	-0.088487983
PTEN	KIAA0664P3	0.0976	0	-0.062530159
PTEN	PCDHGC4	0.0976	0	-0.001694553
PTEN	GDF10	0.0977	0	-0.169499817
PTEN	SET	0.0977	0	0.309292841
PTEN	GPR109B	0.0977	0	-0.160215078
PTEN	PPP2R3C	0.0977	0	-0.001942771
PTEN	DNAJB12	0.0979	0	-0.008121806
PTEN	MUT	0.0979	0	0.019882828
PTEN	METT10D	0.0981	0	-0.005297202
PTEN	AASDHPPT	0.0981	0	0.044413049
PTEN	FASTKD3	0.0982	0	-0.163069756

PTEN	SMG6	0.0982	0	-0.000451972
PTEN	VDAC2	0.0982	0	-0.034213878
PTEN	THTPA	0.0982	0	-0.004090448
PTEN	TXNDC9	0.0983	0	-0.079009508
PTEN	HHATL	0.0983	0	-1.31E-05
PTEN	VPS45	0.0983	0	-0.142551119
PTEN	C1RL	0.0984	0	-0.035808411
PTEN	DNAJC21	0.0984	0	-0.081800676
PTEN	PRMT5	0.0984	0	0.026804843
PTEN	MYO1C	0.0984	0	0.166021915
PTEN	MRRF	0.0985	0	-0.067558762
PTEN	GLIS2	0.0985	0	-0.020042877
PTEN	KIAA0100	0.0985	0	-0.013031888
PTEN	ADIPOR2	0.0985	0	0.204138152
PTEN	PTPN18	0.0986	0	-0.074623702
PTEN	GIMAP6	0.0987	0	-0.268511741
PTEN	LBH	0.0987	0	-0.135674707
PTEN	LOC374443	0.0987	0	-0.126664203
PTEN	C20orf117	0.0987	0	0.003839279
PTEN	ADAMTS1	0.0987	0	-0.265793632
PTEN	PFKFB2	0.0987	0	0.005525962
PTEN	D4S234E	0.0987	0	-0.136423934
PTEN	MAP3K3	0.0988	0	-0.061211317
PTEN	ALOX15B	0.0988	0	0.002415257
PTEN	OTUD3	0.0989	0	-0.069102651
PTEN	EZH1	0.099	0	-0.003493025
PTEN	C20orf132	0.099	0	-0.018361319
PTEN	TXLNA	0.0991	0	0.000473895
PTEN	CSDE1	0.0991	0	0.524673622
PTEN	PAXIP1	0.0991	0	-0.236950853
PTEN	PRPS1	0.0991	0	-0.032248108
PTEN	SLC22A18AS	0.0991	0	-0.00193938
PTEN	ARMC3	0.0991	0	-0.00777368
PTEN	C1orf183	0.0992	0	-0.002512646
PTEN	ZNF480	0.0992	0	-0.108741856
PTEN	KIAA1211	0.0992	0	0.014416767
PTEN	ERBB2	0.0992	0	0.086195305
PTEN	ABCD4	0.0992	0	-0.231922297
PTEN	RNF43	0.0993	0	-0.013359493
PTEN	TNFAIP8	0.0993	0	-0.422664089
PTEN	SNORA28	0.0993	0	-0.00025804

PTEN	SLC14A1	0.0993	0	-0.364046374
PTEN	TSGA14	0.0994	0	-0.049944507
PTEN	CTH	0.0994	0	0.073715717
PTEN	PRKCA	0.0994	0	-0.125762242
PTEN	MAP7	0.0994	0	-0.296939379
PTEN	TFEC	0.0995	0	-0.18221808
PTEN	PTGDR	0.0995	0	-0.141920866
PTEN	KIAA1199	0.0995	0	-0.037441556
PTEN	FLJ34503	0.0995	0	-0.257974393
PTEN	CBX7	0.0996	0	-0.002299756
PTEN	RIMBP2	0.0996	0	-0.156265019
PTEN	PCDHAC1	0.0997	0	-0.443053037
PTEN	CMPK1	0.0997	0	0.346121198
PTEN	1-Dec	0.0997	0	-0.053275232
PTEN	RAB11FIP1	0.0997	0	-0.091697337
PTEN	USP14	0.0997	0	0.201635313
PTEN	C14orf19	0.0998	0	-0.001404884
PTEN	TBC1D20	0.0998	0	0.036406554
PTEN	VIT	0.0998	0	-0.091165111
PTEN	DUSP5	0.0998	0	0.032945427
PTEN	C17orf47	0.0998	0	-0.169238078
PTEN	RPP14	0.0998	0	-0.013132743
PTEN	ZNF714	0.0999	0	-0.092328491
PTEN	IGSF22	0.0999	0	-0.000127216
PTEN	NIPA1	0.0999	0	-0.132048819
PTEN	PRIM1	0.0999	0	-0.003721475
PTEN	LACRT	0.0999	0	-1.99E-05
PTEN	TCEA3	0.0999	0	0.0028083
PTEN	ALG5	0.0999	0	-0.155593208
PTEN	CHRDL1	0.1	0	-0.04738905
PTEN	TMEM67	0.1	0	-0.211928496
PTEN	USP19	0.1	0	-0.003843285
PTEN	CLEC5A	0.1	0	-0.206852041
PTEN	CBX6	0.1	0	-0.010500152
PTEN	PCOTH	0.1	0	-0.012797457
PTEN	CPN2	0.1	0	-0.008225809
PTEN	SYNPR	0.1	0	-0.236017087
PTEN	LRRC8A	0.1	0	-0.00055028
PTEN	SLC45A1	0.1	0	-0.013045275
PTEN	NTF3	0.1	0	-0.215095296
PTEN	TM4SF18	0.1	0	-0.113889437

PTEN	WWC3	0.1	0	-0.110230091
PTEN	PAK7	0.1	0	-0.323109341
PTEN	ITGB1BP3	0.101	0	-1.44E-09
PTEN	IL17RA	0.101	0	-0.141514582
PTEN	MYOM3	0.101	0	-0.280505373
PTEN	RNF219	0.101	0	-0.388468716
PTEN	IL33	0.101	0	-0.220210749
PTEN	AMIGO1	0.101	0	-0.008589854
PTEN	PROCR	0.101	0	-0.202897916
PTEN	YEATS4	0.101	0	0.010402535
PTEN	TYW1	0.101	0	-0.097907569
PTEN	PCGEM1	0.101	0	-0.275786167
PTEN	GNA12	0.101	0	-0.061935858
PTEN	TYW1B	0.101	0	-0.102334275
PTEN	KAAG1	0.101	0	-0.001692822
PTEN	MS4A3	0.101	0	-0.183665593
PTEN	C17orf71	0.101	0	-0.110801406
PTEN	PKP2	0.101	0	-0.152657401
PTEN	ZNF75D	0.101	0	-0.146455413
PTEN	KRIT1	0.101	0	-0.173049015
PTEN	ZNF571	0.101	0	-0.306534529
PTEN	EPOR	0.101	0	-0.037436653
PTEN	PLAC9	0.101	0	-0.015346991
PTEN	RHOH	0.101	0	-0.215860789
PTEN	KDM4DL	0.101	0	-0.09898491
PTEN	PCYT1B	0.101	0	-0.177108703
PTEN	PPPDE1	0.101	0	0.183428103
PTEN	RPF1	0.101	0	-0.266233015
PTEN	SECISBP2	0.101	0	-0.077907871
PTEN	GYPE	0.101	0	-0.19268123
PTEN	ST6GAL1	0.101	0	0.041280716
PTEN	C1orf113	0.101	0	-0.000265575
PTEN	WDR61	0.101	0	0.133439054
PTEN	NAA15	0.101	0	-0.409848218
PTEN	HOXA13	0.101	0	-0.482751903
PTEN	CDH10	0.101	0	-0.140590179
PTEN	ZNF418	0.101	0	-0.021016717
PTEN	PHF8	0.101	0	-0.007386158
PTEN	LOC220429	0.101	0	-0.397308705
PTEN	TNFAIP8L3	0.101	0	-0.135909536
PTEN	C1orf84	0.101	0	-0.021574202

PTEN	MAGEC1	0.102	0	-0.008967703
PTEN	SMC1A	0.102	0	0.001363834
PTEN	PPFIA1	0.102	0	-0.004816302
PTEN	DLL1	0.102	0	-0.2684711
PTEN	ITGAM	0.102	0	-0.020024241
PTEN	CCDC47	0.102	0	0.098960617
PTEN	SLC22A10	0.102	0	-0.309047713
PTEN	FAM101B	0.102	0	-0.18034365
PTEN	HERPUD2	0.102	0	-0.097297527
PTEN	RP1	0.102	0	-0.131329949
PTEN	SMC2	0.102	0	-0.176616934
PTEN	SLC13A2	0.102	0	-0.124910397
PTEN	CYBB	0.102	0	-0.062829476
PTEN	DDX52	0.102	0	0.049982586
PTEN	SPACA5	0.102	0	-0.000620882
PTEN	DOCK8	0.102	0	-0.018821453
PTEN	HDHD2	0.102	0	-0.250635541
PTEN	ZDHHC15	0.102	0	-0.347791075
PTEN	CASK	0.102	0	-0.116441432
PTEN	CFL2	0.102	0	-0.138683358
PTEN	MUCL1	0.102	0	0.021916796
PTEN	PRG4	0.102	0	-0.07907368
PTEN	ZNF398	0.102	0	-0.039984565
PTEN	GALNTL1	0.102	0	-0.003462231
PTEN	ATXN2	0.102	0	-0.217815124
PTEN	USH2A	0.102	0	-0.507906522
PTEN	FRMD5	0.102	0	-0.022274654
PTEN	OSBPL1A	0.103	0	0.090971984
PTEN	C11orf41	0.103	0	-0.351413877
PTEN	LAMC2	0.103	0	-0.101609012
PTEN	SLC23A1	0.103	0	-0.241206582
PTEN	KCNK6	0.103	0	-0.001481459
PTEN	ZNF346	0.103	0	-0.016897108
PTEN	ZFP3	0.103	0	-0.00454063
PTEN	CCR4	0.103	0	-0.141491271
PTEN	ZNF839	0.103	0	-0.161906177
PTEN	EFR3B	0.103	0	-0.036875412
PTEN	SERP1	0.103	0	0.199530377
PTEN	PCDHB6	0.103	0	-0.0243956
PTEN	CLN8	0.103	0	-7.67E-07
PTEN	FAM174B	0.103	0	-0.11632736

PTEN	GLRA3	0.103	0	-0.347552343
PTEN	KCND1	0.103	0	-0.177589682
PTEN	ZNF479	0.103	0	-0.007452569
PTEN	HSD17B12	0.103	0	-0.096718686
PTEN	ZNF350	0.103	0	-0.170019328
PTEN	KCTD6	0.103	0	-0.358244004
PTEN	CNGA2	0.103	0	-0.180237892
PTEN	MAPK14	0.103	0	0.009827363
PTEN	TMEM229B	0.103	0	-0.028096986
PTEN	IK	0.103	0	-0.002115865
PTEN	C1orf88	0.103	0	-0.17586123
PTEN	VSIG2	0.103	0	-2.96E-06
PTEN	VASH1	0.103	0	-0.01168278
PTEN	LRG1	0.103	0	-0.000116226
PTEN	ADCY2	0.103	0	-0.457457717
PTEN	CADM1	0.103	0	-0.22396971
PTEN	FOXA3	0.103	0	-0.010905501
PTEN	CDC23	0.103	0	-0.003179244
PTEN	SV2B	0.103	0	-0.211922149
PTEN	SLC39A11	0.103	0	-0.125422393
PTEN	RPL13AP17	0.103	0	-0.08081907
PTEN	FAM110B	0.103	0	-0.081376906
PTEN	DAZ4	0.103	0	-0.555738314
PTEN	FPR1	0.103	0	-0.001414166
PTEN	BICD1	0.104	0	-0.174028155
PTEN	TMLHE	0.104	0	-0.413751531
PTEN	MYEF2	0.104	0	-0.01685376
PTEN	CLDN18	0.104	0	-0.23143362
PTEN	CDC14A	0.104	0	-0.644927533
PTEN	TPT1	0.104	0	0.004267069
PTEN	SELENBP1	0.104	0	-0.034929013
PTEN	ZCCHC9	0.104	0	-0.029795947
PTEN	CRISP3	0.104	0	-0.052804333
PTEN	MRPL45	0.104	0	-0.005996377
PTEN	C12orf40	0.104	0	-0.073548939
PTEN	HRNR	0.104	0	-0.35068772
PTEN	PDXDC1	0.104	0	-0.074251467
PTEN	C10orf122	0.104	0	-0.009081942
PTEN	ANKRD20A3	0.104	0	-0.226407419
PTEN	FIG4	0.104	0	-0.014932385
PTEN	TMEM140	0.104	0	-0.142781149

PTEN	LRRC37B2	0.104	0	-0.444824657
PTEN	RAD18	0.104	0	-0.085827636
PTEN	C2orf63	0.104	0	-0.452594292
PTEN	ZNF605	0.104	0	-0.526113007
PTEN	LRRIQ4	0.104	0	-0.000869864
PTEN	ZNF566	0.104	0	-0.148073764
PTEN	ZNF439	0.104	0	-0.1718964
PTEN	C20orf177	0.104	0	-0.023325094
PTEN	IL20RA	0.104	0	-0.025293024
PTEN	ATXN7L3B	0.104	0	0.080206102
PTEN	ING3	0.104	0	-0.722918717
PTEN	KTELC1	0.104	0	-0.012684924
PTEN	EGR2	0.104	0	-0.05819632
PTEN	PIGO	0.104	0	-0.03938193
PTEN	ZNF195	0.104	0	-0.157618413
PTEN	CD177	0.104	0	-0.243595046
PTEN	CXorf21	0.104	0	-0.013807818
PTEN	MNS1	0.104	0	0.135169577
PTEN	KDM1B	0.105	0	-0.222414933
PTEN	TMEM33	0.105	0	-0.153273666
PTEN	RGS1	0.105	0	0.148276266
PTEN	ZNF271	0.105	0	-0.140450527
PTEN	ALAD	0.105	0	-0.074578901
PTEN	RAB6A	0.105	0	-0.196186057
PTEN	CACNG1	0.105	0	-0.161195688
PTEN	RNF183	0.105	0	-0.01527276
PTEN	FAM189A2	0.105	0	0.026441316
PTEN	WSCD2	0.105	0	-0.03475501
PTEN	SLCO3A1	0.105	0	-0.165483348
PTEN	TCEAL8	0.105	0	0.02975041
PTEN	CUL4A	0.105	0	-0.043315393
PTEN	TSPAN11	0.105	0	-0.000462704
PTEN	FAM19A1	0.105	0	-0.492709613
PTEN	PMAIP1	0.105	0	-0.208281916
PTEN	LFNG	0.105	0	-0.007050692
PTEN	UQCRC2	0.105	0	0.000921077
PTEN	CCDC66	0.105	0	-0.015244625
PTEN	ACTBL2	0.105	0	-0.641195104
PTEN	TAT	0.105	0	0.102392984
PTEN	DMRTC1	0.105	0	-0.068199522
PTEN	SFMBT2	0.105	0	-0.475504857

PTEN	SHROOM2	0.105	0	-0.201275007
PTEN	UBE4B	0.105	0	-0.241290104
PTEN	A2M	0.105	0	0.009746557
PTEN	FNBP4	0.105	0	-0.205305799
PTEN	CA2	0.105	0	-0.299078487
PTEN	ANKRD5	0.105	0	0.018010269
PTEN	EXTL3	0.105	0	-0.162618833
PTEN	NMBR	0.105	0	-0.127827307
PTEN	CLSTN1	0.105	0	0.007053426
PTEN	STK36	0.105	0	-0.013294546
PTEN	TPCN1	0.106	0	-0.002129117
PTEN	PHKA1	0.106	0	-0.124018663
PTEN	TXNDC8	0.106	0	-0.015792547
PTEN	MDN1	0.106	0	0.010606244
PTEN	HPS3	0.106	0	0.036105329
PTEN	SLC35D3	0.106	0	-0.480633315
PTEN	CTDSP1	0.106	0	-0.003301564
PTEN	SIX4	0.106	0	-0.0936244
PTEN	MRPL43	0.106	0	-0.174648241
PTEN	HS3ST3A1	0.106	0	-0.211016802
PTEN	FAM102A	0.106	0	0.183939049
PTEN	METTL9	0.106	0	0.213752543
PTEN	TWIST2	0.106	0	-0.047538739
PTEN	ZNF267	0.106	0	-0.186071428
PTEN	TRIM38	0.106	0	0.13597903
PTEN	UBR5	0.106	0	-0.121657596
PTEN	C14orf49	0.106	0	-0.036829017
PTEN	B4GALNT2	0.106	0	-0.009571705
PTEN	CFC1B	0.106	0	-1.89E-05
PTEN	ZNF826	0.106	0	-0.356752123
PTEN	APOL6	0.106	0	-2.18E-07
PTEN	HPGD	0.106	0	-0.368866225
PTEN	FKBP9L	0.106	0	-0.288858598
PTEN	LRP2	0.106	0	-0.272213558
PTEN	SLC8A3	0.106	0	-0.199452106
PTEN	MBD6	0.106	0	-0.06701477
PTEN	HARS2	0.106	0	-0.052985755
PTEN	SPP1	0.106	0	-0.085177988
PTEN	BRE	0.106	0	-0.017620655
PTEN	CCDC110	0.106	0	-0.123973427
PTEN	C9orf46	0.106	0	-0.013916025

PTEN	LARP1	0.106	0	0.052209887
PTEN	ZNF549	0.106	0	-0.282868938
PTEN	OLFM1	0.106	0	-0.136991435
PTEN	COG7	0.106	0	0.009608317
PTEN	RRM1	0.106	0	-0.076652616
PTEN	DDX43	0.106	0	-0.2186984
PTEN	ACCN1	0.106	0	-0.087615403
PTEN	CYP2B6	0.106	0	-0.19615812
PTEN	CCNK	0.106	0	0.120294996
PTEN	NBPF22P	0.106	0	-0.242263609
PTEN	CRYZL1	0.106	0	-0.013671178
PTEN	FAM36A	0.106	0	-0.011946832
PTEN	AFAP1L1	0.107	0	-0.033418509
PTEN	RBKS	0.107	0	-0.006497667
PTEN	SLC35E1	0.107	0	-0.182924479
PTEN	ALPK2	0.107	0	-0.293128517
PTEN	SLC41A1	0.107	0	-0.175265552
PTEN	SGEF	0.107	0	-0.355889481
PTEN	CBWD2	0.107	0	-0.180764373
PTEN	CD300LG	0.107	0	-0.02321874
PTEN	TP53I11	0.107	0	0.000947705
PTEN	LASS2	0.107	0	-0.093075041
PTEN	PTPRK	0.107	0	0.021286986
PTEN	FRMPD2L1	0.107	0	-0.01070998
PTEN	ZNF773	0.107	0	-0.005769826
PTEN	ANKRD13A	0.107	0	-0.127181164
PTEN	NDUFAF1	0.107	0	-0.000805205
PTEN	ADCY5	0.107	0	-9.15E-07
PTEN	STC1	0.107	0	0.153612739
PTEN	ZNF567	0.107	0	0.056641524
PTEN	MTMR7	0.107	0	-0.228935959
PTEN	POLN	0.107	0	-0.001419158
PTEN	BNIPL	0.107	0	-0.000431834
PTEN	SCN8A	0.107	0	-0.044895332
PTEN	DCAF6	0.107	0	-0.034692002
PTEN	MRPL35	0.107	0	-0.177596232
PTEN	LPAR3	0.107	0	-0.12709938
PTEN	SPESP1	0.107	0	-0.257197029
PTEN	BASE	0.107	0	-0.341831357
PTEN	C9orf84	0.107	0	-0.028309044
PTEN	ADAM17	0.107	0	-0.089045656

PTEN	PCDHA5	0.107	0	-0.400822074
PTEN	HACE1	0.107	0	-0.094962803
PTEN	FAM5B	0.107	0	-0.381006062
PTEN	FAR2	0.107	0	0.001302521
PTEN	MEX3B	0.107	0	-0.128692769
PTEN	TYR	0.107	0	-0.017409391
PTEN	GFPT1	0.107	0	0.003068564
PTEN	MYO5B	0.107	0	-0.246353788
PTEN	ZCCHC2	0.107	0	-0.304546484
PTEN	ANKRD29	0.107	0	-0.609535498
PTEN	PIGS	0.107	0	-0.013905491
PTEN	ZNF329	0.107	0	-0.25937604
PTEN	PIAS2	0.108	0	-0.114211794
PTEN	SCARNA6	0.108	0	-0.151365918
PTEN	ADIPOQ	0.108	0	-0.444760747
PTEN	BVES	0.108	0	-0.368339307
PTEN	STAM	0.108	0	-0.02759496
PTEN	TOP1	0.108	0	0.170901316
PTEN	DENR	0.108	0	-0.115912535
PTEN	METTL8	0.108	0	-0.068334815
PTEN	ALDH1B1	0.108	0	-0.016890423
PTEN	C14orf119	0.108	0	-0.033190111
PTEN	FAM102B	0.108	0	-0.285850385
PTEN	QSOX1	0.108	0	0.006827801
PTEN	LOC729799	0.108	0	-0.025410978
PTEN	NEFH	0.108	0	-0.053363911
PTEN	STK17A	0.108	0	-0.209406688
PTEN	CHP2	0.108	0	-0.270652609
PTEN	TLR1	0.108	0	-0.022508547
PTEN	ITM2A	0.108	0	-0.303333971
PTEN	NUCB2	0.108	0	0.102857969
PTEN	ESRRB	0.108	0	-0.183916235
PTEN	FNDC1	0.108	0	-0.197336363
PTEN	ACTR2	0.108	0	0.057492971
PTEN	PIWIL2	0.108	0	-0.012247918
PTEN	C11orf53	0.108	0	-0.179248789
PTEN	LOC646813	0.108	0	-0.267783228
PTEN	CDO1	0.108	0	-0.173780794
PTEN	MAB21L2	0.108	0	-0.033739439
PTEN	MBOAT1	0.108	0	0.157714635
PTEN	DISC1	0.108	0	-0.380609924

PTEN	CH25H	0.109	0	-0.416904523
PTEN	VAMP7	0.109	0	-0.147854943
PTEN	TSPAN5	0.109	0	0.000535382
PTEN	SERTAD2	0.109	0	-0.272391059
PTEN	BPNT1	0.109	0	0.04387972
PTEN	RNASEN	0.109	0	-0.019585025
PTEN	ST7	0.109	0	-0.145175962
PTEN	CACNA1F	0.109	0	-3.67E-06
PTEN	ANGPTL7	0.109	0	-0.023918997
PTEN	KBTBD11	0.109	0	-0.416800036
PTEN	LARP7	0.109	0	-0.181433639
PTEN	TMEM128	0.109	0	-0.214340821
PTEN	RFPL1	0.109	0	-0.181750185
PTEN	ERLIN2	0.109	0	0.144021285
PTEN	RPGR	0.109	0	-0.552584471
PTEN	ZNF30	0.109	0	-0.023289393
PTEN	SNRPN	0.109	0	7.15E-05
PTEN	CALCOCO2	0.109	0	0.043183021
PTEN	INTS4L2	0.109	0	-0.083326909
PTEN	SNORD45B	0.109	0	-0.035680222
PTEN	EIF4E2	0.109	0	-0.005600193
PTEN	SPATA5	0.109	0	-0.146414327
PTEN	SFRS6	0.109	0	-0.11786473
PTEN	ANKRD56	0.109	0	-0.027073459
PTEN	LYPD6B	0.109	0	-0.039260548
PTEN	BSDC1	0.109	0	0.032872487
PTEN	GJC1	0.109	0	-0.125672918
PTEN	C2orf44	0.109	0	-0.084701687
PTEN	C20orf96	0.109	0	-0.155687078
PTEN	CPT1A	0.109	0	0.042110683
PTEN	OXSR1	0.109	0	-0.103243632
PTEN	NOG	0.109	0	-0.171901597
PTEN	TNFRSF1A	0.109	0	-0.002572717
PTEN	AKD1	0.109	0	-0.209377639
PTEN	ZNF559	0.109	0	-0.017495543
PTEN	FLI1	0.109	0	-0.403108402
PTEN	BAZ1B	0.109	0	0.139455535
PTEN	TSPAN7	0.109	0	-0.011297288
PTEN	KIAA1310	0.11	0	-0.011846722
PTEN	ATP5S	0.11	0	-0.036798431
PTEN	CCBE1	0.11	0	-0.049555922

PTEN	C1orf124	0.11	0	-0.294476915
PTEN	TSG101	0.11	0	-0.081781554
PTEN	LIX1L	0.11	0	-0.029771318
PTEN	TULP4	0.11	0	0.133954344
PTEN	GNA11	0.11	0	2.84E-05
PTEN	ZRANB2	0.11	0	0.228359413
PTEN	RASA3	0.11	0	-0.225735527
PTEN	CSRNP1	0.11	0	-0.004236893
PTEN	BCAS1	0.11	0	-0.17682201
PTEN	DOK1	0.11	0	-0.004203178
PTEN	CYTH3	0.11	0	-0.204383149
PTEN	SPIN3	0.11	0	-0.159699388
PTEN	FECH	0.11	0	-0.02161529
PTEN	ZDHHC16	0.11	0	0.042674361
PTEN	SNX4	0.11	0	-0.363989332
PTEN	DNASE2B	0.11	0	-0.00102099
PTEN	SIDT2	0.11	0	-0.003059556
PTEN	FGF2	0.11	0	-0.505015425
PTEN	ALPK1	0.11	0	-0.16077157
PTEN	MPZL1	0.11	0	0.088052446
PTEN	ST3GAL5	0.11	0	-0.48566658
PTEN	KRT1	0.11	0	-0.049127609
PTEN	SNORA16B	0.11	0	-0.001315502
PTEN	LRRC39	0.11	0	-0.008673704
PTEN	RAB38	0.11	0	-0.056837101
PTEN	PCDHGA1	0.11	0	-0.001671631
PTEN	ATP13A4	0.11	0	-0.039435417
PTEN	WDR16	0.11	0	-0.157187709
PTEN	LRRC8C	0.11	0	-0.462863659
PTEN	GPR65	0.11	0	-0.019277847
PTEN	RIPK3	0.11	0	-0.00510532
PTEN	LY6G5C	0.111	0	-0.000364031
PTEN	PPM1L	0.111	0	-0.37107767
PTEN	GPBP1L1	0.111	0	-0.058305933
PTEN	CTPS2	0.111	0	-0.029932998
PTEN	SLC9A10	0.111	0	-0.037652688
PTEN	BCAS3	0.111	0	-0.003558291
PTEN	WHSC1L1	0.111	0	0.006027586
PTEN	GNB4	0.111	0	0.08338727
PTEN	CCL21	0.111	0	-0.161935045
PTEN	C4orf38	0.111	0	-0.010711131

PTEN	LXN	0.111	0	-0.060260551
PTEN	IL7R	0.111	0	-0.000895938
PTEN	PSG5	0.111	0	-0.439694065
PTEN	MMP19	0.111	0	-0.028006605
PTEN	KCNK15	0.111	0	-0.081555871
PTEN	C6	0.111	0	-0.176902895
PTEN	RSPH1	0.111	0	-0.001844204
PTEN	ARRB1	0.111	0	-0.034665441
PTEN	PBOV1	0.111	0	-0.235730028
PTEN	ITGA10	0.111	0	-0.270504301
PTEN	PCOLCE	0.111	0	-1.35E-06
PTEN	UBE2R2	0.111	0	-0.294291351
PTEN	PKHD1	0.111	0	-0.577582177
PTEN	C10orf108	0.111	0	-0.399459533
PTEN	ACBD5	0.112	0	0.171132757
PTEN	FKBP1B	0.112	0	-0.017278341
PTEN	SEC11A	0.112	0	-0.134400881
PTEN	RPS3A	0.112	0	7.01E-08
PTEN	SPDYE5	0.112	0	-0.203580859
PTEN	MRC2	0.112	0	-0.000662688
PTEN	SAMD12	0.112	0	-0.467573696
PTEN	AMMECR1	0.112	0	-0.169345054
PTEN	LOC151162	0.112	0	-0.042240523
PTEN	HAUS6	0.112	0	-0.381063663
PTEN	IKBKB	0.112	0	-0.109137966
PTEN	IL6R	0.112	0	0.13675986
PTEN	SOHLH2	0.112	0	-0.315591626
PTEN	CASP4	0.112	0	-0.000941104
PTEN	IFIT1	0.112	0	-0.02557588
PTEN	HOXC8	0.112	0	-0.430261788
PTEN	LPL	0.112	0	-0.060694829
PTEN	LOC100128554	0.112	0	-0.367722476
PTEN	LIN54	0.112	0	-0.09239627
PTEN	HOXA6	0.112	0	-3.32E-06
PTEN	ANKRD20A4	0.112	0	-0.465553536
PTEN	MRE11A	0.112	0	-0.063552677
PTEN	HSD17B7	0.112	0	-0.004850722
PTEN	UGT2B10	0.112	0	-0.210349704
PTEN	TUBGCP2	0.112	0	-0.000119319
PTEN	NAA25	0.112	0	-0.166163473
PTEN	CPNE8	0.112	0	-0.23372794

PTEN	TSC22D3	0.112	0	0.111060457
PTEN	ESPN	0.112	0	0.153690952
PTEN	UBXN2B	0.112	0	-0.122458381
PTEN	WDTC1	0.112	0	0.000850723
PTEN	C11orf63	0.112	0	-0.06987354
PTEN	RGS18	0.112	0	-0.318395899
PTEN	ZHX3	0.113	0	-0.007138051
PTEN	MYO18B	0.113	0	-0.182762709
PTEN	ANAPC13	0.113	0	-0.114782958
PTEN	RRN3	0.113	0	-0.03825201
PTEN	CBLN2	0.113	0	-0.015028426
PTEN	PI4KA	0.113	0	-0.012264051
PTEN	PDE6B	0.113	0	-0.012784055
PTEN	DNAJC25	0.113	0	-0.269172046
PTEN	RNFT1	0.113	0	-0.051772446
PTEN	FGGY	0.113	0	-0.039114904
PTEN	PLEKHM1	0.113	0	0.000174411
PTEN	CD248	0.113	0	-1.33E-05
PTEN	TSPAN13	0.113	0	0.107551352
PTEN	GABRG1	0.113	0	-0.819908976
PTEN	VAPA	0.113	0	0.081175769
PTEN	LZTS1	0.113	0	-0.006729625
PTEN	LOXL2	0.113	0	-0.095476634
PTEN	DNM1	0.113	0	-0.010883626
PTEN	ZHX1	0.113	0	-0.37381261
PTEN	SPINK4	0.113	0	-0.172478261
PTEN	CST9	0.113	0	-0.150101892
PTEN	PCDHA11	0.113	0	-0.397507467
PTEN	MAGEE2	0.113	0	-0.052251897
PTEN	WDR17	0.113	0	-0.230977666
PTEN	KIAA1210	0.113	0	-0.224151449
PTEN	SFXN5	0.113	0	-0.013417316
PTEN	UBE2D2	0.113	0	-0.155405793
PTEN	KCNQ5	0.113	0	-0.179765912
PTEN	SSX2IP	0.113	0	-0.076099408
PTEN	LCLAT1	0.113	0	-0.150361825
PTEN	NAA50	0.113	0	-0.048814737
PTEN	FAM65B	0.113	0	-0.256821915
PTEN	NUP153	0.113	0	-0.120079652
PTEN	TOR1B	0.113	0	-0.003145289
PTEN	TMEM86A	0.113	0	0.033150234

PTEN	SST	0.114	0	-0.207657287
PTEN	C17orf85	0.114	0	-0.041333896
PTEN	C17orf57	0.114	0	-0.389703594
PTEN	PHYHIPL	0.114	0	-0.387903552
PTEN	SDC2	0.114	0	0.067472345
PTEN	RASGRP4	0.114	0	-0.164396064
PTEN	SNORD123	0.114	0	-0.18330632
PTEN	HOXA10	0.114	0	-0.229385084
PTEN	KIF2B	0.114	0	-0.008159791
PTEN	FAM120C	0.114	0	-0.21749944
PTEN	GADD45A	0.114	0	-0.211525039
PTEN	LAMB4	0.114	0	-0.397515424
PTEN	STBD1	0.114	0	-0.174868177
PTEN	ANGPTL5	0.114	0	-0.237807618
PTEN	LIPE	0.114	0	-0.025308295
PTEN	PDE1C	0.114	0	-0.137367759
PTEN	SERPING1	0.114	0	0.000496619
PTEN	EIF3L	0.114	0	0.001112843
PTEN	SYT13	0.114	0	-0.15362711
PTEN	FGFRL1	0.114	0	-0.096647978
PTEN	NCOA3	0.114	0	-0.186984162
PTEN	COLEC10	0.114	0	-0.01832229
PTEN	TMEM170B	0.114	0	0.063180163
PTEN	LRTM1	0.114	0	-7.20E-07
PTEN	STC2	0.114	0	0.014156665
PTEN	ITFG1	0.115	0	-0.021979167
PTEN	SNCAIP	0.115	0	-0.23062015
PTEN	CEP135	0.115	0	-0.438814103
PTEN	MYST2	0.115	0	-0.027766832
PTEN	NR3C2	0.115	0	-0.497545681
PTEN	BAAT	0.115	0	-0.264384874
PTEN	LRP2BP	0.115	0	-0.268726848
PTEN	ROD1	0.115	0	0.016866548
PTEN	COBL	0.115	0	-0.019853436
PTEN	ENPP2	0.115	0	-0.366857224
PTEN	GLG1	0.115	0	-0.168699256
PTEN	LRRC57	0.115	0	-0.158531211
PTEN	TGFBI	0.115	0	0.118250835
PTEN	HBE1	0.115	0	-0.028558457
PTEN	C17orf60	0.115	0	-0.000468749
PTEN	C13orf30	0.115	0	-0.3985156

PTEN	MMP3	0.115	0	-0.103975038
PTEN	NELL2	0.115	0	-0.336521155
PTEN	IPO5	0.115	0	0.155180663
PTEN	ATP1A4	0.115	0	0.000871569
PTEN	LIPI	0.115	0	-0.201601009
PTEN	C12orf5	0.115	0	-0.033802227
PTEN	SERPINC1	0.115	0	-0.001842458
PTEN	C3orf49	0.115	0	-0.000691872
PTEN	RPS13	0.115	0	0.167217521
PTEN	SLC16A5	0.115	0	-0.000207489
PTEN	TMEM150C	0.115	0	-0.12741943
PTEN	MOBKL2C	0.115	0	-0.10689004
PTEN	ARHGAP21	0.115	0	-0.165130506
PTEN	BRAP	0.116	0	0.054948047
PTEN	FAM76B	0.116	0	-0.486030315
PTEN	OAS2	0.116	0	0.140769297
PTEN	LOC257358	0.116	0	-0.384623784
PTEN	RBM39	0.116	0	-0.11003913
PTEN	SNORD116-1	0.116	0	-5.54E-05
PTEN	ZNF610	0.116	0	-0.170051535
PTEN	OPHN1	0.116	0	-0.133636053
PTEN	FAM157A	0.116	0	-0.00056694
PTEN	AQPEP	0.116	0	-0.207370883
PTEN	KCNMB2	0.116	0	-0.540969792
PTEN	NAALAD2	0.116	0	-0.227752605
PTEN	WDR60	0.116	0	-0.181856289
PTEN	ERVFRDE1	0.116	0	-0.2700274
PTEN	MSL1	0.116	0	-0.123102635
PTEN	TUG1	0.116	0	0.01683832
PTEN	P2RY13	0.116	0	-0.072678576
PTEN	CDKN2B	0.116	0	-0.142706602
PTEN	KCTD9	0.116	0	0.144020188
PTEN	ATG4A	0.116	0	-0.074557023
PTEN	C5orf54	0.116	0	-0.340376712
PTEN	CLCF1	0.116	0	-0.199175278
PTEN	NDUFB5	0.116	0	0.000392375
PTEN	ZNF649	0.116	0	-0.164320279
PTEN	PARD6B	0.116	0	-0.031298754
PTEN	TSPAN2	0.116	0	-0.219566436
PTEN	PAIP2	0.116	0	0.038206076
PTEN	CLEC9A	0.116	0	-0.002527078

PTEN	FCHSD2	0.116	0	-0.170903375
PTEN	TOB1	0.116	0	-0.015553971
PTEN	CCDC91	0.116	0	-0.257428406
PTEN	HOXB4	0.116	0	-0.182846055
PTEN	BEYLA	0.116	0	-0.492595093
PTEN	STXBP6	0.116	0	-0.032547835
PTEN	LOC100189589	0.116	0	-0.460646561
PTEN	FAM169B	0.116	0	-0.43486022
PTEN	SFRS5	0.117	0	-0.007502512
PTEN	C6orf204	0.117	0	-0.007010233
PTEN	C10orf82	0.117	0	-5.87E-07
PTEN	SERPINA5	0.117	0	0.004405842
PTEN	ANAPC16	0.117	0	0.004136577
PTEN	EEF2	0.117	0	0.000457088
PTEN	PEX5L	0.117	0	-0.343320598
PTEN	CCDC93	0.117	0	-0.033227178
PTEN	CD300C	0.117	0	-0.010295564
PTEN	CSF1R	0.117	0	-0.017540705
PTEN	SLC22A15	0.117	0	-0.348973772
PTEN	SOX17	0.117	0	-0.352999425
PTEN	TNFRSF10B	0.117	0	-0.078266971
PTEN	PCDHA13	0.117	0	-0.400655587
PTEN	PCDHA8	0.117	0	-0.405083212
PTEN	LTF	0.117	0	-1.52E-05
PTEN	ZNF841	0.117	0	-0.038372567
PTEN	CERKL	0.117	0	-0.005763501
PTEN	RNMT	0.117	0	0.038988377
PTEN	TET1	0.117	0	-0.159150247
PTEN	DHX8	0.117	0	-0.002495057
PTEN	FAM48A	0.117	0	-0.454039883
PTEN	HOXA4	0.117	0	-0.325312023
PTEN	TMCO7	0.117	0	-0.149532722
PTEN	PIGX	0.117	0	0.023964666
PTEN	LDLRAP1	0.117	0	-0.007048636
PTEN	SMYD3	0.117	0	-0.000914601
PTEN	PLD5	0.117	0	-0.183181231
PTEN	GK5	0.118	0	-0.003536073
PTEN	ZFYVE21	0.118	0	-0.077945629
PTEN	PIP4K2C	0.118	0	-0.352019203
PTEN	CLDN22	0.118	0	-0.488041804
PTEN	GPR85	0.118	0	-0.361600859

PTEN	C3orf17	0.118	0	-0.028070629
PTEN	PIP5K1A	0.118	0	-0.009975778
PTEN	RNF34	0.118	0	0.147807506
PTEN	HOXD9	0.118	0	-0.020313306
PTEN	MTHFR	0.118	0	-0.319217653
PTEN	GNAI3	0.118	0	-0.1792061
PTEN	RSL1D1	0.118	0	0.042779779
PTEN	CRYM	0.118	0	-0.199738095
PTEN	SOX2OT	0.118	0	-0.100246379
PTEN	HDLBP	0.118	0	0.009124768
PTEN	ARHGAP31	0.118	0	-0.095132093
PTEN	SERPINA9	0.118	0	-0.007721411
PTEN	OSTalpha	0.118	0	-0.17840569
PTEN	RGS7BP	0.118	0	-0.567126156
PTEN	C12orf29	0.118	0	-0.134992627
PTEN	NHLRC4	0.118	0	-0.000141895
PTEN	RAX2	0.118	0	-0.000976185
PTEN	NFYB	0.118	0	0.124906654
PTEN	CCDC96	0.118	0	-0.002431754
PTEN	NISCH	0.118	0	-0.005093296
PTEN	SPRYD3	0.118	0	-0.003853304
PTEN	FOSB	0.118	0	-0.245551495
PTEN	MSMB	0.118	0	-0.132175203
PTEN	C1S	0.118	0	0.177005408
PTEN	11-Mar	0.118	0	-0.139285209
PTEN	PCDHGB1	0.118	0	-0.001631503
PTEN	YTHDF3	0.118	0	0.056309869
PTEN	L3MBTL3	0.118	0	-0.639126263
PTEN	CDH2	0.118	0	-0.288140009
PTEN	SVIP	0.118	0	-0.147758
PTEN	ERAP2	0.118	0	-0.259347974
PTEN	RNPC3	0.119	0	-0.013257634
PTEN	ALS2CR4	0.119	0	-0.20183536
PTEN	OTUB2	0.119	0	-0.001190145
PTEN	LACTB	0.119	0	-0.11462363
PTEN	LSAMP	0.119	0	-0.003829091
PTEN	LAMB3	0.119	0	-0.159618719
PTEN	IL25	0.119	0	-0.207227383
PTEN	UBFD1	0.119	0	-0.129977898
PTEN	MDM4	0.119	0	-0.00245177
PTEN	FAM162B	0.119	0	-0.146961985

PTEN	LOC154822	0.119	0	-0.009697533
PTEN	MRPS36	0.119	0	-0.265043143
PTEN	LGALS9C	0.119	0	-0.149164116
PTEN	DKFZp566F0947	0.119	0	-0.512153949
PTEN	AKNAD1	0.119	0	-0.027742547
PTEN	CNTNAP1	0.119	0	-0.163427992
PTEN	C15orf57	0.119	0	-0.008033885
PTEN	AUTS2	0.119	0	-0.037738243
PTEN	C16orf46	0.119	0	-0.088267916
PTEN	CEP110	0.119	0	-0.048472538
PTEN	SMURF2	0.119	0	-0.142481738
PTEN	CDSN	0.119	0	0.001314452
PTEN	GNPAT	0.119	0	-0.043123672
PTEN	DAG1	0.119	0	0.157118748
PTEN	ATP6V0D2	0.119	0	-0.240604344
PTEN	HMGCS1	0.119	0	-0.176967805
PTEN	GNAL	0.119	0	-0.053475015
PTEN	PPRC1	0.119	0	-0.045773801
PTEN	LIPT1	0.119	0	6.58E-08
PTEN	TRH	0.119	0	-0.000732753
PTEN	EFHC2	0.119	0	-0.303831829
PTEN	YIPF4	0.119	0	-0.119396065
PTEN	SLITRK3	0.119	0	-0.638227243
PTEN	GRIP1	0.119	0	-0.347893565
PTEN	KCTD7	0.119	0	0.127168715
PTEN	PANK2	0.119	0	-0.151613103
PTEN	DHRS7	0.119	0	-0.009421814
PTEN	FGF18	0.119	0	-0.2961382
PTEN	C14orf166	0.119	0	0.001440828
PTEN	EVI2A	0.119	0	-0.303429513
PTEN	SHANK2	0.119	0	-0.094318444
PTEN	DCTN5	0.12	0	-0.00181463
PTEN	TMEM188	0.12	0	-0.524143244
PTEN	CXCR1	0.12	0	-0.175880897
PTEN	GALNT2	0.12	0	0.047555947
PTEN	PTRF	0.12	0	-0.008108553
PTEN	TNRC6C	0.12	0	-0.079598098
PTEN	KDM4C	0.12	0	-0.367817211
PTEN	WSB1	0.12	0	-0.022289561
PTEN	SORT1	0.12	0	0.005499903
PTEN	G6PC2	0.12	0	-0.291995557

PTEN	PKP4	0.12	0	-0.176333024
PTEN	TOM1L2	0.12	0	-0.001397844
PTEN	VIPR1	0.12	0	0.126623795
PTEN	TTC21B	0.12	0	-0.008648605
PTEN	LINGO4	0.12	0	-0.221462319
PTEN	NPR3	0.12	0	-0.25963331
PTEN	TNR	0.12	0	-0.029623834
PTEN	FKBP15	0.12	0	-0.168683375
PTEN	KANK4	0.12	0	-0.136874663
PTEN	MAT2A	0.12	0	0.405391902
PTEN	ZNF117	0.12	0	-0.127513986
PTEN	LOC340074	0.12	0	-0.276385386
PTEN	TBX22	0.12	0	-0.250652996
PTEN	SLC27A2	0.12	0	-0.003264681
PTEN	TPP2	0.12	0	-0.250272973
PTEN	CCDC8	0.12	0	-0.192853224
PTEN	PCDHA3	0.12	0	-0.399072928
PTEN	ESYT2	0.12	0	-0.014424174
PTEN	BHMT2	0.12	0	-0.199156477
PTEN	PLLP	0.12	0	-0.139360836
PTEN	WISP2	0.12	0	-0.000145797
PTEN	EIF2B2	0.12	0	-0.078435444
PTEN	EMR4P	0.121	0	-0.179875383
PTEN	PODNL1	0.121	0	-0.153313035
PTEN	ADCY6	0.121	0	-0.052413601
PTEN	PCDHGA8	0.121	0	-0.001782105
PTEN	SCUBE1	0.121	0	-0.023668233
PTEN	STX1B	0.121	0	-0.010809688
PTEN	THRA	0.121	0	-0.013182626
PTEN	ABCC4	0.121	0	-0.018832953
PTEN	HOXD1	0.121	0	-0.252651393
PTEN	STEAP4	0.121	0	-0.109892179
PTEN	ACACA	0.121	0	-0.012914502
PTEN	CPXM2	0.121	0	-0.123417397
PTEN	PLP1	0.121	0	-0.68786075
PTEN	TRIM56	0.121	0	-0.01039374
PTEN	ZNF277	0.121	0	0.134615995
PTEN	LRRC1	0.121	0	-0.304116202
PTEN	PRAMEF14	0.121	0	-0.381215311
PTEN	CATSPER3	0.121	0	-0.001870464
PTEN	GDI2	0.121	0	-0.047161832

PTEN	C8orf41	0.121	0	-0.011258018
PTEN	PAN2	0.121	0	-0.113026199
PTEN	UMOD	0.121	0	-0.00024711
PTEN	SP5	0.121	0	-0.051596527
PTEN	ZNF662	0.121	0	-0.373051951
PTEN	HOMEZ	0.121	0	-0.323051576
PTEN	IGDCC4	0.121	0	-0.41983268
PTEN	NCAM1	0.121	0	-0.069426939
PTEN	CRISP1	0.121	0	-0.680577259
PTEN	ATP6V1G2	0.121	0	-0.125365654
PTEN	PDCD11	0.121	0	0.000352068
PTEN	NAP1L1	0.121	0	0.444952238
PTEN	CCDC112	0.121	0	-0.153096703
PTEN	RPE	0.121	0	-0.122767992
PTEN	C1orf89	0.121	0	-1.74E-05
PTEN	ELN	0.121	0	0.005966973
PTEN	RUFY1	0.121	0	-0.007742373
PTEN	ZBTB24	0.121	0	-0.064275614
PTEN	WSCD1	0.121	0	-0.181596185
PTEN	ZNF318	0.122	0	0.027076766
PTEN	DMRT2	0.122	0	-0.238970406
PTEN	SERHL2	0.122	0	-8.41E-06
PTEN	WDR73	0.122	0	0.001668287
PTEN	SOAT1	0.122	0	0.016780481
PTEN	NUP210L	0.122	0	-0.000493994
PTEN	GP5	0.122	0	-0.215520528
PTEN	ST8SIA4	0.122	0	-0.16425606
PTEN	SKAP2	0.122	0	-0.423852214
PTEN	KIAA1683	0.122	0	-7.08E-05
PTEN	HBB	0.122	0	-0.002579032
PTEN	ZNF718	0.122	0	-0.298783251
PTEN	ELAVL4	0.122	0	-0.032960516
PTEN	BFAR	0.122	0	0.004507074
PTEN	TUBA3C	0.122	0	-1.72E-06
PTEN	HEATR4	0.122	0	-0.165609946
PTEN	ZNF93	0.122	0	-9.33E-07
PTEN	GTPBP10	0.122	0	0.163966237
PTEN	UBE2QL1	0.122	0	-0.136386848
PTEN	DLEU2L	0.122	0	-0.356804589
PTEN	LONRF1	0.122	0	0.086029602
PTEN	SLC22A4	0.122	0	-0.182284065

PTEN	IL16	0.122	0	-0.134976211
PTEN	RPS23	0.122	0	0.427589516
PTEN	RPS27	0.122	0	6.26E-09
PTEN	IRF2	0.122	0	-0.209439243
PTEN	ENDOU	0.122	0	-0.161018785
PTEN	SYDE1	0.122	0	-0.189085949
PTEN	ZSWIM2	0.122	0	-0.343062259
PTEN	LRRC8B	0.122	0	-0.431479735
PTEN	PAIP1	0.122	0	0.107594995
PTEN	DUSP27	0.122	0	-0.163409922
PTEN	AQP10	0.122	0	-0.009070503
PTEN	RASSF2	0.122	0	-0.145707329
PTEN	ZNF146	0.122	0	-0.002171326
PTEN	EAF1	0.122	0	0.144705435
PTEN	KLHL1	0.122	0	-0.252342368
PTEN	NFKBIZ	0.122	0	-0.258119167
PTEN	ZNF679	0.122	0	-0.002480214
PTEN	ADAM28	0.122	0	-0.173698414
PTEN	RRAGA	0.123	0	-0.031077699
PTEN	HOXD10	0.123	0	-0.419149975
PTEN	SYPL2	0.123	0	-0.041540031
PTEN	CCDC102B	0.123	0	-0.464375682
PTEN	CUL2	0.123	0	0.01358324
PTEN	ZDHHC1	0.123	0	-0.004344743
PTEN	MMP17	0.123	0	-1.87E-06
PTEN	BCAR3	0.123	0	-0.201281562
PTEN	ARHGAP36	0.123	0	-0.039334282
PTEN	FAT1	0.123	0	-0.020567964
PTEN	MASP1	0.123	0	-0.362205807
PTEN	PPFIBP2	0.123	0	-0.00369198
PTEN	ZNF557	0.123	0	-0.067744276
PTEN	UVRAG	0.123	0	-0.06635008
PTEN	ITGA9	0.123	0	-0.48464736
PTEN	KHDRBS2	0.123	0	-0.494235421
PTEN	PTGER4	0.123	0	-0.441078055
PTEN	ASB1	0.123	0	-0.146022671
PTEN	WDR55	0.123	0	0.010897004
PTEN	PLIN1	0.123	0	-0.080943159
PTEN	ZNF540	0.123	0	-0.021118114
PTEN	ARF6	0.123	0	0.088650834
PTEN	CADPS	0.123	0	-0.036777695

PTEN	C19orf51	0.123	0	-3.40E-05
PTEN	GRIK2	0.124	0	-0.264196408
PTEN	DCAF16	0.124	0	-0.253516512
PTEN	PTGES3	0.124	0	0.107521574
PTEN	IFT46	0.124	0	-0.00777324
PTEN	PTPRN	0.124	0	-0.288063692
PTEN	PAK3	0.124	0	-0.201079753
PTEN	BEST3	0.124	0	-0.478011534
PTEN	NRAS	0.124	0	-0.133579389
PTEN	SLC22A3	0.124	0	-0.171504345
PTEN	WDFY2	0.124	0	0.007348645
PTEN	SLC43A1	0.124	0	-0.022805431
PTEN	MCART3P	0.124	0	-0.274923173
PTEN	TMEM91	0.124	0	-0.007039861
PTEN	GPNMB	0.124	0	0.099144544
PTEN	NCSTN	0.124	0	-0.002749027
PTEN	RFPL4B	0.124	0	-0.143598914
PTEN	RTN4RL1	0.124	0	-0.173444912
PTEN	TNXB	0.124	0	0.168798272
PTEN	SOX7	0.124	0	-0.18442729
PTEN	COIL	0.124	0	-0.12932059
PTEN	PNMA1	0.124	0	0.003229222
PTEN	LOC440925	0.124	0	-0.395098909
PTEN	PCDHGC5	0.124	0	-0.001688646
PTEN	R3HDM2	0.124	0	-0.12987906
PTEN	PRDM16	0.124	0	-0.341833976
PTEN	TMEM41B	0.124	0	-0.018670034
PTEN	SPATA1	0.124	0	-0.198249598
PTEN	PLD4	0.124	0	-4.35E-06
PTEN	ERI1	0.124	0	-0.278486241
PTEN	THAP1	0.124	0	-0.14723674
PTEN	AP1S3	0.124	0	0.020790468
PTEN	DHCR24	0.124	0	0.233347034
PTEN	CCDC147	0.124	0	-0.19306125
PTEN	POLA1	0.124	0	-0.007343034
PTEN	GLRX3	0.124	0	-0.027024576
PTEN	TMEM195	0.125	0	-0.304667785
PTEN	CST5	0.125	0	-0.000129096
PTEN	ZNF829	0.125	0	-0.007949642
PTEN	COQ6	0.125	0	-0.00886688
PTEN	EVPLL	0.125	0	-0.300872976

PTEN	PCDHGB3	0.125	0	-0.001681251
PTEN	COL25A1	0.125	0	-0.176202733
PTEN	HSPB7	0.125	0	-0.152042845
PTEN	ZNF138	0.125	0	-0.031034454
PTEN	PLA2G5	0.125	0	-0.478155329
PTEN	UNC5A	0.125	0	-0.02412207
PTEN	HUNK	0.125	0	-0.167017651
PTEN	FAM46C	0.125	0	-0.196887023
PTEN	KLC1	0.125	0	0.000125787
PTEN	CASP8AP2	0.125	0	-0.091757359
PTEN	UBP1	0.125	0	-0.195094623
PTEN	CDC42SE2	0.125	0	-0.618377974
PTEN	LRRC6	0.125	0	-9.53E-05
PTEN	YPEL5	0.125	0	0.215274413
PTEN	SEC23B	0.125	0	-0.184583359
PTEN	SCOC	0.125	0	-0.050014109
PTEN	CYR61	0.125	0	-0.014590375
PTEN	DLG4	0.125	0	-0.009800723
PTEN	GSPT2	0.125	0	-0.186878116
PTEN	TACR3	0.125	0	-0.182326393
PTEN	SEL1L2	0.125	0	-1.61E-05
PTEN	P2RX7	0.125	0	-0.030675284
PTEN	EPN2	0.125	0	0.021577459
PTEN	CORO6	0.125	0	-0.000648584
PTEN	POLR2B	0.125	0	-0.178286815
PTEN	CNN1	0.125	0	-0.01682138
PTEN	LCA5L	0.126	0	-0.00029414
PTEN	NMUR2	0.126	0	-0.034579423
PTEN	EEF1A1P9	0.126	0	-0.158888513
PTEN	CALML3	0.126	0	-4.84E-05
PTEN	PLK1S1	0.126	0	-2.44E-06
PTEN	SAMD5	0.126	0	-0.221608394
PTEN	PHF21A	0.126	0	-0.01872438
PTEN	GIPR	0.126	0	-0.006983021
PTEN	FAM20B	0.126	0	-0.024526671
PTEN	YY1AP1	0.126	0	-0.00100067
PTEN	BCAN	0.126	0	-0.001059874
PTEN	FAM108B1	0.126	0	-0.417277876
PTEN	DOK7	0.126	0	-0.037732341
PTEN	COPG2	0.126	0	-0.001981379
PTEN	PRKCH	0.126	0	-0.326669578

PTEN	FRMPD4	0.126	0	-0.488352265
PTEN	SYT14	0.126	0	-0.721882602
PTEN	PGAM1	0.126	0	0.190544015
PTEN	HTR2A	0.126	0	-0.323481242
PTEN	ALG2	0.126	0	0.034934488
PTEN	MBTD1	0.126	0	-0.223001383
PTEN	PHF17	0.126	0	-0.08215053
PTEN	KIF1B	0.126	0	-0.372344485
PTEN	GAN	0.126	0	-0.515267857
PTEN	DIMT1L	0.126	0	-0.000734406
PTEN	RNF150	0.126	0	-0.233098943
PTEN	SPATS2	0.126	0	-0.135147971
PTEN	TATDN3	0.126	0	-0.357826265
PTEN	HLTF	0.126	0	-0.092559855
PTEN	C6orf225	0.126	0	0.097350153
PTEN	ABHD12B	0.126	0	-0.001724408
PTEN	XG	0.126	0	-0.393224375
PTEN	LEPROTL1	0.126	0	-0.080248258
PTEN	GTF3C3	0.126	0	-0.055873788
PTEN	FAM160A1	0.127	0	-0.023377625
PTEN	XRRA1	0.127	0	-0.238644653
PTEN	UFM1	0.127	0	0.001760373
PTEN	ACSM1	0.127	0	-0.037495617
PTEN	TACC2	0.127	0	-0.26711719
PTEN	C1orf83	0.127	0	-0.323321321
PTEN	KLF2	0.127	0	-0.204479518
PTEN	ZNF614	0.127	0	-0.242987171
PTEN	ARG2	0.127	0	-0.186449138
PTEN	KCNU1	0.127	0	-0.007516554
PTEN	SNORD12B	0.127	0	-0.001944464
PTEN	COQ7	0.127	0	-0.278206407
PTEN	MYO1E	0.127	0	-0.272823089
PTEN	C3orf59	0.127	0	-0.298235843
PTEN	GUF1	0.127	0	0.004011807
PTEN	CYP7B1	0.127	0	-0.184364058
PTEN	TNFAIP1	0.127	0	-0.055431215
PTEN	EFEMP2	0.127	0	-0.000298236
PTEN	SIGLEC8	0.127	0	-0.033754114
PTEN	HS3ST3B1	0.127	0	-0.053380842
PTEN	AMBRA1	0.127	0	-0.015580764
PTEN	MMGT1	0.127	0	-0.153985483

PTEN	NAALADL1	0.127	0	-0.007666465
PTEN	ZNF275	0.127	0	0.129485913
PTEN	C14orf138	0.127	0	-0.062303763
PTEN	C9orf57	0.128	0	-0.028552237
PTEN	C7orf69	0.128	0	-0.177596076
PTEN	RFK	0.128	0	-0.256647021
PTEN	SAMD9L	0.128	0	-0.005112958
PTEN	NOV	0.128	0	-0.229006697
PTEN	LOC92973	0.128	0	-0.243929648
PTEN	TRIL	0.128	0	0.011800696
PTEN	NOTCH3	0.128	0	-0.010912094
PTEN	CD200	0.128	0	-0.008642299
PTEN	ENOX2	0.128	0	-0.158483618
PTEN	RBMX	0.128	0	0.33763721
PTEN	MEAF6	0.128	0	-0.014761804
PTEN	AKAP2	0.128	0	-0.383806994
PTEN	SULT1C4	0.128	0	-0.167015804
PTEN	DAP	0.128	0	-0.126459225
PTEN	SFRS13A	0.128	0	0.344455495
PTEN	TMEM50B	0.128	0	-0.257909115
PTEN	ARHGEF15	0.128	0	-0.008613401
PTEN	LOC150786	0.128	0	-0.335308243
PTEN	LOC253039	0.128	0	0.11914238
PTEN	SLC38A6	0.128	0	0.105696014
PTEN	ECSCR	0.128	0	-0.000473224
PTEN	SGCG	0.128	0	-0.019407564
PTEN	AKR7L	0.128	0	-0.228676897
PTEN	TSPAN18	0.128	0	-0.005910254
PTEN	SRRM1	0.128	0	-0.018141323
PTEN	B3GNT9	0.128	0	-0.173471641
PTEN	NRBF2	0.129	0	0.008228737
PTEN	CDS2	0.129	0	-0.078029868
PTEN	SOBP	0.129	0	-0.526613431
PTEN	CASP6	0.129	0	-0.00946405
PTEN	MYOZ3	0.129	0	-0.343152416
PTEN	ZKSCAN4	0.129	0	-0.020232744
PTEN	NBPF4	0.129	0	-0.014421633
PTEN	WDR33	0.129	0	-0.011996031
PTEN	USP22	0.129	0	0.077973756
PTEN	CGNL1	0.129	0	-0.006999207
PTEN	LOC148145	0.129	0	-0.067286319

PTEN	USP9Y	0.129	0	-0.082433397
PTEN	SMAP2	0.129	0	0.092084658
PTEN	IPPK	0.129	0	0.004134294
PTEN	SLC44A3	0.129	0	0.117873772
PTEN	ZNF155	0.129	0	-0.03383387
PTEN	ZNF302	0.129	0	-0.040340838
PTEN	TRIM61	0.129	0	-0.1959402
PTEN	PRKACB	0.129	0	-0.204722221
PTEN	PQLC3	0.129	0	-0.035509608
PTEN	LOC284749	0.129	0	-0.02047184
PTEN	SOS1	0.129	0	0.06547472
PTEN	SNX24	0.129	0	-0.150571087
PTEN	AHCTF1	0.129	0	-0.038872635
PTEN	ERMN	0.129	0	-0.029934301
PTEN	FAM174A	0.129	0	-0.057429022
PTEN	C2orf43	0.129	0	-0.197395844
PTEN	MARK3	0.129	0	0.09111627
PTEN	STAT6	0.129	0	0.009417384
PTEN	PAX2	0.129	0	-0.063961376
PTEN	PRKAB1	0.129	0	-0.055784806
PTEN	IGFBP7	0.129	0	0.0277184
PTEN	CNNM4	0.129	0	-0.044946669
PTEN	GNB1	0.129	0	0.101304372
PTEN	C9orf144B	0.129	0	-0.03388469
PTEN	SERP2	0.13	0	-0.000496193
PTEN	C10orf57	0.13	0	-0.014161713
PTEN	CREB5	0.13	0	-0.17523072
PTEN	SIM1	0.13	0	-0.340379836
PTEN	PRKAR2B	0.13	0	-0.189317081
PTEN	HPSE2	0.13	0	-0.024210893
PTEN	TTTY9B	0.13	0	-0.074521198
PTEN	GABRA4	0.13	0	-0.000804833
PTEN	EHMT1	0.13	0	-0.129780824
PTEN	PROX1	0.13	0	-0.330337781
PTEN	ELOVL2	0.13	0	-0.161037276
PTEN	ZNF527	0.13	0	-0.770289132
PTEN	GATAD1	0.13	0	-0.061097787
PTEN	MOAP1	0.13	0	-0.400468052
PTEN	ZNF208	0.13	0	0.118722947
PTEN	RASL11A	0.13	0	-0.291365418
PTEN	RMST	0.13	0	-0.510495294

PTEN	CRHBP	0.13	0	-0.385694382
PTEN	DCAF7	0.13	0	-0.154775442
PTEN	CAMK4	0.13	0	-0.006209945
PTEN	ERGIC2	0.13	0	-0.184899561
PTEN	GJB2	0.13	0	-0.036104918
PTEN	LOC100272216	0.13	0	-0.003295888
PTEN	C9orf116	0.13	0	0.018397135
PTEN	ALX4	0.13	0	-0.013018358
PTEN	MYCBP	0.13	0	-0.12480761
PTEN	ESAM	0.131	0	-0.02188703
PTEN	LHCGR	0.131	0	-0.545397342
PTEN	WWTR1	0.131	0	-0.021999397
PTEN	RIMKLA	0.131	0	-4.53E-05
PTEN	CALN1	0.131	0	-0.000111006
PTEN	ANXA5	0.131	0	0.0061609
PTEN	TMEM168	0.131	0	0.086215986
PTEN	KCNAB1	0.131	0	-0.554730607
PTEN	BASP1	0.131	0	-0.270456772
PTEN	TP53INP2	0.131	0	0.130777376
PTEN	ITIH5L	0.131	0	0.028508779
PTEN	IPW	0.131	0	-0.241119528
PTEN	SUOX	0.131	0	0.001510141
PTEN	XYLT1	0.131	0	-0.007911486
PTEN	C12orf74	0.131	0	-0.018331542
PTEN	PCDHGB4	0.131	0	-0.001639405
PTEN	UBIAD1	0.131	0	-0.043338034
PTEN	ZBTB39	0.131	0	-0.050122516
PTEN	LOC401093	0.131	0	-0.161945274
PTEN	CD36	0.131	0	-0.014260411
PTEN	C7	0.131	0	-0.014325013
PTEN	KRT32	0.131	0	-0.002820229
PTEN	MRPS14	0.131	0	-0.026256758
PTEN	FMO4	0.131	0	-0.021609441
PTEN	SLC7A13	0.131	0	-0.240988135
PTEN	ZNF238	0.132	0	0.008439002
PTEN	ZNF107	0.132	0	-0.420848387
PTEN	BAG3	0.132	0	-0.144737946
PTEN	MGAM	0.132	0	-0.057725404
PTEN	ARPP21	0.132	0	-0.531085239
PTEN	COX11	0.132	0	0.119911313
PTEN	TMED10P1	0.132	0	-0.003851404

PTEN	HADHA	0.132	0	0.01134279
PTEN	TNNT3	0.132	0	-6.96E-07
PTEN	AS3MT	0.132	0	-0.192169687
PTEN	GTF2IRD2	0.132	0	-0.226483461
PTEN	KCNMB4	0.132	0	-0.268368394
PTEN	ZFP2	0.132	0	-0.044796305
PTEN	SLC9A9	0.132	0	-0.177283549
PTEN	SPTBN1	0.132	0	-0.097667067
PTEN	IFIT2	0.132	0	-0.135721002
PTEN	TLR6	0.132	0	-0.016280097
PTEN	ALOX5	0.132	0	0.001623377
PTEN	MEG8	0.132	0	-0.150233907
PTEN	JHDM1D	0.132	0	-0.375986937
PTEN	TGFB1I1	0.132	0	-0.00155559
PTEN	FLJ39653	0.132	0	-0.346160201
PTEN	OR2L13	0.132	0	-0.257521191
PTEN	CLEC14A	0.132	0	-0.019281965
PTEN	ANXA13	0.132	0	-0.387856764
PTEN	PHKB	0.132	0	-0.019081103
PTEN	RBM11	0.132	0	-0.036880569
PTEN	FOXN1	0.132	0	-0.00092508
PTEN	SCN1B	0.132	0	-0.145237694
PTEN	LPAR5	0.132	0	-0.037343241
PTEN	PPM1B	0.132	0	-0.117531414
PTEN	PLEKHA7	0.132	0	-0.09901252
PTEN	ZNF582	0.132	0	-0.334491477
PTEN	CNOT1	0.133	0	0.096924534
PTEN	IFT81	0.133	0	-0.575766147
PTEN	HEATR7B2	0.133	0	-2.13E-07
PTEN	RPS4X	0.133	0	0.000762109
PTEN	PDE4B	0.133	0	-0.098545656
PTEN	ALPK3	0.133	0	-0.028080774
PTEN	MICA	0.133	0	-0.064431746
PTEN	NFATC4	0.133	0	-0.022889631
PTEN	C2orf27A	0.133	0	-0.306052932
PTEN	SNORD12C	0.133	0	-0.002209707
PTEN	FAM129A	0.133	0	0.197615933
PTEN	ACER3	0.133	0	-0.322560835
PTEN	MAPKSP1	0.133	0	-0.064869875
PTEN	CYP2C8	0.133	0	-0.141728721
PTEN	PPIG	0.133	0	-0.381031088

PTEN	PRDM4	0.133	0	-0.049504383
PTEN	CASC3	0.133	0	0.00851008
PTEN	NPR1	0.133	0	0.001436499
PTEN	FAH	0.133	0	3.21E-06
PTEN	FBLN7	0.133	0	-0.175283547
PTEN	KDM4B	0.133	0	0.074600854
PTEN	C3orf34	0.133	0	-0.465441683
PTEN	STRADB	0.133	0	-0.3504154
PTEN	DET1	0.133	0	0.106494573
PTEN	ZFP161	0.133	0	0.000614222
PTEN	MGLL	0.133	0	-0.151133527
PTEN	PAX1	0.134	0	-0.014079693
PTEN	REEP5	0.134	0	-0.095152711
PTEN	ATP1A2	0.134	0	-0.160837964
PTEN	HRASLS5	0.134	0	-0.250342672
PTEN	UBA3	0.134	0	-0.118787838
PTEN	TOMM20	0.134	0	0.530397699
PTEN	ZNF596	0.134	0	-0.162410045
PTEN	SLC4A4	0.134	0	-0.637095135
PTEN	LRRC3B	0.134	0	-0.025621077
PTEN	ABHD4	0.134	0	-0.002215914
PTEN	TMEM185B	0.134	0	0.056298504
PTEN	CCIN	0.134	0	-0.004814401
PTEN	TMEM39A	0.134	0	-0.207125553
PTEN	EYA1	0.134	0	-0.542252259
PTEN	REEP6	0.134	0	0.0025189
PTEN	PRAMEF1	0.134	0	-0.001602592
PTEN	M6PR	0.134	0	0.13707174
PTEN	ICMT	0.134	0	0.011294115
PTEN	EHD3	0.134	0	-0.20567258
PTEN	MEGF10	0.134	0	-0.337174444
PTEN	C17orf68	0.134	0	-0.038544231
PTEN	CNTN6	0.134	0	-0.189857933
PTEN	PCDHA12	0.135	0	-0.397503489
PTEN	IQCK	0.135	0	0.02539722
PTEN	LOC647121	0.135	0	-0.218705881
PTEN	FGFR2	0.135	0	-0.193949727
PTEN	CPSF6	0.135	0	0.072620932
PTEN	ILK	0.135	0	-0.001431771
PTEN	TRIM37	0.135	0	-0.185513058
PTEN	ZKSCAN5	0.135	0	-0.116389576

PTEN	ANXA11	0.135	0	0.01209136
PTEN	IL1RAPL2	0.135	0	-0.08157844
PTEN	RBBP9	0.135	0	-0.242432654
PTEN	NKAIN3	0.135	0	-0.216102072
PTEN	EDARADD	0.135	0	-0.289553097
PTEN	MMP28	0.135	0	-0.19807528
PTEN	C18orf34	0.135	0	-0.284448822
PTEN	PCDH10	0.135	0	-0.249412142
PTEN	VDR	0.135	0	-0.045999167
PTEN	PPEF1	0.135	0	-0.172562062
PTEN	NUP54	0.135	0	-0.013272598
PTEN	POGZ	0.135	0	-0.166488679
PTEN	RERG	0.135	0	-0.017250041
PTEN	TIMM9	0.135	0	-0.002746963
PTEN	RNF20	0.135	0	0.033501396
PTEN	PCDHAC2	0.135	0	-0.402664473
PTEN	15-Sep	0.135	0	0.187816643
PTEN	RAB11FIP5	0.135	0	-0.059456864
PTEN	FBLN2	0.135	0	0.110303102
PTEN	LOC642826	0.135	0	-0.121623598
PTEN	PRUNE	0.135	0	-0.040151566
PTEN	DCUN1D4	0.135	0	-0.218078105
PTEN	GATAD2B	0.135	0	-0.110216521
PTEN	FKSG29	0.135	0	-0.096612326
PTEN	NUDT11	0.135	0	-0.359048116
PTEN	KIAA0556	0.135	0	-0.02201373
PTEN	CCDC103	0.135	0	-0.001880482
PTEN	SPATS2L	0.135	0	-0.099480598
PTEN	GSTM1	0.135	0	0.003480085
PTEN	FAM182A	0.135	0	-0.035396361
PTEN	TRIM68	0.135	0	-0.194571386
PTEN	TSN	0.136	0	0.032574449
PTEN	ZNF320	0.136	0	-0.288260062
PTEN	AHCYL2	0.136	0	-0.185483181
PTEN	CDH17	0.136	0	-0.302322523
PTEN	KIF5A	0.136	0	-0.114743905
PTEN	PKIB	0.136	0	-0.068743346
PTEN	CYB5R4	0.136	0	-0.120610628
PTEN	MAP3K9	0.136	0	-0.488140485
PTEN	SESTD1	0.136	0	-0.12751267
PTEN	GGNBP2	0.136	0	-0.038903512

PTEN	C9orf47	0.136	0	-0.115830823
PTEN	HSD17B6	0.136	0	-0.16880131
PTEN	UTS2	0.136	0	-0.004470378
PTEN	PHF6	0.136	0	-0.198752075
PTEN	PCDHA4	0.136	0	-0.395802761
PTEN	AGBL4	0.136	0	0.013261476
PTEN	C7orf41	0.136	0	-0.08703603
PTEN	UBE2D1	0.136	0	-0.396364465
PTEN	DYRK1A	0.136	0	0.280152157
PTEN	KPNA4	0.136	0	-0.370397987
PTEN	LINGO2	0.136	0	-0.271204881
PTEN	TBC1D2	0.136	0	-0.10030166
PTEN	ARR3	0.136	0	-1.56E-05
PTEN	TBC1D8	0.136	0	-0.125692872
PTEN	WNT2	0.136	0	-0.161656191
PTEN	ZNF468	0.136	0	-0.160549325
PTEN	HSD17B7P2	0.136	0	-0.279960631
PTEN	ACSL3	0.136	0	0.037401425
PTEN	TSPYL2	0.136	0	-0.000934265
PTEN	NAPG	0.136	0	-0.052387316
PTEN	C2orf40	0.136	0	-0.014541136
PTEN	WDR5B	0.137	0	-0.168357045
PTEN	GMCL1L	0.137	0	-0.052615353
PTEN	CBWD5	0.137	0	-0.225549286
PTEN	TMEM48	0.137	0	-0.119562754
PTEN	VKORC1L1	0.137	0	0.073099795
PTEN	PLA2G10	0.137	0	-0.000341541
PTEN	CMAH	0.137	0	-0.198746803
PTEN	FRAS1	0.137	0	-0.161472546
PTEN	OLR1	0.137	0	-0.2273991
PTEN	MSX2	0.137	0	-0.045408094
PTEN	CPB1	0.137	0	0.000773358
PTEN	FKBP3	0.137	0	0.231325916
PTEN	SCN4A	0.137	0	-0.041531226
PTEN	SNORD2	0.137	0	-0.008633924
PTEN	TCTEX1D4	0.137	0	-0.000113009
PTEN	HS3ST2	0.137	0	-0.176202898
PTEN	GCLC	0.137	0	-0.009055227
PTEN	FAM154B	0.137	0	-0.271506917
PTEN	ARMC2	0.137	0	-0.089977514
PTEN	C2orf77	0.137	0	-0.037942588

PTEN	TNFRSF10A	0.137	0	-0.02115404
PTEN	EMR1	0.137	0	-0.136913566
PTEN	CYP2A13	0.137	0	-0.152736709
PTEN	KIAA0562	0.137	0	-0.105527448
PTEN	ARFIP2	0.137	0	0.069091434
PTEN	C16orf70	0.137	0	-0.382833254
PTEN	PSAP	0.137	0	0.11851828
PTEN	MALT1	0.138	0	-0.086203779
PTEN	C12orf66	0.138	0	-0.455683755
PTEN	ZC3H10	0.138	0	-0.15503589
PTEN	TPM4	0.138	0	0.038676322
PTEN	PPHLN1	0.138	0	-0.302498251
PTEN	LOC642587	0.138	0	-0.004005966
PTEN	ZMYND8	0.138	0	-0.1679668
PTEN	TAF13	0.138	0	-0.158416631
PTEN	VANGL1	0.138	0	-0.144383636
PTEN	PANX1	0.138	0	-0.453986814
PTEN	RUFY3	0.138	0	-0.275109082
PTEN	ADH1B	0.138	0	-0.131970572
PTEN	FSD1L	0.138	0	-0.293605835
PTEN	CALCR	0.138	0	-0.547927552
PTEN	C12orf59	0.138	0	-0.356961949
PTEN	CALU	0.138	0	0.092041529
PTEN	ZNF700	0.138	0	-0.004496347
PTEN	C15orf34	0.138	0	-0.233345934
PTEN	HAR1A	0.138	0	-0.005256128
PTEN	ULK2	0.138	0	-0.020253709
PTEN	LOC100130522	0.138	0	-0.177210004
PTEN	ARL17B	0.138	0	-0.112419241
PTEN	SEMA3B	0.138	0	-0.001741935
PTEN	KIAA1467	0.138	0	-0.016267405
PTEN	CMTM6	0.138	0	-0.073456184
PTEN	CST2	0.138	0	-0.002462569
PTEN	SMARCD1	0.139	0	0.021417275
PTEN	GLYAT	0.139	0	-0.334293014
PTEN	MBOAT2	0.139	0	-0.097850322
PTEN	ZNF217	0.139	0	-0.113199232
PTEN	TAF9	0.139	0	-0.342053501
PTEN	PENK	0.139	0	-0.355368331
PTEN	SYCE1	0.139	0	-0.032609895
PTEN	ITIH5	0.139	0	-0.204356827

PTEN	TMCC3	0.139	0	-0.286940761
PTEN	MORN4	0.139	0	-0.030771412
PTEN	MYH2	0.139	0	-0.010298258
PTEN	KHNYN	0.139	0	0.121004905
PTEN	EPGN	0.139	0	-0.067445726
PTEN	FAM22A	0.139	0	2.86E-07
PTEN	GPIHBP1	0.139	0	-0.001606574
PTEN	CHTF8	0.139	0	-0.088420712
PTEN	MAPKAPK2	0.139	0	-0.000360971
PTEN	COL16A1	0.139	0	-0.230276429
PTEN	C6orf89	0.139	0	0.019258379
PTEN	LIMS3	0.139	0	-0.02966765
PTEN	KIAA0317	0.139	0	-0.089627345
PTEN	XPNPEP1	0.139	0	-0.01079919
PTEN	PARP14	0.139	0	-0.514066666
PTEN	NLN	0.139	0	-0.173749846
PTEN	TTN	0.139	0	-0.033875455
PTEN	C17orf48	0.139	0	-0.005217845
PTEN	SLCO4C1	0.139	0	-0.431072088
PTEN	PPA2	0.139	0	-0.067538687
PTEN	TCEANC	0.139	0	-0.149840826
PTEN	CRIP1	0.139	0	6.82E-05
PTEN	TBC1D5	0.139	0	-0.08679195
PTEN	ARFGEF2	0.139	0	-0.040829016
PTEN	CD55	0.139	0	0.079978656
PTEN	PCDHGA3	0.139	0	-0.001642488
PTEN	KIAA1407	0.139	0	-0.177244842
PTEN	GFPT2	0.139	0	0.129301438
PTEN	PRR15	0.14	0	-0.075639232
PTEN	PRPF39	0.14	0	-0.265440127
PTEN	PTAFR	0.14	0	-0.008723972
PTEN	TRPS1	0.14	0	0.436381309
PTEN	PCDHGA7	0.14	0	-0.022853995
PTEN	GPR137B	0.14	0	-0.127591913
PTEN	CCDC87	0.14	0	-0.145715546
PTEN	FMO9P	0.14	0	-0.427033165
PTEN	KIAA1024	0.14	0	-0.242283436
PTEN	SPEF1	0.14	0	-0.00602353
PTEN	FBXL4	0.14	0	-0.031564184
PTEN	C1QL3	0.14	0	-0.207407735
PTEN	COMMD8	0.14	0	-0.034998887

PTEN	PPM1D	0.14	0	-0.331589679
PTEN	ZNF235	0.14	0	-0.178337043
PTEN	SYF2	0.14	0	-0.122022978
PTEN	FGF5	0.14	0	-0.513277283
PTEN	CYP3A4	0.14	0	-0.189641403
PTEN	ZNF330	0.14	0	-0.155254851
PTEN	PNPLA4	0.14	0	-0.181753827
PTEN	FAM117B	0.14	0	-0.222714816
PTEN	CLEC16A	0.14	0	-0.004249965
PTEN	C20orf197	0.14	0	-0.071704443
PTEN	FAM106C	0.14	0	-0.16016324
PTEN	RASSF3	0.14	0	-0.168559204
PTEN	LOC729020	0.14	0	-0.202344448
PTEN	CAMK2D	0.14	0	-0.022818937
PTEN	EPDR1	0.14	0	-0.005190937
PTEN	ZNF606	0.14	0	-0.172674117
PTEN	ACVR2B	0.141	0	-0.010733483
PTEN	BCAT1	0.141	0	-0.059352499
PTEN	ZNF674	0.141	0	-0.388377035
PTEN	SNORD45C	0.141	0	-0.191443414
PTEN	ITGA6	0.141	0	-0.086701345
PTEN	C1D	0.141	0	-0.207615977
PTEN	TCTEX1D1	0.141	0	-0.121258851
PTEN	TCTN1	0.141	0	-0.03519799
PTEN	IVD	0.141	0	0.014973325
PTEN	ZNF433	0.141	0	-0.000268534
PTEN	CBL	0.141	0	-0.006019255
PTEN	KIAA0391	0.141	0	0.043643473
PTEN	ALG13	0.141	0	-0.041078206
PTEN	C14orf101	0.141	0	-0.115717808
PTEN	WWP1	0.141	0	0.175830718
PTEN	UHRF1BP1	0.141	0	-0.210039572
PTEN	LGALS3	0.141	0	-0.236777492
PTEN	PAPOLB	0.141	0	-0.450856944
PTEN	SUCNR1	0.141	0	-0.15344181
PTEN	HOXA5	0.141	0	-0.190442614
PTEN	CDV3	0.141	0	-0.138679961
PTEN	AHI1	0.141	0	-0.232798692
PTEN	SNX7	0.141	0	-0.008852735
PTEN	SHISA9	0.141	0	-0.382912452
PTEN	TTC38	0.142	0	-0.210134068

PTEN	CCNO	0.142	0	-2.14E-07
PTEN	CXorf38	0.142	0	-0.038511913
PTEN	GOLGA6D	0.142	0	-0.018164699
PTEN	SCTR	0.142	0	-2.51E-06
PTEN	RBM23	0.142	0	-0.019607264
PTEN	PLS3	0.142	0	0.213622619
PTEN	DPP6	0.142	0	-0.020855186
PTEN	MPV17L	0.142	0	-0.007685666
PTEN	ORAI3	0.142	0	-0.288437265
PTEN	GPR162	0.142	0	-0.000147661
PTEN	SMPDL3A	0.142	0	-0.040476043
PTEN	CYP4B1	0.142	0	0.065293099
PTEN	C17orf76	0.142	0	-0.044500623
PTEN	KLK3	0.142	0	-0.000918624
PTEN	SCGB2A1	0.142	0	-0.078785958
PTEN	CREG2	0.142	0	-0.071147775
PTEN	TAF7	0.142	0	-0.11962363
PTEN	ZDHHC2	0.142	0	-0.174117113
PTEN	RRM2B	0.142	0	0.141706609
PTEN	ZMYND11	0.142	0	0.242306146
PTEN	ZNF813	0.142	0	-0.187736294
PTEN	ABHD15	0.142	0	-0.25683659
PTEN	SPOCD1	0.142	0	-0.000280334
PTEN	CLRN1	0.142	0	-0.219726628
PTEN	ABHD5	0.142	0	-0.079820515
PTEN	PCDHB16	0.142	0	-0.494782501
PTEN	UBQLN1	0.142	0	-0.031777574
PTEN	PHYHD1	0.142	0	-0.159119197
PTEN	C8orf48	0.142	0	-0.252377377
PTEN	MEGF6	0.142	0	-0.026149279
PTEN	AKR7A3	0.142	0	-0.006555162
PTEN	USPL1	0.143	0	-0.109919197
PTEN	PURB	0.143	0	-0.036451813
PTEN	NRG3	0.143	0	-0.539133673
PTEN	CASQ2	0.143	0	-0.217315385
PTEN	SCIN	0.143	0	-0.037488983
PTEN	KLF13	0.143	0	-0.004815256
PTEN	C4orf33	0.143	0	-0.267648844
PTEN	MBTPS1	0.143	0	0.020349866
PTEN	EFHC1	0.143	0	-0.291092361
PTEN	FUT2	0.143	0	-0.002552433

PTEN	GRK5	0.143	0	-0.188458753
PTEN	USP44	0.143	0	-0.537405725
PTEN	MACROD2	0.143	0	-0.664787005
PTEN	MRPS30	0.143	0	0.047679132
PTEN	PHLDB1	0.143	0	-0.012461205
PTEN	ZNF415	0.143	0	-0.010968311
PTEN	JAKMIP3	0.143	0	0.063066985
PTEN	FAM119B	0.143	0	0.006446594
PTEN	GLYATL1	0.143	0	-0.289648873
PTEN	LOC100132111	0.143	0	-0.083758364
PTEN	ZNF713	0.143	0	-0.005664927
PTEN	SS18	0.143	0	-0.082457023
PTEN	IMPG2	0.143	0	-0.205355572
PTEN	IGFL3	0.143	0	-0.003209831
PTEN	DACH2	0.144	0	-0.230933113
PTEN	PGAM4	0.144	0	0.098717181
PTEN	ASTN1	0.144	0	-0.485426338
PTEN	LETMD1	0.144	0	-0.156582326
PTEN	KIAA1009	0.144	0	-0.058206162
PTEN	CYTSB	0.144	0	-0.005997072
PTEN	SLC16A2	0.144	0	-0.242280029
PTEN	CEP192	0.144	0	-0.168961747
PTEN	ZNF460	0.144	0	-0.118647536
PTEN	PARD6G	0.144	0	-0.098541577
PTEN	DDB2	0.144	0	-0.001483397
PTEN	SLC6A1	0.144	0	-0.267537984
PTEN	INTS12	0.144	0	-0.14200175
PTEN	TRIM14	0.144	0	0.044367351
PTEN	IL1RAPL1	0.144	0	-0.163525983
PTEN	USP4	0.144	0	-0.047229712
PTEN	PCDHGA11	0.144	0	-0.043644037
PTEN	SYT6	0.144	0	-0.193711646
PTEN	TAL2	0.144	0	-0.01234274
PTEN	LARS	0.144	0	-0.145820168
PTEN	SLC47A2	0.144	0	-0.224655315
PTEN	RD3	0.144	0	-0.342001114
PTEN	TMEM116	0.144	0	-0.088220185
PTEN	C9orf98	0.144	0	-7.11E-05
PTEN	FSTL3	0.144	0	-0.000473871
PTEN	DYNC2LI1	0.144	0	-0.358659746
PTEN	STARD4	0.144	0	-0.188485446

PTEN	HIGD1B	0.144	0	-1.09E-05
PTEN	TFDP3	0.144	0	-0.180354361
PTEN	ST5	0.144	0	-0.135170683
PTEN	PLCE1	0.144	0	-0.023403937
PTEN	PGRMC2	0.144	0	0.030201315
PTEN	MMP21	0.144	0	-0.403618354
PTEN	CRYZ	0.145	0	-0.013883371
PTEN	CYP46A1	0.145	0	0.0008784
PTEN	GPR135	0.145	0	-0.011780441
PTEN	ZNF592	0.145	0	-0.20513831
PTEN	MORC4	0.145	0	-0.060154892
PTEN	SLC25A36	0.145	0	0.146376892
PTEN	INTS7	0.145	0	-0.110490995
PTEN	UTS2D	0.145	0	-0.049115389
PTEN	SCARF2	0.145	0	-1.48E-06
PTEN	LRIG3	0.145	0	-0.302535518
PTEN	ZIK1	0.145	0	0.105635282
PTEN	GNPTAB	0.145	0	-0.20728127
PTEN	IP6K1	0.145	0	0.021071771
PTEN	GOLGA2B	0.145	0	-0.118329402
PTEN	GLB1L	0.145	0	-0.000797857
PTEN	SRP72	0.145	0	0.064438985
PTEN	ZNF521	0.145	0	0.051824088
PTEN	CCL14	0.145	0	-1.20E-05
PTEN	PALM2-AKAP2	0.145	0	-0.242490883
PTEN	TLE4	0.145	0	-0.523485349
PTEN	MBIP	0.145	0	-0.302555702
PTEN	SLCO2A1	0.145	0	-0.100520692
PTEN	FBXW4	0.145	0	-0.005110783
PTEN	HOOK1	0.145	0	-0.50103617
PTEN	LRRC46	0.145	0	0.000435418
PTEN	UBA6	0.145	0	-0.003138939
PTEN	KCNC2	0.145	0	-0.337133469
PTEN	MPZL3	0.145	0	-0.131581337
PTEN	ZNF79	0.145	0	-4.31E-08
PTEN	AP3S1	0.145	0	0.138483912
PTEN	FAM126A	0.145	0	-0.248339126
PTEN	C12orf50	0.145	0	-0.143091986
PTEN	RNF213	0.145	0	0.338591303
PTEN	LARP1B	0.145	0	-0.089662221
PTEN	RHOBTB3	0.146	0	-0.094624646

PTEN	XPO7	0.146	0	-0.042396261
PTEN	BTBD10	0.146	0	0.033833743
PTEN	SH3RF2	0.146	0	-5.67E-05
PTEN	NAB1	0.146	0	-0.206343165
PTEN	SYNJ2	0.146	0	-0.254348554
PTEN	ZCWPW2	0.146	0	-0.175799203
PTEN	GP2	0.146	0	-0.009172425
PTEN	ZNF569	0.146	0	-0.190925365
PTEN	EVL	0.146	0	-4.17E-05
PTEN	ZNF664	0.146	0	-0.116617623
PTEN	PRICKLE1	0.146	0	0.080671435
PTEN	DTX3	0.146	0	-0.003774616
PTEN	HOXB3	0.146	0	-0.222049984
PTEN	MECP2	0.146	0	-0.103402887
PTEN	C1orf95	0.146	0	-0.00593218
PTEN	CFHR1	0.146	0	-0.129345485
PTEN	TM7SF3	0.146	0	0.001380571
PTEN	ZKSCAN3	0.146	0	-0.182964965
PTEN	ARSD	0.146	0	0.375241454
PTEN	C3orf23	0.146	0	-0.202068186
PTEN	RNF148	0.146	0	-0.014063739
PTEN	SPRED3	0.146	0	-1.49E-06
PTEN	BACH2	0.146	0	-0.263865985
PTEN	OSBPL10	0.146	0	-0.22838119
PTEN	ZNF506	0.146	0	-0.139814985
PTEN	FXR1	0.146	0	0.383611004
PTEN	SLC2A9	0.146	0	-0.008779504
PTEN	PLXNA2	0.146	0	-0.281112284
PTEN	HPX	0.147	0	-0.001708825
PTEN	NUP155	0.147	0	-0.01639101
PTEN	GPR133	0.147	0	-0.027099049
PTEN	ATXN7L1	0.147	0	-0.369656739
PTEN	CDH18	0.147	0	-0.009696941
PTEN	LNX1	0.147	0	-0.133540596
PTEN	C15orf59	0.147	0	-0.006733998
PTEN	LOC387647	0.147	0	-0.225003403
PTEN	PCDHGA5	0.147	0	-0.001655131
PTEN	PAR-SN	0.147	0	-0.267707155
PTEN	MARVELD1	0.147	0	-0.013381104
PTEN	SENP6	0.147	0	-0.033708843
PTEN	C5orf28	0.147	0	-0.369351203

PTEN	ANKRD57	0.147	0	-0.213399923
PTEN	FAM189A1	0.147	0	-0.020906412
PTEN	FAM154A	0.147	0	-0.185029579
PTEN	NEFM	0.147	0	-0.420231588
PTEN	RARS	0.147	0	-0.001284534
PTEN	ADAP2	0.147	0	-0.080613577
PTEN	TCP11L1	0.147	0	-0.049208035
PTEN	SLCO2B1	0.147	0	-0.142496888
PTEN	OPRM1	0.147	0	-0.545099014
PTEN	LPIN2	0.147	0	-0.157617539
PTEN	BDKRB2	0.147	0	0.026385207
PTEN	CASP8	0.147	0	-0.188121069
PTEN	CACNA2D2	0.147	0	-0.245231822
PTEN	ALG14	0.147	0	-0.159988969
PTEN	JDP2	0.147	0	0.001788574
PTEN	GOLGA9P	0.147	0	-0.01244295
PTEN	NUDT21	0.147	0	0.030478168
PTEN	ACSM5	0.147	0	-0.001578111
PTEN	PPP2R3A	0.147	0	-0.392601638
PTEN	ZNF317	0.147	0	-0.364275076
PTEN	CILP2	0.147	0	-0.013937831
PTEN	ZZEF1	0.147	0	0.003504952
PTEN	DNAJC14	0.147	0	0.004033183
PTEN	ETV5	0.147	0	-0.058205671
PTEN	ZNF85	0.147	0	-0.261431311
PTEN	LRRC50	0.147	0	-7.02E-05
PTEN	S100Z	0.147	0	-0.024500044
PTEN	LACE1	0.147	0	-0.260228603
PTEN	PALB2	0.147	0	-0.007594815
PTEN	BRD8	0.148	0	-0.008580522
PTEN	SEMA3G	0.148	0	-0.000554579
PTEN	IFI16	0.148	0	-0.004752468
PTEN	CACNA2D4	0.148	0	-0.159681605
PTEN	GPR155	0.148	0	-0.092151845
PTEN	KCTD2	0.148	0	-0.021704517
PTEN	ABCB1	0.148	0	-0.352987331
PTEN	PTPN3	0.148	0	-0.162565385
PTEN	SPATA9	0.148	0	-0.309172964
PTEN	BECN1	0.148	0	-0.092072091
PTEN	ZFP1	0.148	0	-0.00244407
PTEN	APBB1	0.148	0	-0.030057355

PTEN	TIRAP	0.148	0	-0.304328478
PTEN	FBXW8	0.148	0	0.144705917
PTEN	USP30	0.148	0	-0.172061902
PTEN	MFN2	0.148	0	-0.151402031
PTEN	CXADR	0.148	0	0.012475468
PTEN	SRP54	0.148	0	-0.087700624
PTEN	PTPRO	0.148	0	-0.278474306
PTEN	ABCA11P	0.148	0	-0.288627132
PTEN	PARP9	0.148	0	0.016641526
PTEN	RGS7	0.148	0	-0.574522676
PTEN	C6orf165	0.148	0	-0.107270415
PTEN	MIPEP	0.148	0	-0.169082387
PTEN	PDE2A	0.148	0	-0.2803673
PTEN	LOC494141	0.148	0	-0.575098681
PTEN	SLC46A3	0.148	0	-0.008340645
PTEN	GRM8	0.148	0	-0.656870485
PTEN	C3AR1	0.148	0	-0.025748334
PTEN	TAB2	0.148	0	-0.036978009
PTEN	NLRP3	0.148	0	-0.135580176
PTEN	UBA5	0.148	0	-0.300777948
PTEN	KIAA0090	0.148	0	-0.075353346
PTEN	CAPSL	0.149	0	-0.130642533
PTEN	DSG4	0.149	0	-0.05542998
PTEN	LAMP2	0.149	0	0.386864327
PTEN	SEC22C	0.149	0	-0.054396315
PTEN	FAM133A	0.149	0	-0.221127693
PTEN	ITIH3	0.149	0	-0.13442753
PTEN	NSUN4	0.149	0	-0.089446445
PTEN	ADAM21	0.149	0	-0.170947827
PTEN	BCL8	0.149	0	-0.000618856
PTEN	ZNF626	0.149	0	-0.511704318
PTEN	TNFRSF11B	0.149	0	-0.13274983
PTEN	CAMK2N1	0.149	0	0.058457018
PTEN	EPHX2	0.149	0	-0.074272179
PTEN	STXBP1	0.149	0	-0.391881477
PTEN	P2RY4	0.149	0	-0.002700858
PTEN	OLA1	0.149	0	-0.144342384
PTEN	EHD4	0.149	0	-5.93E-05
PTEN	RAB11A	0.149	0	0.137499212
PTEN	HOXB8	0.149	0	-0.042139893
PTEN	NXPH1	0.149	0	-0.08254228

PTEN	TPST2	0.149	0	-0.173887937
PTEN	ANXA9	0.149	0	-0.002166713
PTEN	SNURF	0.149	0	-0.244565426
PTEN	C9orf6	0.149	0	-0.023193588
PTEN	MAP4	0.149	0	0.105794988
PTEN	RHOB	0.149	0	0.181509262
PTEN	INTS9	0.149	0	-0.071746065
PTEN	PCDHGB5	0.149	0	-0.001566331
PTEN	TMEM35	0.149	0	-0.256934372
PTEN	IGSF3	0.149	0	-0.187767296
PTEN	C10orf81	0.149	0	-0.026778755
PTEN	GORAB	0.149	0	-0.298920992
PTEN	ADH1C	0.149	0	-0.226061657
PTEN	BCL6	0.149	0	-0.13733032
PTEN	SIAH2	0.149	0	0.000239339
PTEN	COPZ1	0.149	0	0.033337733
PTEN	FAM190A	0.149	0	-0.2780446
PTEN	CCDC83	0.149	0	-0.076464279
PTEN	ZNF594	0.149	0	-0.150111904
PTEN	C2orf49	0.149	0	-0.002661834
PTEN	KCNQ3	0.149	0	-0.033319836
PTEN	FITM2	0.15	0	-1.79E-05
PTEN	EIF2AK2	0.15	0	-0.183027904
PTEN	NKTR	0.15	0	-0.083890626
PTEN	IL13RA2	0.15	0	-0.17837103
PTEN	DDX31	0.15	0	-0.008874954
PTEN	SCN2A	0.15	0	-0.340543132
PTEN	DRAM2	0.15	0	-0.054200292
PTEN	LOC143188	0.15	0	-0.30660415
PTEN	FBXO16	0.15	0	-0.010274563
PTEN	WDR25	0.15	0	-5.53E-05
PTEN	KIAA1147	0.15	0	0.163736862
PTEN	CLDN20	0.15	0	-0.032398658
PTEN	CRBN	0.15	0	-0.143580657
PTEN	ZPLD1	0.15	0	-0.441991247
PTEN	ERCC5	0.15	0	-2.75E-05
PTEN	XRN2	0.15	0	-0.234887694
PTEN	C16orf71	0.15	0	-0.139646119
PTEN	SUPT16H	0.15	0	-0.06004013
PTEN	PCCA	0.15	0	-0.004343927
PTEN	SMCHD1	0.15	0	-0.258880533

PTEN	AGBL2	0.15	0	-0.18561471
PTEN	MYOM1	0.15	0	-0.023555958
PTEN	LITAF	0.15	0	0.002812648
PTEN	HTRA3	0.15	0	0.166086569
PTEN	DSG2	0.15	0	0.219225895
PTEN	CABP4	0.15	0	-0.149899941
PTEN	SLTM	0.15	0	0.091252017
PTEN	PDE12	0.15	0	-0.204829481
PTEN	BBS2	0.15	0	-0.1333175
PTEN	CLRN3	0.15	0	-0.008795638
PTEN	FAM171B	0.15	0	-0.461925588
PTEN	SUSD1	0.15	0	-0.282396932
PTEN	CARTPT	0.15	0	-0.001800877
PTEN	STX7	0.15	0	-0.472654483
PTEN	TM9SF1	0.15	0	0.010578661
PTEN	SRCAP	0.151	0	-0.014068504
PTEN	BET3L	0.151	0	-0.200212363
PTEN	TPR	0.151	0	0.000186894
PTEN	C18orf25	0.151	0	-0.144467723
PTEN	C2orf73	0.151	0	-0.19622351
PTEN	PPIL3	0.151	0	0.14506096
PTEN	1-Mar	0.151	0	-0.062126431
PTEN	SLC16A12	0.151	0	-0.088014946
PTEN	DCAF8	0.151	0	-0.209194791
PTEN	DIS3	0.151	0	-0.024646769
PTEN	N6AMT1	0.151	0	-0.18761361
PTEN	STAMBPL1	0.151	0	-0.153594309
PTEN	WNK1	0.151	0	0.261578201
PTEN	RPS6KA3	0.151	0	-0.114243321
PTEN	PEX11A	0.151	0	0.006682006
PTEN	ACVR1C	0.151	0	-0.164231539
PTEN	NKD1	0.151	0	-0.16182309
PTEN	LDHAL6A	0.151	0	-0.114311004
PTEN	HNRNPA1	0.151	0	-0.064070932
PTEN	C19orf54	0.151	0	-0.032138046
PTEN	TMPRSS6	0.151	0	-0.007847137
PTEN	PDSS2	0.151	0	-0.235292276
PTEN	PATZ1	0.151	0	0.107681069
PTEN	CST4	0.151	0	-3.61E-05
PTEN	FAAH2	0.151	0	-0.187197846
PTEN	PPP3CC	0.151	0	-0.110601744

PTEN	PHYHIP	0.151	0	-0.157120288
PTEN	CCDC144A	0.151	0	-0.435006131
PTEN	ADAMTS18	0.151	0	-0.498173747
PTEN	EDC3	0.151	0	-0.058308251
PTEN	MIDN	0.151	0	0.001559451
PTEN	C2CD2	0.151	0	-0.091003838
PTEN	MUL1	0.151	0	-0.089474
PTEN	RBM24	0.151	0	-0.372344278
PTEN	GLIPR1L2	0.152	0	-0.031055761
PTEN	PARP3	0.152	0	-0.280042035
PTEN	PCDHGB2	0.152	0	-0.001637111
PTEN	C13orf36	0.152	0	-0.431931456
PTEN	BRAF	0.152	0	-0.038168735
PTEN	ZNF207	0.152	0	-0.015624788
PTEN	VIPAR	0.152	0	-0.01429396
PTEN	KIAA1409	0.152	0	-0.137443385
PTEN	FKBP5	0.152	0	-0.079441311
PTEN	SNAP29	0.152	0	-0.073400057
PTEN	MAGI1	0.152	0	-0.099403674
PTEN	ATPAF1	0.152	0	-0.298354477
PTEN	TNS4	0.152	0	-0.254513965
PTEN	AKR1C3	0.152	0	-0.006431848
PTEN	SLC16A14	0.152	0	-0.523570047
PTEN	C6orf211	0.152	0	-0.107006378
PTEN	LPHN2	0.152	0	-0.366566459
PTEN	MFSD1	0.152	0	0.036737163
PTEN	ZBED1	0.152	0	0.167654153
PTEN	ZNF804A	0.152	0	-0.541472778
PTEN	TXLNG	0.152	0	-0.122197041
PTEN	GUCY1A3	0.152	0	-0.183622632
PTEN	KIAA1671	0.152	0	-0.109565036
PTEN	LTA4H	0.152	0	-0.046134636
PTEN	MANSC1	0.152	0	-0.01275944
PTEN	STOX1	0.152	0	-0.026407248
PTEN	SPAST	0.152	0	0.122010475
PTEN	KCNK2	0.152	0	-0.046027326
PTEN	C17orf103	0.152	0	-0.001705792
PTEN	RGL1	0.152	0	-0.521751444
PTEN	GNPNAT1	0.152	0	-0.054598027
PTEN	PALMD	0.152	0	-0.197663852
PTEN	CPXM1	0.152	0	-9.26E-05

PTEN	MUTED	0.153	0	-0.287149123
PTEN	PTGR2	0.153	0	-0.130103498
PTEN	EFNB2	0.153	0	-0.441250855
PTEN	RAB23	0.153	0	-0.043271384
PTEN	TFPI	0.153	0	-0.129139708
PTEN	SLC45A2	0.153	0	-0.021772788
PTEN	ZC3HAV1L	0.153	0	-0.147791338
PTEN	SNAPC3	0.153	0	-0.093023756
PTEN	CBLL1	0.153	0	-0.165160823
PTEN	CYTSA	0.153	0	-0.004248502
PTEN	CD84	0.153	0	-0.108973732
PTEN	GALR1	0.153	0	-0.17908358
PTEN	SERPINA3	0.153	0	3.50E-06
PTEN	LOC283392	0.153	0	-0.376552631
PTEN	ZNF383	0.153	0	-4.91E-05
PTEN	ATP2A2	0.153	0	0.287726562
PTEN	HEATR1	0.153	0	-0.00079934
PTEN	IGSF9B	0.153	0	-0.001504756
PTEN	PCDHA10	0.153	0	-0.380190311
PTEN	TMEM132C	0.153	0	-0.267436151
PTEN	PPARGC1A	0.153	0	-0.186259705
PTEN	ARL6	0.153	0	-0.225934302
PTEN	LOC284441	0.153	0	-0.17128534
PTEN	HADHB	0.153	0	0.079403736
PTEN	HCN1	0.153	0	-0.554832382
PTEN	PPP1R1C	0.153	0	-0.360079253
PTEN	ZFP36L2	0.153	0	0.10676959
PTEN	NAA16	0.153	0	-0.523981495
PTEN	C19orf21	0.153	0	-0.01022507
PTEN	CYP4F2	0.153	0	-0.011984359
PTEN	SEC22B	0.153	0	-0.062274901
PTEN	MXRA8	0.153	0	0.000113212
PTEN	CNTNAP2	0.153	0	-0.05758035
PTEN	FAM188A	0.153	0	-0.007281382
PTEN	SERPINA6	0.153	0	-0.001948499
PTEN	ANGPT1	0.154	0	-0.232651903
PTEN	INPP5B	0.154	0	-0.251355299
PTEN	ASB3	0.154	0	-0.243661397
PTEN	FAM180A	0.154	0	-0.008272221
PTEN	MAK	0.154	0	-0.419318647
PTEN	FDXACB1	0.154	0	-0.300726811

PTEN	DIS3L	0.154	0	-0.008435808
PTEN	PPP1CC	0.154	0	0.15134416
PTEN	NR1D2	0.154	0	-0.048250925
PTEN	C2CD3	0.154	0	-0.003600608
PTEN	P2RX4	0.154	0	0.003275948
PTEN	ALDH3A2	0.154	0	0.011491
PTEN	ASAP1	0.154	0	-0.329555389
PTEN	ALDH1A1	0.154	0	-0.207696974
PTEN	LMX1A	0.154	0	-0.538798667
PTEN	SGTB	0.154	0	-0.221818537
PTEN	CYP1B1	0.154	0	-0.035837985
PTEN	PPIL4	0.154	0	0.117848701
PTEN	OSBPL11	0.154	0	-0.075639081
PTEN	PDE6C	0.154	0	-0.184685704
PTEN	GLE1	0.154	0	-0.018912689
PTEN	COPB2	0.154	0	-0.00065621
PTEN	KCTD21	0.154	0	-0.066122946
PTEN	ANXA6	0.154	0	0.001766278
PTEN	FMO1	0.154	0	-0.004027535
PTEN	DZIP1L	0.154	0	-0.314186847
PTEN	SYT15	0.154	0	-0.04326049
PTEN	NUDT16P1	0.154	0	0.093126246
PTEN	FAM19A2	0.155	0	-0.667687086
PTEN	AWAT1	0.155	0	-0.247904075
PTEN	TMEM106A	0.155	0	-0.003346446
PTEN	C10orf129	0.155	0	-0.0005074
PTEN	RPGRIP1L	0.155	0	-0.376446379
PTEN	EIF4A2	0.155	0	-0.06837574
PTEN	PDE1B	0.155	0	-0.018340846
PTEN	GGPS1	0.155	0	-0.132570279
PTEN	SMARCC2	0.155	0	-0.04077898
PTEN	WDR93	0.155	0	-0.144767797
PTEN	C1orf220	0.155	0	-0.577632892
PTEN	PYROXD1	0.155	0	-0.130520796
PTEN	TTC39A	0.155	0	0.149308207
PTEN	C14orf21	0.155	0	-1.66E-05
PTEN	SLC39A14	0.155	0	-0.140782137
PTEN	SPINLW1	0.155	0	-0.252984309
PTEN	CTAGE1	0.155	0	-0.181921753
PTEN	IKBIP	0.155	0	-0.110175443
PTEN	ZCCHC8	0.155	0	-0.135147305

PTEN	B3GNT2	0.155	0	-0.189998847
PTEN	FBXO15	0.155	0	-0.033676514
PTEN	ANAPC4	0.155	0	1.46E-07
PTEN	FAM135B	0.155	0	-0.636553566
PTEN	SUSD3	0.155	0	0.010137141
PTEN	SLC35E2	0.156	0	-0.019436647
PTEN	ZSCAN12	0.156	0	-0.269988401
PTEN	LOC339524	0.156	0	-0.751499442
PTEN	FGFR1OP2	0.156	0	-0.004520602
PTEN	DGAT2L6	0.156	0	-0.032414878
PTEN	ADAMTS14	0.156	0	-0.001450659
PTEN	EFCAB2	0.156	0	-0.361727189
PTEN	CITED2	0.156	0	0.098689272
PTEN	HOXB6	0.156	0	-0.018806994
PTEN	GRIA1	0.156	0	-0.421457959
PTEN	AFF2	0.156	0	-4.87E-05
PTEN	RASSF9	0.156	0	-0.142552772
PTEN	PCGF6	0.156	0	-0.100818477
PTEN	LOXHD1	0.156	0	-0.271801384
PTEN	ZNF184	0.156	0	-0.052826173
PTEN	DPY19L2P4	0.156	0	-0.379168522
PTEN	TAF1B	0.156	0	-0.120233877
PTEN	TRIM22	0.156	0	-0.096186078
PTEN	COL5A3	0.156	0	-0.031612253
PTEN	SEMA3A	0.156	0	-0.22039133
PTEN	SLC36A1	0.156	0	-0.261595894
PTEN	MAPK1	0.156	0	0.195156508
PTEN	PEX1	0.156	0	-0.017703742
PTEN	LOC153684	0.156	0	-0.004746507
PTEN	FAM107B	0.156	0	-0.170133306
PTEN	RAB10	0.156	0	0.129878728
PTEN	NRIP3	0.156	0	-0.375140201
PTEN	FLJ10213	0.156	0	-0.01349894
PTEN	HDAC4	0.156	0	-0.109367165
PTEN	RXFP3	0.156	0	-0.000111541
PTEN	NKX3-1	0.156	0	-0.051873957
PTEN	CSGALNACT1	0.156	0	-0.393019384
PTEN	SERPINF1	0.157	0	0.00246643
PTEN	PTPRN2	0.157	0	-0.008450459
PTEN	BNIP2	0.157	0	-0.013056272
PTEN	BTBD9	0.157	0	-0.007961365

PTEN	CSNK1A1L	0.157	0	-0.38100036
PTEN	THBS4	0.157	0	-0.112095597
PTEN	GRIK5	0.157	0	-0.00015088
PTEN	WIPF1	0.157	0	0.071945684
PTEN	VPRBP	0.157	0	-0.017928741
PTEN	TSHZ1	0.157	0	-0.382749111
PTEN	STMN2	0.157	0	-0.129355618
PTEN	ZCCHC14	0.157	0	-0.066433048
PTEN	USP16	0.157	0	0.001961626
PTEN	MYOC	0.157	0	-0.163554196
PTEN	LOC149620	0.157	0	-0.463611057
PTEN	MMP13	0.157	0	-0.120827853
PTEN	C1orf115	0.157	0	-0.034193354
PTEN	ANO1	0.157	0	0.016162156
PTEN	C11orf54	0.157	0	-0.170987047
PTEN	ESD	0.157	0	-0.121380517
PTEN	DOPEY2	0.157	0	-0.184585459
PTEN	KDM4A	0.157	0	0.00400536
PTEN	PTPRJ	0.157	0	-0.295262591
PTEN	PRDM8	0.157	0	-0.05070634
PTEN	CAB39L	0.157	0	-0.188603782
PTEN	AAGAB	0.157	0	-0.174575545
PTEN	PCDH1	0.157	0	0.008517284
PTEN	DLG2	0.157	0	-0.357592665
PTEN	DRD1	0.157	0	-0.229598941
PTEN	PAPSS2	0.158	0	-0.089083289
PTEN	GPR88	0.158	0	-0.483218091
PTEN	C6orf208	0.158	0	-0.014053422
PTEN	MTRF1L	0.158	0	-0.106684054
PTEN	SLC12A4	0.158	0	0.004483542
PTEN	PLD1	0.158	0	-0.114074311
PTEN	TSPAN31	0.158	0	-0.000171822
PTEN	FGL2	0.158	0	0.104266479
PTEN	FBXO25	0.158	0	-0.388416338
PTEN	ZC3HAV1	0.158	0	0.036338265
PTEN	MGAT4C	0.158	0	-0.001667052
PTEN	CAV2	0.158	0	0.013603741
PTEN	FAM13A	0.158	0	-0.035888827
PTEN	ATXN1L	0.158	0	-0.005195914
PTEN	PHF2	0.158	0	0.012990684
PTEN	ASPH	0.158	0	0.077086724

PTEN	CFI	0.158	0	-0.010969978
PTEN	SLC24A3	0.158	0	-0.212663969
PTEN	TASP1	0.158	0	-0.321011608
PTEN	ANG	0.158	0	0.094267634
PTEN	GON4L	0.158	0	-0.010126698
PTEN	GOLGA8C	0.158	0	-0.614380594
PTEN	C10orf116	0.158	0	1.66E-05
PTEN	CCDC149	0.158	0	-0.324018545
PTEN	NCRNA00160	0.158	0	-0.168398301
PTEN	LOC100130148	0.158	0	-0.022223661
PTEN	DUSP19	0.159	0	-0.332610616
PTEN	EIF4E	0.159	0	-0.186646557
PTEN	SNX14	0.159	0	-0.143456247
PTEN	EML5	0.159	0	-0.356009506
PTEN	MED28	0.159	0	0.15013669
PTEN	UBE2K	0.159	0	-0.106487638
PTEN	ACO1	0.159	0	-0.086718509
PTEN	FGF20	0.159	0	-0.184510876
PTEN	HAUS3	0.159	0	-0.408394374
PTEN	C13orf31	0.159	0	-0.216058709
PTEN	ANXA2P2	0.159	0	0.044983256
PTEN	CYP4F11	0.159	0	-0.181984132
PTEN	CNOT4	0.159	0	-0.631901221
PTEN	KLF6	0.159	0	0.239385028
PTEN	KIAA1324L	0.159	0	-0.362932784
PTEN	SIGLECP3	0.159	0	-0.00452213
PTEN	CDK7	0.159	0	-0.049413743
PTEN	KIAA1522	0.159	0	0.175322217
PTEN	PMEPA1	0.159	0	-0.156247553
PTEN	FLJ10357	0.159	0	-0.083647725
PTEN	CRMP1	0.159	0	0.115052745
PTEN	CREG1	0.159	0	0.235960353
PTEN	GSTM4	0.159	0	-0.005113274
PTEN	KCNJ15	0.159	0	-0.295090841
PTEN	SIPA1L2	0.159	0	-0.146286381
PTEN	LIMD1	0.159	0	-0.055379731
PTEN	KPNA3	0.159	0	0.110925776
PTEN	SERPINA4	0.159	0	-1.54E-05
PTEN	FLJ46111	0.159	0	-0.151911961
PTEN	SLC7A4	0.159	0	-2.75E-05
PTEN	NAP1L5	0.159	0	-0.297067111

PTEN	ITGA3	0.159	0	-0.159147924
PTEN	LRRC18	0.159	0	-0.038036298
PTEN	SPATA21	0.159	0	-0.12965352
PTEN	ETF1	0.159	0	0.042522438
PTEN	ABCC3	0.16	0	-0.110700451
PTEN	TSC22D2	0.16	0	-0.550571001
PTEN	ZNF532	0.16	0	-0.160814759
PTEN	PCDHA6	0.16	0	-0.381392191
PTEN	WDR41	0.16	0	0.04563417
PTEN	RNF125	0.16	0	-0.164775638
PTEN	C6orf174	0.16	0	-0.280805618
PTEN	FREM1	0.16	0	-0.026380601
PTEN	MEX3C	0.16	0	-0.007495397
PTEN	SEC16B	0.16	0	-0.308659604
PTEN	MMP11	0.16	0	-0.005129018
PTEN	USP7	0.16	0	0.049686592
PTEN	UGT2B4	0.16	0	-0.008221518
PTEN	PABPC4L	0.16	0	-0.503993123
PTEN	ST6GALNAC3	0.16	0	-0.133679123
PTEN	MOBKL3	0.16	0	-0.031964765
PTEN	MDFIC	0.16	0	-0.090675272
PTEN	SCAND2	0.16	0	-0.025767832
PTEN	DNM3	0.16	0	-0.319713925
PTEN	PRDM11	0.16	0	-0.179653395
PTEN	NAT2	0.16	0	-0.022931653
PTEN	CHMP7	0.16	0	-0.105977908
PTEN	ZNF876P	0.16	0	-0.339191184
PTEN	CDS1	0.16	0	0.011700856
PTEN	GPRC5A	0.16	0	0.017372004
PTEN	TMPPE	0.16	0	-0.021121639
PTEN	MNAT1	0.16	0	-0.226224169
PTEN	RAB18	0.16	0	0.088173585
PTEN	EPB41L2	0.16	0	0.058428626
PTEN	TTC18	0.16	0	-0.023432429
PTEN	CEPT1	0.16	0	-0.130226849
PTEN	LYVE1	0.16	0	-0.307028735
PTEN	EIF1AX	0.16	0	0.062563343
PTEN	SLC10A6	0.16	0	-0.134837645
PTEN	ZNF586	0.16	0	-0.019321214
PTEN	APOB	0.16	0	-0.001651989
PTEN	GRIK1	0.16	0	-0.280119931

PTEN	BHLHE41	0.16	0	-0.208514005
PTEN	CLCA2	0.161	0	-0.021474828
PTEN	MYRIP	0.161	0	-0.055471938
PTEN	SLC1A4	0.161	0	-0.000735985
PTEN	CLEC2B	0.161	0	-0.474437694
PTEN	FAM18B2	0.161	0	0.104013554
PTEN	RRAGB	0.161	0	0.073200624
PTEN	ESR1	0.161	0	0.172093521
PTEN	LOC441046	0.161	0	-0.105379138
PTEN	ZMAT4	0.161	0	-0.174051527
PTEN	NOTCH4	0.161	0	-0.006835842
PTEN	RGS13	0.161	0	-0.032024878
PTEN	ZNF385A	0.161	0	0.044278563
PTEN	PXDN	0.161	0	-0.131859825
PTEN	CEP170L	0.161	0	-0.007310558
PTEN	RYK	0.161	0	0.062373647
PTEN	DYNC1LI2	0.161	0	0.057558917
PTEN	C11orf74	0.161	0	-9.03E-05
PTEN	RPRD1A	0.161	0	0.3890824
PTEN	ENOX1	0.161	0	-0.197122157
PTEN	BTBD19	0.161	0	-0.000259724
PTEN	FYTTD1	0.161	0	0.175728811
PTEN	ZSCAN23	0.161	0	-0.164345831
PTEN	LRRC37A3	0.161	0	-0.137701952
PTEN	OPA1	0.161	0	-0.068090373
PTEN	BRD3	0.161	0	-0.076257722
PTEN	RAB2B	0.161	0	-0.22119088
PTEN	TDRD3	0.161	0	-0.08219372
PTEN	TBX3	0.161	0	-0.092748029
PTEN	RNF185	0.161	0	0.023688415
PTEN	ISLR	0.161	0	0.004891377
PTEN	HEATR6	0.161	0	-0.019850697
PTEN	TIMP4	0.161	0	-0.001643657
PTEN	SILV	0.162	0	-0.17266122
PTEN	STK19	0.162	0	0.186454864
PTEN	HNRNPU	0.162	0	-0.141520974
PTEN	AGFG1	0.162	0	-0.123707872
PTEN	AHNAK2	0.162	0	-0.003067316
PTEN	EXOC1	0.162	0	-0.263603317
PTEN	C17orf91	0.162	0	-0.268671824
PTEN	SF3A1	0.162	0	-0.00417782

PTEN	SYT1	0.162	0	-0.378258536
PTEN	PCDHB7	0.162	0	-0.021036482
PTEN	GJA5	0.162	0	-0.251223734
PTEN	LAMA1	0.162	0	0.053420466
PTEN	SFMBT1	0.162	0	-0.01717164
PTEN	UBAP1	0.162	0	-0.333010445
PTEN	LIPF	0.162	0	-0.010258351
PTEN	ABCA10	0.162	0	-0.031289984
PTEN	PCYT1A	0.162	0	-0.168436846
PTEN	PPIP5K1	0.162	0	-0.169344496
PTEN	PHLPP2	0.162	0	-0.473923385
PTEN	SRGAP2	0.162	0	0.04101605
PTEN	HTT	0.162	0	0.070719082
PTEN	SCG2	0.162	0	-0.186238188
PTEN	DDX20	0.162	0	-0.270158072
PTEN	MEOX2	0.162	0	-0.335497759
PTEN	PRR16	0.162	0	-0.237641479
PTEN	SLC6A6	0.162	0	-0.024577701
PTEN	CYP4F8	0.163	0	-0.001231051
PTEN	MLEC	0.163	0	-0.02349697
PTEN	VWF	0.163	0	6.23E-05
PTEN	MEIS3	0.163	0	-0.000698862
PTEN	PHACTR2	0.163	0	-0.045789977
PTEN	HS2ST1	0.163	0	-0.179707681
PTEN	RBM3	0.163	0	0.145054404
PTEN	SCN3B	0.163	0	-0.354300424
PTEN	NFX1	0.163	0	-0.209399
PTEN	MAOA	0.163	0	-0.369564556
PTEN	NR5A2	0.163	0	-0.319123595
PTEN	IGFL4	0.163	0	-0.263531987
PTEN	DNALI1	0.163	0	-0.197896605
PTEN	PTPN11	0.163	0	-0.130110628
PTEN	THY1	0.163	0	0.167684884
PTEN	MAN1C1	0.163	0	-0.031476706
PTEN	SLC9A1	0.163	0	-0.16411327
PTEN	TRIM4	0.163	0	-0.127866143
PTEN	RELN	0.163	0	-0.396533802
PTEN	CELSR2	0.163	0	-0.023445045
PTEN	FOXF2	0.163	0	-0.292843636
PTEN	GMPR2	0.163	0	-0.020872422
PTEN	HYDIN	0.163	0	-0.043133512

PTEN	TMEM181	0.163	0	0.214605505
PTEN	HAND2	0.163	0	-0.079969417
PTEN	SLC41A2	0.163	0	0.12977449
PTEN	BLVRA	0.163	0	-0.00046905
PTEN	TINF2	0.163	0	-0.014874653
PTEN	NLGN4Y	0.163	0	-0.585846192
PTEN	TDRD1	0.163	0	-0.142422613
PTEN	KDM3A	0.164	0	0.124139854
PTEN	ATP6AP2	0.164	0	0.09097584
PTEN	TDRD7	0.164	0	-0.220871195
PTEN	LRRC27	0.164	0	-0.030579742
PTEN	FHL2	0.164	0	-0.164328604
PTEN	PCDHGA6	0.164	0	-0.001663176
PTEN	RAD23B	0.164	0	0.164089824
PTEN	BCL6B	0.164	0	-0.389006837
PTEN	ZNF28	0.164	0	-0.056015195
PTEN	FICD	0.164	0	-0.001134481
PTEN	NHS	0.164	0	-0.409025291
PTEN	ZKSCAN2	0.164	0	-0.413912312
PTEN	PGM3	0.164	0	0.157514964
PTEN	GBP3	0.164	0	-0.225012016
PTEN	PLCD3	0.164	0	-0.169264407
PTEN	RAPGEF3	0.164	0	-0.27883623
PTEN	SHOX2	0.164	0	-0.055768776
PTEN	BST1	0.164	0	-0.010967289
PTEN	DDX18	0.164	0	-0.01147062
PTEN	PYGL	0.164	0	-0.015821125
PTEN	RIOK3	0.164	0	0.044077481
PTEN	UHRF2	0.164	0	-0.133078848
PTEN	TTC12	0.164	0	-0.158200474
PTEN	KIAA2022	0.165	0	-0.34482427
PTEN	MESDC2	0.165	0	-0.044637514
PTEN	AMOTL2	0.165	0	-0.059436384
PTEN	PTP4A1	0.165	0	0.180760505
PTEN	BCL9	0.165	0	-0.315500609
PTEN	RSRC2	0.165	0	-0.184041965
PTEN	RAP1GDS1	0.165	0	0.003474722
PTEN	PDZK1	0.165	0	0.140973262
PTEN	PACRGL	0.165	0	-0.27971174
PTEN	HOXB2	0.165	0	-0.029559092
PTEN	UNC13C	0.165	0	-0.236725839

PTEN	FOXI2	0.165	0	-0.012659393
PTEN	PGM2	0.165	0	0.104841276
PTEN	ZNF397OS	0.165	0	-0.08955357
PTEN	PTH2R	0.165	0	-0.218670672
PTEN	CES1	0.165	0	-0.005510821
PTEN	CCDC76	0.165	0	-0.533097054
PTEN	NDFIP2	0.165	0	-0.083052432
PTEN	TRDN	0.165	0	-0.280648549
PTEN	PRKCI	0.165	0	-0.015292658
PTEN	QDPR	0.165	0	-0.256269243
PTEN	GDF6	0.165	0	-0.599349158
PTEN	HEY1	0.165	0	0.039358029
PTEN	ARL4A	0.166	0	-0.172579166
PTEN	TRPC3	0.166	0	-0.393753844
PTEN	RMI1	0.166	0	0.104363658
PTEN	SERPINB8	0.166	0	-0.32403773
PTEN	ZNF799	0.166	0	-0.145624846
PTEN	TCEAL5	0.166	0	-0.020695075
PTEN	MLH1	0.166	0	-0.042936561
PTEN	NBPF3	0.166	0	0.127548896
PTEN	TADA2B	0.166	0	-0.076728481
PTEN	CASP12	0.166	0	-1.70E-05
PTEN	ALDH1L2	0.166	0	-0.146594082
PTEN	C7orf60	0.166	0	-0.277585176
PTEN	DIRC1	0.166	0	-0.048266043
PTEN	FASTKD2	0.166	0	-0.391353898
PTEN	PCDHB15	0.166	0	-0.048381096
PTEN	ZNF187	0.166	0	-0.045346768
PTEN	FAM76A	0.166	0	-0.307247989
PTEN	CYP4A11	0.166	0	-0.002497853
PTEN	HSPB6	0.166	0	-0.016762523
PTEN	MTOR	0.166	0	-0.271390171
PTEN	SACM1L	0.166	0	-0.396025724
PTEN	TTF1	0.166	0	-0.187275415
PTEN	KCNJ12	0.166	0	0.063761876
PTEN	SP110	0.166	0	0.002639983
PTEN	PCDH12	0.167	0	-0.074414963
PTEN	CDKN1B	0.167	0	0.034843896
PTEN	KIAA0753	0.167	0	-0.179224595
PTEN	QKI	0.167	0	0.192583152
PTEN	SRPK2	0.167	0	-0.089407822

PTEN	SLC22A5	0.167	0	-0.104257109
PTEN	C9orf71	0.167	0	-0.03078141
PTEN	ACVR1B	0.167	0	-0.103717683
PTEN	ACSL4	0.167	0	-0.247009433
PTEN	ERO1LB	0.167	0	-0.067008634
PTEN	TGFB2	0.167	0	-0.35030568
PTEN	LAPTM4A	0.167	0	0.213994427
PTEN	CYTL1	0.167	0	-0.129372753
PTEN	SRGAP3	0.167	0	-0.199566045
PTEN	CCDC74A	0.167	0	-0.10126627
PTEN	POT1	0.167	0	-0.160062991
PTEN	CCNYL1	0.167	0	-0.260816113
PTEN	ZNF483	0.167	0	-0.33577428
PTEN	CCDC50	0.167	0	-0.157185034
PTEN	POC5	0.167	0	-0.010441412
PTEN	CA5B	0.167	0	-0.478582927
PTEN	ASCC1	0.167	0	-0.055716599
PTEN	LDLRAD1	0.167	0	-0.161320091
PTEN	SLFN11	0.167	0	-0.028950188
PTEN	SGCB	0.167	0	-0.089712943
PTEN	LRRN4CL	0.168	0	-0.005577945
PTEN	SMU1	0.168	0	-0.147648423
PTEN	SOCS7	0.168	0	-0.130761048
PTEN	RASL11B	0.168	0	-0.11980773
PTEN	DDX60	0.168	0	0.107783213
PTEN	STK32A	0.168	0	-0.015936549
PTEN	WDR70	0.168	0	-0.154628633
PTEN	NAV2	0.168	0	-0.039125252
PTEN	NBPF1	0.168	0	-1.28E-06
PTEN	NUP160	0.168	0	-0.311390055
PTEN	NLGN1	0.168	0	-0.490614378
PTEN	DCAF12L1	0.168	0	-0.077005857
PTEN	ABCC5	0.168	0	-0.125417064
PTEN	HSPA4	0.168	0	-0.177945008
PTEN	TPM1	0.168	0	0.034042239
PTEN	BAZ1A	0.168	0	-0.332777561
PTEN	IRF6	0.168	0	0.10970929
PTEN	C13orf33	0.168	0	-0.021363925
PTEN	FLVCR1	0.168	0	-0.020029506
PTEN	NSF	0.168	0	-0.051434634
PTEN	RSPO1	0.168	0	-0.15979036

PTEN	CC2D2B	0.168	0	-0.1515833
PTEN	C14orf184	0.168	0	-0.25412905
PTEN	ANXA10	0.168	0	-0.028064895
PTEN	POC1B	0.168	0	-0.141339693
PTEN	STEAP1	0.168	0	0.140306283
PTEN	BCHE	0.168	0	-0.140794141
PTEN	SNORD127	0.168	0	-0.011588277
PTEN	SLC3A1	0.168	0	-0.087086173
PTEN	ANGEL1	0.169	0	-0.022186473
PTEN	DNAH9	0.169	0	-6.65E-05
PTEN	GLIPR1	0.169	0	-0.103508336
PTEN	MSTN	0.169	0	-0.436092761
PTEN	YME1L1	0.169	0	0.17379333
PTEN	CCDC121	0.169	0	-0.035348727
PTEN	LEPREL1	0.169	0	-0.032256987
PTEN	FZD3	0.169	0	-0.18767363
PTEN	GOLPH3	0.169	0	0.196563833
PTEN	GATA3	0.169	0	0.268464277
PTEN	LYRM5	0.169	0	-0.111364067
PTEN	TMEM9B	0.169	0	-0.028274647
PTEN	EPM2AIP1	0.169	0	-0.052873124
PTEN	PTPRR	0.17	0	-0.075406118
PTEN	HTR7P1	0.17	0	-0.154943819
PTEN	RBBP4	0.17	0	-0.000912065
PTEN	DNAJC25-GNG10	0.17	0	-0.029088725
PTEN	HNRPLL	0.17	0	0.02054821
PTEN	PRKAG1	0.17	0	-0.121198927
PTEN	ALOX5AP	0.17	0	-0.006202381
PTEN	C4orf3	0.17	0	-0.010989928
PTEN	RBM44	0.17	0	-0.36082156
PTEN	SIK2	0.17	0	-0.041120222
PTEN	HDC	0.17	0	-0.118830067
PTEN	GAD1	0.17	0	-0.514089377
PTEN	HEXIM1	0.17	0	-0.281705204
PTEN	ANK2	0.17	0	-0.46189451
PTEN	LASP1	0.17	0	0.011481839
PTEN	ZNF781	0.17	0	-0.047416953
PTEN	NEK5	0.17	0	-0.254258776
PTEN	TRIM63	0.17	0	-0.220130613
PTEN	RBM25	0.17	0	-0.156899174
PTEN	KPNA1	0.17	0	0.001848588

PTEN	PIAS3	0.17	0	0.022294216
PTEN	MAPT	0.17	0	0.001563219
PTEN	GTF2IRD2B	0.17	0	-0.234558531
PTEN	CDK5RAP2	0.17	0	-0.051306805
PTEN	ANKIB1	0.17	0	-0.20579269
PTEN	WDR72	0.17	0	-0.3082037
PTEN	UBE4A	0.17	0	0.079192023
PTEN	INSIG2	0.17	0	-0.065772437
PTEN	COMMD3	0.17	0	0.003424281
PTEN	ANKRD52	0.17	0	0.003343185
PTEN	PIP4K2A	0.17	0	-0.456717781
PTEN	KCNJ13	0.171	0	-0.056051752
PTEN	CNOT7	0.171	0	0.127622951
PTEN	MMP14	0.171	0	0.160823994
PTEN	ZNF181	0.171	0	-0.054923731
PTEN	PHEX	0.171	0	-0.042341193
PTEN	AMOTL1	0.171	0	-0.256790994
PTEN	SYNPO	0.171	0	-0.146850183
PTEN	FBXO30	0.171	0	-0.422452759
PTEN	CCDC146	0.171	0	-0.350054161
PTEN	FMN2	0.171	0	-0.173274503
PTEN	MRGPRF	0.171	0	-0.028501729
PTEN	ZNF211	0.171	0	-0.197024658
PTEN	MAP3K5	0.171	0	-0.197971536
PTEN	C2orf69	0.171	0	-0.21705362
PTEN	LDLRAD3	0.171	0	0.004363628
PTEN	TLN1	0.171	0	0.096489732
PTEN	SVIL	0.171	0	-0.025372725
PTEN	BDH2	0.171	0	-0.119284821
PTEN	DDX60L	0.171	0	-0.052331244
PTEN	KBTBD2	0.171	0	-0.203645199
PTEN	SYNC	0.171	0	-0.591066354
PTEN	COX7A1	0.171	0	-0.166318494
PTEN	ADCY1	0.171	0	-0.006949391
PTEN	AFP	0.171	0	-0.031002225
PTEN	ADD3	0.171	0	-0.191395901
PTEN	GLIS1	0.171	0	-0.128307416
PTEN	COL6A1	0.171	0	0.005672019
PTEN	ZNF222	0.171	0	-0.003411238
PTEN	CSF1	0.171	0	-0.062947406
PTEN	ODZ2	0.171	0	-0.462609812

PTEN	DYNLRB2	0.172	0	-0.022787196
PTEN	MYLK	0.172	0	0.017351367
PTEN	MKX	0.172	0	-0.302813591
PTEN	STAB2	0.172	0	-0.000703156
PTEN	CTSG	0.172	0	-0.006002801
PTEN	WEE1	0.172	0	-0.371950741
PTEN	HPS5	0.172	0	-0.382736232
PTEN	CSNK2A1P	0.172	0	-0.005952426
PTEN	TPTE2P3	0.172	0	-0.287343374
PTEN	C5orf33	0.172	0	-0.052422472
PTEN	KIAA0408	0.172	0	-0.418151294
PTEN	DEGS2	0.172	0	-0.000582254
PTEN	FBXL20	0.172	0	0.129785491
PTEN	PREX1	0.172	0	-0.022905426
PTEN	PCDHB5	0.172	0	-0.026948328
PTEN	PTN	0.172	0	-0.317440625
PTEN	FAM196B	0.172	0	-0.092815109
PTEN	CYB5RL	0.172	0	-0.009101978
PTEN	UROS	0.172	0	3.35E-07
PTEN	TRIM3	0.172	0	-0.007271333
PTEN	LOC400043	0.172	0	-0.136110995
PTEN	NUDT16	0.172	0	-0.002512341
PTEN	EVC2	0.172	0	-0.021039593
PTEN	LARGE	0.172	0	0.013673503
PTEN	GRAMD1C	0.173	0	-0.507170624
PTEN	CLASP1	0.173	0	-0.211984665
PTEN	CNTLN	0.173	0	-0.686358527
PTEN	SASH1	0.173	0	-0.046017819
PTEN	LRRN1	0.173	0	-0.443158843
PTEN	RUNDC1	0.173	0	-0.007743785
PTEN	BRPF3	0.173	0	-0.136595743
PTEN	CDH8	0.173	0	-0.003435794
PTEN	NEK6	0.173	0	-0.033308263
PTEN	CCND2	0.173	0	-0.053194748
PTEN	GSTM2	0.173	0	-0.049993149
PTEN	RBL2	0.173	0	-0.284793364
PTEN	SUZ12	0.173	0	-0.191741867
PTEN	CYP2B7P1	0.173	0	-0.155853433
PTEN	KBTBD4	0.173	0	-0.010724596
PTEN	GPR176	0.173	0	-0.011192407
PTEN	C3orf57	0.173	0	0.013249365

PTEN	AK7	0.173	0	-0.184700944
PTEN	IRAK4	0.173	0	-0.388874243
PTEN	HOXA9	0.173	0	-0.20845388
PTEN	RNF38	0.173	0	-0.317570191
PTEN	UBE3C	0.173	0	0.041191882
PTEN	ARL1	0.173	0	-0.189148248
PTEN	ZNF167	0.173	0	-0.327272704
PTEN	PRDM1	0.173	0	-0.103349289
PTEN	PLEKHF2	0.173	0	-0.260188721
PTEN	SENP2	0.173	0	-0.116625305
PTEN	CYP4F22	0.174	0	-0.000154924
PTEN	FOS	0.174	0	-0.253264354
PTEN	GOPC	0.174	0	-0.084234988
PTEN	STAT2	0.174	0	0.230524058
PTEN	MOCS1	0.174	0	-0.029621624
PTEN	SNCA	0.174	0	-0.285909277
PTEN	AEBP1	0.174	0	7.16E-05
PTEN	PDE5A	0.174	0	-0.36861623
PTEN	ZFAND5	0.174	0	0.141536489
PTEN	FOXJ2	0.174	0	-0.025178234
PTEN	SERPINE1	0.174	0	0.088574019
PTEN	RAPGEF5	0.174	0	-0.021839694
PTEN	APAF1	0.174	0	-0.126112969
PTEN	ABCC13	0.174	0	-0.456628169
PTEN	ZNF737	0.174	0	-0.224613325
PTEN	RPSAP52	0.174	0	-0.000411788
PTEN	PRODH	0.174	0	-2.47E-07
PTEN	MYST3	0.174	0	-0.305522722
PTEN	GAP43	0.174	0	-0.212984045
PTEN	MCM9	0.174	0	-0.25053514
PTEN	MEIS1	0.174	0	-0.5280092
PTEN	BBS7	0.174	0	-0.218917053
PTEN	ARL6IP5	0.174	0	0.069421836
PTEN	ATP6V1D	0.174	0	-0.011880322
PTEN	AP1G1	0.174	0	-0.128964951
PTEN	CPO	0.174	0	-2.92E-07
PTEN	ZADH2	0.174	0	-0.098535559
PTEN	C3orf18	0.174	0	-0.293144276
PTEN	MTMR11	0.174	0	-0.028087159
PTEN	DCBLD2	0.175	0	-0.178809617
PTEN	PTER	0.175	0	-0.267137375

PTEN	FRMD7	0.175	0	-0.027977966
PTEN	LOC645752	0.175	0	-0.241977427
PTEN	TUBA3E	0.175	0	-0.017911489
PTEN	CRYBG3	0.175	0	-0.473003208
PTEN	OSCP1	0.175	0	-0.00551331
PTEN	TNC	0.175	0	0.00048386
PTEN	EMP1	0.175	0	0.280425931
PTEN	DIP2C	0.175	0	0.159658635
PTEN	COL24A1	0.175	0	-0.933649677
PTEN	STXBP3	0.175	0	-0.022691685
PTEN	DNAJC3	0.175	0	0.059361612
PTEN	BARD1	0.175	0	-0.002083702
PTEN	ZMYND10	0.175	0	-0.001103794
PTEN	ACPL2	0.175	0	-0.127325079
PTEN	KCTD18	0.175	0	-0.264028809
PTEN	ACOX2	0.175	0	-0.016474656
PTEN	KCNJ8	0.175	0	-0.00104603
PTEN	CCDC109A	0.175	0	-0.096383168
PTEN	HSDL2	0.175	0	-0.019486137
PTEN	GDPD1	0.175	0	-0.093661451
PTEN	TMEM45A	0.175	0	-0.021936188
PTEN	TIGD4	0.175	0	-0.002692817
PTEN	HMGCS2	0.175	0	0.006725431
PTEN	TOR1A	0.175	0	-0.014824013
PTEN	C2orf15	0.176	0	-0.263978726
PTEN	PMS1	0.176	0	-0.021162916
PTEN	DDX58	0.176	0	-0.471207536
PTEN	ANKRD6	0.176	0	-0.334239956
PTEN	EPAS1	0.176	0	-0.102671871
PTEN	CLIC5	0.176	0	-0.143046372
PTEN	ZFYVE9	0.176	0	-0.387159866
PTEN	GSTM2P1	0.176	0	-0.007772511
PTEN	HOXA7	0.176	0	-0.00381269
PTEN	RBBP8	0.176	0	-0.119973908
PTEN	COL17A1	0.176	0	-0.009954491
PTEN	EGR1	0.176	0	0.18440891
PTEN	GNG10	0.176	0	-0.002786678
PTEN	CITED1	0.176	0	-2.68E-07
PTEN	LIG4	0.176	0	-0.318541818
PTEN	LAMA3	0.176	0	-0.265625384
PTEN	CCDC68	0.176	0	-0.45427845

PTEN	ZNF613	0.176	0	-7.80E-05
PTEN	C14orf109	0.176	0	-0.066600078
PTEN	SH2B3	0.176	0	-0.236199857
PTEN	TMEM192	0.176	0	-0.288883149
PTEN	ZFP30	0.176	0	-0.432632101
PTEN	CYP7A1	0.176	0	-0.249496172
PTEN	AGAP5	0.176	0	-0.035122307
PTEN	ARSG	0.176	0	-0.001089558
PTEN	MMP10	0.176	0	-0.139118598
PTEN	MLPH	0.176	0	-0.000734118
PTEN	RAB3IP	0.176	0	-0.139969494
PTEN	SH3PXD2B	0.176	0	-0.02949312
PTEN	UNC13B	0.176	0	0.016849192
PTEN	PLCL2	0.176	0	-0.231805543
PTEN	PLXNA4	0.176	0	-0.169014775
PTEN	AGAP1	0.176	0	0.010508212
PTEN	ZNF33B	0.176	0	0.009895207
PTEN	CBLN4	0.176	0	-0.38477207
PTEN	KIAA1712	0.177	0	-0.421935608
PTEN	SGK223	0.177	0	-0.000765218
PTEN	EFR3A	0.177	0	-0.125772874
PTEN	PPAPDC3	0.177	0	-0.012070408
PTEN	ANGPT4	0.177	0	-0.000140736
PTEN	TMEM84	0.177	0	-0.280673276
PTEN	ARCN1	0.177	0	0.178257925
PTEN	GPC3	0.177	0	-0.008225408
PTEN	THOC2	0.177	0	0.110271695
PTEN	LOC284233	0.177	0	-0.013365584
PTEN	KIAA1919	0.177	0	-0.136487566
PTEN	CDC40	0.177	0	-0.117196706
PTEN	GATM	0.177	0	0.04931631
PTEN	FAM18B	0.177	0	0.022415858
PTEN	HEPACAM2	0.177	0	-0.17380102
PTEN	RGNEF	0.177	0	-0.011967774
PTEN	GRM1	0.177	0	-0.346000856
PTEN	SATB2	0.177	0	-0.80719225
PTEN	ATMIN	0.177	0	-0.056761958
PTEN	EMX2	0.177	0	-0.339749085
PTEN	NECAB1	0.177	0	-0.090531844
PTEN	PRCP	0.178	0	-0.083408446
PTEN	PCDHGA4	0.178	0	-0.001668872

PTEN	TMEM178	0.178	0	-0.21557889
PTEN	SAR1B	0.178	0	-0.085064732
PTEN	SRGAP1	0.178	0	-0.159034107
PTEN	QRICH1	0.178	0	0.045136134
PTEN	NPHP3	0.178	0	-0.191257077
PTEN	FHOD3	0.178	0	-0.021641038
PTEN	ZNF669	0.178	0	-0.031493422
PTEN	EIF2C1	0.178	0	0.05818505
PTEN	CYB5A	0.178	0	-0.179056764
PTEN	GPCPD1	0.178	0	-0.285162962
PTEN	ROPN1L	0.178	0	-0.004123105
PTEN	BMX	0.178	0	-0.373142081
PTEN	MTMR12	0.178	0	-0.072155209
PTEN	HSPG2	0.178	0	0.006823022
PTEN	LDB3	0.178	0	-0.483278654
PTEN	IFT80	0.178	0	-0.095735484
PTEN	RNF144A	0.178	0	-0.12511659
PTEN	SEZ6L	0.179	0	-0.000454036
PTEN	DUSP18	0.179	0	-0.261330294
PTEN	AFTPH	0.179	0	-0.167414079
PTEN	CELF2	0.179	0	-0.220704449
PTEN	BEND5	0.179	0	-4.04E-08
PTEN	ZSWIM5	0.179	0	-0.225373563
PTEN	C9orf4	0.179	0	-0.043064979
PTEN	C4orf36	0.179	0	-0.001981754
PTEN	PHF12	0.179	0	-0.031755625
PTEN	SP4	0.179	0	-0.289736802
PTEN	DLX2	0.179	0	-0.237424584
PTEN	DYX1C1	0.179	0	-0.45672414
PTEN	LDOC1L	0.179	0	-0.139724363
PTEN	MAPKBP1	0.179	0	-0.259950291
PTEN	EGF	0.179	0	-0.004465287
PTEN	CYP2A6	0.179	0	-0.006132531
PTEN	NTRK2	0.179	0	-0.237454439
PTEN	TPST1	0.179	0	-0.048004866
PTEN	FCN2	0.179	0	4.34E-05
PTEN	LPPR4	0.179	0	-0.250113588
PTEN	OAT	0.179	0	0.049242254
PTEN	OLFML3	0.179	0	0.00114274
PTEN	EGR3	0.179	0	-0.142814837
PTEN	OSTCL	0.179	0	-0.059566779

PTEN	AKAP6	0.179	0	-0.677103895
PTEN	DGKD	0.179	0	0.112994422
PTEN	SNX19	0.179	0	0.028866917
PTEN	FZD1	0.179	0	-0.001330626
PTEN	DSTN	0.179	0	0.219305252
PTEN	DNAJB9	0.179	0	-0.091852393
PTEN	CSDC2	0.179	0	-0.009351956
PTEN	TACC1	0.18	0	0.129820929
PTEN	KCNA1	0.18	0	-0.419238937
PTEN	RNF14	0.18	0	-0.01395955
PTEN	ACTA2	0.18	0	0.083535191
PTEN	ZNF816A	0.18	0	-0.0173399
PTEN	ROBO1	0.18	0	-0.312483311
PTEN	RBBP6	0.18	0	-0.223646938
PTEN	RNASE7	0.18	0	-0.03014686
PTEN	KIAA1468	0.18	0	-0.325674189
PTEN	ZBTB16	0.18	0	-0.006295129
PTEN	CNIH3	0.18	0	-0.00160878
PTEN	SIAE	0.18	0	-0.107407452
PTEN	PEX12	0.18	0	-0.069012378
PTEN	ZNF292	0.18	0	-0.019109646
PTEN	LOC645431	0.18	0	-0.170038248
PTEN	SPTAN1	0.18	0	0.00494793
PTEN	PGBD4	0.18	0	-0.110665895
PTEN	MYO3A	0.18	0	-0.007012217
PTEN	DEPDC6	0.18	0	-0.124073056
PTEN	TMBIM6	0.18	0	0.219771301
PTEN	HUWE1	0.18	0	0.078140036
PTEN	ZNF595	0.181	0	-0.205979435
PTEN	ADK	0.181	0	-0.518750999
PTEN	AMMECR1L	0.181	0	-0.196481628
PTEN	TWISTNB	0.181	0	0.106862932
PTEN	TRIM66	0.181	0	-0.194508242
PTEN	AGXT2L1	0.181	0	-0.021804675
PTEN	RASSF6	0.181	0	-0.415197096
PTEN	SLITRK4	0.181	0	-0.272025911
PTEN	PRPH2	0.181	0	-0.313566293
PTEN	ARRDC4	0.181	0	-0.12135433
PTEN	ACSF2	0.181	0	-0.001217473
PTEN	TUFT1	0.181	0	-0.030590507
PTEN	NPR2	0.181	0	-0.002123229

PTEN	C16orf62	0.181	0	-0.06667881
PTEN	TMEM59L	0.181	0	-2.90E-06
PTEN	CDC27	0.181	0	-0.012121045
PTEN	SSBP2	0.181	0	-0.129820047
PTEN	ARMC8	0.181	0	-0.366315106
PTEN	C8orf12	0.181	0	-0.264150961
PTEN	DDAH1	0.181	0	0.105856359
PTEN	SPOCK3	0.181	0	-0.732798315
PTEN	KDSR	0.181	0	-0.388817572
PTEN	LOC284276	0.181	0	-0.302381504
PTEN	UNC119B	0.181	0	-0.114508043
PTEN	MAN2B2	0.181	0	-0.152729345
PTEN	PRELID2	0.181	0	-0.284205381
PTEN	KCNIP4	0.181	0	-0.322844617
PTEN	RBM5	0.181	0	0.172377275
PTEN	GEM	0.181	0	-0.269802899
PTEN	ITGA5	0.181	0	0.457453523
PTEN	CDK13	0.181	0	-0.285841628
PTEN	RIPK1	0.181	0	-0.146640056
PTEN	ZNF182	0.181	0	-0.293857082
PTEN	NAIP	0.181	0	-0.173787428
PTEN	FZD10	0.182	0	-0.260673886
PTEN	PTPRA	0.182	0	0.014559106
PTEN	POTEG	0.182	0	-0.310677914
PTEN	SNX33	0.182	0	0.157503991
PTEN	DNAH6	0.182	0	-3.14E-05
PTEN	HDGFRP3	0.182	0	-0.1994838
PTEN	GPR39	0.182	0	-0.298456314
PTEN	NEDD4L	0.182	0	-0.157620366
PTEN	WAC	0.182	0	0.186665763
PTEN	SMPD1	0.182	0	-0.123529636
PTEN	ZDHHC3	0.182	0	-0.333385251
PTEN	ZNF548	0.182	0	-0.043632499
PTEN	TRAM1L1	0.182	0	-0.318024532
PTEN	ATP6V1B2	0.182	0	0.170113384
PTEN	AEBP2	0.182	0	-0.055758056
PTEN	FAM105A	0.182	0	-0.310179931
PTEN	GTF2IRD2P1	0.182	0	-0.003179362
PTEN	ABCB10	0.183	0	-0.312068496
PTEN	ZBED3	0.183	0	-0.000565651
PTEN	C9orf91	0.183	0	-0.067091775

PTEN	FBXO42	0.183	0	-0.022704651
PTEN	PIP5K1B	0.183	0	-0.397757735
PTEN	CDKL1	0.183	0	-9.93E-07
PTEN	DUSP3	0.183	0	-0.084253136
PTEN	TMEM19	0.183	0	-0.169565887
PTEN	PIBF1	0.183	0	-0.111780495
PTEN	ZNF451	0.183	0	-0.152354936
PTEN	ALS2CR12	0.183	0	-0.171316233
PTEN	MTRR	0.183	0	-0.182427505
PTEN	OCLN	0.183	0	-0.020421751
PTEN	KIAA1107	0.183	0	-0.028449746
PTEN	C1orf150	0.183	0	-0.050097814
PTEN	DKFZp686O24166	0.183	0	-0.003020218
PTEN	KLF10	0.183	0	0.048232181
PTEN	KIAA1826	0.183	0	-0.210544999
PTEN	C15orf29	0.183	0	-0.080162296
PTEN	NMNAT1	0.183	0	-0.032482685
PTEN	EAPP	0.183	0	-0.019269456
PTEN	ZCCHC5	0.183	0	-0.212400351
PTEN	NEFL	0.183	0	-0.091428096
PTEN	ARNTL	0.183	0	-0.178148172
PTEN	CCDC40	0.183	0	-0.520568173
PTEN	KDELC1	0.183	0	-0.061694
PTEN	ACTR1A	0.183	0	0.195299107
PTEN	DLST	0.183	0	0.119422697
PTEN	AUH	0.183	0	-0.255581351
PTEN	NKAIN2	0.183	0	-0.518819894
PTEN	MAGT1	0.183	0	0.043777188
PTEN	KIAA1644	0.184	0	-0.009305253
PTEN	NPY1R	0.184	0	0.039975767
PTEN	PRND	0.184	0	-0.347408399
PTEN	TNS3	0.184	0	-0.154159513
PTEN	TTC23L	0.184	0	-4.20E-05
PTEN	TAOK3	0.184	0	-0.298944783
PTEN	GRAMD3	0.184	0	-0.068380715
PTEN	CCND1	0.184	0	0.026325073
PTEN	SHROOM1	0.184	0	0.000341086
PTEN	ARHGEF17	0.184	0	-0.031765711
PTEN	CISH	0.184	0	0.004168729
PTEN	SLC5A7	0.184	0	-0.405075151
PTEN	TRHDE	0.184	0	-0.517735465

PTEN	DMXL2	0.184	0	-0.066567943
PTEN	MAX	0.184	0	0.229686999
PTEN	C3orf52	0.184	0	-0.042777752
PTEN	COL15A1	0.184	0	0.218170756
PTEN	NUPL1	0.184	0	-0.040079412
PTEN	MERTK	0.184	0	-0.04932876
PTEN	ERLEC1	0.184	0	0.078429259
PTEN	PDE4DIP	0.184	0	0.361147965
PTEN	HEPH	0.184	0	-0.26109406
PTEN	PCDH20	0.184	0	-0.516570957
PTEN	HAS2AS	0.184	0	-0.148325446
PTEN	PTGR1	0.184	0	-0.037090686
PTEN	USP51	0.184	0	-0.186863029
PTEN	CCDC74B	0.185	0	-0.153150702
PTEN	CACNB4	0.185	0	-0.202189269
PTEN	TNFSF8	0.185	0	-0.133212278
PTEN	CCKAR	0.185	0	-5.22E-09
PTEN	FBLN1	0.185	0	-0.344722813
PTEN	LMTK2	0.185	0	-0.124397738
PTEN	LOC647859	0.185	0	-0.000471896
PTEN	C11orf70	0.185	0	-0.0213682
PTEN	FAIM2	0.185	0	-0.000403705
PTEN	TMEM232	0.185	0	-0.286014736
PTEN	CUEDC1	0.185	0	-0.014105434
PTEN	SERPINI1	0.185	0	0.043031308
PTEN	LOC643763	0.185	0	-0.009622037
PTEN	CYP2A7	0.185	0	-0.137963066
PTEN	MEIS2	0.185	0	-0.190761788
PTEN	ZNF449	0.185	0	-0.21927743
PTEN	SLC33A1	0.185	0	-0.013646376
PTEN	C22orf30	0.185	0	0.124722903
PTEN	RNF217	0.185	0	-0.16388614
PTEN	LUZP2	0.185	0	-0.418067643
PTEN	PARP11	0.185	0	-0.004474042
PTEN	SLCO1C1	0.185	0	-0.364347742
PTEN	SAMHD1	0.186	0	0.128838608
PTEN	PCDP1	0.186	0	-0.030224528
PTEN	SEPN1	0.186	0	0.001305023
PTEN	MTMR3	0.186	0	-0.069985846
PTEN	AGAP11	0.186	0	-0.01357363
PTEN	SNX25	0.186	0	-0.257812049

PTEN	PIGG	0.186	0	0.002151275
PTEN	FAF2	0.186	0	0.138836529
PTEN	CCDC113	0.186	0	-0.068626531
PTEN	CEP170	0.186	0	-0.365027772
PTEN	MAFB	0.186	0	-0.038772545
PTEN	GABPB2	0.186	0	-0.38478187
PTEN	DUSP10	0.186	0	-0.144778307
PTEN	CARD8	0.186	0	-0.358944637
PTEN	PCBD2	0.186	0	-0.107724334
PTEN	SMEK2	0.186	0	0.157502162
PTEN	CDH5	0.187	0	-0.014701932
PTEN	HECW1	0.187	0	-0.220906813
PTEN	ZFP62	0.187	0	-0.18865261
PTEN	N4BP1	0.187	0	-0.012940702
PTEN	NLGN3	0.187	0	-0.281275278
PTEN	PLA2G3	0.187	0	-0.235494763
PTEN	ATG4C	0.187	0	-0.267781354
PTEN	C2orf42	0.187	0	-0.02785583
PTEN	C9orf156	0.187	0	-0.094667622
PTEN	PRRG3	0.187	0	-0.00197745
PTEN	CLINT1	0.187	0	-0.374858358
PTEN	G2E3	0.187	0	-0.216631874
PTEN	ADAMTS19	0.187	0	-0.332894143
PTEN	OSGEPL1	0.187	0	0.050334291
PTEN	PLA2G12A	0.187	0	0.121242706
PTEN	ELP4	0.187	0	-0.015816746
PTEN	C12orf4	0.187	0	-0.467694953
PTEN	CD99L2	0.187	0	-0.00051124
PTEN	KIF26B	0.187	0	-0.160510127
PTEN	DFNA5	0.187	0	-0.224918216
PTEN	CCDC111	0.187	0	-0.118421772
PTEN	BIRC2	0.187	0	-0.278132114
PTEN	C9orf21	0.187	0	-0.126672723
PTEN	MFSD6	0.187	0	-0.112954357
PTEN	AREG	0.188	0	-0.188555848
PTEN	ATR	0.188	0	-0.096793801
PTEN	TPTE	0.188	0	-0.194999332
PTEN	EIF1B	0.188	0	-0.251746051
PTEN	SERPINB1	0.188	0	-0.019080438
PTEN	SPTLC3	0.188	0	-0.087454864
PTEN	SYT17	0.188	0	-0.075078829

PTEN	CCBL2	0.188	0	-0.010684265
PTEN	DCDC2	0.188	0	-0.495800323
PTEN	DACT3	0.188	0	-0.046269187
PTEN	F3	0.188	0	-0.144759893
PTEN	HOXB5	0.188	0	-0.020264392
PTEN	MBNL3	0.188	0	-0.222848565
PTEN	ZNF563	0.188	0	-0.008753775
PTEN	RCVRN	0.188	0	-0.00969547
PTEN	MFN1	0.188	0	-0.085682088
PTEN	RNF6	0.188	0	-0.152405247
PTEN	GFRA1	0.188	0	0.000492203
PTEN	N4BP2L2	0.188	0	-0.039527435
PTEN	ATP13A3	0.188	0	-0.292356872
PTEN	ZNF749	0.188	0	-0.008441915
PTEN	PHGR1	0.188	0	-0.001139864
PTEN	GTF2H1	0.188	0	0.039156997
PTEN	LOC90586	0.188	0	-0.197297336
PTEN	PID1	0.189	0	-0.018061075
PTEN	GLS2	0.189	0	-0.030497934
PTEN	METTL10	0.189	0	-0.054185016
PTEN	SLC5A4	0.189	0	-0.004857394
PTEN	IQUB	0.189	0	-0.387603128
PTEN	DPT	0.189	0	-0.102431983
PTEN	GRIN2A	0.189	0	-0.00019358
PTEN	BAG4	0.189	0	-0.000644278
PTEN	S1PR3	0.189	0	-0.050628037
PTEN	ANKRD26	0.189	0	-0.252993111
PTEN	SQRDL	0.189	0	-0.156211522
PTEN	DZIP3	0.189	0	-0.145200004
PTEN	CTNNAL1	0.189	0	-0.037611773
PTEN	EPHA5	0.189	0	-0.782744472
PTEN	HGD	0.189	0	-0.048914534
PTEN	AFAP1	0.189	0	-0.009912201
PTEN	ACADM	0.189	0	0.045027957
PTEN	LIN7A	0.189	0	-0.184487014
PTEN	FAR1	0.189	0	-0.063488363
PTEN	FLJ11235	0.189	0	-0.050074743
PTEN	DSTYK	0.189	0	0.057646106
PTEN	ZNF551	0.189	0	-0.043978571
PTEN	MREG	0.189	0	-0.357220426
PTEN	SFRS11	0.189	0	0.074998244

PTEN	IFT74	0.189	0	0.03633629
PTEN	CCDC144B	0.189	0	-0.103862773
PTEN	LPHN3	0.189	0	-0.080614135
PTEN	HELB	0.19	0	-5.88E-08
PTEN	PSMC6	0.19	0	-0.078141206
PTEN	MYH11	0.19	0	-0.052955805
PTEN	PPAP2A	0.19	0	-0.018693803
PTEN	NUMA1	0.19	0	0.029726584
PTEN	C1orf27	0.19	0	-0.149016529
PTEN	CMTM1	0.19	0	-0.006390314
PTEN	NALCN	0.19	0	-0.063030196
PTEN	CACHD1	0.19	0	-0.185546191
PTEN	SEC24C	0.19	0	0.000391151
PTEN	COPS8	0.19	0	-0.19361346
PTEN	DMGDH	0.19	0	-0.353422034
PTEN	TFAP2B	0.19	0	-0.028161038
PTEN	PIGV	0.19	0	-0.026971384
PTEN	ROR1	0.19	0	-0.02784539
PTEN	MYO1B	0.19	0	0.18767241
PTEN	FAM47E	0.19	0	-0.036574222
PTEN	WDR63	0.19	0	-0.157428286
PTEN	ARSB	0.19	0	-0.67094076
PTEN	NKAIN4	0.19	0	-0.039807744
PTEN	REEP1	0.19	0	-0.23938388
PTEN	HECW2	0.19	0	-0.193671797
PTEN	ZNRF2	0.19	0	-0.240357202
PTEN	LMBRD1	0.19	0	-0.184753925
PTEN	S1PR1	0.19	0	-0.285160741
PTEN	MAGED2	0.19	0	0.017503475
PTEN	ACOT4	0.19	0	-0.000115017
PTEN	ZNF385D	0.19	0	-0.139546988
PTEN	SH2D4A	0.19	0	-0.193692664
PTEN	PCDHGA10	0.19	0	-0.001609653
PTEN	CSMD1	0.19	0	-0.453038644
PTEN	TRIM33	0.19	0	-0.449732448
PTEN	SMAD9	0.19	0	-0.083495338
PTEN	TMEM49	0.19	0	0.038580866
PTEN	UBE3B	0.19	0	-0.105503524
PTEN	GPR124	0.19	0	-0.034087269
PTEN	ZNF611	0.191	0	-0.211404716
PTEN	SLC38A9	0.191	0	-0.044863569

PTEN	TRAK1	0.191	0	-0.187784574
PTEN	SCG5	0.191	0	-0.243413133
PTEN	SCN3A	0.191	0	-0.141053054
PTEN	MADD	0.191	0	-0.007949205
PTEN	CNTN2	0.191	0	-0.018291362
PTEN	ESCO1	0.191	0	-0.120124337
PTEN	UBE2B	0.191	0	-0.121614341
PTEN	KCNJ2	0.191	0	-0.31959453
PTEN	ZNF154	0.191	0	-0.192687475
PTEN	SGK196	0.191	0	-0.008066887
PTEN	CNTN4	0.191	0	-0.199731617
PTEN	ISCA1P1	0.191	0	-0.042126944
PTEN	MAPK1IP1L	0.191	0	-0.374637588
PTEN	KRT222	0.191	0	-0.183750814
PTEN	SRRM2	0.191	0	-0.04293567
PTEN	RC3H1	0.191	0	-0.008164147
PTEN	SP100	0.191	0	-0.00946163
PTEN	CDH23	0.191	0	-0.003548618
PTEN	ITGB6	0.191	0	0.00116619
PTEN	SEMA4B	0.191	0	-0.024664014
PTEN	GMEB1	0.191	0	-0.131216486
PTEN	ACTR8	0.191	0	-0.040319528
PTEN	ZNF507	0.191	0	-0.198310383
PTEN	C21orf34	0.192	0	-0.424988068
PTEN	TRIM78P	0.192	0	-0.616862321
PTEN	RBPJ	0.192	0	-0.107994456
PTEN	EGLN3	0.192	0	-0.089337606
PTEN	UBE2D3	0.192	0	0.40963758
PTEN	LOXL1	0.192	0	-0.000620216
PTEN	PITX2	0.192	0	-0.491886687
PTEN	C1orf173	0.192	0	-0.231897236
PTEN	SIGLEC6	0.192	0	-0.297012339
PTEN	TRIM58	0.192	0	-0.021206828
PTEN	HRH1	0.192	0	-0.193218811
PTEN	EBF3	0.192	0	-0.061175336
PTEN	PLS1	0.192	0	-0.297975255
PTEN	ADNP	0.192	0	-0.067723381
PTEN	ARHGEF3	0.192	0	-0.167450361
PTEN	ATRNL1	0.192	0	-0.222251023
PTEN	NUP214	0.192	0	-0.000140254
PTEN	BHLHB9	0.192	0	-0.155642899

PTEN	GAS1	0.192	0	-0.201366716
PTEN	CANX	0.192	0	0.295402795
PTEN	MGAT4A	0.192	0	-0.147243293
PTEN	CWC27	0.193	0	-0.283282024
PTEN	HHIP	0.193	0	-0.43163817
PTEN	ZFR	0.193	0	0.039071657
PTEN	GUSBP1	0.193	0	-0.21163563
PTEN	TRIM5	0.193	0	-0.080482639
PTEN	CDC42BPA	0.193	0	0.08859008
PTEN	VPS36	0.193	0	-0.025650068
PTEN	SCCPDH	0.193	0	-0.039937636
PTEN	FLT3	0.193	0	-0.159529499
PTEN	NFATC2	0.193	0	-9.13E-07
PTEN	ZNF620	0.193	0	-0.003471759
PTEN	IMPG1	0.193	0	-0.201543306
PTEN	BMP4	0.193	0	-0.042269479
PTEN	PRKCE	0.193	0	-0.336770186
PTEN	EXOC4	0.193	0	-0.149906615
PTEN	ANAPC1	0.193	0	-0.171401747
PTEN	TOB2	0.193	0	-0.071323517
PTEN	ARHGEF10	0.194	0	-0.072317345
PTEN	GNG11	0.194	0	-0.004121137
PTEN	TSC1	0.194	0	-0.092678222
PTEN	DDO	0.194	0	-0.168770219
PTEN	FMNL3	0.194	0	-8.21E-05
PTEN	CREB3L4	0.194	0	1.46E-05
PTEN	PAFAH2	0.194	0	-0.181380631
PTEN	NR2C2	0.194	0	-0.137267463
PTEN	POTEE	0.194	0	-0.000131976
PTEN	UTP20	0.194	0	-0.238681713
PTEN	ABI3BP	0.194	0	-0.203443091
PTEN	ENDOD1	0.194	0	-0.115379598
PTEN	C2orf60	0.194	0	-0.262065079
PTEN	NXNL2	0.194	0	-0.045215017
PTEN	ZBTB7C	0.194	0	0.03198922
PTEN	OCLM	0.194	0	-0.012685458
PTEN	SLC5A3	0.194	0	-0.015428551
PTEN	METAP2	0.194	0	0.169798035
PTEN	VPS37A	0.194	0	-0.26388831
PTEN	SMC5	0.194	0	-0.231015458
PTEN	WHAMM	0.194	0	-0.213136967

PTEN	MGAT2	0.194	0	-0.087601079
PTEN	EIF2S3	0.194	0	0.108349543
PTEN	ERG	0.195	0	-0.174301919
PTEN	TMEM57	0.195	0	-0.279338668
PTEN	USO1	0.195	0	0.007986425
PTEN	CAP2	0.195	0	-0.118776188
PTEN	ZNF587	0.195	0	-0.015623464
PTEN	ZNF583	0.195	0	-0.025731924
PTEN	CYFIP1	0.195	0	-0.102900032
PTEN	POLR3B	0.195	0	-0.166950892
PTEN	IMPDH2	0.195	0	8.39E-05
PTEN	MEF2A	0.195	0	-0.079578284
PTEN	SENP8	0.195	0	-0.335507786
PTEN	ZNF740	0.195	0	-0.004558242
PTEN	IARS2	0.195	0	0.016031121
PTEN	ZNF704	0.195	0	-0.141978929
PTEN	DMBT1	0.195	0	-0.001583649
PTEN	USP40	0.195	0	1.34E-05
PTEN	CLK1	0.195	0	-0.160364799
PTEN	LOC100286844	0.195	0	-0.107485641
PTEN	BCCIP	0.195	0	-0.065918843
PTEN	ADAL	0.195	0	-0.038731175
PTEN	FLNC	0.195	0	-0.082893113
PTEN	CRNKL1	0.195	0	0.127582413
PTEN	C4orf49	0.195	0	-0.044429791
PTEN	FKBP9	0.195	0	-0.205460885
PTEN	AOC3	0.195	0	-0.147358633
PTEN	ZNF543	0.195	0	-0.012164114
PTEN	CEACAM7	0.195	0	-0.189079521
PTEN	PPP2R2D	0.195	0	-0.001152235
PTEN	ZNF552	0.195	0	0.167973973
PTEN	PPAPDC2	0.195	0	-0.005217114
PTEN	OLFML2A	0.195	0	-0.034599044
PTEN	CLIC6	0.195	0	0.015947861
PTEN	MED14	0.196	0	-0.19439298
PTEN	BAT2L1	0.196	0	0.015039485
PTEN	SDR16C5	0.196	0	-0.032428341
PTEN	LOC641298	0.196	0	-0.306398079
PTEN	PTPN14	0.196	0	-0.009839961
PTEN	LRRC4	0.196	0	-0.207702074
PTEN	ZBTB44	0.196	0	-0.004225577

PTEN	ASNSD1	0.196	0	0.000523879
PTEN	ZNF304	0.196	0	-0.141605265
PTEN	ZNF823	0.196	0	-0.144491944
PTEN	SGIP1	0.196	0	-0.429238103
PTEN	CLDN11	0.196	0	-0.538955504
PTEN	GHRH	0.196	0	-0.00071317
PTEN	TEKT3	0.196	0	-0.00049647
PTEN	NKAPL	0.196	0	-0.313414898
PTEN	RCAN3	0.196	0	-0.022127862
PTEN	SLC38A1	0.196	0	0.07041924
PTEN	PTGS1	0.196	0	-0.202761379
PTEN	ADRB2	0.196	0	0.045322558
PTEN	COL11A1	0.196	0	-0.147054485
PTEN	NPHP1	0.196	0	-0.168074917
PTEN	GUCY1B3	0.196	0	-0.216323541
PTEN	ARAP2	0.196	0	-0.199345288
PTEN	ERO1L	0.196	0	-0.167885531
PTEN	MMRN2	0.196	0	-0.100341066
PTEN	LGALS9B	0.196	0	-7.19E-07
PTEN	LRRC70	0.196	0	-0.184491753
PTEN	IFITM2	0.196	0	0.005696725
PTEN	ZNF788	0.196	0	0.009821421
PTEN	EFCAB7	0.196	0	-0.093986437
PTEN	ESYT1	0.196	0	0.074757168
PTEN	ZNF19	0.196	0	-0.253331673
PTEN	EXT2	0.196	0	0.005443219
PTEN	INVS	0.196	0	-0.259523759
PTEN	ADRBK2	0.196	0	-0.097921016
PTEN	CDADC1	0.197	0	-0.265018601
PTEN	C1orf111	0.197	0	-0.002016038
PTEN	DEFB132	0.197	0	-0.233107458
PTEN	PLBD2	0.197	0	-0.000877951
PTEN	TEX2	0.197	0	-0.086800035
PTEN	NPEPPS	0.197	0	-0.278085981
PTEN	KIAA0368	0.197	0	-0.071073055
PTEN	XPC	0.197	0	-0.007766099
PTEN	ZNF648	0.197	0	-0.278597042
PTEN	ATP8B4	0.197	0	-0.278833014
PTEN	KLHL15	0.197	0	-0.247080493
PTEN	ARL2BP	0.197	0	-3.50E-05
PTEN	RNF133	0.197	0	-0.001408535

PTEN	AGR2	0.197	0	0.02155256
PTEN	NFIC	0.197	0	-2.51E-05
PTEN	C2CD4A	0.197	0	-0.052994346
PTEN	EIF2S1	0.197	0	0.038316046
PTEN	SNX9	0.197	0	0.090976018
PTEN	C1orf226	0.197	0	-0.007095498
PTEN	C6orf191	0.198	0	-0.131896193
PTEN	FAM200A	0.198	0	-0.019822724
PTEN	SPCS3	0.198	0	0.112672526
PTEN	NUDT10	0.198	0	-0.359681986
PTEN	CPZ	0.198	0	-0.110259941
PTEN	P2RY12	0.198	0	-0.027071812
PTEN	NNT	0.198	0	-0.216305404
PTEN	TMEM167B	0.198	0	0.2037117
PTEN	DDX21	0.198	0	0.015685235
PTEN	C1orf64	0.198	0	-0.011511666
PTEN	GOLGA8E	0.198	0	-0.592188788
PTEN	SLC35B4	0.198	0	0.076720259
PTEN	SLC25A30	0.198	0	0.101618012
PTEN	HMGCR	0.198	0	-0.105058917
PTEN	FAM8A1	0.198	0	-0.045960554
PTEN	ZNF708	0.198	0	-0.110585492
PTEN	C21orf96	0.198	0	-0.000794295
PTEN	GOLGA8F	0.198	0	-0.612070724
PTEN	POTEH	0.198	0	-0.002727707
PTEN	PBX3	0.198	0	-0.316387534
PTEN	EYA4	0.198	0	-0.332711198
PTEN	RPL41	0.198	0	0.12809576
PTEN	MED12L	0.198	0	-0.576725709
PTEN	COL6A6	0.198	0	-0.332094112
PTEN	CXXC4	0.198	0	-0.191547532
PTEN	CMTM3	0.198	0	-0.171946089
PTEN	CTTNBP2NL	0.198	0	0.139832866
PTEN	KLK4	0.198	0	-0.000529392
PTEN	OPCML	0.198	0	-0.151639837
PTEN	PARP4	0.198	0	-0.161158761
PTEN	DOCK7	0.198	0	-0.0603366
PTEN	GALNTL6	0.198	0	-0.365884474
PTEN	NLRP5	0.198	0	-0.001477574
PTEN	SSTR2	0.199	0	-0.026272986
PTEN	MYOCD	0.199	0	-0.191728758

PTEN	DEGS1	0.199	0	0.188581016
PTEN	EEF2K	0.199	0	-0.224724038
PTEN	ZDBF2	0.199	0	-0.329294535
PTEN	IL20	0.199	0	-0.019259044
PTEN	ZNF782	0.199	0	-0.023521389
PTEN	CYP4V2	0.199	0	-0.09559455
PTEN	FAM13C	0.199	0	0.014554675
PTEN	C20orf26	0.199	0	-0.001197092
PTEN	FAM196A	0.199	0	-0.255738159
PTEN	PCSK6	0.199	0	-0.115995954
PTEN	RORC	0.199	0	-0.162613441
PTEN	C8orf84	0.199	0	-0.405247204
PTEN	KCNJ3	0.199	0	0.124136876
PTEN	MAP3K12	0.199	0	-0.043392078
PTEN	GFM2	0.199	0	-0.161748065
PTEN	REP15	0.199	0	-0.011520736
PTEN	ST6GALNAC2	0.199	0	-0.022936737
PTEN	FRZB	0.199	0	0.002917136
PTEN	ZNF160	0.199	0	-0.042464479
PTEN	RPS27L	0.199	0	0.065719126
PTEN	KCNS3	0.199	0	-0.282715838
PTEN	SPATA4	0.199	0	-0.16904672
PTEN	RBPMS	0.199	0	0.016323929
PTEN	CYP4A22	0.199	0	-0.002653785
PTEN	LOC641367	0.199	0	-0.338006606
PTEN	LMBR1	0.199	0	-0.095448764
PTEN	SACS	0.199	0	-0.116407864
PTEN	OSBP	0.2	0	-0.125794286
PTEN	GDE1	0.2	0	0.239294478
PTEN	USP54	0.2	0	-0.0261225
PTEN	CCDC36	0.2	0	-0.038505986
PTEN	SETDB2	0.2	0	-0.155431706
PTEN	CWC22	0.2	0	-0.135514455
PTEN	STK17B	0.2	0	-0.220428018
PTEN	CNN3	0.2	0	0.147358955
PTEN	ADAT1	0.2	0	-0.135993372
PTEN	ADAMTSL3	0.2	0	-0.266285979
PTEN	C14orf106	0.2	0	-0.195867447
PTEN	ACMSD	0.2	0	-0.005531608
PTEN	ZNF84	0.2	0	-0.095326051
PTEN	COL21A1	0.2	0	-0.058190633

PTEN	ATP10A	0.2	0	0.014748341
PTEN	CTAGE5	0.2	0	-0.182881001
PTEN	SHC1	0.2	0	-0.014032566
PTEN	AGXT2	0.2	0	-0.02513395
PTEN	CWF19L1	0.2	0	-0.057992662
PTEN	TUBA3D	0.2	0	-0.000135675
PTEN	GTF2H2B	0.2	0	-0.285806472
PTEN	SLC29A3	0.2	0	0.118017597
PTEN	SAMD4A	0.2	0	0.072890531
PTEN	PAFAH1B1	0.2	0	0.042518297
PTEN	ATP2B1	0.2	0	0.141963379
PTEN	C12orf35	0.2	0	-0.308556774
PTEN	TPH1	0.2	0	-0.259495821
PTEN	CALHM2	0.201	0	-0.014402848
PTEN	ANGPT2	0.201	0	-0.289017569
PTEN	ZNF311	0.201	0	-3.11E-05
PTEN	C1orf101	0.201	0	-0.353110987
PTEN	ATL1	0.201	0	0.033757595
PTEN	POLL	0.201	0	-0.003712573
PTEN	EMCN	0.201	0	-0.096722137
PTEN	TAF1L	0.201	0	-0.178457293
PTEN	CLK4	0.201	0	0.0659408
PTEN	LRRC48	0.201	0	-0.167244044
PTEN	GABPB1	0.201	0	-0.364310383
PTEN	PDPK1	0.201	0	-0.120530813
PTEN	TAL1	0.201	0	-0.252708786
PTEN	KLK2	0.201	0	-0.341503406
PTEN	ADAM23	0.201	0	-0.308562996
PTEN	ZNF226	0.201	0	-0.006959796
PTEN	CAPN9	0.201	0	-0.096867989
PTEN	ADSS	0.201	0	0.201258409
PTEN	ADO	0.201	0	-0.163797753
PTEN	FAM169A	0.201	0	-0.371045347
PTEN	COLEC12	0.201	0	-0.18731918
PTEN	EIF5A2	0.201	0	-0.141644007
PTEN	SELT	0.202	0	0.015104482
PTEN	PHACTR4	0.202	0	-0.093326663
PTEN	MANEA	0.202	0	-0.232719497
PTEN	C1orf190	0.202	0	-0.539500019
PTEN	ERI2	0.202	0	-0.235993986
PTEN	ANKS1B	0.202	0	-0.021974947

PTEN	DYM	0.202	0	0.018320575
PTEN	MAP2K5	0.202	0	-4.37E-06
PTEN	LRP4	0.202	0	-0.436039855
PTEN	IQCH	0.202	0	-0.198323312
PTEN	PCDHGB6	0.202	0	-0.001647698
PTEN	DGKA	0.202	0	-0.084860515
PTEN	CAP1	0.202	0	0.034629192
PTEN	G3BP1	0.202	0	-0.10524948
PTEN	C20orf114	0.202	0	-0.014753886
PTEN	FAM21B	0.202	0	-0.10006
PTEN	MEG3	0.202	0	-0.004313718
PTEN	C2orf86	0.202	0	-0.065197356
PTEN	TMEM59	0.202	0	0.07313378
PTEN	RGAG4	0.202	0	-0.020853956
PTEN	IGF1R	0.202	0	0.013505165
PTEN	NRP2	0.202	0	0.122292174
PTEN	ICK	0.202	0	-0.110754175
PTEN	DDX17	0.202	0	0.09347577
PTEN	EFHA1	0.203	0	-0.010548107
PTEN	LRGUK	0.203	0	-0.031138722
PTEN	CXorf36	0.203	0	-0.019705086
PTEN	MTMR15	0.203	0	0.130530139
PTEN	ANKRD28	0.203	0	-0.260600808
PTEN	IRAK3	0.203	0	-0.009343521
PTEN	JAZF1	0.203	0	-0.185949816
PTEN	TSSK4	0.203	0	-4.70E-06
PTEN	ZNF681	0.203	0	-0.378079672
PTEN	CLCN6	0.203	0	-0.029902175
PTEN	ACVR2A	0.203	0	-0.433180014
PTEN	RAD54L2	0.203	0	-0.003160372
PTEN	PTPDC1	0.203	0	-0.090364362
PTEN	ZNF675	0.203	0	-0.003994545
PTEN	PAPPA2	0.203	0	-0.29906403
PTEN	PDPR	0.203	0	-0.114522803
PTEN	LPCAT2	0.204	0	0.004484042
PTEN	KIAA1267	0.204	0	-0.194846021
PTEN	CCDC30	0.204	0	-0.170231217
PTEN	NDUFS1	0.204	0	-0.049695698
PTEN	ERMP1	0.204	0	-0.340288566
PTEN	USP46	0.204	0	-0.104830061
PTEN	RALGAPB	0.204	0	-0.060039291

PTEN	MOBKL1A	0.204	0	-0.202468142
PTEN	VPS13B	0.204	0	-0.113242894
PTEN	HSP90AB2P	0.204	0	-0.021239094
PTEN	PPIC	0.204	0	-0.031465298
PTEN	CTHRC1	0.204	0	0.179629077
PTEN	BACE1	0.204	0	-0.217502841
PTEN	SAV1	0.204	0	-0.273059571
PTEN	PRO0611	0.204	0	-0.054479554
PTEN	ADORA3	0.204	0	-0.133350136
PTEN	RBM26	0.204	0	-0.310513446
PTEN	PCDHGA2	0.204	0	-0.001653768
PTEN	KIF20B	0.204	0	-0.044214364
PTEN	VPS4B	0.204	0	-0.014517892
PTEN	ZNF843	0.204	0	-0.000265575
PTEN	TNFRSF10C	0.204	0	-0.001152226
PTEN	RG9MTD2	0.204	0	-0.30089541
PTEN	RRN3P2	0.204	0	-0.300995837
PTEN	ZNF461	0.204	0	-0.154904884
PTEN	IGFBP4	0.204	0	0.131675139
PTEN	CLDN12	0.204	0	0.160839931
PTEN	CEP63	0.205	0	-0.584269102
PTEN	DHX9	0.205	0	0.11084156
PTEN	ARL5B	0.205	0	0.068789953
PTEN	HMGXB4	0.205	0	-0.242656334
PTEN	PDP2	0.205	0	-0.400739219
PTEN	NDUFS4	0.205	0	-0.067380313
PTEN	JAK2	0.205	0	-0.248278938
PTEN	C5orf35	0.205	0	-0.115325827
PTEN	BHLHE40	0.205	0	0.22111874
PTEN	NME5	0.205	0	-0.010086023
PTEN	RNF212	0.205	0	-0.041149894
PTEN	ATXN7	0.205	0	-0.337161164
PTEN	RIT1	0.205	0	-0.036158901
PTEN	SGMS2	0.205	0	-0.250092346
PTEN	PLCXD3	0.205	0	-0.472923611
PTEN	CS	0.205	0	0.138626269
PTEN	PIGM	0.205	0	0.074044648
PTEN	PCDHB14	0.206	0	-0.340887074
PTEN	AMOT	0.206	0	-0.215811566
PTEN	RAB28	0.206	0	-0.033008733
PTEN	C3orf64	0.206	0	-0.30270781

PTEN	PTH1R	0.206	0	-0.239175855
PTEN	SLC26A5	0.206	0	-0.403379398
PTEN	SCFD1	0.206	0	-0.023213075
PTEN	SNORD116-4	0.206	0	-5.23E-05
PTEN	RPS6KB1	0.206	0	0.073211422
PTEN	MYB	0.206	0	-0.165105657
PTEN	DHX15	0.206	0	-0.191933533
PTEN	HERC2	0.206	0	-0.011246462
PTEN	ANKRD44	0.206	0	-0.014203817
PTEN	GLI2	0.206	0	-0.020139638
PTEN	MFAP1	0.206	0	-0.000964322
PTEN	ABCB4	0.206	0	-0.026779319
PTEN	TOMM20L	0.206	0	-0.026121214
PTEN	CCDC25	0.206	0	-0.073799332
PTEN	RSL24D1	0.207	0	0.021312049
PTEN	C1orf161	0.207	0	-0.034195839
PTEN	TRO	0.207	0	0.138134185
PTEN	C5	0.207	0	-0.18520932
PTEN	ACACB	0.207	0	-0.011612692
PTEN	CBR4	0.207	0	-0.175896368
PTEN	RPS6KA2	0.207	0	0.059093653
PTEN	CALCOCO1	0.207	0	0.039845412
PTEN	TRIP4	0.207	0	-0.058933596
PTEN	HNRNPR	0.207	0	-0.188431741
PTEN	RNF168	0.207	0	-0.007436327
PTEN	ALS2	0.207	0	-0.032358771
PTEN	ETFDH	0.207	0	-0.154450408
PTEN	FHAD1	0.207	0	-0.051304736
PTEN	LRFN5	0.207	0	-0.392253309
PTEN	ITCH	0.207	0	-0.21771726
PTEN	KIAA1324	0.207	0	0.008340357
PTEN	CCDC52	0.207	0	-0.136894932
PTEN	C11orf58	0.207	0	0.175455855
PTEN	RET	0.207	0	-0.074315961
PTEN	PDS5A	0.207	0	0.108282309
PTEN	MLL2	0.207	0	-0.009692304
PTEN	ACOX1	0.207	0	-0.124905987
PTEN	ZNF655	0.207	0	-0.011691798
PTEN	PICALM	0.207	0	0.322124322
PTEN	SNORD45A	0.207	0	-0.027602425
PTEN	BAT2L2	0.208	0	0.094531167

PTEN	TRAF5	0.208	0	-0.00927698
PTEN	OLFML2B	0.208	0	-0.040110423
PTEN	ZNF808	0.208	0	0.008031244
PTEN	LPPR5	0.208	0	-0.681158461
PTEN	EP400	0.208	0	-0.117935905
PTEN	P4HA3	0.208	0	-0.253486462
PTEN	NCOA2	0.208	0	-0.079259692
PTEN	COPZ2	0.208	0	0.000646845
PTEN	FOXO3	0.208	0	0.069895287
PTEN	MFAP4	0.208	0	-0.071169806
PTEN	FUBP3	0.208	0	0.02306095
PTEN	CNNM2	0.208	0	-0.180700407
PTEN	HOXC6	0.208	0	-0.145009242
PTEN	TBC1D2B	0.208	0	0.16565232
PTEN	SC5DL	0.208	0	-0.219245296
PTEN	P704P	0.208	0	-0.064338792
PTEN	LONRF3	0.208	0	-0.051355423
PTEN	AXIN2	0.208	0	-0.05798592
PTEN	SHE	0.208	0	-0.187853216
PTEN	DUSP6	0.208	0	-0.057031537
PTEN	C10orf107	0.208	0	-0.02102756
PTEN	C11orf46	0.208	0	-0.014549967
PTEN	SETD3	0.208	0	0.166244865
PTEN	ISCA1	0.208	0	-0.130112606
PTEN	C9orf93	0.208	0	-0.00398437
PTEN	GNG2	0.208	0	-0.357365021
PTEN	CADM2	0.209	0	-0.546944146
PTEN	FGD5	0.209	0	0.089707364
PTEN	MBLAC2	0.209	0	-0.643670739
PTEN	RNF180	0.209	0	-0.344274159
PTEN	AMIGO2	0.209	0	-0.134128991
PTEN	PAN3	0.209	0	-0.361717268
PTEN	PCNP	0.209	0	0.202764778
PTEN	IQCA1	0.209	0	-0.200583768
PTEN	COPS4	0.209	0	-0.17134164
PTEN	ARNT2	0.209	0	-0.002517955
PTEN	PIK3R3	0.209	0	-0.003997685
PTEN	ZG16B	0.209	0	0.072436061
PTEN	B3GALTL	0.209	0	-0.321634249
PTEN	PAX9	0.209	0	-0.453810619
PTEN	FRMPD2	0.209	0	-0.010522909

PTEN	THSD1	0.209	0	-0.015073371
PTEN	WDR52	0.209	0	-0.00481039
PTEN	RAB6C	0.209	0	-0.195087506
PTEN	CIRBP	0.209	0	0.140037246
PTEN	SSTR1	0.209	0	-0.157202344
PTEN	CLIC4	0.209	0	0.18458667
PTEN	C1QTNF6	0.21	0	-0.069913037
PTEN	OR3A2	0.21	0	-2.07E-06
PTEN	RNF152	0.21	0	-0.296853991
PTEN	TTC26	0.21	0	-0.069774711
PTEN	CLGN	0.21	0	-0.050709867
PTEN	PPP2R5A	0.21	0	-0.022377677
PTEN	ORC4L	0.21	0	-0.022678329
PTEN	LOC152225	0.21	0	-0.76857808
PTEN	FBXO8	0.21	0	-0.351619581
PTEN	BCL2	0.21	0	-0.008219582
PTEN	GPM6A	0.21	0	-0.214296035
PTEN	TSSK1B	0.21	0	-0.056331438
PTEN	TMX3	0.21	0	-0.017324216
PTEN	IGF2R	0.21	0	0.147068108
PTEN	ZFYVE20	0.21	0	-0.379470123
PTEN	TLN2	0.21	0	-0.161274961
PTEN	ZNF92	0.21	0	-0.027102861
PTEN	GNA14	0.21	0	-0.256066338
PTEN	TMEM119	0.21	0	0.012176387
PTEN	ZNF486	0.21	0	-0.020198251
PTEN	CXCL14	0.21	0	0.017796023
PTEN	TP63	0.21	0	-0.19511945
PTEN	ZNF121	0.21	0	-0.378808528
PTEN	RORB	0.21	0	-0.824558384
PTEN	TPK1	0.21	0	-0.181189678
PTEN	YTHDC1	0.211	0	0.079736164
PTEN	KLHL2	0.211	0	-0.431117608
PTEN	SLC26A2	0.211	0	-0.179488389
PTEN	C18orf1	0.211	0	-0.011212022
PTEN	KGFLP1	0.211	0	-0.504279027
PTEN	CXCR7	0.211	0	-0.157688213
PTEN	KIF3B	0.211	0	-0.108513722
PTEN	ECE1	0.211	0	-0.012129424
PTEN	EFEMP1	0.211	0	0.019453377
PTEN	FAM124B	0.211	0	-0.158138458

PTEN	EHD2	0.211	0	-0.002827773
PTEN	KCNJ11	0.211	0	-0.006322164
PTEN	HNRNPK	0.211	0	0.422307303
PTEN	PCDHGA9	0.211	0	-0.0016606
PTEN	ZNFX1	0.211	0	-0.000843087
PTEN	POTEF	0.211	0	-0.189580348
PTEN	PHF20	0.211	0	0.009464103
PTEN	ATP6V1A	0.211	0	-0.049683545
PTEN	KIAA0776	0.211	0	0.141135736
PTEN	KIAA0141	0.211	0	-0.013231334
PTEN	FAS	0.211	0	0.046784584
PTEN	DHX36	0.211	0	0.10704761
PTEN	HAUS2	0.211	0	-0.194436212
PTEN	WDR78	0.212	0	-0.157647872
PTEN	PGM2L1	0.212	0	-0.085697759
PTEN	FAM155A	0.212	0	-0.311368529
PTEN	GSTM3	0.212	0	0.004080986
PTEN	RUNDC3B	0.212	0	-0.45888668
PTEN	POMT2	0.212	0	-0.315154325
PTEN	PPP1R2P3	0.212	0	-0.239372324
PTEN	GREB1	0.212	0	0.062426443
PTEN	SAMD9	0.212	0	-0.048530949
PTEN	MOBKL1B	0.212	0	-0.024763195
PTEN	SLC8A1	0.212	0	-0.424840656
PTEN	AP1AR	0.212	0	0.027319964
PTEN	MAGI2	0.212	0	-0.199826919
PTEN	PHTF2	0.212	0	-0.141934802
PTEN	TMEM132B	0.212	0	-0.447795533
PTEN	BAI3	0.212	0	-0.402747816
PTEN	TIGD6	0.212	0	-0.3162915
PTEN	C6orf170	0.213	0	-0.332370722
PTEN	EPHA3	0.213	0	-0.423267729
PTEN	FAM134B	0.213	0	-0.141955104
PTEN	RHOA	0.213	0	0.085118861
PTEN	APOLD1	0.213	0	-0.012420485
PTEN	RFX3	0.213	0	-0.192709174
PTEN	ASB7	0.213	0	-0.284559743
PTEN	DTX3L	0.213	0	-0.025228034
PTEN	ZNF774	0.213	0	-0.001605805
PTEN	C5orf22	0.213	0	-0.028955075
PTEN	ALMS1	0.213	0	-0.123063193

PTEN	MAP1B	0.213	0	-0.497680625
PTEN	C16orf52	0.213	0	-0.185217914
PTEN	FRG1B	0.213	0	-0.256857009
PTEN	HK1	0.213	0	-0.001811721
PTEN	SPAG9	0.213	0	0.081652377
PTEN	GHITM	0.213	0	0.106861415
PTEN	WDR35	0.213	0	-0.002389598
PTEN	DCTN6	0.213	0	-0.172406699
PTEN	PARM1	0.213	0	-0.011047113
PTEN	GMCL1	0.213	0	-0.174349087
PTEN	TAC1	0.213	0	-0.200538348
PTEN	ATAD2B	0.213	0	-0.196057921
PTEN	SPATA17	0.213	0	-0.002788706
PTEN	C9orf150	0.213	0	-0.018817731
PTEN	CBX5	0.214	0	-1.09E-06
PTEN	CRK	0.214	0	0.014349462
PTEN	ADAM33	0.214	0	-0.27790151
PTEN	CELF1	0.214	0	-0.026496046
PTEN	DYRK2	0.214	0	-0.349646879
PTEN	PCDHB18	0.214	0	-0.130085354
PTEN	EHF	0.214	0	0.13803737
PTEN	CHMP2B	0.214	0	0.065382994
PTEN	IQCJ	0.214	0	-0.50869636
PTEN	IGSF21	0.214	0	-0.001826941
PTEN	HSPA2	0.214	0	-0.202011255
PTEN	TAPT1	0.214	0	-0.199052146
PTEN	DYRK3	0.214	0	-0.01983062
PTEN	MEF2D	0.214	0	0.11401149
PTEN	GOLM1	0.214	0	0.109151544
PTEN	FGD4	0.214	0	-0.185229255
PTEN	TXNIP	0.214	0	0.091999068
PTEN	TMEM90B	0.214	0	-0.041559194
PTEN	NEU3	0.214	0	-0.008987844
PTEN	LCMT2	0.214	0	-0.282919341
PTEN	TMED5	0.214	0	-0.063879671
PTEN	C12orf51	0.214	0	-0.058153372
PTEN	CHD4	0.214	0	-0.158842974
PTEN	CD34	0.214	0	0.009663052
PTEN	C8orf79	0.214	0	-0.024812616
PTEN	ICA1	0.214	0	-0.130298988
PTEN	GRM5	0.214	0	-0.558307674

PTEN	TCF7L2	0.215	0	-0.220374567
PTEN	ZSWIM6	0.215	0	-0.511253528
PTEN	SSH2	0.215	0	0.15242864
PTEN	SYDE2	0.215	0	-0.272459551
PTEN	MSR1	0.215	0	-0.165091364
PTEN	FUCA1	0.215	0	0.01141858
PTEN	FAM59A	0.215	0	-0.292481301
PTEN	RTF1	0.215	0	-0.013997288
PTEN	SULF2	0.215	0	0.084699713
PTEN	LIPH	0.215	0	-0.161674808
PTEN	KPNA6	0.215	0	0.157177328
PTEN	PPP2CA	0.215	0	0.119568945
PTEN	KCTD10	0.215	0	-0.018408689
PTEN	UNC5B	0.215	0	-0.090290509
PTEN	CUL4B	0.215	0	-0.066945675
PTEN	ZNF493	0.215	0	-0.374575787
PTEN	CAPS2	0.215	0	-0.021731149
PTEN	DOCK10	0.215	0	-0.119209774
PTEN	ITPR2	0.215	0	-0.225441024
PTEN	FST	0.215	0	-0.026671885
PTEN	TAF1A	0.215	0	-0.168533758
PTEN	LONP2	0.216	0	0.001827467
PTEN	LRRC40	0.216	0	-0.021859752
PTEN	SEMA6A	0.216	0	-0.144636596
PTEN	GANC	0.216	0	0.134290721
PTEN	SPRY4	0.216	0	-0.191094977
PTEN	EEF1A1	0.216	0	0.540326041
PTEN	TMEM233	0.216	0	-0.003457798
PTEN	NBPF14	0.216	0	-0.004525505
PTEN	ADCY9	0.216	0	-0.075119133
PTEN	SLC17A7	0.216	0	-0.0254185
PTEN	BMPER	0.216	0	-0.073097249
PTEN	KAT2B	0.216	0	-0.36507652
PTEN	SUCLG2	0.216	0	0.076158528
PTEN	SLC25A12	0.216	0	-0.223777776
PTEN	CASZ1	0.216	0	-0.334698033
PTEN	INTS2	0.216	0	-0.18788874
PTEN	HIGD1A	0.216	0	-0.12767078
PTEN	DNAJC24	0.217	0	-0.425776348
PTEN	RNF2	0.217	0	-0.31824502
PTEN	TTC5	0.217	0	-0.17199464

PTEN	GNB5	0.217	0	-0.126503182
PTEN	ALDH1A2	0.217	0	-0.392542888
PTEN	TNRC6A	0.217	0	0.112967325
PTEN	RHOJ	0.217	0	-0.172459033
PTEN	DCHS1	0.217	0	-0.056471818
PTEN	PPAPDC1A	0.217	0	-0.004597287
PTEN	KDELC2	0.217	0	0.097237989
PTEN	FAM111A	0.217	0	-0.20746193
PTEN	C2orf55	0.217	0	-0.024344854
PTEN	TBX5	0.217	0	-0.383973841
PTEN	CORO2B	0.217	0	-0.134368343
PTEN	CDR1	0.217	0	-0.000719248
PTEN	BCDIN3D	0.217	0	-0.235486064
PTEN	JMY	0.217	0	-0.300317171
PTEN	BBS1	0.217	0	-0.005907104
PTEN	GPR77	0.217	0	-8.05E-05
PTEN	SLC16A4	0.217	0	-0.095780006
PTEN	GRID1	0.217	0	-0.139502346
PTEN	TNFSF4	0.217	0	-0.316923019
PTEN	RFX8	0.217	0	-0.190174489
PTEN	KIAA0754	0.217	0	-0.174214072
PTEN	C2orf14	0.217	0	-0.187689622
PTEN	MPHOSPH9	0.217	0	-0.560281791
PTEN	NOTCH2NL	0.217	0	0.016747441
PTEN	PPM1H	0.217	0	-0.087311454
PTEN	GPBP1	0.217	0	-0.239754249
PTEN	REV1	0.217	0	0.004856236
PTEN	PDCD4	0.218	0	-0.161879229
PTEN	IL1R1	0.218	0	-0.050094129
PTEN	EFCAB4B	0.218	0	-0.019933914
PTEN	TBK1	0.218	0	-0.066592059
PTEN	TMEM98	0.218	0	-0.019058264
PTEN	NEK11	0.218	0	-0.027094144
PTEN	RHOBTB2	0.218	0	-0.282526188
PTEN	C9orf82	0.218	0	-0.340589146
PTEN	DDX5	0.218	0	-0.01223164
PTEN	WASF2	0.218	0	0.03188716
PTEN	FTSJD1	0.218	0	-0.17928526
PTEN	CCAR1	0.218	0	0.040928689
PTEN	IMPACT	0.218	0	-0.035104266
PTEN	MTM1	0.218	0	-0.379145498

PTEN	KIFAP3	0.218	0	-0.14848134
PTEN	RNF115	0.218	0	-0.041516364
PTEN	LIN7C	0.218	0	-0.392737626
PTEN	MDM1	0.219	0	-0.545177599
PTEN	ANKRD43	0.219	0	-0.074625081
PTEN	TBC1D23	0.219	0	-0.059969358
PTEN	WARS2	0.219	0	-0.17781486
PTEN	PIGH	0.219	0	-0.014147358
PTEN	SGPP1	0.219	0	-0.181012962
PTEN	C7orf63	0.219	0	-0.214707611
PTEN	F2RL1	0.219	0	-0.18209932
PTEN	RBM45	0.219	0	-0.113275652
PTEN	CCNH	0.219	0	-0.010756636
PTEN	KLHL8	0.219	0	-0.161015611
PTEN	ABCC8	0.219	0	-0.000348292
PTEN	FEZ1	0.219	0	-0.409393516
PTEN	TLL2	0.219	0	0.019862693
PTEN	LOC100128977	0.219	0	-0.006973514
PTEN	ETV3	0.219	0	-0.074476306
PTEN	TMEM5	0.219	0	-6.35E-06
PTEN	LPGAT1	0.219	0	-0.111729159
PTEN	TPO	0.219	0	-0.000624412
PTEN	JAG1	0.22	0	-0.089766453
PTEN	PPP4R1	0.22	0	-0.201214312
PTEN	NOVA1	0.22	0	-0.162630945
PTEN	PRSS54	0.22	0	-0.003601435
PTEN	MSMP	0.22	0	-0.000108298
PTEN	PRPF4B	0.22	0	0.044050039
PTEN	FAM21C	0.22	0	-0.050834104
PTEN	ZNF680	0.22	0	-0.093181683
PTEN	PDLIM5	0.22	0	-0.329714368
PTEN	PRUNE2	0.22	0	-0.054362475
PTEN	MYH10	0.22	0	-0.024157108
PTEN	CAPN13	0.22	0	-0.041209436
PTEN	KLHL24	0.22	0	0.139640562
PTEN	OTUD1	0.22	0	-0.289393648
PTEN	PKD1L1	0.22	0	-0.191000374
PTEN	PDPN	0.22	0	-0.13747076
PTEN	PPP3CA	0.22	0	-0.342504047
PTEN	LEF1	0.22	0	-0.313783599
PTEN	CLN5	0.22	0	-0.02181297

PTEN	SMARCA2	0.22	0	0.037605914
PTEN	LOC728323	0.22	0	-0.200207716
PTEN	GFM1	0.221	0	-0.136302407
PTEN	FAM7A2	0.221	0	-0.109452467
PTEN	SLC19A2	0.221	0	-0.194213047
PTEN	PLEKHA5	0.221	0	-0.082155141
PTEN	FAM116A	0.221	0	-0.269071725
PTEN	LEMD3	0.221	0	-0.319344875
PTEN	SPOP	0.221	0	-0.07747894
PTEN	ITGA4	0.221	0	-0.02973158
PTEN	POLR2A	0.221	0	0.165810478
PTEN	LOC148696	0.221	0	-0.329329986
PTEN	CWF19L2	0.221	0	-0.185412548
PTEN	C9orf44	0.221	0	-0.028462641
PTEN	TLR7	0.221	0	-0.144211503
PTEN	STARD8	0.221	0	-0.229318945
PTEN	PUM2	0.221	0	-0.327025937
PTEN	TUBGCP5	0.221	0	-0.151256426
PTEN	NT5E	0.221	0	0.003764269
PTEN	CEACAM6	0.221	0	0.019455252
PTEN	AMACR	0.221	0	-0.094322264
PTEN	GTF2H2	0.221	0	-0.271353681
PTEN	C2orf16	0.221	0	-0.000131456
PTEN	FGD3	0.221	0	-0.003815661
PTEN	CEACAM5	0.222	0	-0.005243383
PTEN	FHL1	0.222	0	-0.052217519
PTEN	ZFP37	0.222	0	-0.016176876
PTEN	ALDH18A1	0.222	0	-0.020357189
PTEN	PDK4	0.222	0	-0.425216455
PTEN	FAM131B	0.222	0	0.072979849
PTEN	FAM98B	0.222	0	-0.012296096
PTEN	ZNF766	0.222	0	-0.002630584
PTEN	VIPR2	0.222	0	-0.224074724
PTEN	BLZF1	0.222	0	-0.116118747
PTEN	PRSS35	0.222	0	-0.204046108
PTEN	MCTP1	0.222	0	-0.302194921
PTEN	ARL10	0.222	0	-0.197037723
PTEN	PIH1D2	0.222	0	-0.227940244
PTEN	IFT88	0.222	0	-0.012586283
PTEN	BLNK	0.223	0	-0.130350773
PTEN	GPATCH8	0.223	0	-0.198707276

PTEN	ISPD	0.223	0	-0.185930256
PTEN	MMADHC	0.223	0	0.055638434
PTEN	RALGPS1	0.223	0	0.092664766
PTEN	SLC20A1	0.223	0	-0.171243199
PTEN	SFRS12IP1	0.223	0	-0.160334563
PTEN	ZNF100	0.223	0	-0.101320912
PTEN	BCL10	0.223	0	0.134485391
PTEN	TTC17	0.223	0	-4.10E-06
PTEN	AP1S2	0.223	0	-0.063159709
PTEN	IKBKAP	0.223	0	-0.169480092
PTEN	H6PD	0.223	0	0.163605327
PTEN	FNDC3B	0.223	0	-0.093887691
PTEN	CTGF	0.223	0	-0.071046575
PTEN	KCNA6	0.223	0	-0.621114953
PTEN	ZBTB4	0.223	0	-0.067282085
PTEN	HAT1	0.223	0	0.043261533
PTEN	F7	0.223	0	-0.375871558
PTEN	LAMB1	0.223	0	0.018434882
PTEN	LOC100126784	0.224	0	-0.167966375
PTEN	ZNF44	0.224	0	-0.022768707
PTEN	PDE8B	0.224	0	-0.016468119
PTEN	SLC17A5	0.224	0	-0.009246354
PTEN	ABCC12	0.224	0	-0.204684643
PTEN	MMRN1	0.224	0	-0.170535104
PTEN	HSPA13	0.224	0	-0.42270039
PTEN	TTC3	0.224	0	0.000272736
PTEN	TENC1	0.224	0	0.005069656
PTEN	CENPP	0.224	0	-0.158276975
PTEN	LILRA1	0.224	0	-0.022767555
PTEN	ZNF396	0.224	0	-0.020104512
PTEN	PPFIA2	0.224	0	-0.002317038
PTEN	ZNF432	0.224	0	-0.006250088
PTEN	YY1	0.224	0	0.104184093
PTEN	CCDC148	0.224	0	-0.017808255
PTEN	DOCK11	0.224	0	-0.373500754
PTEN	NOL9	0.224	0	-0.014952553
PTEN	DUSP1	0.224	0	-0.061770558
PTEN	LOC646999	0.224	0	-0.367604008
PTEN	KIAA0319L	0.224	0	-0.112528926
PTEN	AGR3	0.224	0	0.158226244
PTEN	BPIL1	0.224	0	-0.010037728

PTEN	MDM2	0.224	0	0.033496284
PTEN	WDR47	0.224	0	-0.223410757
PTEN	DDX6	0.224	0	0.020391346
PTEN	TMBIM4	0.224	0	-0.016558566
PTEN	PDE4A	0.225	0	-0.163810583
PTEN	FOXP2	0.225	0	-0.374524357
PTEN	RASAL2	0.225	0	-0.037248384
PTEN	NCRNA00110	0.225	0	-0.269238387
PTEN	PDZRN3	0.225	0	0.08594923
PTEN	PNMA2	0.225	0	-0.340818493
PTEN	PLEKHM3	0.225	0	-0.010111432
PTEN	CRY1	0.225	0	-0.338124494
PTEN	XPA	0.225	0	-0.007387788
PTEN	HEYL	0.225	0	-0.401459569
PTEN	DCUN1D3	0.225	0	-0.052215953
PTEN	MYL3	0.225	0	-3.75E-06
PTEN	ADD1	0.225	0	0.090455757
PTEN	TSPYL4	0.225	0	0.143502681
PTEN	TMCC1	0.225	0	0.044137718
PTEN	MAP9	0.225	0	-0.106764294
PTEN	UBR4	0.225	0	-0.000543765
PTEN	GIPC2	0.225	0	-0.487023282
PTEN	ZNF671	0.225	0	-0.008458417
PTEN	KHDRBS1	0.225	0	0.066852445
PTEN	C10orf68	0.225	0	-0.355791657
PTEN	GSTO2	0.225	0	-0.000228962
PTEN	EDNRB	0.226	0	-0.687688722
PTEN	RABGAP1	0.226	0	-0.109683792
PTEN	NUCKS1	0.226	0	0.163713947
PTEN	LYRM7	0.226	0	-0.193522381
PTEN	ATP9B	0.226	0	0.033403914
PTEN	KLHDC2	0.226	0	0.042787608
PTEN	THSD7B	0.226	0	-0.449529455
PTEN	GPRIN3	0.226	0	-0.247268078
PTEN	ZNF287	0.226	0	-0.211485546
PTEN	PON2	0.226	0	0.164736635
PTEN	SSH1	0.226	0	-0.039316758
PTEN	PDE4D	0.226	0	-0.048610126
PTEN	C14orf28	0.226	0	-0.195042877
PTEN	SLC22A23	0.227	0	0.157496691
PTEN	ATXN3	0.227	0	-0.24218189

PTEN	MYNN	0.227	0	-0.296051866
PTEN	GABRA2	0.227	0	-0.068858722
PTEN	PTPN4	0.227	0	-0.485526937
PTEN	NT5DC1	0.227	0	-0.119893234
PTEN	KDM5A	0.227	0	0.034305059
PTEN	CYSLTR2	0.227	0	-0.20386879
PTEN	GPR81	0.227	0	-0.157247318
PTEN	NOS1AP	0.227	0	-0.103527158
PTEN	GOLGA8G	0.227	0	-0.592889841
PTEN	ABL1	0.227	0	0.132355305
PTEN	LOC650623	0.227	0	-0.132848223
PTEN	FAM63A	0.227	0	0.017059755
PTEN	ADAR	0.227	0	0.082837355
PTEN	ETAA1	0.227	0	-0.146724882
PTEN	KCNT2	0.227	0	-0.608141198
PTEN	GAPT	0.227	0	-0.008318831
PTEN	PIP4K2B	0.227	0	0.226856517
PTEN	SLC25A40	0.227	0	-0.005229148
PTEN	TRPC6	0.227	0	-0.148728826
PTEN	USP25	0.228	0	-0.230125332
PTEN	SRP9	0.228	0	0.152335915
PTEN	PARP8	0.228	0	-0.508948002
PTEN	TIMP3	0.228	0	0.187560524
PTEN	PECI	0.228	0	0.001542049
PTEN	SIK3	0.228	0	0.030577773
PTEN	EMB	0.228	0	0.016665383
PTEN	UST	0.228	0	-0.334329877
PTEN	TMEM45B	0.228	0	-0.125164803
PTEN	FAM120AOS	0.228	0	-0.019399361
PTEN	NRXN3	0.228	0	-0.263922033
PTEN	USP48	0.228	0	0.113186356
PTEN	RSBN1L	0.228	0	-0.395445688
PTEN	CHD6	0.228	0	-0.034926797
PTEN	TMEM200A	0.228	0	-0.34782089
PTEN	KLHDC10	0.228	0	-0.20282708
PTEN	SHANK1	0.228	0	-0.000351211
PTEN	MAP3K13	0.228	0	-0.448210392
PTEN	FSIP1	0.228	0	-0.158664051
PTEN	ZNF385B	0.229	0	-0.150993551
PTEN	SHPRH	0.229	0	-0.143871351
PTEN	MACC1	0.229	0	0.089469928

PTEN	PRKG2	0.229	0	-0.716702659
PTEN	NUFIP2	0.229	0	0.107556106
PTEN	TMEM215	0.229	0	-0.480297927
PTEN	FCER1A	0.229	0	-0.045278269
PTEN	AP2B1	0.229	0	0.267377976
PTEN	SNW1	0.229	0	-0.000633704
PTEN	ZNF676	0.229	0	-0.046252075
PTEN	PDLIM1	0.229	0	0.00821433
PTEN	THAP6	0.229	0	-0.153010732
PTEN	NDST2	0.229	0	-0.006998945
PTEN	MED7	0.229	0	-0.010278605
PTEN	C10orf93	0.229	0	-0.028486305
PTEN	LHFPL2	0.229	0	0.043575757
PTEN	CPS1	0.229	0	-0.237881001
PTEN	SMAD2	0.229	0	-6.55E-05
PTEN	ASCC3	0.229	0	-0.051040778
PTEN	PPP1R3B	0.229	0	0.118643435
PTEN	RBM22	0.23	0	-0.082543628
PTEN	ORC2L	0.23	0	-0.061392154
PTEN	PGPEP1	0.23	0	-0.000420008
PTEN	HOXD8	0.23	0	-0.194174547
PTEN	TM9SF2	0.23	0	-0.00113468
PTEN	DACH1	0.23	0	-0.276290917
PTEN	ZFHX3	0.23	0	-0.207758018
PTEN	C8orf4	0.23	0	-0.10646922
PTEN	LRRC49	0.23	0	-0.040049948
PTEN	CCRL1	0.23	0	-0.050887043
PTEN	ZNF132	0.23	0	-0.000184019
PTEN	ADAMTSL5	0.23	0	-0.062273552
PTEN	PCDHGA12	0.23	0	-0.00164696
PTEN	CASC1	0.23	0	-0.204846228
PTEN	KCNH1	0.23	0	-0.141902191
PTEN	BAG5	0.23	0	-0.322675851
PTEN	QSER1	0.23	0	-0.014478921
PTEN	GPR107	0.23	0	-0.010404173
PTEN	MEF2C	0.23	0	-0.291003018
PTEN	KIAA0528	0.23	0	-0.394044755
PTEN	PTHLH	0.23	0	-0.248092649
PTEN	ZC3H12B	0.23	0	-0.134775989
PTEN	SIGLEC15	0.231	0	-0.001444538
PTEN	UGT2B28	0.231	0	-0.00060076

PTEN	PDE7B	0.231	0	-0.204462631
PTEN	PFKFB3	0.231	0	-0.036004028
PTEN	ARHGAP32	0.231	0	0.069375002
PTEN	RGPD6	0.231	0	-0.128729304
PTEN	CNGA3	0.231	0	-0.403852408
PTEN	CEP290	0.231	0	-0.015724473
PTEN	PDZD2	0.231	0	-0.323943656
PTEN	IGF2AS	0.231	0	-0.02591776
PTEN	ITGA11	0.231	0	-0.052974103
PTEN	PPP1R15B	0.231	0	-0.060529578
PTEN	NAP1L2	0.231	0	-0.157676029
PTEN	SEC14L2	0.231	0	0.003038792
PTEN	SUV420H1	0.231	0	-0.095091059
PTEN	PIK3CG	0.231	0	-0.202692004
PTEN	CATSPERB	0.231	0	-0.034177761
PTEN	PRKAA1	0.231	0	-0.166830733
PTEN	ABCA8	0.231	0	-0.112480936
PTEN	CPE	0.231	0	0.179645774
PTEN	TROVE2	0.231	0	-0.093373259
PTEN	ZNF487	0.231	0	-0.383319226
PTEN	THRB	0.231	0	-0.034048749
PTEN	CAPN7	0.231	0	-0.089091702
PTEN	LMCD1	0.231	0	-0.009993889
PTEN	SEC16A	0.231	0	0.012461875
PTEN	SPEF2	0.231	0	-0.370010879
PTEN	LMX1B	0.232	0	-0.005304612
PTEN	GLUL	0.232	0	0.006773624
PTEN	THUMPD1	0.232	0	-0.010021832
PTEN	ASTE1	0.232	0	-0.172923835
PTEN	RGAG1	0.232	0	-0.34363462
PTEN	UBXN8	0.232	0	-0.091482844
PTEN	CNTF	0.232	0	-0.332488053
PTEN	ZNF410	0.232	0	-0.006116038
PTEN	B3GALT1	0.232	0	-0.201475432
PTEN	DNAJC22	0.232	0	-0.001654575
PTEN	SLC44A5	0.232	0	-0.270259661
PTEN	TSHZ2	0.232	0	-0.032928905
PTEN	MOSC2	0.232	0	-0.259171516
PTEN	CSRNP2	0.232	0	-0.143911621
PTEN	CA12	0.232	0	0.177428318
PTEN	RRAGC	0.232	0	-0.052336831

PTEN	UBQLN2	0.232	0	-0.048456992
PTEN	SCNN1A	0.232	0	0.000192199
PTEN	ESRRG	0.232	0	-0.441959367
PTEN	KIF3A	0.232	0	-0.277401527
PTEN	PPP2R5C	0.232	0	0.033732778
PTEN	MANBA	0.232	0	-0.056892225
PTEN	NMD3	0.232	0	0.023744088
PTEN	KIF5B	0.232	0	0.112796148
PTEN	CHST8	0.232	0	-0.001007783
PTEN	ZNF20	0.233	0	0.043919786
PTEN	PDCL	0.233	0	-0.052263176
PTEN	SCN4B	0.233	0	-0.257465386
PTEN	RBM7	0.233	0	-0.06066493
PTEN	7-Sep	0.233	0	0.040401944
PTEN	TFF3	0.233	0	0.001186231
PTEN	ALG10B	0.233	0	-0.00554564
PTEN	DGKH	0.233	0	-0.187679378
PTEN	EPS8	0.233	0	-0.13876044
PTEN	KDR	0.233	0	-0.115042111
PTEN	AGL	0.233	0	-0.224657288
PTEN	RAI14	0.233	0	-0.096649301
PTEN	TCN1	0.233	0	-0.039407843
PTEN	TTC14	0.233	0	-0.228336589
PTEN	KRR1	0.233	0	-0.048212141
PTEN	ZBTB33	0.233	0	-0.156720512
PTEN	PEBP4	0.233	0	-0.00011788
PTEN	ZNF860	0.233	0	-0.07078565
PTEN	ELP3	0.233	0	-0.002434691
PTEN	SNX27	0.233	0	-0.090798943
PTEN	TGFBRAP1	0.233	0	0.001435159
PTEN	POLI	0.233	0	-0.021840582
PTEN	NSA2	0.233	0	2.03E-05
PTEN	ERMAP	0.233	0	0.136201391
PTEN	C6orf97	0.234	0	-0.227211575
PTEN	NCRNA00093	0.234	0	-0.600317607
PTEN	PCBP2	0.234	0	0.028718653
PTEN	CAPRIN1	0.234	0	0.407807093
PTEN	NCKAP5	0.234	0	-0.287913093
PTEN	C5orf30	0.234	0	-0.062453609
PTEN	CAMSAP1L1	0.234	0	-0.428315198
PTEN	CDK12	0.234	0	0.077431221

PTEN	CEP68	0.234	0	-0.182002785
PTEN	IL1RL1	0.234	0	-0.337392131
PTEN	SKP1	0.234	0	0.395860101
PTEN	C14orf143	0.234	0	-0.004465707
PTEN	CUL5	0.234	0	-0.078968496
PTEN	MAP2K4	0.234	0	-0.213609864
PTEN	CCDC46	0.234	0	0.110421916
PTEN	TLE3	0.234	0	-0.118165982
PTEN	C14orf148	0.234	0	-0.111234393
PTEN	CELSR1	0.234	0	-0.132702458
PTEN	PCDH17	0.234	0	-0.21218165
PTEN	RHOT1	0.235	0	-0.21212097
PTEN	TXNDC15	0.235	0	-0.051172178
PTEN	SOX5	0.235	0	-0.347648847
PTEN	PCDHB4	0.235	0	-0.344787187
PTEN	CAMLG	0.235	0	-0.161289387
PTEN	ZRANB3	0.235	0	-0.128003537
PTEN	MYO6	0.235	0	-0.17595519
PTEN	ANKRD42	0.235	0	-0.19415318
PTEN	RGS22	0.235	0	0.13197394
PTEN	PAMR1	0.235	0	-0.019260423
PTEN	CYB5D1	0.235	0	-0.098419197
PTEN	CRY2	0.235	0	-0.023245933
PTEN	NEDD9	0.235	0	-0.291858744
PTEN	C9orf68	0.235	0	-0.0565207
PTEN	MCFD2	0.235	0	-0.148557203
PTEN	NR2F1	0.235	0	1.04E-05
PTEN	C3orf38	0.235	0	0.123150936
PTEN	BCAP29	0.235	0	0.12291591
PTEN	TDRD9	0.235	0	-0.014347758
PTEN	ZNF490	0.236	0	-0.105617174
PTEN	STK39	0.236	0	-0.013941591
PTEN	TTC39C	0.236	0	-0.048873811
PTEN	ERBB3	0.236	0	0.188375304
PTEN	CMBL	0.236	0	0.012731259
PTEN	ERGIC1	0.236	0	0.106835825
PTEN	ZBED5	0.236	0	0.002942427
PTEN	NUP98	0.236	0	0.011776767
PTEN	MMP27	0.236	0	-0.005413265
PTEN	ADAMTSL1	0.236	0	-0.115414047
PTEN	LAYN	0.236	0	-0.169883398

PTEN	NEXN	0.236	0	-0.022356997
PTEN	ST8SIA2	0.236	0	-0.193190603
PTEN	SLU7	0.236	0	0.186590604
PTEN	ZNF720	0.236	0	-0.292984229
PTEN	NLGN4X	0.236	0	-0.306220571
PTEN	CDKL3	0.236	0	-0.131164978
PTEN	SFRP4	0.237	0	-0.125400016
PTEN	AFF3	0.237	0	0.032540655
PTEN	RPS6KA6	0.237	0	-0.024299292
PTEN	FIBIN	0.237	0	-0.179603436
PTEN	VAMP4	0.237	0	-0.362835561
PTEN	GPRASP1	0.237	0	-0.066974272
PTEN	TRPC1	0.237	0	-0.383051762
PTEN	NDST1	0.237	0	-0.223260375
PTEN	YAF2	0.237	0	-0.16669793
PTEN	TMEM63C	0.237	0	-0.163023469
PTEN	ARMCX1	0.237	0	-0.063322277
PTEN	BUB3	0.237	0	0.068983707
PTEN	SNIP1	0.237	0	-0.108665105
PTEN	UGT2B11	0.237	0	0.000641225
PTEN	GSPT1	0.237	0	0.163449418
PTEN	EPHA4	0.237	0	-0.35100491
PTEN	UBE2H	0.237	0	-0.154850024
PTEN	PCLO	0.237	0	-0.11537015
PTEN	USP15	0.237	0	0.052440537
PTEN	SDPR	0.237	0	-0.229386695
PTEN	ZNF260	0.237	0	0.03292505
PTEN	AKT3	0.237	0	0.005537491
PTEN	JAM2	0.237	0	-0.167383245
PTEN	TRAM2	0.237	0	-0.00266569
PTEN	MAP1A	0.237	0	-0.021997085
PTEN	DNAJC1	0.237	0	0.001600116
PTEN	MAP3K4	0.238	0	-0.370578397
PTEN	CILP	0.238	0	-0.15073991
PTEN	ZNF17	0.238	0	-0.038666773
PTEN	PRPF40A	0.238	0	0.336035371
PTEN	B4GALT1	0.238	0	-0.144306658
PTEN	LHFP	0.238	0	-0.032221882
PTEN	GNA13	0.238	0	0.043256392
PTEN	ZNF43	0.238	0	-0.07260483
PTEN	SRPX2	0.238	0	-0.004963356

PTEN	SRPX	0.238	0	-0.153866835
PTEN	ZNF366	0.238	0	-0.148252516
PTEN	RPAP3	0.238	0	-0.037972144
PTEN	RFC1	0.238	0	-0.187680318
PTEN	BMPR1B	0.238	0	0.145202966
PTEN	ZNF141	0.238	0	-0.002009282
PTEN	C9orf64	0.238	0	-0.089066467
PTEN	LMAN1	0.238	0	0.128961293
PTEN	RHBDL3	0.238	0	-0.006188714
PTEN	TFCP2	0.238	0	-0.000390516
PTEN	ZNF354C	0.238	0	-0.01084003
PTEN	FBXO4	0.238	0	-0.29901004
PTEN	MSL2	0.238	0	-0.035664538
PTEN	AVL9	0.239	0	0.161541652
PTEN	SMAD3	0.239	0	-0.087023614
PTEN	CCDC85A	0.239	0	-0.250567713
PTEN	TM2D1	0.239	0	-0.149841818
PTEN	FBXO21	0.239	0	-0.097399924
PTEN	MAT2B	0.239	0	-0.008448614
PTEN	ZNF701	0.239	0	-0.164977721
PTEN	KCNE4	0.239	0	-0.151980919
PTEN	RAB27A	0.239	0	-0.091655327
PTEN	MTR	0.239	0	-0.019659196
PTEN	SERPINA11	0.239	0	-0.000107947
PTEN	AFAP1L2	0.239	0	0.142327612
PTEN	SMCR8	0.239	0	-0.01554702
PTEN	ATG16L1	0.239	0	-0.348377018
PTEN	LIMCH1	0.239	0	0.023109934
PTEN	SESN3	0.239	0	-0.143753666
PTEN	ALKBH8	0.239	0	-0.14193336
PTEN	LRRC32	0.239	0	-0.029573678
PTEN	CLSTN2	0.24	0	-0.226992998
PTEN	TMEM144	0.24	0	-0.148857633
PTEN	ASB8	0.24	0	-0.035927327
PTEN	ABHD13	0.24	0	-0.293442167
PTEN	STAT3	0.24	0	0.023028785
PTEN	CCDC65	0.24	0	-0.000108012
PTEN	ZNF395	0.24	0	-0.008793687
PTEN	CAV1	0.24	0	-0.160684037
PTEN	RTN4	0.24	0	0.137390381
PTEN	AOX1	0.24	0	-0.016732253

PTEN	CCDC132	0.24	0	-0.32385371
PTEN	FAM82A1	0.24	0	-0.000120655
PTEN	ZNF709	0.24	0	-0.36632061
PTEN	SLC7A8	0.24	0	-0.116792433
PTEN	NEK4	0.24	0	0.127608804
PTEN	ANKRD32	0.24	0	-0.160301055
PTEN	KIAA1958	0.24	0	-0.184979322
PTEN	DOPEY1	0.24	0	-0.025712954
PTEN	ZNF230	0.24	0	-0.24978085
PTEN	GLT8D1	0.24	0	-7.68E-05
PTEN	TOP2B	0.24	0	-0.011574798
PTEN	SNX1	0.241	0	-0.020074689
PTEN	ADAM22	0.241	0	-0.271386909
PTEN	FER1L6	0.241	0	-0.015959145
PTEN	TRANK1	0.241	0	-0.043206622
PTEN	NEBL	0.241	0	-0.009394637
PTEN	CYP4X1	0.241	0	-0.02353397
PTEN	DAAM2	0.241	0	-0.036878204
PTEN	ZNF445	0.241	0	-0.243061339
PTEN	ZFC3H1	0.241	0	-0.003253929
PTEN	PPT1	0.241	0	0.220824697
PTEN	YIPF6	0.241	0	-0.165910332
PTEN	FN1	0.241	0	0.078319572
PTEN	C9orf125	0.241	0	0.009481623
PTEN	VEGFC	0.241	0	-0.00459605
PTEN	SERINC3	0.241	0	0.000831367
PTEN	UFSP2	0.242	0	-0.131848687
PTEN	ARHGAP24	0.242	0	-0.162216416
PTEN	RIC8B	0.242	0	-0.359927629
PTEN	ASTN2	0.242	0	2.87E-05
PTEN	CLPX	0.242	0	-0.005171741
PTEN	POLR3A	0.242	0	-0.270899672
PTEN	ZNF516	0.242	0	-0.197004468
PTEN	ZNF845	0.242	0	-0.030038048
PTEN	HIVEP3	0.242	0	-0.000453341
PTEN	KRBA2	0.242	0	-0.169819116
PTEN	SLC2A10	0.242	0	-0.008192621
PTEN	AK3	0.242	0	-0.007701389
PTEN	ARMCX2	0.242	0	-0.225501468
PTEN	RHOBTB1	0.242	0	-0.157764531
PTEN	GSK3B	0.242	0	-0.157476331

PTEN	ZNF570	0.242	0	-0.007292005
PTEN	C6orf120	0.242	0	-0.001974707
PTEN	LRP6	0.242	0	0.097527152
PTEN	ASAM	0.242	0	-0.011293387
PTEN	EFHA2	0.242	0	-0.244173445
PTEN	FAM177B	0.242	0	-0.162645735
PTEN	11-Sep	0.243	0	-0.137651837
PTEN	CLTC	0.243	0	0.355565928
PTEN	NEK7	0.243	0	-0.035768443
PTEN	DBX2	0.243	0	-0.233637335
PTEN	RBMXL1	0.243	0	-0.590228728
PTEN	CAPN2	0.243	0	-0.228127054
PTEN	SAPS3	0.243	0	0.030469281
PTEN	GLCCI1	0.243	0	0.086458104
PTEN	ITSN2	0.243	0	-0.284858894
PTEN	DOCK5	0.243	0	0.121533258
PTEN	CORO2A	0.243	0	-0.051831447
PTEN	IPO7	0.243	0	-0.297675144
PTEN	GRP	0.243	0	-0.04552667
PTEN	INSR	0.243	0	-0.018965356
PTEN	VPS41	0.243	0	0.139111499
PTEN	NUBPL	0.243	0	0.007336134
PTEN	DYNC1H1	0.243	0	0.063081891
PTEN	EBF1	0.243	0	-0.329457738
PTEN	HHEX	0.243	0	-0.354858095
PTEN	SENP1	0.243	0	-0.234369495
PTEN	PLXDC2	0.243	0	-0.40070601
PTEN	TSPAN15	0.243	0	0.04306789
PTEN	SH3BGRL2	0.243	0	-0.31695824
PTEN	RBM47	0.243	0	-0.10641669
PTEN	ANKAR	0.243	0	-5.28E-05
PTEN	SCN9A	0.244	0	-0.225224209
PTEN	SPRY1	0.244	0	-0.317604334
PTEN	API5	0.244	0	0.208434523
PTEN	POTEC	0.244	0	-0.234036013
PTEN	ZNF562	0.244	0	-0.042002768
PTEN	EIF2A	0.244	0	0.01442127
PTEN	DNAJC12	0.244	0	-0.012390195
PTEN	C12orf72	0.244	0	0.012136776
PTEN	GOLGA5	0.244	0	-0.01525068
PTEN	ZNF780B	0.244	0	-0.122247048

PTEN	CYP26A1	0.244	0	-0.040959204
PTEN	TMEM22	0.244	0	0.001053572
PTEN	BTF3	0.244	0	0.160753445
PTEN	SORCS1	0.244	0	-0.51223792
PTEN	MYLIP	0.244	0	-0.375731292
PTEN	LIMS3-LOC440895	0.244	0	-0.3236883
PTEN	STIM2	0.244	0	-0.243781914
PTEN	DKK2	0.244	0	-0.38157471
PTEN	TMEM87A	0.244	0	0.330898136
PTEN	KBTBD3	0.244	0	-0.272818252
PTEN	TRAF3IP1	0.244	0	-0.373768632
PTEN	SLC39A6	0.245	0	0.231359582
PTEN	IRS1	0.245	0	-0.217963301
PTEN	ZNF828	0.245	0	-0.066830462
PTEN	MYH13	0.245	0	-0.000219685
PTEN	STEAP2	0.245	0	-0.016560694
PTEN	RBAK	0.245	0	-0.309924064
PTEN	LOC145837	0.245	0	-0.141047301
PTEN	PPP1R2	0.245	0	0.023211874
PTEN	KIAA1217	0.245	0	-0.01103702
PTEN	IKZF2	0.245	0	-0.35610713
PTEN	ABCB7	0.245	0	-0.088831481
PTEN	TARSL2	0.245	0	-0.392927245
PTEN	SHISA2	0.245	0	-0.168250322
PTEN	PPWD1	0.245	0	-0.03578422
PTEN	RAG1	0.245	0	-0.425516071
PTEN	CCDC158	0.245	0	-0.019437567
PTEN	ZNF528	0.245	0	-0.271255862
PTEN	ERAP1	0.245	0	0.053315818
PTEN	HIVEP1	0.245	0	0.024109322
PTEN	KIAA1143	0.245	0	-0.050011792
PTEN	WIPF2	0.245	0	0.018152162
PTEN	PKNOX1	0.245	0	-0.252506242
PTEN	SCGB1D2	0.245	0	0.003872499
PTEN	FAM199X	0.245	0	0.074328647
PTEN	WDR20	0.245	0	-0.09760352
PTEN	TMCO3	0.246	0	-0.219394005
PTEN	ZDHHC6	0.246	0	-0.194667718
PTEN	CCDC152	0.246	0	0.148703837
PTEN	DYNLT3	0.246	0	-0.052066793
PTEN	PTPLAD2	0.246	0	-0.090454132

PTEN	ANKRD40	0.246	0	-0.311051521
PTEN	TGFBR1	0.246	0	-0.16182119
PTEN	LOC653653	0.246	0	-0.270125046
PTEN	TCEAL1	0.246	0	-0.17037219
PTEN	LRRN3	0.246	0	-0.026280879
PTEN	HHAT	0.246	0	-0.084339181
PTEN	CYS1	0.246	0	-0.004890585
PTEN	EIF2C3	0.246	0	-0.053105184
PTEN	EHHADH	0.246	0	-0.291857879
PTEN	KIAA0240	0.246	0	-0.540269301
PTEN	WLS	0.246	0	-0.180594621
PTEN	RASA2	0.246	0	-0.293395992
PTEN	PAM	0.246	0	0.051384806
PTEN	UBLCP1	0.246	0	-0.275334798
PTEN	ZNF37A	0.246	0	-0.124703295
PTEN	THAP5	0.246	0	-0.070493104
PTEN	RBM16	0.246	0	-0.290833749
PTEN	C10orf78	0.246	0	-0.011916058
PTEN	SNORD116-28	0.246	0	-0.157645312
PTEN	ARHGAP18	0.246	0	-0.088454978
PTEN	RSPH3	0.247	0	-0.016521638
PTEN	F8	0.247	0	-0.12370838
PTEN	FBXO36	0.247	0	-0.038148893
PTEN	MN1	0.247	0	-0.403609062
PTEN	ZNF621	0.247	0	-0.00895215
PTEN	NAPEPLD	0.247	0	-0.087394844
PTEN	PER3	0.247	0	-0.003273636
PTEN	DOCK9	0.247	0	0.01075107
PTEN	TEP1	0.247	0	-0.103148455
PTEN	PTPN13	0.247	0	-0.007512088
PTEN	CTBS	0.247	0	-0.559101951
PTEN	LATS2	0.247	0	-0.49519343
PTEN	NFAT5	0.247	0	0.151262567
PTEN	DIRAS3	0.247	0	0.001815548
PTEN	ABCA12	0.247	0	-0.143955587
PTEN	SLC10A7	0.247	0	-0.146901354
PTEN	ZNF236	0.247	0	-0.241829279
PTEN	FAM69A	0.247	0	-0.020663347
PTEN	SCYL3	0.247	0	-0.039676104
PTEN	ANKRD17	0.247	0	0.00497371
PTEN	ENC1	0.247	0	0.023807332

PTEN	RNF103	0.247	0	-0.072391097
PTEN	COL1A1	0.247	0	0.046912871
PTEN	ZBTB34	0.247	0	-0.261505801
PTEN	RBM18	0.247	0	-0.145130466
PTEN	PMP22	0.248	0	0.009850901
PTEN	FAM21A	0.248	0	-0.188446066
PTEN	FBXL7	0.248	0	-0.168244597
PTEN	DDX24	0.248	0	0.060909348
PTEN	IL1RAP	0.248	0	-0.507711594
PTEN	ZNF283	0.248	0	-0.008133714
PTEN	CHN2	0.248	0	-0.455489437
PTEN	LMOD1	0.248	0	-0.167185885
PTEN	P2RY1	0.248	0	-0.38743914
PTEN	DCUN1D1	0.248	0	-0.289315477
PTEN	CYP21A2	0.248	0	-0.001367058
PTEN	LRRC58	0.248	0	-0.025822112
PTEN	HEXB	0.248	0	-0.020845746
PTEN	AOX2P	0.248	0	-0.309380036
PTEN	PHF15	0.248	0	-0.208752725
PTEN	AIDA	0.248	0	0.113776403
PTEN	C4orf29	0.248	0	-0.119749449
PTEN	LRP10	0.248	0	-0.000974522
PTEN	NBR1	0.249	0	0.274225089
PTEN	TK2	0.249	0	-0.304399712
PTEN	NFKB1	0.249	0	-0.170666052
PTEN	TOPORS	0.249	0	-0.49813048
PTEN	PRR5L	0.249	0	-0.109719261
PTEN	TEK	0.249	0	-0.340166701
PTEN	MTF1	0.249	0	-0.221366056
PTEN	GTF2H2C	0.249	0	-0.237734454
PTEN	IFIT5	0.249	0	-0.078357964
PTEN	GRPR	0.249	0	-0.009992548
PTEN	EGFLAM	0.249	0	-0.104596466
PTEN	PDIK1L	0.249	0	-0.173138538
PTEN	PLEKHA3	0.249	0	-0.052258465
PTEN	CTCF	0.249	0	0.164249828
PTEN	TMEM161B	0.249	0	-0.190481076
PTEN	GSR	0.249	0	0.031874559
PTEN	TMEM66	0.249	0	0.025285366
PTEN	ASAH2	0.25	0	-0.021152091
PTEN	ADNP2	0.25	0	-0.258481987

PTEN	FLT1	0.25	0	0.141458794
PTEN	ABI2	0.25	0	-0.164883048
PTEN	LOC100129550	0.25	0	-0.093081935
PTEN	ZNF790	0.25	0	-0.39589023
PTEN	ISM1	0.25	0	-0.36740481
PTEN	SNAI2	0.25	0	-0.262241383
PTEN	CACNA1D	0.25	0	-0.22918484
PTEN	TCTN2	0.25	0	-0.138765439
PTEN	ROCK2	0.25	0	-0.221403122
PTEN	TMEM62	0.25	0	0.021520201
PTEN	COL5A1	0.25	0	0.043914955
PTEN	ADAMTS16	0.25	0	-0.042492719
PTEN	STRN	0.25	0	-0.505745007
PTEN	USP32	0.251	0	-0.137215195
PTEN	ZCCHC4	0.251	0	-0.072986381
PTEN	LOC344595	0.251	0	-0.527361373
PTEN	DENND1B	0.251	0	-0.021251477
PTEN	KCTD20	0.251	0	-0.031206503
PTEN	PRKAR2A	0.251	0	-0.083806465
PTEN	COL8A2	0.251	0	-0.000201588
PTEN	TOX3	0.251	0	-0.343679172
PTEN	ERCC8	0.251	0	-0.151207114
PTEN	CYSLTR1	0.251	0	-0.008339722
PTEN	ARID1B	0.251	0	-0.179866154
PTEN	PDCD6IP	0.251	0	-0.150798415
PTEN	SORCS2	0.251	0	-0.001252494
PTEN	C9orf129	0.251	0	-0.003503644
PTEN	HAS2	0.251	0	-0.306722559
PTEN	TMEM135	0.251	0	0.056928769
PTEN	USP24	0.251	0	-0.271492287
PTEN	GNRHR	0.251	0	-0.475044592
PTEN	SFXN2	0.251	0	-0.212483963
PTEN	GHR	0.251	0	-0.164455182
PTEN	ARRDC3	0.252	0	-0.014444378
PTEN	GRLF1	0.252	0	-0.152976913
PTEN	LEO1	0.252	0	-0.062210087
PTEN	CD164	0.252	0	0.05485683
PTEN	FOSL2	0.252	0	0.009958153
PTEN	GREM1	0.252	0	-0.105213225
PTEN	SSR3	0.252	0	0.063429399
PTEN	MORF4L1	0.252	0	-0.121730293

PTEN	C15orf38	0.252	0	-0.089214994
PTEN	FARP2	0.252	0	-0.000125101
PTEN	MYO5A	0.252	0	0.14196809
PTEN	NHLRC3	0.252	0	-0.478147332
PTEN	CRAT	0.252	0	5.49E-05
PTEN	AZI2	0.252	0	0.078330423
PTEN	UGT2B15	0.252	0	-0.161036112
PTEN	LPAR6	0.252	0	-0.067619247
PTEN	IGFBP5	0.252	0	0.082465126
PTEN	TXNDC16	0.253	0	-0.238111205
PTEN	GABRB2	0.253	0	-0.617720964
PTEN	TRIM34	0.253	0	-0.289305109
PTEN	UGGT2	0.253	0	-0.029797428
PTEN	ZNF347	0.253	0	-0.060805733
PTEN	MCCC2	0.253	0	0.195471647
PTEN	BZW1	0.253	0	0.00054194
PTEN	CDC37L1	0.253	0	-0.195466671
PTEN	IPP	0.253	0	-0.000372445
PTEN	DLC1	0.253	0	-0.194505562
PTEN	ZNF417	0.253	0	-0.126273453
PTEN	ZNF792	0.253	0	-0.052666743
PTEN	SLITRK6	0.253	0	-0.141250971
PTEN	XRCC5	0.253	0	0.012808261
PTEN	THSD4	0.253	0	-0.037958455
PTEN	CP110	0.253	0	-0.142490172
PTEN	C11orf30	0.253	0	-0.452396221
PTEN	CDC14B	0.253	0	-0.336966939
PTEN	F2RL2	0.253	0	-0.179535541
PTEN	DNAJC18	0.254	0	-0.056750482
PTEN	ENPEP	0.254	0	-0.508110485
PTEN	FCF1	0.254	0	-0.051947747
PTEN	PLEKHB2	0.254	0	0.145582477
PTEN	AASS	0.254	0	-0.430674091
PTEN	DLG1	0.254	0	-0.315334386
PTEN	KBTBD10	0.254	0	-0.295753912
PTEN	AK5	0.254	0	-0.171892688
PTEN	DPP4	0.254	0	-0.307939612
PTEN	C15orf33	0.254	0	-0.02587814
PTEN	PER2	0.254	0	-0.043550394
PTEN	KCND3	0.254	0	-0.005920612
PTEN	SYNPO2	0.254	0	-0.30458911

PTEN	BBS12	0.254	0	-0.041142092
PTEN	DCAF10	0.254	0	-0.030059096
PTEN	TSNAX	0.254	0	-0.109973669
PTEN	ZMIZ1	0.254	0	0.00753204
PTEN	TFAM	0.254	0	-0.027942971
PTEN	NRG1	0.254	0	-0.549254172
PTEN	KAZALD1	0.254	0	-0.091441555
PTEN	ZNF33A	0.254	0	-0.161310977
PTEN	COG5	0.254	0	-0.246269228
PTEN	CBLB	0.254	0	0.01574301
PTEN	JUB	0.255	0	-0.450446925
PTEN	MYH8	0.255	0	-0.019334406
PTEN	ZNF441	0.255	0	-0.314176869
PTEN	SEMA3E	0.255	0	-0.335307476
PTEN	ZNF518A	0.255	0	-0.263111771
PTEN	REPS2	0.255	0	-0.198742407
PTEN	ZNF721	0.255	0	-0.452808466
PTEN	GALNT6	0.255	0	-0.127940558
PTEN	TMEM133	0.255	0	-0.587966994
PTEN	ABCD3	0.255	0	-0.000352303
PTEN	CSRNP3	0.255	0	-0.394658474
PTEN	LIPJ	0.255	0	-0.163542182
PTEN	LAMB2	0.256	0	0.009728335
PTEN	PCDH18	0.256	0	-0.308389521
PTEN	CT62	0.256	0	-0.219979672
PTEN	SYTL4	0.256	0	-0.01280252
PTEN	CCNT2	0.256	0	-0.400288255
PTEN	PRKAB2	0.256	0	-0.222894297
PTEN	SLC16A7	0.256	0	-0.382729383
PTEN	ZNF608	0.256	0	-0.053184101
PTEN	UTP15	0.256	0	-0.278289351
PTEN	GEMIN5	0.256	0	-0.006825902
PTEN	DR1	0.256	0	0.125071955
PTEN	ANK3	0.256	0	-0.449593169
PTEN	DCLK1	0.256	0	-0.152113982
PTEN	SYNJ1	0.256	0	-0.141782812
PTEN	ABCC11	0.256	0	-0.021120072
PTEN	OTUD4	0.256	0	-0.158188747
PTEN	ZNF652	0.256	0	-0.001541014
PTEN	ANKRD13C	0.256	0	-0.433744284
PTEN	SULF1	0.256	0	0.109943236

PTEN	FEZ2	0.256	0	-0.177144089
PTEN	RTTN	0.256	0	-0.13113063
PTEN	NAA35	0.256	0	-0.051283235
PTEN	RIOK2	0.256	0	-0.180946295
PTEN	ADCYAP1R1	0.256	0	-3.71E-06
PTEN	GSTM5	0.256	0	-0.000212413
PTEN	C1orf26	0.256	0	-0.03920292
PTEN	NCEH1	0.256	0	0.043277316
PTEN	FAM7A3	0.257	0	-0.10579197
PTEN	KIAA1704	0.257	0	-0.082746947
PTEN	ZNF295	0.257	0	-0.311187669
PTEN	ECD	0.257	0	-0.004134502
PTEN	ATP8B2	0.257	0	0.010077743
PTEN	MFAP5	0.257	0	-0.011603989
PTEN	KLHL3	0.257	0	-0.32581901
PTEN	BBS9	0.257	0	-0.176120659
PTEN	KPNA5	0.257	0	-0.17043939
PTEN	ZMYM5	0.257	0	-0.447722877
PTEN	TGFBR3	0.257	0	-0.257485574
PTEN	ZNF658	0.257	0	-0.042296249
PTEN	TNN	0.257	0	-0.013079968
PTEN	SLC30A9	0.257	0	-0.038489827
PTEN	CD109	0.257	0	-0.339810251
PTEN	ITGA8	0.257	0	-1.71E-07
PTEN	ARHGAP19	0.258	0	-0.131873016
PTEN	SCGB2A2	0.258	0	0.167080255
PTEN	C15orf50	0.258	0	-0.186719665
PTEN	LOH3CR2A	0.258	0	-0.030874218
PTEN	SLC4A8	0.258	0	-0.284091497
PTEN	TPP1	0.258	0	-0.031649029
PTEN	ARL5A	0.258	0	-0.158090421
PTEN	COG3	0.258	0	-0.245570399
PTEN	PCYOX1	0.258	0	0.072173314
PTEN	ARHGAP26	0.258	0	0.155500584
PTEN	SYBU	0.258	0	-0.030161471
PTEN	ZNF644	0.258	0	0.040898436
PTEN	WDR48	0.258	0	0.009056866
PTEN	MS4A2	0.258	0	-0.247749552
PTEN	EXD2	0.258	0	0.104405962
PTEN	GPR1	0.258	0	-0.197096153
PTEN	FBXO11	0.258	0	-0.23377616

PTEN	ZNF423	0.258	0	-0.459445065
PTEN	CDKN2AIP	0.258	0	-0.115415598
PTEN	ROR2	0.258	0	-0.217731508
PTEN	FUBP1	0.258	0	-0.109150855
PTEN	IFNAR1	0.258	0	-0.173930427
PTEN	ZNF268	0.259	0	-0.176376426
PTEN	PAPOLG	0.259	0	-0.059725212
PTEN	TTLL11	0.259	0	-0.247634164
PTEN	C10orf79	0.259	0	-0.140962504
PTEN	PIGB	0.259	0	-0.011260208
PTEN	ANGPTL2	0.259	0	-0.094028604
PTEN	TECPR2	0.259	0	-0.023010286
PTEN	ANKMY2	0.259	0	-0.025375044
PTEN	CTTNBP2	0.259	0	-0.202302061
PTEN	GLUD2	0.259	0	-0.001651146
PTEN	ATF7IP	0.259	0	-0.086303143
PTEN	MYO1D	0.259	0	-0.05074998
PTEN	SPPL2A	0.259	0	-0.132950501
PTEN	SHQ1	0.259	0	-0.193566416
PTEN	FLG	0.259	0	-0.169433115
PTEN	SETD2	0.259	0	-0.185147552
PTEN	SLC16A6	0.259	0	-0.155778765
PTEN	EDA2R	0.259	0	-0.184719353
PTEN	FAM115A	0.259	0	-0.010105735
PTEN	ZNF555	0.259	0	-0.000104824
PTEN	MRPS27	0.259	0	-0.071462408
PTEN	CHD2	0.26	0	0.065637222
PTEN	CPA3	0.26	0	-0.140200458
PTEN	CCDC125	0.26	0	-0.082952181
PTEN	ZNF322A	0.26	0	-0.343528955
PTEN	MECOM	0.26	0	-0.447591358
PTEN	ENTPD3	0.26	0	-0.197029529
PTEN	FBXO34	0.26	0	-0.348108378
PTEN	KDM5B	0.26	0	0.016906536
PTEN	ATL3	0.26	0	-0.113185994
PTEN	ZNF697	0.26	0	-0.20847832
PTEN	C20orf194	0.26	0	-0.127287543
PTEN	ZBTB40	0.26	0	-0.04636667
PTEN	PREPL	0.26	0	0.05139204
PTEN	BAGE2	0.26	0	-0.509657934
PTEN	BTBD8	0.26	0	-0.160522643

PTEN	VWA5A	0.261	0	-0.027053565
PTEN	ITPRIPL2	0.261	0	0.000695596
PTEN	VPS13A	0.261	0	-0.300284509
PTEN	TNFSF10	0.261	0	0.057728324
PTEN	8-Sep	0.261	0	0.135555675
PTEN	RNF141	0.261	0	-0.394451592
PTEN	SPATA18	0.261	0	-0.269554349
PTEN	CLIP1	0.261	0	-0.207989111
PTEN	XBP1	0.261	0	0.109583975
PTEN	ZNF546	0.261	0	-0.150219324
PTEN	SEC31A	0.261	0	-0.01193446
PTEN	TRIM6-TRIM34	0.261	0	-0.332046241
PTEN	ULK4	0.261	0	-0.130556777
PTEN	C1orf9	0.261	0	-0.120728886
PTEN	ZNF609	0.261	0	-0.008179491
PTEN	TP53INP1	0.261	0	0.172677226
PTEN	ZNF776	0.261	0	-0.063042948
PTEN	RLF	0.261	0	-0.124103536
PTEN	ZNF14	0.261	0	-0.016811694
PTEN	SLC12A6	0.262	0	-0.123220131
PTEN	PKD2L2	0.262	0	-0.301270765
PTEN	GRIK3	0.262	0	-0.007926216
PTEN	UGGT1	0.262	0	-0.004600973
PTEN	PEX19	0.262	0	-0.097461099
PTEN	LOC647979	0.262	0	0.027893526
PTEN	ABCG2	0.262	0	-0.458247462
PTEN	KCTD16	0.262	0	-0.88067742
PTEN	RBM41	0.262	0	-0.082660595
PTEN	EXOC8	0.262	0	-0.29053037
PTEN	TRERF1	0.262	0	-0.083778239
PTEN	TFF1	0.262	0	5.33E-05
PTEN	ZDHHC17	0.262	0	0.283740789
PTEN	RNF41	0.262	0	-0.024061574
PTEN	SH3BP4	0.262	0	-0.016664229
PTEN	KLHDC1	0.262	0	-0.454651512
PTEN	YIPF1	0.262	0	-0.17652743
PTEN	TPBG	0.262	0	-0.116602913
PTEN	CNRIP1	0.263	0	-0.130462325
PTEN	SOCS5	0.263	0	-0.306148292
PTEN	PCDHB13	0.263	0	-0.209257156
PTEN	VPS8	0.263	0	-0.000649273

PTEN	CDC73	0.263	0	-0.141493954
PTEN	EBF2	0.263	0	-0.462792037
PTEN	XRN1	0.263	0	-0.37599512
PTEN	RNF144B	0.263	0	0.081036147
PTEN	ANXA7	0.263	0	-0.024092295
PTEN	TTC33	0.263	0	-0.464323749
PTEN	ERBB4	0.263	0	-0.178965836
PTEN	FHL5	0.263	0	-0.061213948
PTEN	HIP1	0.263	0	-0.002422419
PTEN	PGM5	0.263	0	-0.514528266
PTEN	KLHDC7A	0.263	0	-0.006384363
PTEN	SALL2	0.263	0	-0.22117473
PTEN	NUP133	0.263	0	-0.15777326
PTEN	KLHDC5	0.263	0	-0.145408141
PTEN	ABCA1	0.263	0	-0.212033069
PTEN	PCF11	0.264	0	-0.162367501
PTEN	CACNA1C	0.264	0	-0.027536194
PTEN	ZNF772	0.264	0	-0.343985471
PTEN	ZCCHC6	0.264	0	0.028773616
PTEN	SEPSECS	0.264	0	-0.141298802
PTEN	FGF7	0.264	0	-0.322288354
PTEN	NR2F2	0.264	0	-0.082607567
PTEN	XRCC4	0.264	0	-0.322059993
PTEN	THSD7A	0.264	0	-0.212236235
PTEN	SLC30A6	0.264	0	-0.171717101
PTEN	TMEM127	0.264	0	0.008154859
PTEN	PIK3C3	0.264	0	-0.004741562
PTEN	CPEB4	0.265	0	-0.22084689
PTEN	C1orf25	0.265	0	-0.139166063
PTEN	RBBP5	0.265	0	-0.089102233
PTEN	DENND5B	0.265	0	-0.066510386
PTEN	TSC22D1	0.265	0	0.005318227
PTEN	MMAA	0.265	0	-0.391956165
PTEN	GXYLT1	0.265	0	0.118075723
PTEN	NAV1	0.265	0	-0.000497099
PTEN	FAM120B	0.265	0	-0.068099042
PTEN	PBLD	0.265	0	-0.430691191
PTEN	ALCAM	0.265	0	0.114123798
PTEN	SUCLA2	0.265	0	-0.116950539
PTEN	LOC653501	0.265	0	-0.060300483
PTEN	NPAT	0.265	0	-0.78902529

PTEN	SNX6	0.265	0	0.010989195
PTEN	SLC1A7	0.265	0	-0.016439325
PTEN	WDR82	0.265	0	0.298225845
PTEN	TNFAIP6	0.265	0	-0.318690267
PTEN	RSF1	0.265	0	0.06485781
PTEN	ZNF585A	0.265	0	-0.113973078
PTEN	TBC1D8B	0.265	0	-0.402516354
PTEN	FANK1	0.265	0	-0.010502559
PTEN	LDB2	0.265	0	-0.253251739
PTEN	FZD4	0.265	0	-0.34441351
PTEN	CREB3L1	0.265	0	-0.000839774
PTEN	PPAP2B	0.265	0	-0.250185022
PTEN	MAF	0.265	0	-0.055897164
PTEN	NDFIP1	0.266	0	0.075454712
PTEN	MMP2	0.266	0	0.097628027
PTEN	AP4S1	0.266	0	-0.237932536
PTEN	G3BP2	0.266	0	-0.16687887
PTEN	FUT10	0.266	0	-0.108044863
PTEN	ERC1	0.266	0	0.017923018
PTEN	CHRD	0.266	0	-0.131298051
PTEN	MATN3	0.266	0	-0.31283902
PTEN	LZTFL1	0.266	0	-0.159197156
PTEN	ASAH2B	0.266	0	-0.007368332
PTEN	MED1	0.266	0	-0.135042794
PTEN	RPAP2	0.266	0	-0.177604764
PTEN	PROS1	0.266	0	-0.108636341
PTEN	MYCT1	0.266	0	-0.434600185
PTEN	FGF14	0.266	0	-0.125879575
PTEN	CACNA1G	0.266	0	-0.203431016
PTEN	ZC3H11A	0.266	0	0.043525411
PTEN	FAM81B	0.266	0	0.06693454
PTEN	GPATCH2	0.266	0	-0.011892066
PTEN	CHL1	0.266	0	-0.390775883
PTEN	ZNF689	0.266	0	-0.217465433
PTEN	HIAT1	0.266	0	-0.293655493
PTEN	ENTPD4	0.266	0	0.007208633
PTEN	GABPA	0.267	0	-0.144374635
PTEN	TOX4	0.267	0	-0.029595994
PTEN	SLC38A11	0.267	0	-0.012494244
PTEN	SALL1	0.267	0	-0.39915938
PTEN	XPO4	0.267	0	-0.137194371

PTEN	CC2D2A	0.267	0	-0.030102452
PTEN	ABLIM3	0.267	0	-0.347291652
PTEN	ZNF224	0.267	0	-0.032259996
PTEN	ARMCX5	0.267	0	-0.059067604
PTEN	ZNF641	0.267	0	-0.273560575
PTEN	ITM2B	0.267	0	0.273618935
PTEN	ZBTB43	0.267	0	-0.248112247
PTEN	EIF2AK4	0.267	0	-0.034750091
PTEN	RGS4	0.267	0	-0.036679897
PTEN	CX3CR1	0.267	0	-0.17018693
PTEN	HEATR5B	0.267	0	-0.001625469
PTEN	APH1B	0.267	0	-0.420067495
PTEN	EIF2AK3	0.267	0	-0.520853682
PTEN	KATNAL1	0.267	0	-0.04257744
PTEN	FBXW11	0.267	0	-0.319266551
PTEN	NAF1	0.267	0	-0.043200904
PTEN	C4orf41	0.267	0	-0.125447756
PTEN	FOXA1	0.268	0	-0.082868652
PTEN	GPC6	0.268	0	-0.315084738
PTEN	CTNNB1	0.268	0	0.234913831
PTEN	HTR2B	0.268	0	-0.04707979
PTEN	VAV3	0.268	0	0.19814009
PTEN	INHBA	0.268	0	-0.007316266
PTEN	NCAM2	0.268	0	-0.161651587
PTEN	ZFP14	0.268	0	-0.376649994
PTEN	GPC4	0.268	0	-0.182306218
PTEN	NDN	0.268	0	-0.184171587
PTEN	CTR9	0.268	0	-0.169578202
PTEN	KIF27	0.268	0	-0.131203135
PTEN	ATRN	0.268	0	-0.082379898
PTEN	CNTN3	0.268	0	-0.212428786
PTEN	KIRREL	0.268	0	-0.148368508
PTEN	ARHGAP12	0.268	0	-0.250659948
PTEN	CHAD	0.268	0	-0.004841167
PTEN	EIF2C4	0.268	0	-0.284904373
PTEN	CYP2U1	0.268	0	0.073069013
PTEN	SNX18	0.268	0	-0.164015969
PTEN	TSTD2	0.269	0	-0.080433672
PTEN	FANCM	0.269	0	-0.476399714
PTEN	KCNS2	0.269	0	-0.037120313
PTEN	KIAA1328	0.269	0	-0.137934487

PTEN	SPTLC1	0.269	0	-0.129558182
PTEN	NBLA00301	0.269	0	-0.168125032
PTEN	GALNT1	0.269	0	0.160177188
PTEN	MPP7	0.269	0	-0.241059181
PTEN	UBXN4	0.269	0	-0.084997149
PTEN	UBXN10	0.269	0	-0.091972661
PTEN	THPO	0.269	0	-0.135928039
PTEN	EFTUD1	0.269	0	-0.011504749
PTEN	CNOT2	0.269	0	-0.055802596
PTEN	ASPA	0.269	0	-0.049814932
PTEN	SAP30L	0.269	0	-0.05309778
PTEN	THAP2	0.269	0	-0.244615364
PTEN	SYT11	0.269	0	-0.19338058
PTEN	PURA	0.269	0	-0.454581303
PTEN	KITLG	0.269	0	0.164336028
PTEN	EPS15	0.27	0	-0.163511102
PTEN	AHCYL1	0.27	0	0.018976958
PTEN	C9orf80	0.27	0	-0.179599366
PTEN	PRTG	0.27	0	-0.047876579
PTEN	NVL	0.27	0	-0.155411026
PTEN	RECQL	0.27	0	0.135270004
PTEN	FTO	0.27	0	-0.051141774
PTEN	PCDHGB7	0.27	0	0.000161679
PTEN	BAI2	0.27	0	-0.096171652
PTEN	THG1L	0.27	0	-0.02493939
PTEN	KIF2A	0.27	0	-0.316062473
PTEN	TIMP2	0.27	0	0.053025577
PTEN	NOL8	0.27	0	0.041465364
PTEN	DIO2	0.27	0	-0.143640879
PTEN	ECM1	0.271	0	0.001089182
PTEN	IGF1	0.271	0	-0.444775217
PTEN	BTG2	0.271	0	0.030376364
PTEN	IGF2	0.271	0	0.002166904
PTEN	INS-IGF2	0.271	0	0.000153909
PTEN	CPA6	0.271	0	-0.375634895
PTEN	PLXDC1	0.271	0	-0.007342156
PTEN	IPO8	0.271	0	-0.070149358
PTEN	LIFR	0.271	0	0.11785752
PTEN	PTPLB	0.271	0	-0.121365923
PTEN	MED23	0.271	0	-0.152238348
PTEN	DENND5A	0.271	0	-0.180127852

PTEN	ZNF24	0.272	0	-0.008405001
PTEN	FAM126B	0.272	0	-0.015999357
PTEN	USP38	0.272	0	-0.264579788
PTEN	SLC2A13	0.272	0	-0.063784566
PTEN	PNPLA8	0.272	0	-0.058738538
PTEN	MUM1L1	0.272	0	-0.723685528
PTEN	IKZF4	0.272	0	-0.416269961
PTEN	TLR4	0.272	0	-0.296019813
PTEN	PPP2CB	0.272	0	-0.013846741
PTEN	ZNF180	0.272	0	-0.084175724
PTEN	MSI2	0.272	0	0.027941765
PTEN	JAM3	0.272	0	-0.25133234
PTEN	C6orf122	0.272	0	-0.250004242
PTEN	GPD1L	0.272	0	-0.133048131
PTEN	ANKRD34C	0.272	0	-0.325610829
PTEN	SCN2B	0.272	0	-0.143081091
PTEN	TMEM30B	0.273	0	-0.122492364
PTEN	PAFAH1B2	0.273	0	-0.12142078
PTEN	PLN	0.273	0	-0.214328008
PTEN	STS	0.273	0	0.219365747
PTEN	CPSF2	0.273	0	0.068093569
PTEN	TSPYL1	0.273	0	-0.04768539
PTEN	GPRASP2	0.273	0	-0.165861686
PTEN	STK38L	0.273	0	-0.071179582
PTEN	LRRTM2	0.273	0	-0.340549889
PTEN	CREBBP	0.273	0	-0.153114595
PTEN	FOXO1	0.273	0	-0.225304721
PTEN	DPYSL3	0.273	0	0.045370824
PTEN	FAM55C	0.273	0	-0.123116202
PTEN	SYNRG	0.273	0	-0.215710705
PTEN	RPRD2	0.273	0	0.034455992
PTEN	ILDR2	0.273	0	-0.030853262
PTEN	TUBGCP4	0.274	0	-0.183168102
PTEN	GLRB	0.274	0	-0.243725923
PTEN	ABCA9	0.274	0	-0.069959935
PTEN	CTBP2	0.274	0	0.180132023
PTEN	COG6	0.274	0	-0.285728733
PTEN	HP1BP3	0.274	0	0.250102076
PTEN	RPS6KC1	0.274	0	-0.02584841
PTEN	ATP8A2	0.274	0	-0.014283952
PTEN	ZNF223	0.274	0	-0.323427174

PTEN	TBC1D9	0.274	0	0.281785477
PTEN	SLC25A24	0.274	0	0.025683841
PTEN	ZNF333	0.274	0	-0.29359661
PTEN	SLC16A9	0.274	0	-0.202827396
PTEN	PNRC2	0.274	0	0.287857414
PTEN	ARMCX3	0.274	0	-0.061688825
PTEN	RCHY1	0.274	0	0.054741025
PTEN	WDR31	0.274	0	-0.02758504
PTEN	CBARA1	0.274	0	-0.019471443
PTEN	BBS4	0.274	0	0.001114596
PTEN	TRIM13	0.274	0	-0.087690643
PTEN	OSTM1	0.274	0	0.024127412
PTEN	TYW3	0.274	0	-0.082050107
PTEN	ABL2	0.274	0	-0.163144505
PTEN	RANBP3L	0.274	0	-0.371587063
PTEN	SNX30	0.275	0	0.107558836
PTEN	ZBTB37	0.275	0	-0.009606158
PTEN	THBD	0.275	0	-0.178454404
PTEN	PSMD5	0.275	0	0.13387348
PTEN	FRRS1	0.275	0	-0.004616152
PTEN	CHRNA6	0.275	0	-0.048775433
PTEN	GAPVD1	0.275	0	-0.081317666
PTEN	PODN	0.275	0	9.53E-06
PTEN	ZNF638	0.275	0	-0.048035266
PTEN	CUL3	0.275	0	0.082024232
PTEN	TWF1	0.275	0	0.174260783
PTEN	C10orf119	0.275	0	-0.059047699
PTEN	LIMS1	0.275	0	0.140894073
PTEN	TANC1	0.275	0	0.078195693
PTEN	DKK3	0.275	0	-0.241648594
PTEN	C5orf62	0.275	0	-0.099054007
PTEN	RCBTB1	0.276	0	0.102829759
PTEN	ZNF397	0.276	0	-0.230230303
PTEN	RAB5B	0.276	0	0.180261798
PTEN	ARHGAP28	0.276	0	-0.196964995
PTEN	ANKFY1	0.276	0	-0.141307745
PTEN	CALD1	0.276	0	0.424288758
PTEN	EXTL2	0.276	0	-0.143615185
PTEN	GPR160	0.276	0	-0.101280569
PTEN	STX17	0.276	0	-0.174291742
PTEN	TOM1L1	0.276	0	-0.167738703

PTEN	CCDC75	0.276	0	-0.077328911
PTEN	ZC3H7A	0.276	0	-0.20123921
PTEN	SMG1	0.276	0	-0.071361566
PTEN	ZFP90	0.276	0	-0.332044483
PTEN	BBS10	0.276	0	-0.334066702
PTEN	TAF1	0.276	0	-0.015533185
PTEN	MAPK8	0.277	0	-0.000173113
PTEN	TRIM32	0.277	0	0.008225176
PTEN	PTPN21	0.277	0	-0.523560036
PTEN	KCTD12	0.277	0	-0.169851952
PTEN	MCPH1	0.277	0	-0.196519258
PTEN	SIPA1L1	0.277	0	0.001860288
PTEN	ATP7B	0.277	0	-0.206281963
PTEN	NPNT	0.277	0	0.141375454
PTEN	ABCC9	0.277	0	0.171289599
PTEN	AHR	0.277	0	-0.046142592
PTEN	AASDH	0.277	0	-0.016484269
PTEN	RHBDD1	0.277	0	-0.105610047
PTEN	CNOT6L	0.277	0	-0.008232687
PTEN	TMEM30A	0.277	0	-0.043317826
PTEN	C1orf192	0.277	0	-0.294321268
PTEN	ADAMTS6	0.277	0	-0.127645197
PTEN	ADAMTS5	0.277	0	-0.270186493
PTEN	DPY19L1	0.277	0	0.065253171
PTEN	ANKRA2	0.278	0	-0.020417586
PTEN	PCDHGC3	0.278	0	-0.002720453
PTEN	LRP1B	0.278	0	-0.357561728
PTEN	NCKAP1	0.278	0	0.015490668
PTEN	ZNF227	0.278	0	-0.001300595
PTEN	ZNF431	0.278	0	-0.170423027
PTEN	TICAM2	0.278	0	-0.07519621
PTEN	CLCN3	0.278	0	-0.057394801
PTEN	MAPK6	0.278	0	-0.26057781
PTEN	LRP1	0.278	0	0.015222578
PTEN	FAM151B	0.278	0	-0.332376721
PTEN	PARG	0.278	0	-0.213739125
PTEN	SPG20	0.278	0	0.032615759
PTEN	RABGAP1L	0.278	0	-0.153128012
PTEN	CAT	0.278	0	0.119493662
PTEN	SNED1	0.278	0	-0.01521927
PTEN	ITGBL1	0.278	0	-0.000948835

PTEN	C9orf41	0.278	0	-0.18396941
PTEN	KCNRG	0.278	0	-0.260511175
PTEN	BRWD1	0.278	0	0.299022163
PTEN	SLC25A16	0.278	0	-0.378487265
PTEN	DACT1	0.278	0	0.097610908
PTEN	GBE1	0.278	0	-0.019296618
PTEN	DCAF17	0.278	0	0.076827259
PTEN	MIPOL1	0.278	0	-0.275354744
PTEN	GOLGA4	0.278	0	0.161374919
PTEN	MAPK9	0.279	0	-0.12615586
PTEN	C5orf43	0.279	0	0.038146741
PTEN	TMTC1	0.279	0	-0.014563342
PTEN	ZNF568	0.279	0	-0.035209009
PTEN	COPB1	0.279	0	0.071598744
PTEN	TMEM2	0.279	0	-0.177219185
PTEN	LTBP2	0.279	0	0.036267071
PTEN	RGS5	0.279	0	-0.178189603
PTEN	NTM	0.279	0	-0.167930306
PTEN	BMP2K	0.279	0	-0.002141082
PTEN	C11orf87	0.279	0	-0.249689735
PTEN	KIAA1797	0.279	0	-0.129751158
PTEN	KAL1	0.279	0	-0.364750585
PTEN	CD93	0.279	0	-0.159541972
PTEN	CHP	0.279	0	0.129823993
PTEN	KIAA0040	0.279	0	0.232586822
PTEN	RASEF	0.279	0	6.61E-05
PTEN	MAGI3	0.279	0	0.129324478
PTEN	AP4E1	0.279	0	0.119365866
PTEN	STYX	0.279	0	-0.535616953
PTEN	AKAP5	0.28	0	-0.369719615
PTEN	SECISBP2L	0.28	0	-0.21996297
PTEN	SH3GLB1	0.28	0	0.21149825
PTEN	KIAA1012	0.28	0	-0.119178669
PTEN	PIK3R1	0.28	0	-0.23755987
PTEN	VPS39	0.28	0	0.002976215
PTEN	ARHGAP1	0.28	0	0.011972785
PTEN	C5orf44	0.28	0	-0.18422986
PTEN	THBS1	0.28	0	0.073370223
PTEN	LOC402377	0.28	0	-0.027846657
PTEN	MYH1	0.28	0	-0.009719333
PTEN	ZXDB	0.28	0	-0.36156172

PTEN	PKN2	0.28	0	-0.019232756
PTEN	CHSY1	0.28	0	-0.018070551
PTEN	WHAMML2	0.281	0	-0.064925741
PTEN	ALS2CR8	0.281	0	-0.003139569
PTEN	ZEB2	0.281	0	-0.388495606
PTEN	C1orf55	0.281	0	-0.107118406
PTEN	CASP7	0.281	0	-0.205775499
PTEN	FRK	0.281	0	-0.121069075
PTEN	ZNF420	0.281	0	-0.021787815
PTEN	RFTN2	0.281	0	-0.300114028
PTEN	ASAH1	0.281	0	0.138122317
PTEN	NCOR1	0.282	0	0.144239887
PTEN	NOX4	0.282	0	-0.323970958
PTEN	LDB1	0.282	0	0.005731237
PTEN	CGRRF1	0.282	0	-0.312715365
PTEN	PEAR1	0.282	0	-0.008504539
PTEN	10-Sep	0.282	0	-0.057568242
PTEN	PDGFRB	0.282	0	0.170913423
PTEN	COL8A1	0.282	0	-0.001590423
PTEN	NSD1	0.282	0	-0.104883837
PTEN	UBR7	0.282	0	0.024426658
PTEN	C14orf43	0.282	0	-0.045297844
PTEN	RCBTB2	0.282	0	-0.341568422
PTEN	KL	0.282	0	-0.651028658
PTEN	TTLL5	0.282	0	-0.04005166
PTEN	SENP7	0.282	0	-0.582060492
PTEN	DCDC1	0.282	0	-0.15642054
PTEN	NOSTRIN	0.282	0	-0.003494711
PTEN	RCAN2	0.282	0	-0.151729929
PTEN	CXorf23	0.283	0	-0.208536693
PTEN	GCOM1	0.283	0	-0.0637466
PTEN	MAML1	0.283	0	0.125481566
PTEN	KIAA0947	0.283	0	-0.047100879
PTEN	FGF1	0.283	0	-0.223301204
PTEN	LRRC15	0.283	0	0.034136503
PTEN	RAI2	0.283	0	-0.412934719
PTEN	GREM2	0.283	0	-0.691480947
PTEN	RBM12	0.283	0	-0.046563829
PTEN	ZFAND6	0.283	0	-0.18802149
PTEN	CFH	0.283	0	0.019943498
PTEN	HSD17B11	0.283	0	-0.360111994

PTEN	ERN1	0.283	0	-0.193424425
PTEN	C20orf103	0.283	0	-0.151145005
PTEN	HPGDS	0.283	0	-0.504469168
PTEN	RLIM	0.283	0	-5.60E-06
PTEN	SLC26A3	0.283	0	-0.339207145
PTEN	TBL1XR1	0.283	0	0.128608413
PTEN	RAP2C	0.283	0	0.059058824
PTEN	FAM198B	0.283	0	-0.192506438
PTEN	SYTL2	0.283	0	0.025209078
PTEN	C5orf53	0.284	0	-0.330590977
PTEN	HERC3	0.284	0	0.075552987
PTEN	DDX46	0.284	0	-0.037001221
PTEN	CNIH	0.284	0	0.040671828
PTEN	ALKBH1	0.284	0	-0.338543935
PTEN	FAM70A	0.284	0	-0.562073265
PTEN	SH3PXD2A	0.284	0	0.152181275
PTEN	ATP1B1	0.284	0	-0.029672975
PTEN	ENPP4	0.285	0	0.036855032
PTEN	RBM9	0.285	0	-0.056218973
PTEN	APPL1	0.285	0	-0.093060247
PTEN	DSE	0.285	0	-0.219527256
PTEN	MME	0.285	0	-0.277908813
PTEN	LOC100132707	0.285	0	-0.444960884
PTEN	RERE	0.285	0	-0.274755962
PTEN	RNF169	0.285	0	0.095260875
PTEN	SHISA6	0.285	0	-0.004191532
PTEN	ODZ4	0.285	0	-0.030591734
PTEN	MPHOSPH8	0.285	0	-0.003742357
PTEN	BAZ2A	0.285	0	-0.012104152
PTEN	COMMD10	0.286	0	-0.113433866
PTEN	KBTBD7	0.286	0	-0.294776373
PTEN	HTR1F	0.286	0	-0.232578891
PTEN	EEF1DP3	0.286	0	-0.167865489
PTEN	SMAD4	0.286	0	0.010684339
PTEN	NUDT4	0.286	0	0.040048726
PTEN	PTCD2	0.286	0	0.004262065
PTEN	EML1	0.286	0	-0.331544678
PTEN	EDEM1	0.286	0	0.009047268
PTEN	C4A	0.286	0	0.006123728
PTEN	ELTD1	0.286	0	-0.302344113
PTEN	CYP4Z1	0.286	0	-0.015321673

PTEN	RAD17	0.286	0	-0.152239899
PTEN	TLK1	0.286	0	0.101963982
PTEN	SYPL1	0.286	0	0.202016705
PTEN	ARID4B	0.286	0	-0.181673502
PTEN	TRIM44	0.287	0	0.030557476
PTEN	RGPD4	0.287	0	-0.371904198
PTEN	SMNDC1	0.287	0	-0.102635344
PTEN	CCDC39	0.287	0	-0.225137927
PTEN	CTSK	0.287	0	0.087181143
PTEN	EPB41L5	0.287	0	-0.120231484
PTEN	MLLT3	0.287	0	-0.340907568
PTEN	CASC2	0.287	0	-0.201478937
PTEN	BTBD1	0.287	0	0.246725865
PTEN	CPD	0.287	0	-0.020057629
PTEN	NME7	0.287	0	-0.230108736
PTEN	INTU	0.288	0	-0.171800382
PTEN	ZNF234	0.288	0	-0.175464704
PTEN	SDCCAG1	0.288	0	-0.16806781
PTEN	LANCL1	0.288	0	-0.089649104
PTEN	ITGB3	0.288	0	-0.035681891
PTEN	AVPR1A	0.288	0	-0.298777837
PTEN	ANUBL1	0.288	0	-0.049138948
PTEN	EP300	0.288	0	-0.430061118
PTEN	WDR65	0.288	0	-0.015205514
PTEN	CTNNA1	0.288	0	-0.211125438
PTEN	WWC2	0.288	0	-0.543879255
PTEN	ZNF827	0.288	0	-0.171211098
PTEN	RAPGEF2	0.288	0	-0.072467126
PTEN	ZNF474	0.288	0	-0.201447025
PTEN	ZZZ3	0.288	0	0.015707486
PTEN	ITSN1	0.288	0	-0.050523141
PTEN	FNBP1L	0.289	0	0.016280868
PTEN	PPP1R3C	0.289	0	-0.058249576
PTEN	PHIP	0.289	0	-0.20135459
PTEN	C5orf4	0.289	0	0.013301948
PTEN	RUNDC2A	0.289	0	-0.338160758
PTEN	MARVELD2	0.289	0	-0.095111284
PTEN	TRAPPC6B	0.289	0	-0.244762483
PTEN	ZNF597	0.289	0	-0.146328677
PTEN	C1QTNF7	0.289	0	0.011670518
PTEN	CNST	0.289	0	-0.41904347

PTEN	ATP2B4	0.289	0	0.066533082
PTEN	PCDHB12	0.289	0	-0.174898126
PTEN	PLEKHH2	0.289	0	-0.122851005
PTEN	ACADSB	0.289	0	-0.177906098
PTEN	FBXO3	0.289	0	-0.262903041
PTEN	RC3H2	0.289	0	-0.194985375
PTEN	BRWD3	0.289	0	-0.075545614
PTEN	CDC42	0.289	0	0.186971204
PTEN	INPP5A	0.289	0	-0.141434964
PTEN	SHROOM4	0.289	0	-0.288957444
PTEN	SCP2	0.289	0	0.049568936
PTEN	SLMAP	0.289	0	-0.255043434
PTEN	USP37	0.29	0	-0.15585063
PTEN	ZNF624	0.29	0	-0.276889924
PTEN	MAGEL2	0.29	0	-0.01881901
PTEN	STX12	0.29	0	0.045298137
PTEN	FAM149B1	0.29	0	-0.187225457
PTEN	DUSP16	0.29	0	-0.082409957
PTEN	RREB1	0.29	0	-0.13294685
PTEN	STK32B	0.29	0	-0.250719409
PTEN	ACBD3	0.29	0	-0.009607434
PTEN	ANGEL2	0.29	0	-0.206298004
PTEN	DDX50	0.29	0	-0.026778348
PTEN	ERCC6	0.29	0	-0.291078651
PTEN	CRISPLD2	0.29	0	0.030825129
PTEN	ZNF189	0.29	0	-0.261920165
PTEN	TBX18	0.29	0	-0.082716848
PTEN	RPP30	0.291	0	-0.290038996
PTEN	SCYL2	0.291	0	-0.003449209
PTEN	DHX40	0.291	0	0.151492878
PTEN	TM6SF1	0.291	0	-0.145129645
PTEN	TMED8	0.291	0	-0.000500053
PTEN	6-Mar	0.291	0	0.133137818
PTEN	KLF12	0.291	0	-0.010342229
PTEN	C6orf72	0.291	0	-0.005041586
PTEN	RABEP1	0.291	0	-0.10789001
PTEN	ZBTB25	0.291	0	-0.000218686
PTEN	KIAA1737	0.291	0	-0.119949062
PTEN	PRDM10	0.291	0	-0.277394025
PTEN	CDON	0.291	0	-0.192210068
PTEN	CD46	0.291	0	0.165117498

PTEN	GNPDA2	0.292	0	-0.038953453
PTEN	PRSS23	0.292	0	-0.047144295
PTEN	FKBP14	0.292	0	-0.035523442
PTEN	ZNF322B	0.292	0	-0.179433176
PTEN	USP3	0.292	0	-0.111848061
PTEN	ASXL3	0.292	0	-0.188659771
PTEN	SMOC2	0.292	0	-0.105958671
PTEN	ZNF442	0.292	0	-0.005765458
PTEN	HSD17B4	0.292	0	-0.010616167
PTEN	ETNK1	0.292	0	-0.125552707
PTEN	SYNE1	0.292	0	0.009656544
PTEN	GAS7	0.292	0	-0.006833791
PTEN	SNX13	0.292	0	-0.228257743
PTEN	BLOC1S2	0.292	0	-0.168543455
PTEN	ZFP36L1	0.292	0	-0.005637566
PTEN	GRIA3	0.292	0	-0.34552298
PTEN	MTMR6	0.292	0	-0.384005811
PTEN	ALG10	0.292	0	0.081291579
PTEN	HABP2	0.292	0	-0.014779335
PTEN	ACTR10	0.293	0	-0.095306694
PTEN	PLAT	0.293	0	-0.027958826
PTEN	PPP1R12B	0.293	0	-1.83E-05
PTEN	BPTF	0.293	0	-0.138881711
PTEN	TSPAN1	0.293	0	0.005440205
PTEN	NRK	0.293	0	-0.243529016
PTEN	C5orf13	0.293	0	0.08453063
PTEN	SCARB2	0.293	0	0.038449122
PTEN	HMG20A	0.294	0	0.02144052
PTEN	RNASE4	0.294	0	-0.244470162
PTEN	ARSJ	0.294	0	-0.438680782
PTEN	VGLL3	0.294	0	-0.009112226
PTEN	KLF7	0.294	0	-0.134117124
PTEN	ZMYM1	0.294	0	-0.038490975
PTEN	ITGB1	0.294	0	0.151067388
PTEN	SH3RF1	0.294	0	0.089810588
PTEN	CALCRL	0.294	0	-0.057100314
PTEN	DUSP4	0.294	0	-0.023989321
PTEN	GLUD1	0.294	0	0.157032515
PTEN	FAM168A	0.294	0	-0.007789226
PTEN	FILIP1	0.294	0	-0.315071882
PTEN	SFRP2	0.294	0	0.134734674

PTEN	CNOT6	0.294	0	-0.00482431
PTEN	PIP	0.294	0	0.001176259
PTEN	LRCH1	0.294	0	-0.412561453
PTEN	ADRA2A	0.295	0	0.028796401
PTEN	ZNF625	0.295	0	-0.011292943
PTEN	CAPZA2	0.295	0	0.033713294
PTEN	C14orf132	0.295	0	-0.006764668
PTEN	MBNL2	0.295	0	0.038401565
PTEN	ZNF429	0.295	0	-4.92E-05
PTEN	MYCBP2	0.295	0	-0.28248336
PTEN	FOXJ3	0.295	0	-0.027433245
PTEN	ZNF440	0.295	0	-0.002666071
PTEN	CXCL12	0.295	0	-0.236805572
PTEN	EFCAB6	0.295	0	-7.58E-05
PTEN	MS4A14	0.295	0	-0.178123832
PTEN	LZIC	0.295	0	-0.006022447
PTEN	SCAPER	0.296	0	-0.267640521
PTEN	WDR19	0.296	0	-0.1199617
PTEN	SLC35A3	0.296	0	-0.051417245
PTEN	USP9X	0.296	0	-0.048709229
PTEN	C6orf201	0.296	0	-0.28674502
PTEN	KIAA1244	0.296	0	-0.169320359
PTEN	MAMDC2	0.296	0	-0.263802152
PTEN	CAPN8	0.296	0	1.12E-06
PTEN	SLC18A2	0.296	0	-0.175075232
PTEN	BCLAF1	0.296	0	0.070054243
PTEN	PCSK5	0.296	0	-0.002610308
PTEN	LRRC4C	0.296	0	-0.138202807
PTEN	GOLIM4	0.296	0	-0.167973294
PTEN	CDH6	0.296	0	-0.587547815
PTEN	ZNF627	0.296	0	-0.034990063
PTEN	NBAS	0.297	0	-0.160502215
PTEN	PGBD3	0.297	0	-0.0008922
PTEN	LOX	0.297	0	0.090528287
PTEN	MID2	0.297	0	0.094714469
PTEN	ZNF800	0.297	0	-0.150226573
PTEN	SMARCA5	0.297	0	0.051308537
PTEN	CCDC80	0.297	0	-0.147309483
PTEN	STON1-GTF2A1L	0.297	0	-0.003160826
PTEN	CHD1	0.297	0	-0.116137615
PTEN	MIOS	0.297	0	-0.130598308

PTEN	FKTN	0.297	0	-0.126679756
PTEN	CD2AP	0.298	0	0.072250654
PTEN	SYTL5	0.298	0	-0.205985557
PTEN	C1QTNF9	0.298	0	-0.217084309
PTEN	SIDT1	0.298	0	-0.090457697
PTEN	PTGER3	0.298	0	-0.068238193
PTEN	ZNF175	0.298	0	-0.196096721
PTEN	IL13RA1	0.298	0	0.444764123
PTEN	ZNF197	0.298	0	-0.066642506
PTEN	KIAA2026	0.298	0	-0.107208783
PTEN	NAT1	0.298	0	-0.0116272
PTEN	CORIN	0.298	0	-0.274565241
PTEN	PLK2	0.298	0	-0.022296396
PTEN	MYH4	0.298	0	-0.139376561
PTEN	INO80	0.298	0	-0.406842458
PTEN	TBC1D15	0.298	0	-0.035955783
PTEN	SHROOM3	0.299	0	-0.133492543
PTEN	TBCK	0.299	0	-0.294831186
PTEN	KDM3B	0.299	0	-0.160927756
PTEN	WASL	0.299	0	-0.081975983
PTEN	MLL	0.299	0	0.323755829
PTEN	LRBA	0.299	0	0.154480436
PTEN	ELK4	0.299	0	-0.189276913
PTEN	MPP5	0.299	0	-0.109842499
PTEN	MICAL2	0.299	0	0.126178664
PTEN	FILIP1L	0.299	0	-0.291154608
PTEN	PCDHB11	0.299	0	-0.000877193
PTEN	DCP1A	0.299	0	0.040093458
PTEN	RP2	0.299	0	-0.14624249
PTEN	LPAR4	0.299	0	-0.223657569
PTEN	SLC30A7	0.299	0	-0.08255525
PTEN	PURG	0.3	0	-0.942533955
PTEN	SESN1	0.3	0	-0.03340366
PTEN	ZNF253	0.3	0	-0.254457552
PTEN	ZNF780A	0.3	0	-0.188960093
PTEN	LRRK2	0.3	0	-0.10525723
PTEN	ANGPTL1	0.3	0	-0.405217118
PTEN	SMEK1	0.3	0	-0.065050935
PTEN	WNT5A	0.3	0	-0.213576047
PTEN	PRRX1	0.3	0	0.145860399
PTEN	DOCK4	0.3	0	0.007466788

PTEN	LAMC1	0.3	0	0.099608392
PTEN	PRDX3	0.3	0	-0.251653992
PTEN	SMARCAD1	0.3	0	-0.365106205
PTEN	PIGN	0.3	0	-0.066066203
PTEN	CRTC3	0.3	0	-0.018947855
PTEN	PTPRM	0.301	0	-0.22982953
PTEN	GPR116	0.301	0	-0.237409483
PTEN	DHFRL1	0.301	0	-0.285045137
PTEN	PTGFRN	0.301	0	0.165047943
PTEN	ITGB5	0.301	0	-0.026692037
PTEN	RGPD5	0.301	0	-0.065109835
PTEN	NUDT12	0.301	0	-0.235538088
PTEN	TNFRSF19	0.301	0	0.113395652
PTEN	LAMA4	0.301	0	-0.071695067
PTEN	STAG2	0.301	0	-0.221338388
PTEN	MMS19	0.301	0	-0.162819489
PTEN	LYPD6	0.301	0	-0.478608028
PTEN	SERINC5	0.301	0	1.66E-08
PTEN	C5orf51	0.301	0	-0.32297507
PTEN	HELZ	0.301	0	0.15544649
PTEN	DST	0.301	0	0.032415084
PTEN	MPDZ	0.301	0	-0.24505029
PTEN	ZSCAN29	0.301	0	0.058993841
PTEN	PRDM6	0.301	0	-0.124663863
PTEN	HSPA12A	0.301	0	-0.387146908
PTEN	TRMT5	0.301	0	-0.052642993
PTEN	STXBP5	0.301	0	-0.014204774
PTEN	ERCC4	0.302	0	0.064812758
PTEN	MBNL1	0.302	0	0.259069058
PTEN	FBXL5	0.302	0	0.026042369
PTEN	SPIN1	0.302	0	-0.380939541
PTEN	DGKI	0.302	0	-0.437157462
PTEN	PDE10A	0.302	0	-0.075772263
PTEN	ATM	0.302	0	-0.065623246
PTEN	ZC3H13	0.302	0	0.059259616
PTEN	ZXDA	0.302	0	-0.041435915
PTEN	WRN	0.302	0	-0.212972477
PTEN	KCTD3	0.302	0	0.161290905
PTEN	PDGFRA	0.302	0	-0.130828705
PTEN	ZNF134	0.303	0	-0.032674781
PTEN	GOLGA1	0.303	0	-0.019477514

PTEN	INPP4B	0.303	0	0.088306908
PTEN	ZKSCAN1	0.303	0	0.088363032
PTEN	HFE	0.303	0	-0.04888711
PTEN	RCOR1	0.303	0	0.079206326
PTEN	ARID1A	0.303	0	0.11505703
PTEN	KIAA0355	0.303	0	0.119675223
PTEN	IPMK	0.303	0	-0.115204826
PTEN	ARIH1	0.303	0	-0.105930912
PTEN	COL10A1	0.303	0	-0.056207743
PTEN	NEDD1	0.303	0	-0.040614081
PTEN	BTD	0.303	0	-0.006792429
PTEN	HELQ	0.303	0	0.005547909
PTEN	LRIG1	0.303	0	-0.227315712
PTEN	PXK	0.303	0	0.153278345
PTEN	RANBP6	0.303	0	-0.343334874
PTEN	KIF5C	0.304	0	-0.047402005
PTEN	GUCY1A2	0.304	0	-0.001490721
PTEN	TMC5	0.304	0	0.029409421
PTEN	THRAP3	0.304	0	0.063360527
PTEN	TTC8	0.304	0	0.143580241
PTEN	TBCEL	0.304	0	-0.17365512
PTEN	ANKHD1-EIF4EBP3	0.304	0	-0.003773148
PTEN	ZNF573	0.304	0	-5.53E-05
PTEN	FRMD4B	0.304	0	-0.139102611
PTEN	ZSCAN20	0.304	0	-0.164512815
PTEN	WDFY1	0.304	0	-0.22688933
PTEN	PAPPA	0.304	0	-0.128778991
PTEN	SCRN3	0.304	0	-0.054862291
PTEN	PTPRT	0.304	0	-6.37E-05
PTEN	PDE1A	0.304	0	-0.299928408
PTEN	LARP4	0.304	0	0.110446163
PTEN	ABAT	0.304	0	-0.076404882
PTEN	ZFP91-CNTF	0.305	0	-0.190821428
PTEN	F13A1	0.305	0	-0.010330375
PTEN	COG2	0.305	0	0.156375877
PTEN	CTDSPL	0.305	0	-0.160716773
PTEN	ARNT	0.305	0	0.045655671
PTEN	EID1	0.305	0	0.036418212
PTEN	NR3C1	0.305	0	-0.261102826
PTEN	PREX2	0.305	0	-0.016334719
PTEN	AMPH	0.305	0	-0.168546328

PTEN	ZNF45	0.305	0	-0.302783582
PTEN	OTUD7B	0.305	0	-0.018647823
PTEN	ENPP1	0.305	0	-0.057783786
PTEN	RTN1	0.305	0	-0.514746492
PTEN	ZNF491	0.305	0	-0.156412288
PTEN	AXL	0.305	0	-0.271360836
PTEN	PPP4R2	0.306	0	-0.058889364
PTEN	CLOCK	0.306	0	0.001559187
PTEN	DDR2	0.306	0	-0.140134941
PTEN	GALNTL2	0.306	0	-0.637059467
PTEN	YAP1	0.306	0	-0.235004901
PTEN	FAM66E	0.306	0	-0.173941548
PTEN	RAB8B	0.306	0	-0.173613152
PTEN	DPYD	0.306	0	-0.163390098
PTEN	ARFIP1	0.306	0	0.138145778
PTEN	DDI2	0.306	0	-0.001821837
PTEN	N4BP2	0.306	0	-0.030523566
PTEN	ZCCHC10	0.306	0	-0.151436411
PTEN	GAB1	0.306	0	0.042768955
PTEN	OLFML1	0.306	0	-0.113428791
PTEN	ANKRD30A	0.306	0	-0.010818347
PTEN	DCTN4	0.306	0	0.183847073
PTEN	PWWP2A	0.307	0	-0.350768653
PTEN	WDR89	0.307	0	-0.138931604
PTEN	ZFP112	0.307	0	-0.165583568
PTEN	NID2	0.307	0	-0.031196853
PTEN	KLF4	0.307	0	-0.099159759
PTEN	PTPRB	0.307	0	-0.175257361
PTEN	MR1	0.307	0	0.000309715
PTEN	CDC42BPB	0.307	0	0.013843551
PTEN	PTPRE	0.307	0	-0.017937633
PTEN	ODZ3	0.307	0	-0.257009948
PTEN	TCEAL7	0.307	0	-0.26563254
PTEN	SCUBE2	0.307	0	0.065005735
PTEN	ZNF615	0.307	0	-0.047159207
PTEN	RPS6KA5	0.307	0	-0.374859694
PTEN	CXADRP2	0.308	0	-0.064646505
PTEN	PTPRD	0.308	0	-0.455048559
PTEN	LRRFIP1	0.308	0	0.086044886
PTEN	DAZAP2	0.308	0	0.106436327
PTEN	RNASEL	0.308	0	-0.144518189

PTEN	JKAMP	0.308	0	-0.012849333
PTEN	SORL1	0.308	0	-0.108353243
PTEN	MAP4K3	0.308	0	-0.099712332
PTEN	ROBO2	0.308	0	-0.487785986
PTEN	CRTAP	0.308	0	-0.009803633
PTEN	ITGA2	0.308	0	0.015937753
PTEN	RALB	0.308	0	-0.124323792
PTEN	ZMAT3	0.308	0	-0.118767963
PTEN	FAT3	0.308	0	-0.287398618
PTEN	ZNF136	0.308	0	-0.178116203
PTEN	LYST	0.308	0	-0.135119949
PTEN	VEZT	0.309	0	-0.096569158
PTEN	NR4A2	0.309	0	0.13839339
PTEN	KLHL5	0.309	0	-0.095027798
PTEN	FAM22D	0.309	0	-0.241073368
PTEN	FNTB	0.309	0	-0.080735267
PTEN	TGFBR2	0.309	0	-0.069252481
PTEN	IBTK	0.309	0	-0.341841175
PTEN	ZC3H6	0.309	0	-0.025499014
PTEN	GULP1	0.309	0	-0.397717192
PTEN	SSPN	0.309	0	-0.116906153
PTEN	ANTXR1	0.309	0	0.292949114
PTEN	UACA	0.309	0	-0.193047248
PTEN	ARID5B	0.309	0	0.268314035
PTEN	MUC1	0.309	0	-0.005590143
PTEN	PPP6C	0.309	0	-0.153961369
PTEN	RAB3GAP1	0.309	0	-0.256315994
PTEN	LMLN	0.309	0	0.009265746
PTEN	SLC35A5	0.309	0	0.031758488
PTEN	ABHD2	0.309	0	-0.004811354
PTEN	COL14A1	0.31	0	-0.029665071
PTEN	LRCH3	0.31	0	-0.000506658
PTEN	ARMC9	0.31	0	-0.016582306
PTEN	LRIG2	0.31	0	-0.173626669
PTEN	CCDC126	0.31	0	-0.185323629
PTEN	CCNDBP1	0.31	0	-0.134628202
PTEN	PANK3	0.31	0	0.05432707
PTEN	ADAM12	0.31	0	0.058168188
PTEN	TTC30B	0.31	0	-0.107631716
PTEN	ENPP5	0.31	0	0.040278956
PTEN	PELI2	0.31	0	-0.184925618

PTEN	TMEM131	0.311	0	-0.010609831
PTEN	C16orf45	0.311	0	-0.00192875
PTEN	PAPOLA	0.311	0	0.200808381
PTEN	SWAP70	0.311	0	0.031554984
PTEN	RAPH1	0.311	0	0.151131311
PTEN	SPOCK1	0.311	0	-0.235378969
PTEN	GLIS3	0.311	0	-0.042389796
PTEN	MXRA5	0.311	0	0.14697507
PTEN	ZFP91	0.311	0	0.006418272
PTEN	DTWD1	0.311	0	-0.281336156
PTEN	ATG12	0.311	0	-0.053327421
PTEN	PUS7L	0.311	0	-0.166100842
PTEN	KIAA0831	0.311	0	-0.556283354
PTEN	ZNF12	0.311	0	-0.189570085
PTEN	C12orf26	0.311	0	-0.000128109
PTEN	PRKAR1A	0.312	0	0.166803333
PTEN	RNF11	0.312	0	0.129633793
PTEN	PRLR	0.312	0	1.61E-07
PTEN	GLT8D2	0.312	0	-0.089267864
PTEN	MYSM1	0.312	0	-2.23E-05
PTEN	COL4A5	0.312	0	-0.043472551
PTEN	PHF3	0.312	0	-0.179602105
PTEN	ZMYM4	0.312	0	-0.080315289
PTEN	TMEM26	0.312	0	-0.079520797
PTEN	COL6A3	0.312	0	0.272656955
PTEN	EPC1	0.312	0	-0.063564411
PTEN	PI4K2A	0.312	0	-0.022244978
PTEN	SYNE2	0.312	0	-0.120965268
PTEN	RBMS2	0.312	0	-0.000719497
PTEN	C10orf4	0.312	0	-0.414820643
PTEN	PDGFRL	0.312	0	0.000423518
PTEN	IGBP1	0.313	0	-0.032716192
PTEN	SMC3	0.313	0	-0.152097109
PTEN	REV3L	0.313	0	-0.187573624
PTEN	GNS	0.313	0	0.044670807
PTEN	CTDSP2	0.313	0	0.01850758
PTEN	SLC1A1	0.313	0	-0.137770267
PTEN	GOLPH3L	0.313	0	-0.042512076
PTEN	SON	0.313	0	0.016820696
PTEN	ABCA6	0.313	0	-0.057824219
PTEN	RCOR3	0.313	0	0.178539381

PTEN	C12orf23	0.313	0	0.077661921
PTEN	PTPN9	0.313	0	-0.264600874
PTEN	DCP2	0.314	0	-0.128529205
PTEN	VCL	0.314	0	0.138141007
PTEN	SNX2	0.314	0	0.149116394
PTEN	RYBP	0.314	0	0.020160563
PTEN	SKIL	0.314	0	-0.357967855
PTEN	ZFYVE26	0.314	0	0.067883529
PTEN	FAM38B	0.314	0	-0.111477768
PTEN	F2R	0.314	0	-0.007165154
PTEN	KIAA0430	0.314	0	-0.120623266
PTEN	USP34	0.314	0	0.089331076
PTEN	SLC30A4	0.314	0	-0.411461211
PTEN	GLI3	0.314	0	-0.200269879
PTEN	SFXN3	0.314	0	-0.099322988
PTEN	ZMPSTE24	0.314	0	-0.11182597
PTEN	SLFN12	0.314	0	-0.044637753
PTEN	TRPM7	0.315	0	-0.062033389
PTEN	HIPK1	0.315	0	-0.49355327
PTEN	ZBTB26	0.315	0	-0.438102146
PTEN	SPTY2D1	0.315	0	-0.130967178
PTEN	LOC729082	0.315	0	-0.181550501
PTEN	GTF2H3	0.315	0	-0.125607848
PTEN	TBX15	0.315	0	-0.219826234
PTEN	TPRG1	0.315	0	-0.042969538
PTEN	RIF1	0.315	0	-0.165678358
PTEN	EIF5	0.315	0	0.149920835
PTEN	NPTN	0.315	0	0.034515178
PTEN	SLC24A1	0.315	0	-0.348160415
PTEN	SLFN5	0.315	0	-0.206881342
PTEN	SERINC1	0.315	0	0.073245327
PTEN	FNIP2	0.316	0	0.023479631
PTEN	FLRT2	0.316	0	-0.661430412
PTEN	NFIA	0.316	0	0.16343945
PTEN	PIK3R4	0.316	0	-0.076189897
PTEN	CDH13	0.316	0	-0.123953574
PTEN	COL1A2	0.316	0	0.629742538
PTEN	ZNF284	0.316	0	-0.036229519
PTEN	UNC5C	0.316	0	0.007901317
PTEN	ZDHHC20	0.316	0	-0.161393318
PTEN	KIAA1377	0.317	0	-0.319594989

PTEN	UGCG	0.317	0	0.008088135
PTEN	ZNF407	0.317	0	-0.196377076
PTEN	HNRNPH2	0.317	0	-0.023376919
PTEN	LGALS8	0.317	0	-0.037251499
PTEN	TNIK	0.317	0	-0.315740047
PTEN	KIF16B	0.317	0	0.120376075
PTEN	ARL3	0.317	0	-0.204936227
PTEN	SLC38A2	0.317	0	0.373611165
PTEN	METT5D1	0.317	0	-0.064795334
PTEN	FRS2	0.317	0	-0.018792033
PTEN	PDZRN4	0.317	0	-0.186075806
PTEN	PTAR1	0.317	0	-0.004741857
PTEN	TMX1	0.318	0	-0.115409617
PTEN	ZNF221	0.318	0	-1.13E-05
PTEN	C14orf102	0.318	0	-0.011862839
PTEN	SLC44A4	0.318	0	0.009206583
PTEN	AKAP13	0.318	0	-0.097327025
PTEN	MAB21L1	0.318	0	-0.475162993
PTEN	ARL13B	0.318	0	0.060289328
PTEN	DNAJB4	0.318	0	-0.347497377
PTEN	SORBS1	0.318	0	-0.318203629
PTEN	C16orf72	0.318	0	-0.077317153
PTEN	DLG5	0.318	0	0.154592037
PTEN	CADPS2	0.319	0	-0.013772037
PTEN	C1orf168	0.319	0	-0.473708373
PTEN	CHD8	0.319	0	0.000834167
PTEN	PAPD4	0.319	0	-0.13091783
PTEN	KANK2	0.319	0	-0.022929549
PTEN	COL4A6	0.319	0	-0.244931335
PTEN	KLHL4	0.319	0	-0.45222787
PTEN	SLC25A21	0.319	0	-0.142984247
PTEN	MIB1	0.319	0	-0.056001897
PTEN	NAP1L3	0.319	0	-0.328572653
PTEN	KLHL11	0.319	0	0.006117041
PTEN	FBXW2	0.319	0	-0.022975676
PTEN	CNTN1	0.319	0	-0.017670765
PTEN	SRBD1	0.319	0	-0.071099767
PTEN	ANKFN1	0.319	0	-0.000193794
PTEN	C10orf137	0.319	0	-0.133902457
PTEN	RNF160	0.319	0	-0.165534563
PTEN	SLC7A2	0.32	0	0.254571343

PTEN	SF3B1	0.32	0	0.256349352
PTEN	SPARC	0.32	0	0.046834573
PTEN	TGOLN2	0.32	0	0.021905056
PTEN	CMYA5	0.32	0	-0.065438936
PTEN	ZNF660	0.32	0	-0.456269475
PTEN	MRVI1	0.32	0	-0.190733265
PTEN	RGPD3	0.32	0	-0.175134971
PTEN	SEC62	0.32	0	0.216108295
PTEN	TLL1	0.32	0	-0.321747131
PTEN	PPP1R12A	0.32	0	-0.125565952
PTEN	DNAL1	0.32	0	-0.024895671
PTEN	PPTC7	0.32	0	-0.032827675
PTEN	KIAA2018	0.321	0	-0.284161287
PTEN	KIAA0564	0.321	0	-0.296293818
PTEN	PIKFYVE	0.321	0	-0.010795643
PTEN	FMN1	0.321	0	-0.288346515
PTEN	C4orf32	0.321	0	-0.44077347
PTEN	ITPR1	0.321	0	-0.263155656
PTEN	UBXN7	0.321	0	-0.229082727
PTEN	NID1	0.321	0	-0.02235762
PTEN	IDS	0.321	0	-0.212051738
PTEN	HTRA1	0.321	0	-0.000120322
PTEN	DNAH5	0.321	0	-0.089663295
PTEN	TAF9B	0.322	0	-0.138473756
PTEN	ELL2	0.322	0	0.06220716
PTEN	MED13L	0.322	0	-0.156933913
PTEN	C9orf152	0.322	0	-0.177527321
PTEN	SEC24D	0.322	0	0.155496328
PTEN	DNAJC27	0.322	0	-0.367767895
PTEN	ZBTB41	0.322	0	-0.064416307
PTEN	TNS1	0.322	0	0.08329963
PTEN	GTF3C4	0.322	0	-0.072182669
PTEN	CAND1	0.322	0	0.075399264
PTEN	TC2N	0.322	0	0.195830628
PTEN	ZCCHC24	0.322	0	0.056390863
PTEN	ARSK	0.322	0	-0.421987411
PTEN	CAB39	0.322	0	0.076332319
PTEN	BCOR	0.322	0	-0.196446838
PTEN	RSPRY1	0.322	0	-4.06E-05
PTEN	PAK2	0.323	0	-0.045586627
PTEN	ZNF143	0.323	0	-0.189824005

PTEN	PRKAA2	0.323	0	-0.15584135
PTEN	SLC12A2	0.323	0	-0.343054618
PTEN	PDE3A	0.323	0	-0.011657662
PTEN	KIAA1598	0.323	0	-0.198549664
PTEN	USP12	0.323	0	-0.198972432
PTEN	ANKRD12	0.323	0	-0.185347463
PTEN	MCL1	0.323	0	0.14957467
PTEN	NSUN3	0.323	0	-0.166210233
PTEN	MTMR10	0.323	0	-0.176934573
PTEN	SLAIN2	0.323	0	-0.062038572
PTEN	RAB31	0.323	0	-0.072039232
PTEN	TWSG1	0.324	0	-0.251964933
PTEN	GXYLT2	0.324	0	-0.021393216
PTEN	MKLN1	0.324	0	-0.014465596
PTEN	MBTPS2	0.324	0	-0.099765952
PTEN	CROT	0.324	0	-0.033456849
PTEN	COL12A1	0.324	0	0.317468962
PTEN	ELMOD2	0.324	0	0.136536108
PTEN	COL3A1	0.324	0	0.226888032
PTEN	RGS17	0.324	0	-0.258706773
PTEN	SLC44A1	0.324	0	-0.142379747
PTEN	MACF1	0.324	0	0.183437313
PTEN	APBB2	0.325	0	-0.064257718
PTEN	GLCE	0.325	0	0.235883721
PTEN	FKBP7	0.325	0	-0.15222544
PTEN	WDR26	0.325	0	0.443461713
PTEN	RANBP2	0.325	0	-0.010064809
PTEN	CCNG1	0.325	0	0.143673841
PTEN	RAPGEF4	0.325	0	0.01355178
PTEN	XPR1	0.325	0	0.110248312
PTEN	EYS	0.325	0	-0.715534654
PTEN	SPON1	0.325	0	-0.036782619
PTEN	SAR1A	0.325	0	0.006174318
PTEN	SLIT3	0.325	0	-0.056051964
PTEN	BACH1	0.325	0	-0.497410287
PTEN	DNAJC16	0.325	0	-0.250759395
PTEN	ZNF564	0.325	0	-0.088179735
PTEN	PLEKHA1	0.326	0	-0.072965894
PTEN	FAM13B	0.326	0	-0.152065961
PTEN	HERC1	0.326	0	-0.041609527
PTEN	GIT2	0.326	0	-0.158321408

PTEN	ZNF616	0.326	0	-0.058741054
PTEN	ZDHHC21	0.326	0	-0.135340229
PTEN	MTX3	0.326	0	-0.162120115
PTEN	BIRC6	0.326	0	-0.318786108
PTEN	EYA3	0.326	0	-0.074089399
PTEN	CDKL5	0.326	0	-0.000215725
PTEN	SH3BGRL	0.327	0	0.221277652
PTEN	PSD3	0.327	0	-0.010107822
PTEN	INTS6	0.327	0	-0.115166026
PTEN	ACAP2	0.327	0	0.06764929
PTEN	LEPR	0.327	0	-0.001335559
PTEN	YTHDC2	0.327	0	-0.290691504
PTEN	NIN	0.327	0	-0.171012395
PTEN	SYT9	0.327	0	-0.153279884
PTEN	AKAP10	0.327	0	-0.227142447
PTEN	SNX29	0.327	0	-0.009658943
PTEN	PDE11A	0.328	0	-0.214164945
PTEN	TTC30A	0.328	0	-0.41899289
PTEN	RAB21	0.328	0	-0.142325959
PTEN	MS4A7	0.328	0	0.144604962
PTEN	PPIP5K2	0.328	0	0.095849466
PTEN	FBXO28	0.328	0	0.315005454
PTEN	RUNX2	0.328	0	-0.201488839
PTEN	PIAS1	0.328	0	0.087506835
PTEN	DNAJC13	0.328	0	0.118789383
PTEN	CAST	0.328	0	0.130087986
PTEN	EIF4G2	0.329	0	0.1565374
PTEN	RAB30	0.329	0	-0.029987685
PTEN	TOR1AIP2	0.329	0	0.081392433
PTEN	FRYL	0.329	0	-0.255218805
PTEN	ARHGAP5	0.329	0	-0.000805497
PTEN	CRIM1	0.329	0	-0.291093781
PTEN	PCDH19	0.329	0	-0.68049737
PTEN	HTR7	0.329	0	-0.448265895
PTEN	ZNF192	0.329	0	-0.217345765
PTEN	BBX	0.33	0	-0.258558839
PTEN	THBS2	0.33	0	0.195365814
PTEN	EXOC5	0.33	0	-0.372263617
PTEN	PIK3CA	0.33	0	-0.331363396
PTEN	CDH11	0.33	0	-0.002807314
PTEN	KIAA0232	0.33	0	-0.231878609

PTEN	ZNF91	0.33	0	-0.010452684
PTEN	NFE2L2	0.33	0	0.119496044
PTEN	FSTL1	0.33	0	0.048845404
PTEN	MUDENG	0.33	0	-0.058819078
PTEN	FAM120A	0.33	0	0.11548955
PTEN	RSBN1	0.33	0	-0.277478034
PTEN	FOXN3	0.331	0	-0.012059139
PTEN	MYST4	0.331	0	-0.206970106
PTEN	KIAA1632	0.331	0	0.143236126
PTEN	SPARCL1	0.331	0	0.142529049
PTEN	SSFA2	0.331	0	-0.08850985
PTEN	AGPS	0.331	0	0.09536787
PTEN	SLC4A7	0.331	0	-0.262088055
PTEN	ZNF214	0.331	0	-0.109560235
PTEN	MKL2	0.331	0	0.120150409
PTEN	ZNF436	0.331	0	-0.154247902
PTEN	DAAM1	0.331	0	-0.038642595
PTEN	ZMYM2	0.331	0	0.140491822
PTEN	ARPP19	0.331	0	-0.021503227
PTEN	KIAA1715	0.331	0	-0.08970073
PTEN	SCN7A	0.332	0	-0.218459714
PTEN	KIF13B	0.332	0	0.009234084
PTEN	FAP	0.332	0	0.236669924
PTEN	ZBTB20	0.332	0	-0.147206224
PTEN	TRAF6	0.332	0	-0.031277464
PTEN	ZNF365	0.332	0	-0.326542456
PTEN	TMEM47	0.332	0	-0.156613305
PTEN	PRKD1	0.332	0	-0.491099798
PTEN	PCM1	0.332	0	0.044647645
PTEN	VPS26A	0.332	0	-0.112426593
PTEN	ZNF430	0.332	0	-0.196598696
PTEN	ZNF41	0.333	0	-0.135012798
PTEN	2-Sep	0.333	0	0.420444273
PTEN	C5orf15	0.333	0	-0.116444738
PTEN	ZFYVE1	0.333	0	-0.020849382
PTEN	FAM161B	0.333	0	-0.140516917
PTEN	COL5A2	0.333	0	0.348611375
PTEN	SPEN	0.333	0	-0.017674355
PTEN	FGD6	0.333	0	-0.176289425
PTEN	SUFU	0.333	0	-0.200953508
PTEN	CTNND1	0.333	0	-0.037164143

PTEN	TAB3	0.333	0	-0.084983622
PTEN	LUZP1	0.333	0	-0.027904875
PTEN	LOC100129034	0.333	0	-1.76E-05
PTEN	DAB2	0.334	0	-0.219460113
PTEN	NUMB	0.334	0	-0.312267603
PTEN	DNAJC10	0.334	0	-0.131606845
PTEN	SPRED2	0.334	0	-0.030115443
PTEN	METTL14	0.334	0	-0.381697701
PTEN	CSTF2T	0.334	0	-0.226545954
PTEN	CNOT8	0.334	0	-0.045964294
PTEN	UBR3	0.335	0	-0.122873271
PTEN	ABLIM1	0.335	0	0.061908718
PTEN	DHX29	0.335	0	-0.147605286
PTEN	C4orf12	0.335	0	-0.032559856
PTEN	C1QTNF3	0.335	0	-0.062985424
PTEN	ZC3H14	0.335	0	-0.342188418
PTEN	PABPC5	0.335	0	-0.391034173
PTEN	SOCS6	0.335	0	-0.175573348
PTEN	NAV3	0.335	0	-0.314935064
PTEN	AGTR1	0.335	0	-0.172107434
PTEN	PDS5B	0.335	0	0.151587032
PTEN	BOD1L	0.336	0	-0.00856234
PTEN	FERMT2	0.336	0	-0.053830947
PTEN	SBF2	0.336	0	-0.168443163
PTEN	7-Mar	0.336	0	0.106272507
PTEN	MGA	0.336	0	-0.187739345
PTEN	PDGFD	0.336	0	-0.091289261
PTEN	KIAA1279	0.336	0	0.143473249
PTEN	FMO5	0.336	0	-0.341576463
PTEN	LRRC31	0.336	0	-0.26913663
PTEN	STOM	0.336	0	0.242706121
PTEN	TEX9	0.336	0	-0.100632089
PTEN	ELF2	0.337	0	-0.144281114
PTEN	MOSPD2	0.337	0	-0.454263925
PTEN	CASD1	0.337	0	-0.172289644
PTEN	HEATR5A	0.337	0	-0.067034912
PTEN	SIN3A	0.337	0	-0.071171516
PTEN	GJA1	0.337	0	0.43310738
PTEN	LASS6	0.338	0	0.222918721
PTEN	COPS2	0.338	0	0.031670457
PTEN	MED21	0.338	0	-0.12669994

PTEN	C1orf21	0.338	0	-4.47E-05
PTEN	MAML3	0.338	0	-0.426911193
PTEN	GPAM	0.338	0	-0.301701471
PTEN	AGGF1	0.338	0	0.102300233
PTEN	CREBL2	0.338	0	0.045583738
PTEN	ASXL2	0.339	0	0.037268124
PTEN	ST6GAL2	0.339	0	-0.211410425
PTEN	C14orf118	0.339	0	-0.537427091
PTEN	GPR34	0.339	0	-0.346005858
PTEN	TMOD3	0.339	0	-0.063175827
PTEN	MED13	0.34	0	-0.114323918
PTEN	TMTC2	0.34	0	-0.323041
PTEN	CENPC1	0.34	0	-0.019747178
PTEN	C14orf37	0.34	0	-0.263956228
PTEN	TMF1	0.34	0	-0.255507125
PTEN	STARD13	0.34	0	-0.661157556
PTEN	SOCS4	0.34	0	-0.050783851
PTEN	FLRT3	0.34	0	-0.540678244
PTEN	C3orf63	0.34	0	0.028569246
PTEN	REL	0.34	0	-0.026094423
PTEN	C1orf58	0.34	0	-0.129438207
PTEN	TMEM167A	0.34	0	-0.094245989
PTEN	MSH3	0.34	0	-0.107220495
PTEN	AP3M1	0.34	0	-0.095890169
PTEN	APOOL	0.341	0	-0.180199506
PTEN	RGMB	0.341	0	-0.019215863
PTEN	APPBP2	0.341	0	-0.218178461
PTEN	KRAS	0.341	0	0.182034868
PTEN	NOTCH2	0.342	0	0.253171769
PTEN	GPR98	0.342	0	-0.373895418
PTEN	HERC4	0.342	0	0.072981794
PTEN	FAT4	0.342	0	-0.372685691
PTEN	USP33	0.342	0	-0.030877876
PTEN	ZNF770	0.342	0	0.109945933
PTEN	APPL2	0.342	0	-0.143126159
PTEN	FAM26E	0.342	0	-0.199637359
PTEN	GIN1	0.342	0	-0.288880052
PTEN	LIN52	0.342	0	0.034172768
PTEN	PUM1	0.342	0	0.213634305
PTEN	CCDC6	0.342	0	0.30776669
PTEN	MAP4K5	0.343	0	-0.090553926

PTEN	ASPN	0.343	0	-0.10108056
PTEN	ANKRD30B	0.343	0	-0.144401626
PTEN	ZNF844	0.343	0	-0.009284386
PTEN	ZNF654	0.343	0	-0.202549745
PTEN	SGPL1	0.344	0	-0.00099999
PTEN	MSRB3	0.344	0	-0.10673545
PTEN	SVEP1	0.344	0	-0.23207847
PTEN	HEG1	0.344	0	-0.299649618
PTEN	ETV1	0.344	0	-0.17703221
PTEN	HBP1	0.344	0	-0.149067149
PTEN	IQGAP2	0.344	0	-0.019273075
PTEN	SNAP23	0.344	0	0.146847698
PTEN	LNX2	0.344	0	-0.145358318
PTEN	RAB14	0.344	0	0.220800971
PTEN	FAM114A1	0.345	0	-0.024783895
PTEN	SLC35F5	0.345	0	-0.101216466
PTEN	RASGRF2	0.345	0	-0.164284655
PTEN	RORA	0.345	0	-0.183108743
PTEN	WISP1	0.345	0	-0.077108992
PTEN	ZFX	0.345	0	-0.039234079
PTEN	SPATA7	0.345	0	-0.000923666
PTEN	FBXO38	0.345	0	-0.033904969
PTEN	C7orf58	0.345	0	-0.302382598
PTEN	GALC	0.345	0	-0.108665364
PTEN	GMFB	0.345	0	-0.081654318
PTEN	ATP10D	0.345	0	-0.346565401
PTEN	FBXL17	0.345	0	-0.093799013
PTEN	SLC39A10	0.345	0	-0.038944207
PTEN	TMEM184C	0.346	0	-0.195345991
PTEN	ZNF281	0.346	0	-0.031236027
PTEN	HGF	0.346	0	-0.225603069
PTEN	FAM175A	0.346	0	-0.04162856
PTEN	PCDH7	0.346	0	-0.026127825
PTEN	CLASP2	0.346	0	0.012606239
PTEN	EIF4B	0.346	0	0.144545041
PTEN	ZNF148	0.346	0	-0.118262691
PTEN	S100PBP	0.346	0	0.004044694
PTEN	CPEB2	0.346	0	-0.077467809
PTEN	KLHL9	0.347	0	-0.025190055
PTEN	VAMP3	0.347	0	0.164963778
PTEN	CSNK1A1	0.347	0	0.071562115

PTEN	PBX1	0.347	0	-0.067809844
PTEN	NOC3L	0.347	0	-0.007308486
PTEN	BICC1	0.347	0	-0.005990177
PTEN	NAA30	0.347	0	-0.179049367
PTEN	FER	0.347	0	-0.000636851
PTEN	KTN1	0.347	0	-0.079266763
PTEN	GNAQ	0.347	0	0.072760189
PTEN	USP47	0.348	0	0.171557307
PTEN	SGMS1	0.348	0	-0.111759378
PTEN	PALLD	0.348	0	-0.120147263
PTEN	RALGAPA2	0.348	0	-0.205610703
PTEN	INPP5F	0.348	0	-0.253540439
PTEN	NEGR1	0.348	0	-0.53778619
PTEN	SLC38A4	0.348	0	-0.09153165
PTEN	SEC24A	0.349	0	0.107788469
PTEN	STAG1	0.349	0	-0.099759892
PTEN	LAMA2	0.349	0	-0.01179166
PTEN	PRMT10	0.349	0	-0.000198441
PTEN	AP3B1	0.349	0	-0.006772288
PTEN	ARHGAP29	0.349	0	0.077565355
PTEN	IQGAP1	0.349	0	0.032671987
PTEN	PKD2	0.349	0	-0.39778146
PTEN	DNAH7	0.349	0	-0.302243086
PTEN	COBLL1	0.349	0	-0.111120421
PTEN	PBRM1	0.35	0	-0.229276386
PTEN	EIF4E3	0.35	0	-0.511755654
PTEN	MLL3	0.35	0	-0.080654461
PTEN	ANKRD50	0.35	0	-0.041634047
PTEN	ARHGAP6	0.35	0	-0.72295393
PTEN	KIAA1430	0.35	0	-0.048158637
PTEN	SNRK	0.35	0	-0.282182063
PTEN	NRP1	0.35	0	-0.022790015
PTEN	OSBPL8	0.351	0	-0.270999509
PTEN	RALGAPA1	0.351	0	-0.155878248
PTEN	C4orf31	0.351	0	-0.106629924
PTEN	ZNF585B	0.351	0	-0.086922957
PTEN	UBE2Q2	0.351	0	-0.14615742
PTEN	SPRED1	0.351	0	-0.135565235
PTEN	TMEM87B	0.351	0	0.213964737
PTEN	NAALADL2	0.351	0	-0.091027357
PTEN	CEP350	0.351	0	0.032919935

PTEN	GALNT4	0.351	0	-0.341309631
PTEN	MIER3	0.351	0	-0.081934423
PTEN	SFRS12	0.352	0	-0.110643736
PTEN	ZBTB11	0.352	0	0.115841883
PTEN	GIGYF2	0.352	0	0.113885597
PTEN	DCLRE1A	0.352	0	-0.303522749
PTEN	KDM6A	0.352	0	-0.263041853
PTEN	CSGALNACT2	0.352	0	-0.375767147
PTEN	PHAX	0.352	0	-0.171110503
PTEN	DDX3X	0.353	0	0.398067108
PTEN	TANC2	0.353	0	0.014784256
PTEN	MTUS1	0.353	0	-0.052623938
PTEN	DCAF5	0.353	0	0.102841581
PTEN	GCNT4	0.353	0	-0.197995995
PTEN	GSTCD	0.353	0	-0.163646009
PTEN	AKAP12	0.353	0	-0.263160928
PTEN	RECK	0.353	0	-0.177571854
PTEN	MMP16	0.353	0	-0.374477528
PTEN	VPS13D	0.353	0	-0.038006024
PTEN	SLC39A9	0.354	0	-0.056107476
PTEN	CDK17	0.354	0	0.058620133
PTEN	TNKS	0.354	0	0.156478694
PTEN	PPM1A	0.354	0	-0.109168307
PTEN	UGDH	0.354	0	-0.024884527
PTEN	CDK15	0.354	0	-0.011979752
PTEN	MORC3	0.354	0	-0.310749312
PTEN	C9orf5	0.354	0	-0.11879009
PTEN	SOS2	0.354	0	-0.044966299
PTEN	FAM172A	0.354	0	-0.298249258
PTEN	TAOK1	0.354	0	0.043297469
PTEN	RICTOR	0.355	0	-0.295290853
PTEN	SLC30A5	0.355	0	-0.005021548
PTEN	INO80D	0.355	0	-0.000472752
PTEN	OGN	0.355	0	-0.257333724
PTEN	TSHZ3	0.355	0	-0.600710444
PTEN	MIA3	0.355	0	0.050889842
PTEN	NEK10	0.355	0	-0.032741004
PTEN	LRCH2	0.355	0	-0.364761126
PTEN	NCOA1	0.356	0	0.032678002
PTEN	C10orf72	0.356	0	-0.056608713
PTEN	UBE3A	0.356	0	-0.066708526

PTEN	C9orf102	0.356	0	-0.175083907
PTEN	FUT8	0.356	0	-0.006617863
PTEN	ELOVL5	0.356	0	0.332702355
PTEN	SEMA3D	0.356	0	-0.371970419
PTEN	ST8SIA6	0.356	0	-0.000133617
PTEN	CHSY3	0.356	0	-0.54418987
PTEN	JAK1	0.356	0	-0.335378208
PTEN	RAB3GAP2	0.356	0	-0.208719657
PTEN	KLF9	0.357	0	0.032914048
PTEN	C14orf45	0.357	0	-0.068582073
PTEN	CEP97	0.357	0	-0.064125651
PTEN	ZAK	0.357	0	-0.169939101
PTEN	TRIM8	0.357	0	0.008648198
PTEN	EIF4G3	0.357	0	-0.062080441
PTEN	FMOD	0.357	0	0.070630551
PTEN	GALNT7	0.357	0	0.219889019
PTEN	RAD50	0.357	0	-0.104228277
PTEN	SLC24A2	0.357	0	-0.141929424
PTEN	ZFPM2	0.358	0	-0.466161531
PTEN	OBFC1	0.358	0	-0.140359
PTEN	STRN3	0.358	0	-0.10702248
PTEN	ZYG11B	0.358	0	0.13118076
PTEN	RIN2	0.358	0	-0.04966505
PTEN	ZNF254	0.358	0	-0.28502682
PTEN	ZNF791	0.358	0	-0.031157339
PTEN	SEMA6D	0.358	0	-0.658991117
PTEN	NBEA	0.358	0	-0.360550534
PTEN	DIP2B	0.358	0	0.160015014
PTEN	NF1	0.358	0	-0.039618275
PTEN	CDYL2	0.359	0	-0.007959694
PTEN	SEMA5A	0.359	0	-0.000210611
PTEN	ZBTB8A	0.359	0	-0.173637324
PTEN	VCAN	0.359	0	-0.075996801
PTEN	TTC28	0.359	0	-0.024284033
PTEN	KERA	0.359	0	-0.4166599
PTEN	OSMR	0.359	0	-0.006760017
PTEN	KIAA1109	0.359	0	-0.003657848
PTEN	TTC37	0.359	0	-0.065078218
PTEN	DCN	0.36	0	0.207478244
PTEN	RABL3	0.36	0	-0.117059412
PTEN	DENND4A	0.36	0	-0.642552966

PTEN	XIAP	0.36	0	-0.07862417
PTEN	IREB2	0.36	0	-0.038780199
PTEN	FRY	0.36	0	-0.126817928
PTEN	MCC	0.36	0	-0.191610402
PTEN	MLL5	0.36	0	-0.10501417
PTEN	SKIV2L2	0.36	0	-0.061280344
PTEN	SLIT2	0.36	0	-0.163650215
PTEN	TNNI3K	0.36	0	-0.015431063
PTEN	DPY19L3	0.36	0	-0.020966172
PTEN	LUM	0.36	0	0.124044994
PTEN	PPFIBP1	0.36	0	-0.094208126
PTEN	ZNF81	0.361	0	-0.190012726
PTEN	NEK1	0.361	0	0.085538612
PTEN	EIF4EBP2	0.361	0	-0.054540559
PTEN	RAP1B	0.361	0	0.25124785
PTEN	PLCL1	0.361	0	-0.34755992
PTEN	ZMYM6	0.361	0	-0.010328908
PTEN	MAN1A1	0.361	0	-0.256860715
PTEN	CEP120	0.361	0	-0.137876086
PTEN	NEO1	0.362	0	-0.037974916
PTEN	PGGT1B	0.362	0	-0.159588869
PTEN	BMPR1A	0.362	0	-0.277833747
PTEN	DYNC1I2	0.362	0	-0.068974702
PTEN	RNLS	0.362	0	-0.445972208
PTEN	EDIL3	0.362	0	-0.046329504
PTEN	ARHGEF12	0.362	0	0.092535597
PTEN	C5orf36	0.362	0	-0.6773231
PTEN	DNMBP	0.362	0	-0.052150661
PTEN	LRRC17	0.363	0	0.028227956
PTEN	CTDSPL2	0.363	0	-0.022162248
PTEN	FAM175B	0.363	0	-0.220926396
PTEN	MBD5	0.364	0	-0.335967019
PTEN	MYO5C	0.364	0	-0.069623857
PTEN	MTMR9	0.364	0	-0.109599441
PTEN	WDR7	0.364	0	-0.1123857
PTEN	C4orf34	0.364	0	0.092189747
PTEN	CCNI	0.364	0	0.001415288
PTEN	ZNF25	0.364	0	-0.284086808
PTEN	PHLDB2	0.364	0	-0.187535429
PTEN	KCND2	0.364	0	-0.132169442
PTEN	NCOA4	0.364	0	0.021081287

PTEN	ZNF225	0.364	0	-0.118337844
PTEN	ENTPD5	0.365	0	-0.191044319
PTEN	ANTXR2	0.365	0	-0.01711817
PTEN	WDR36	0.365	0	-0.144609276
PTEN	SGK269	0.365	0	-0.040812311
PTEN	ATF6	0.365	0	-0.001565939
PTEN	YPEL2	0.365	0	0.085736927
PTEN	GPD2	0.366	0	0.006028273
PTEN	C10orf84	0.366	0	-0.156816596
PTEN	TIPARP	0.366	0	-0.280834134
PTEN	TMED10	0.366	0	0.039257984
PTEN	SMAD1	0.366	0	-0.026691263
PTEN	DSEL	0.366	0	0.078394083
PTEN	MAN1A2	0.366	0	-0.169096205
PTEN	SETBP1	0.366	0	-0.038044278
PTEN	KIDINS220	0.366	0	-0.234494932
PTEN	BNIP3L	0.366	0	0.113395488
PTEN	C5orf42	0.367	0	-0.456232675
PTEN	ZBTB6	0.367	0	-0.000854974
PTEN	FAM122A	0.367	0	-0.175149798
PTEN	CHIC1	0.367	0	-0.295564024
PTEN	KCNMA1	0.367	0	-0.060507677
PTEN	TMEM106B	0.367	0	-0.128524301
PTEN	FBXL3	0.367	0	-0.010081759
PTEN	PAWR	0.367	0	0.096357808
PTEN	ZNF699	0.367	0	-0.003650129
PTEN	ITGA1	0.367	0	-0.402096229
PTEN	ANKHD1	0.368	0	-0.000272808
PTEN	DMXL1	0.368	0	-0.032568242
PTEN	SLC40A1	0.368	0	0.076085498
PTEN	NRIP1	0.368	0	0.182151299
PTEN	TRIP12	0.368	0	0.08911005
PTEN	ATP8B1	0.368	0	0.09043579
PTEN	CDK14	0.368	0	-0.253077294
PTEN	ATP7A	0.369	0	-0.266474034
PTEN	FAM179B	0.369	0	-0.060597089
PTEN	SCAMP1	0.369	0	0.075723246
PTEN	SNTB2	0.369	0	0.017973995
PTEN	TRIM23	0.369	0	0.022069906
PTEN	FBN1	0.369	0	0.033425427
PTEN	GPX8	0.37	0	-0.285321408

PTEN	ZRANB1	0.37	0	0.004431105
PTEN	SPOPL	0.37	0	-0.131881625
PTEN	SRFBP1	0.37	0	-0.191605818
PTEN	KIAA1462	0.37	0	-0.318503644
PTEN	RALGPS2	0.371	0	-0.181845032
PTEN	TP53BP1	0.371	0	-0.228929833
PTEN	UBL3	0.371	0	-0.130775826
PTEN	RUNX1T1	0.372	0	-0.3828502
PTEN	ELK3	0.372	0	-0.442800168
PTEN	FPGT	0.372	0	0.004075407
PTEN	KLHL20	0.372	0	-0.113756251
PTEN	EPC2	0.372	0	-0.424350325
PTEN	NUAK1	0.372	0	-0.320796519
PTEN	ARHGEF38	0.372	0	-0.368045908
PTEN	PCNX	0.372	0	-0.106501904
PTEN	CTSO	0.372	0	-0.168005465
PTEN	SYNJ2BP	0.373	0	-0.111956816
PTEN	FAM45B	0.373	0	-0.324076079
PTEN	REST	0.373	0	-3.84E-07
PTEN	PSEN1	0.373	0	-0.139235031
PTEN	MIER1	0.373	0	0.063540347
PTEN	PIK3C2A	0.373	0	0.102462615
PTEN	ARHGAP42	0.374	0	-0.399213667
PTEN	PLXNC1	0.374	0	-0.091706643
PTEN	DOK6	0.374	0	-0.019620515
PTEN	ZNF426	0.374	0	-0.148773434
PTEN	ZFYVE16	0.375	0	-0.148401296
PTEN	NTN4	0.375	0	-0.358862543
PTEN	LSM11	0.376	0	-0.008196696
PTEN	NARG2	0.376	0	-0.301095618
PTEN	PRDM2	0.376	0	-0.140231571
PTEN	GALNT5	0.377	0	-0.001402269
PTEN	ROCK1	0.377	0	-0.136883961
PTEN	PRDM5	0.377	0	-0.009893894
PTEN	TCP11L2	0.377	0	-0.034075689
PTEN	RB1	0.377	0	0.275079052
PTEN	BNC2	0.377	0	-4.87E-05
PTEN	UTRN	0.377	0	-0.062177286
PTEN	RAPGEF6	0.378	0	-0.165657849
PTEN	WDFY3	0.378	0	0.061941115
PTEN	USP53	0.378	0	-0.028912316

PTEN	VTI1A	0.378	0	-0.317312389
PTEN	CHD9	0.378	0	-0.252873346
PTEN	PPP3CB	0.378	0	0.007380049
PTEN	RASSF8	0.379	0	-0.077137226
PTEN	KIAA1033	0.38	0	-0.087403481
PTEN	HMBOX1	0.38	0	-0.207462488
PTEN	HIPK3	0.38	0	-0.281315048
PTEN	NEK9	0.38	0	-0.204962467
PTEN	CACNA2D1	0.381	0	-0.001395932
PTEN	HDX	0.381	0	-0.20893561
PTEN	ZNF619	0.381	0	-0.01902756
PTEN	USP8	0.381	0	-0.138338884
PTEN	LATS1	0.381	0	-0.410470422
PTEN	EIF3A	0.381	0	0.103730602
PTEN	POSTN	0.382	0	0.315477994
PTEN	CCNG2	0.382	0	0.001699914
PTEN	KIAA0586	0.382	0	-0.293610558
PTEN	TCF4	0.382	0	-0.227444974
PTEN	CSNK1G1	0.382	0	0.008380864
PTEN	MLH3	0.383	0	-0.241651427
PTEN	CGGBP1	0.383	0	-0.34180752
PTEN	TMED7	0.383	0	-0.016293745
PTEN	TET2	0.383	0	-0.644642101
PTEN	EVI5	0.384	0	-0.102445111
PTEN	PDGFC	0.384	0	-0.209711628
PTEN	ATXN1	0.384	0	-0.041865713
PTEN	SGCD	0.385	0	-0.038606219
PTEN	AQR	0.385	0	0.0400946
PTEN	ALDH6A1	0.385	0	-0.098008274
PTEN	SPG11	0.385	0	-0.152996677
PTEN	ELF1	0.385	0	0.207920141
PTEN	C5orf24	0.385	0	-0.157548152
PTEN	ZFHX4	0.385	0	-0.593790672
PTEN	HECTD1	0.386	0	-0.197813147
PTEN	NIPBL	0.386	0	-0.41301128
PTEN	TRIO	0.386	0	-0.128212034
PTEN	TGFB3	0.386	0	0.021086628
PTEN	ATF2	0.386	0	-0.10002363
PTEN	ECM2	0.386	0	-0.177176506
PTEN	C10orf88	0.386	0	-0.059816075
PTEN	MAP3K1	0.386	0	-0.098547027

PTEN	CCNT1	0.386	0	-0.000576921
PTEN	ZFP106	0.386	0	-0.090540997
PTEN	KIAA0247	0.386	0	-0.021859409
PTEN	TRAK2	0.386	0	-0.244721937
PTEN	AKAP11	0.387	0	-0.214189835
PTEN	IPO11	0.387	0	-0.001300808
PTEN	SEC23A	0.387	0	-0.051908533
PTEN	KIF13A	0.387	0	-0.480324206
PTEN	VPS13C	0.387	0	-0.149866014
PTEN	PRRC1	0.388	0	-0.069772462
PTEN	GOLGB1	0.388	0	-0.089743584
PTEN	OMD	0.388	0	-0.081514496
PTEN	CHM	0.388	0	-0.297642523
PTEN	FAM73A	0.389	0	0.064598827
PTEN	ZNF510	0.389	0	-0.116301132
PTEN	UHMK1	0.389	0	-0.393793076
PTEN	ACVR1	0.389	0	0.099315768
PTEN	STXBP4	0.39	0	-0.206607323
PTEN	FAM114A2	0.39	0	-0.014504007
PTEN	DNAJB14	0.39	0	-0.128454161
PTEN	EDNRA	0.39	0	0.045355933
PTEN	HNMT	0.39	0	-0.193713969
PTEN	SEMA3C	0.39	0	0.035754693
PTEN	CSNK1G3	0.39	0	-0.350696132
PTEN	MON2	0.39	0	-0.104118678
PTEN	RUFY2	0.391	0	-0.065612922
PTEN	GCC2	0.391	0	-0.281756936
PTEN	SEC24B	0.391	0	-0.206460035
PTEN	MAP3K2	0.391	0	-0.111850818
PTEN	FNIP1	0.392	0	-0.131603907
PTEN	EDEM3	0.392	0	0.080055103
PTEN	LMBRD2	0.392	0	-0.303466637
PTEN	LIPA	0.392	0	-0.031602108
PTEN	FOXP1	0.392	0	-0.048637331
PTEN	YIPF5	0.393	0	0.097238667
PTEN	PIGK	0.393	0	-0.140392285
PTEN	FNDC3A	0.393	0	0.136067998
PTEN	MGEA5	0.394	0	0.254217503
PTEN	ZNF561	0.394	0	-0.04077174
PTEN	KLF3	0.394	0	-0.056210258
PTEN	RNF111	0.394	0	-0.182868078

PTEN	BTRC	0.394	0	-0.030929748
PTEN	SLC30A1	0.394	0	-0.180273127
PTEN	AHNAK	0.395	0	0.230095881
PTEN	ARHGAP20	0.395	0	-0.359356455
PTEN	ASH1L	0.395	0	-0.371836421
PTEN	EPB41L4A	0.395	0	-0.097000718
PTEN	AKAP9	0.395	0	-0.070381288
PTEN	CHST15	0.396	0	-0.05293223
PTEN	VWA2	0.396	0	-0.098837754
PTEN	SIRT1	0.396	0	-0.221593094
PTEN	INADL	0.396	0	0.02084083
PTEN	TLR3	0.397	0	-0.140616878
PTEN	FRMD6	0.398	0	0.104267255
PTEN	EVC	0.398	0	-0.051634887
PTEN	KIAA1432	0.398	0	-0.152414704
PTEN	MITF	0.398	0	-0.436759506
PTEN	PTPRG	0.398	0	-0.307655762
PTEN	SLC25A46	0.398	0	-0.016733117
PTEN	TBC1D19	0.398	0	-0.253126824
PTEN	UBTD2	0.399	0	-0.3818801
PTEN	LYSMD3	0.4	0	-0.021337461
PTEN	HMCN1	0.4	0	-0.039607019
PTEN	MEGF9	0.401	0	-0.016892947
PTEN	FAM190B	0.401	0	-0.154472414
PTEN	AFF1	0.401	0	0.111475464
PTEN	LIMA1	0.402	0	0.085587168
PTEN	NBEAL1	0.402	0	-0.096909912
PTEN	TJP1	0.402	0	-0.139239215
PTEN	SMAD5	0.402	0	0.002812051
PTEN	ACER2	0.403	0	-0.151315057
PTEN	SP3	0.404	0	-0.287647063
PTEN	C14orf129	0.404	0	-0.218373895
PTEN	COX15	0.404	0	-0.164476555
PTEN	TOR1AIP1	0.404	0	-0.15354302
PTEN	DPP8	0.404	0	-0.478420447
PTEN	UEVLD	0.406	0	0.009289657
PTEN	TIAL1	0.406	0	-0.004456085
PTEN	DYNC2H1	0.406	0	-0.204750022
PTEN	ZBTB1	0.406	0	-0.040406625
PTEN	DHX32	0.406	0	-0.060828863
PTEN	PRICKLE2	0.406	0	-0.14820236

PTEN	DIXDC1	0.406	0	0.124022369
PTEN	PCGF5	0.407	0	-0.194876815
PTEN	TTBK2	0.407	0	-0.033638117
PTEN	RUNX1	0.408	0	0.173944686
PTEN	C10orf76	0.408	0	0.125945401
PTEN	GRINL1A	0.408	0	-0.207531702
PTEN	FEM1B	0.408	0	-0.000336797
PTEN	TMTC3	0.408	0	-0.02295203
PTEN	PHC3	0.409	0	0.000101358
PTEN	BDP1	0.409	0	0.013950641
PTEN	PARVA	0.41	0	-0.031832976
PTEN	PRKG1	0.41	0	-0.206871763
PTEN	MAST4	0.41	0	0.000687653
PTEN	UBR1	0.411	0	-0.111402277
PTEN	TNPO1	0.412	0	0.059020174
PTEN	RBM27	0.412	0	-0.293651046
PTEN	MYO9A	0.412	0	-0.162639501
PTEN	SPATA6	0.412	0	-0.009663329
PTEN	PLA2R1	0.413	0	-0.356648671
PTEN	COL4A3BP	0.413	0	-0.067854144
PTEN	ZBTB38	0.413	0	-0.085551545
PTEN	RAB33B	0.414	0	-0.351693918
PTEN	ATF1	0.414	0	-0.221516941
PTEN	MICALCL	0.415	0	-0.521351048
PTEN	EEA1	0.415	0	-0.122612882
PTEN	LNPEP	0.415	0	-0.296544882
PTEN	RBMS3	0.415	0	-0.358446149
PTEN	TEAD1	0.416	0	-0.229793119
PTEN	ADAM10	0.417	0	-0.08464517
PTEN	C10orf32	0.417	0	-0.153434341
PTEN	ZNF678	0.417	0	-0.349444102
PTEN	MEIS3P1	0.417	0	-0.015149308
PTEN	FUT11	0.418	0	-0.039924987
PTEN	VEZF1	0.418	0	0.045496602
PTEN	ERLIN1	0.419	0	-0.017226713
PTEN	MATR3	0.419	0	0.23832468
PTEN	SETX	0.42	0	-0.035039237
PTEN	CCPG1	0.42	0	0.169800197
PTEN	KIAA1370	0.421	0	0.134823448
PTEN	C14orf135	0.421	0	-0.097638403
PTEN	FYCO1	0.421	0	-0.041386344

PTEN	FAM35B	0.421	0	-0.189187334
PTEN	DENND4C	0.422	0	0.077193066
PTEN	BAZ2B	0.422	0	-0.187667621
PTEN	BCL2L11	0.423	0	0.082480838
PTEN	ARL15	0.423	0	0.010392827
PTEN	CASC4	0.423	0	0.122880067
PTEN	JMJD1C	0.423	0	-0.074040997
PTEN	WDR44	0.423	0	0.009527114
PTEN	GALNT10	0.424	0	0.180016621
PTEN	FCHO2	0.424	0	-0.232412722
PTEN	SAMD8	0.425	0	-0.135866483
PTEN	DICER1	0.425	0	-0.010527962
PTEN	PLSCR4	0.425	0	-0.291755509
PTEN	HCFC2	0.425	0	-0.02947293
PTEN	POLK	0.426	0	-0.226707529
PTEN	ARID2	0.426	0	-0.260509495
PTEN	LPAR1	0.427	0	-0.260802363
PTEN	C10orf12	0.427	0	-0.282760273
PTEN	CYBRD1	0.427	0	0.256691455
PTEN	RASA1	0.428	0	0.161555151
PTEN	PPP2R5E	0.428	0	-0.123302242
PTEN	ZNF280D	0.428	0	-0.199924328
PTEN	PLDN	0.429	0	-0.015350691
PTEN	AR	0.429	0	0.163390128
PTEN	RAB27B	0.429	0	-0.183600491
PTEN	C5orf41	0.429	0	-0.097239382
PTEN	FAM63B	0.431	0	-0.380243548
PTEN	STAM2	0.431	0	0.190159709
PTEN	ZNF484	0.431	0	-0.0199122
PTEN	WDR11	0.431	0	-0.190486235
PTEN	LCA5	0.431	0	-0.384715135
PTEN	KLHL28	0.431	0	0.115578951
PTEN	BTBD7	0.431	0	-0.517972925
PTEN	BTAF1	0.432	0	-0.092808546
PTEN	ZEB1	0.433	0	-0.131948069
PTEN	APC	0.434	0	-0.370966449
PTEN	SH3D19	0.434	0	-0.279732909
PTEN	IL6ST	0.434	0	0.108446485
PTEN	HIF1AN	0.435	0	-0.193347914
PTEN	ARID4A	0.435	0	-0.127526946
PTEN	C10orf46	0.435	0	-0.004894858

PTEN	MINPP1	0.435	0	0.043137685
PTEN	PTP4A2	0.436	0	0.341163674
PTEN	C10orf28	0.436	0	-0.232790412
PTEN	FAM35B2	0.436	0	-0.153202555
PTEN	ERBB2IP	0.436	0	-0.377269647
PTEN	5-Mar	0.439	0	0.110740268
PTEN	REEP3	0.439	0	0.059116001
PTEN	SFRS2IP	0.439	0	0.242326684
PTEN	LPP	0.439	0	-0.162481923
PTEN	SETD7	0.44	0	0.144223762
PTEN	SLC35D1	0.441	0	-0.273092233
PTEN	IKZF5	0.441	0	-0.000114242
PTEN	NEDD4	0.442	0	-0.182664031
PTEN	FAM35A	0.444	0	-0.252974631
PTEN	MAN2A1	0.444	0	-0.020187875
PTEN	CREB1	0.445	0	-0.114158064
PTEN	AFF4	0.445	0	-0.069327591
PTEN	FEM1C	0.445	0	-0.149330642
PTEN	TCTN3	0.446	0	0.179866377
PTEN	C10orf26	0.446	0	0.113949814
PTEN	SEL1L	0.448	0	-0.035441899
PTEN	RFX7	0.45	0	-0.041107973
PTEN	GTF2A1	0.452	0	-0.000683522
PTEN	ITGAV	0.453	0	0.054928348
PTEN	YLPM1	0.453	0	-0.369710479
PTEN	KIAA0494	0.455	0	0.268910112
PTEN	ANO6	0.458	0	-0.064861505
PTEN	CPEB3	0.458	0	-0.441025609
PTEN	RBM43	0.458	0	-0.045172518
PTEN	UHRF1BP1L	0.458	0	-0.152787701
PTEN	DOCK1	0.458	0	-0.115314974
PTEN	TBC1D12	0.458	0	-0.329343687
PTEN	CHUK	0.459	0	-0.213080077
PTEN	SPTLC2	0.462	0	-0.105043564
PTEN	RAB11FIP2	0.463	0	-0.306322039
PTEN	ATRX	0.465	0	-0.149227263
PTEN	DTWD2	0.465	0	-0.073491767
PTEN	ENTPD7	0.466	0	-0.018006173
PTEN	TRIP11	0.467	0	-0.246605402
PTEN	TNKS2	0.467	0	0.108745604
PTEN	KILLIN	0.469	0	-0.476950882

PTEN	PDZD8	0.469	0	-0.099403175
PTEN	PJA2	0.469	0	-0.105185854
PTEN	TRUB1	0.472	0	-0.139074351
PTEN	SP1	0.475	0	-0.083014429
PTEN	LEPROT	0.475	0	-0.190286151
PTEN	ATG2B	0.476	0	-0.184488693
PTEN	GBF1	0.477	0	0.001417435
PTEN	SHOC2	0.478	0	0.004973386
PTEN	EXOC6	0.478	0	0.115177643
PTEN	LCOR	0.481	0	-0.293554709
PTEN	ATE1	0.482	0	-0.338241291
PTEN	TM9SF3	0.483	0	0.035000812
PTEN	IDE	0.486	0	-0.009471593
PTEN	GNG12	0.487	0	-0.098036727
PTEN	MFAP3	0.487	0	0.167346473
PTEN	C10orf118	0.494	0	0.004255647
PTEN	MYOF	0.494	0	0.001033796
PTEN	HECTD2	0.495	0	-0.309053254
PTEN	ATAD1	0.498	0	0.070075597
PTEN	FAM178A	0.498	0	-0.02432713
PTEN	BMPR2	0.499	0	-0.020517866
PTEN	TCF12	0.504	0	-0.129238456
PTEN	WAPAL	0.509	0	0.015362338
PTEN	NHLRC2	0.509	0	-0.008568124
PTEN	SLK	0.509	0	-0.156915133
PTEN	ATF7	0.519	0	-0.249986991
PTEN	SEC23IP	0.519	0	0.100233959
PTEN	FAM160B1	0.54	0	-0.259708093
PTEN	PTENP1	0.933	0	-0.670486037

Table S4: List of shared miRNAs of PTEN-PTENP1 pairs in BRCA along with their respective SoCeR.

Common miRNA	SoCeR
hsa-miR-454	-1.31E+01
hsa-miR-219-5p	-1.87E+00
hsa-miR-769-3p	-2.81E+01
hsa-miR-216a	-6.11E-01
hsa-miR-214	-3.20E+01
hsa-miR-642	-4.88E+00
hsa-miR-125a-3p	7.77E+02
hsa-miR-432*	2.35E+00
hsa-miR-653	-1.68E+01
hsa-miR-495	-7.12E+00
hsa-miR-1179	-1.91E-01
hsa-miR-519a	-2.43E+00
hsa-miR-193b	-1.34E+02
hsa-miR-582-3p	-1.73E+02
hsa-miR-891b	-9.27E-03
hsa-miR-519d	-1.35E-01
hsa-miR-106a	-1.70E+01
hsa-miR-542-3p	-2.10E+02
hsa-miR-130a*	-8.47E+01
hsa-miR-138-2*	3.87E-01
hsa-miR-545	-9.62E-01
hsa-miR-539	-1.09E+01
hsa-miR-198	-1.24E-02
hsa-miR-527	-3.90E-01
hsa-miR-30a*	-5.41E+04
hsa-miR-944	-1.44E+00
hsa-miR-100*	-2.76E+03
hsa-miR-101*	-8.24E+03
hsa-miR-29a	-4.26E+03
hsa-miR-29b-1*	-2.62E+02
hsa-miR-298	-1.22E-03
hsa-miR-338-3p	-2.80E+02
hsa-miR-17	-5.49E+02
hsa-miR-19a	-2.51E+01
hsa-miR-20a	-1.69E+02
hsa-miR-19b	-1.38E+02
hsa-miR-22	-5.97E+04
hsa-miR-548a-3p	-1.40E-03

hsa-miR-586	-3.24E-03
hsa-miR-1193	-1.02E-02
hsa-miR-589*	-5.54E+01
hsa-miR-33a*	-2.15E+01
hsa-miR-3122	-2.37E-03
hsa-miR-507	-2.54E-02
hsa-miR-93	-3.94E+03
hsa-miR-188-5p	-3.36E+00
hsa-miR-3177	-8.52E-02
hsa-miR-323b-3p	-6.02E+00
hsa-miR-3176	-2.77E-01
hsa-miR-584	-4.43E+01
hsa-miR-340	-2.32E+01
hsa-miR-486-5p	-3.13E-02
hsa-miR-577	-1.67E+01
hsa-miR-580	-2.64E-01
hsa-miR-579	-7.31E-01
hsa-miR-524-5p	-3.63E-02
hsa-miR-219-2-3p	-1.15E-01
hsa-miR-548e	-4.35E-01
hsa-miR-3148	-1.79E-02
hsa-miR-20b	-3.48E+01
hsa-miR-3153	-4.71E-03
hsa-miR-590-3p	-1.89E+01
hsa-miR-1238	-1.70E-02
hsa-miR-616	-3.68E+00
hsa-miR-603	-1.43E-02
hsa-miR-10b	-6.34E+04
hsa-miR-130a	-7.73E+01
hsa-miR-10a	-2.95E+04
hsa-miR-518a-5p	-2.77E-01
hsa-miR-301a	-2.27E+01
hsa-miR-106b	-5.48E+02
hsa-miR-29c	-1.63E+03
hsa-miR-520d-5p	-1.28E-01
hsa-miR-16-1*	-6.46E+02
hsa-miR-628-3p	-4.37E+01
hsa-miR-622	-1.28E-02
hsa-miR-664	-3.39E+01
hsa-miR-382	-2.26E+01
hsa-miR-378*	-1.76E+02

hsa-miR-3163	-7.19E-03
hsa-miR-153	-5.75E+01
hsa-miR-513a-3p	-7.98E-03
hsa-miR-301b	-1.95E+00
hsa-miR-500	-1.83E+02
hsa-miR-374b	-5.63E+01
hsa-miR-130b	-2.88E+01
hsa-miR-30e*	-1.29E+04
hsa-miR-10b*	-6.40E+04
hsa-miR-34a*	-1.50E+02
hsa-miR-7-2*	-7.75E-01
hsa-miR-1305	-1.09E-01
hsa-miR-154	-6.46E+00
hsa-miR-30d*	-6.49E+03
hsa-miR-498	-2.12E-02
hsa-miR-515-3p	-6.75E-02
hsa-miR-3137	-6.46E-02
hsa-miR-3136	-6.09E-01
hsa-miR-374a	-7.65E+02
hsa-miR-29b	-2.82E+02
hsa-miR-744*	-4.74E+01
hsa-miR-548f	-4.33E-04
hsa-miR-514b-3p	-2.63E-02
hsa-miR-335*	-8.11E+01
hsa-miR-1264	-4.07E-03
hsa-miR-570	-4.31E-01
hsa-miR-655	-1.93E+00
hsa-miR-525-5p	-3.43E-01
hsa-miR-519b-3p	-4.66E-02
hsa-miR-526b*	-2.30E+00
hsa-miR-520a-5p	-7.01E-01
hsa-miR-519c-3p	-2.43E-01
hsa-miR-1272	-8.06E-04
hsa-miR-193a-3p	-4.23E+02
hsa-miR-3171	-1.16E-03
hsa-miR-337-3p	-3.22E+01
hsa-miR-323-3p	-4.65E+00
hsa-miR-425	-4.26E+02
hsa-miR-1294	-1.39E-01
hsa-miR-514	-9.35E-01
hsa-miR-205	-2.76E+03

hsa-miR-509-3p	-1.27E+00
hsa-miR-7-1*	-3.75E+01
hsa-miR-511	-8.82E+00
hsa-miR-488*	-1.87E-01
hsa-miR-4310	-4.80E-03
hsa-miR-875-5p	-4.06E-03
hsa-miR-889	-1.12E+01

Table S5: The shared miRNAs of VCAN-CD34 pairs in LIHC along with their corresponding scores.

Common miRNA	SoCeR
hsa-miR-552	4.74E+01
hsa-miR-548f	6.96E-03
hsa-miR-708	1.14E+01
hsa-miR-16-1*	3.73E+02
hsa-miR-551b*	5.47E+00
hsa-miR-545	4.37E-01
hsa-miR-224*	6.86E+01
hsa-miR-421	4.86E+00
hsa-miR-92a	1.50E+03
hsa-miR-367	2.75E-03
hsa-miR-30e*	9.32E+03
hsa-miR-548k	1.03E-02
hsa-miR-1258	-1.59E-01
hsa-miR-548h	-1.81E-04
hsa-miR-323b-3p	4.51E+00
hsa-miR-140-5p	1.73E+03
hsa-miR-548d-5p	-5.43E-02
hsa-miR-1290	3.42E-03
hsa-miR-330-3p	1.72E+01
hsa-miR-578	-9.73E-02
hsa-miR-107	-1.50E+02
hsa-miR-142-5p	1.54E+03
hsa-miR-103	-2.96E+04
hsa-miR-922	-2.89E-03
hsa-miR-616	4.65E+00
hsa-miR-875-3p	1.38E-01
hsa-miR-30d*	9.09E+03
hsa-miR-200a*	-1.30E+02
hsa-miR-138-2*	1.97E-01
hsa-miR-431	1.48E+01
hsa-miR-186*	2.30E+02
hsa-miR-30c-2*	7.46E+02
hsa-miR-9	1.78E+02
hsa-miR-200b*	-1.19E+02
hsa-miR-30a*	1.80E+04
hsa-miR-631	9.35E-03
hsa-miR-522	1.17E+00
hsa-miR-28-5p	4.45E+03

hsa-miR-30c-1*	2.72E+00
hsa-miR-513a-3p	2.06E-02
hsa-miR-29b-2*	3.22E+02
hsa-miR-548l	-3.42E-02
hsa-miR-548i	-6.76E-05
hsa-miR-548e	4.30E-01
hsa-miR-548j	-4.60E-03
hsa-miR-17*	1.37E+03
hsa-miR-590-3p	-1.80E+01
hsa-miR-548a-5p	-1.89E-02
hsa-miR-548w	-7.75E-04
hsa-miR-559	-1.33E-02
hsa-miR-548b-5p	-1.13E+00
hsa-miR-548a-3p	2.43E-02
hsa-miR-128	2.87E+01

Table S6: The shared miRNAs of VCAN-FN1 pairs in LIHC along with their corresponding scores.

Common miRNA	SoCeR
hsa-miR-552	6.40E+01
hsa-let-7f-1*	2.23E+01
hsa-miR-136	7.10E+01
hsa-let-7a*	6.83E+03
hsa-let-7b*	7.01E+03
hsa-miR-767-5p	3.49E+01
hsa-miR-126*	3.79E+03
hsa-miR-409-3p	6.07E+01
hsa-miR-573	1.65E-01
hsa-miR-16-1*	4.84E+02
hsa-miR-647	3.06E-03
hsa-miR-656	1.07E+00
hsa-miR-1252	1.75E-02
hsa-miR-3121	2.06E-02
hsa-miR-219-1-3p	6.24E+00
hsa-miR-892a	3.14E-01
hsa-miR-548s	6.50E-01
hsa-miR-335*	1.22E+02
hsa-miR-628-3p	1.12E+01
hsa-miR-622	1.29E-02
hsa-miR-9	6.43E+02
hsa-miR-1272	-3.18E-04
hsa-miR-3165	4.87E-03
hsa-miR-3164	3.51E-02
hsa-miR-33a*	4.16E+01
hsa-miR-570	9.73E-01
hsa-miR-3163	6.72E-02
hsa-miR-323b-3p	7.83E+00
hsa-miR-340	5.07E+01
hsa-miR-3190-5p	1.61E-01
hsa-miR-616*	9.90E+00
hsa-miR-887	4.31E+00
hsa-miR-582-5p	2.72E+02
hsa-miR-579	1.03E+00
hsa-miR-200a*	1.71E+02
hsa-miR-20a*	8.10E+02

hsa-miR-186	3.15E+02
hsa-miR-27b	2.28E+03
hsa-miR-195*	3.01E+00
hsa-miR-539	2.00E+01
hsa-miR-30c-2*	7.09E+02
hsa-miR-224	1.99E+02
hsa-miR-200b*	1.55E+02
hsa-miR-511	1.33E+01
hsa-miR-548o	7.30E-01
hsa-miR-944	2.45E+00
hsa-miR-27a	6.90E+02
hsa-miR-30c-1*	2.63E+00
hsa-miR-551b*	9.20E+00
hsa-miR-548l	2.39E-02
hsa-miR-138-2*	2.13E-01
hsa-miR-15b*	2.88E+02
hsa-miR-1276	3.69E-01
hsa-miR-3148	3.90E-03
hsa-miR-655	5.46E+00
hsa-miR-3153	6.03E-03
hsa-miR-548t	3.86E-02
hsa-miR-875-3p	1.86E-01
hsa-miR-590-3p	2.29E+01
hsa-miR-607	1.77E-01
hsa-miR-708*	1.03E+01
hsa-miR-33b	8.37E-01
hsa-miR-625*	6.31E+01
hsa-miR-628-5p	1.10E+01
hsa-miR-128	9.04E+01
hsa-miR-4326	6.79E+00
hsa-miR-1323	7.13E+00

Table S7: The percentage of predicted pairs lying in the cut-off range across cancers.

Cancers	Percentage
ACC	67.85
BLCA	66.71
BRCA	65.09
CESC	67.33
CHOL	66.39
COAD	67.75
DLBC	68.21
ESCA	66.42
HNSC	65.08
KICH	61.27
KIRC	62.72
KIRP	61.72
LAML	70.79
LGG	64.50
LIHC	66.52
LUAD	65.38
LUSC	67.68
MESO	64.97
OV	66.59
PAAD	65.85
PCPG	69.39
PRAD	62.94
READ	68.36
SARC	64.87
SKCM	70.36
STAD	65.97
TGCT	73.67
THCA	65.36
THYM	72.68
UCEC	67.91
UCS	70.78
UVM	69.77
